# Epitope Generation Gateway: A biological context-dependent approach to cancer vaccine development

**DOI:** 10.1093/gigascience/giag095

**Published:** 2026-10-01

**Authors:** Luca Mannino, Michele Fratello, Jacopo Chiaro, Federica D’Alessio, Emanuele Di Lieto, Minna Niittykoski, Angela Serra, Seppo Ylä-Herttuala, Vincenzo Cerullo, Antonio Federico, Dario Greco

**Affiliations:** Faculty of Medicine and Health Technology, Tampere University, Arvo Ylpön katu 34, Kauppi, 33520, Tampere, Finland; Faculty of Medicine and Health Technology, Tampere University, Arvo Ylpön katu 34, Kauppi, 33520, Tampere, Finland; Department of Pharmaceutical Biosciences, Faculty of Pharmacy ImmunoViroTherapy Lab, Drug Research Program, University of Helsinki, Viikinkaari 5 E, Viikki, 00790, Helsinki, Finland; Helsinki Institute of Life Science (HiLIFE), University of Helsinki, Viikinkaari 5 D, Viikki, 00790, Helsinki, Finland; Translational Immunology Program (TRIMM), Faculty of Medicine Helsinki University, University of Helsinki, Haartmaninkatu 8, Meilahti, 00290, Helsinki, Finland; Digital Precision Cancer Medicine Flagship (iCAN), University of Helsinki. Haartmaninkatu 8, Meilahti, 00290, Helsinki, Finland; Department of Pharmaceutical Biosciences, Faculty of Pharmacy ImmunoViroTherapy Lab, Drug Research Program, University of Helsinki, Viikinkaari 5 E, Viikki, 00790, Helsinki, Finland; Helsinki Institute of Life Science (HiLIFE), University of Helsinki, Viikinkaari 5 D, Viikki, 00790, Helsinki, Finland; Translational Immunology Program (TRIMM), Faculty of Medicine Helsinki University, University of Helsinki, Haartmaninkatu 8, Meilahti, 00290, Helsinki, Finland; Digital Precision Cancer Medicine Flagship (iCAN), University of Helsinki. Haartmaninkatu 8, Meilahti, 00290, Helsinki, Finland; Faculty of Medicine and Health Technology, Tampere University, Arvo Ylpön katu 34, Kauppi, 33520, Tampere, Finland; A.I. Virtanen Institute for Molecular Sciences, University of Eastern Finland, Neulaniementie 2, 70211, Kuopio, Finland; Faculty of Medicine and Health Technology, Tampere University, Arvo Ylpön katu 34, Kauppi, 33520, Tampere, Finland; Tampere Institute for Advanced Study, Tampere University, Kalevantie 4, City Centre Campus, 33100, Tampere, Finland; Division of Pharmaceutical Biosciences, Faculty of Pharmacy, University of Helsinki, Viikinkaari 5 E, Viikki, 00790, Helsinki, Finland; A.I. Virtanen Institute for Molecular Sciences, University of Eastern Finland, Neulaniementie 2, 70211, Kuopio, Finland; Heart Center and Gene Therapy Unit, Kuopio University Hospital, Puijonlaaksontie 2, 70210 Kuopio, Kuopio, Finland; Department of Pharmaceutical Biosciences, Faculty of Pharmacy ImmunoViroTherapy Lab, Drug Research Program, University of Helsinki, Viikinkaari 5 E, Viikki, 00790, Helsinki, Finland; Helsinki Institute of Life Science (HiLIFE), University of Helsinki, Viikinkaari 5 D, Viikki, 00790, Helsinki, Finland; Translational Immunology Program (TRIMM), Faculty of Medicine Helsinki University, University of Helsinki, Haartmaninkatu 8, Meilahti, 00290, Helsinki, Finland; Digital Precision Cancer Medicine Flagship (iCAN), University of Helsinki. Haartmaninkatu 8, Meilahti, 00290, Helsinki, Finland; Department of Molecular Medicine and Medical Biotechnology and CEINGE, Naples University Federico II, Via Sergio Pansini 5, Zona Ospedaliera, 80131, Naples, Italy; Faculty of Medicine and Health Technology, Tampere University, Arvo Ylpön katu 34, Kauppi, 33520, Tampere, Finland; Tampere Institute for Advanced Study, Tampere University, Kalevantie 4, City Centre Campus, 33100, Tampere, Finland; Division of Pharmaceutical Biosciences, Faculty of Pharmacy, University of Helsinki, Viikinkaari 5 E, Viikki, 00790, Helsinki, Finland; Faculty of Medicine and Health Technology, Tampere University, Arvo Ylpön katu 34, Kauppi, 33520, Tampere, Finland; Division of Pharmaceutical Biosciences, Faculty of Pharmacy, University of Helsinki, Viikinkaari 5 E, Viikki, 00790, Helsinki, Finland

**Keywords:** cancer vaccines, neoantigens, epitope prioritisation, personalised immunotherapy, tumour evolution, patient-specific networks, co-expression networks, Snakemake workflow, melanoma

## Abstract

Background: Personalized neoantigen discovery requires tools that not only identify putative epitopes but also prioritize candidates using biological features relevant to tumor fitness and immune escape. Here, we present Epitope Generation Gateway (EGG), a containerized pipeline that streamlines discovery and prioritization across variant types and human leukocyte antigen (HLA) contexts. EGG coordinates established components, Mutect2 for somatic single nucleotide variations (SNVs) and indels, STAR-Fusion for fusion events, ArcasHLA for HLA typing, and pVACtools for epitope sequence prediction, then augments peptide-level scores with biological context. For each patient, EGG builds co-expression and protein–protein interaction networks, uses centrality measures and CRISPR dependency data (DepMap) to flag genes important for tumor fitness, and annotates source genes with gene ontology terms, Hallmarks of Cancer processes, and IntOGen driver status. Results: This multi-layer information supports ranking of candidates that are predicted to be presented and that arise from genes essential to tumor fitness or embedded in key pathways, providing a framework to nominate epitopes whose source genes may be less dispensable under immune pressure. As a case study, we applied EGG to 30 TCGA skin cutaneous melanoma patients, demonstrating end-to-end execution and producing prioritized epitopes that emphasize peptides and alterations in high-centrality, dependency-supported essential genes. Conclusions: Overall, EGG links epitope prioritization to tumor systems biology and evolutionary rationale, prioritizing biologically contextualized cancer vaccine candidates from SNVs, indels, fusions, and splicing-derived epitopes while streamlining dataset analysis. EGG is available from GitHub at https://github.com/fhaive/epitope_generation_gateway.

## Introduction

Cancer immunotherapy has emerged as a dominant therapeutic modality in modern oncology, fundamentally transforming treatment paradigms across multiple malignancies. Among the most promising immunotherapeutic approaches, personalized neoantigen vaccines exploit tumor-specific mutations to generate highly targeted immune responses, offering unprecedented precision in cancer treatment [1, 2]. Unlike conventional cytotoxic therapies such as chemotherapy and radiotherapy, which broadly target rapidly dividing cells, neoantigen vaccines provide enhanced tumor specificity and may mitigate the systemic toxicity associated with these standard interventions [3].

The therapeutic efficacy of cancer vaccines essentially depends on their capacity to trigger robust immune responses while maintaining tumor specificity, minimizing off-target effects that could compromise patient safety [4]. Current computational pipelines can identify candidate neo-antigens from single nucleotide variations (SNVs), small insertions or deletions (indels), gene fusions, and aberrant splicing events. Early generation tools primarily targeted SNVs and indels with exclusive focus on Major Histocompatibility Complex (MHC) class I ligand prediction, addressing only CD8⁺ T-cell responses [5–7]. Subsequent frameworks expanded to include gene-fusion breakpoints and now commonly support both class I and class II molecule prediction, engaging both cytotoxic and helper T-cell responses. The most recently developed tools further capture epitopes from aberrant splicing events [8–10]. However, existing approaches largely overlook the functional context of neoantigens, failing to integrate them within their broader transcriptomic landscape or consider the biological significance of their source genes. Moreover, vaccine efficacy is challenged by the tumor intrinsic capacity for immune evasion and the functional importance of targeted mutations. Recent findings by Al Bakir et al. demonstrate that tumors can eliminate immunogenic targets through clonal deletion under selective pressure, emphasizing the critical need to prioritize mutations in functionally indispensable genes [11]. Effective neoantigen selection must therefore consider gene essentiality: alterations in genes essential for malignant fitness or in hub genes anchoring critical signaling pathways are less likely to be purged during tumor evolution, thus representing more durable vaccine targets. Gene co-expression networks provide a practical framework for inferring such functional importance, as network centrality metrics correlate with gene essentiality and the evolutionary cost of mutational escape [12].

Systems biology treats cancer as a dynamic, complex disease, where phenotype arises from perturbed interactions rather than isolated genes. Network models are widespread tools for representing and analyzing complex systems such as tumor biology. Yet, most neoantigen pipelines still emphasize sequence level features for epitope prioritization (such as peptide MHC binding) and give limited weight to the broader context that governs whether a candidate peptide is generated, displayed, and retained by the tumor. Recent spatial approaches such as SpatialSNV further illustrate that tumor SNVs can vary across local tumor regions and microenvironmental contexts [13]. Evidence shows that subcellular origin influences both presentation and outcome: neoantigens from plasma membrane proteins are disproportionately presented and have been associated with improved responses to immune checkpoint blockade [14, 15]. At the same time, tumors can evade immunity by purging dispensable targets during evolution, underscoring the value of prioritizing alterations in vital genes, which are less likely to be lost [11, 16, 17].

To address these gaps, we developed Epitope Generation Gateway (EGG), an automated workflow that integrates standard callers and predictors: Mutect2 [18] for somatic SNVs/indels, STAR-Fusion [19] for fusion events, ArcasHLA [20] for HLA typing, and pVACtools [21–23] for class I/II epitope prediction. EGG augments sequence level scoring with essentiality aware features and places each candidate in its biological setting by building patient specific co-expression/protein–protein-interaction (PPI) networks. For prioritization, EGG integrates multiple layers of evidence regarding the source gene. These include patient specific co-expression/PPI centrality measures as network derived indicators of gene importance or essentiality, complemented by CRISPR knockout based dependency scores from DepMap [24] that provide experimentally grounded, though not patient-specific, gene essentiality information. Candidate genes are further annotated with gene ontology (GO) [25] terms and MSigDB Cancer Hallmarks [26] gene sets to capture pathway-level context, as well as with IntOGen [27] annotations to identify established cancer driver genes. Taken together, these data-driven and knowledge based features help to prioritize neoantigens that are not only predicted to be presented but also arise from genes whose functions are critical to tumor fitness and therefore less likely to be lost through immune evasion. EGG’s design is intentionally holistic: it integrates patient specific networks with curated biological knowledge (GO, MSigDB Cancer Hallmarks, and DepMap) and presentation relevant attributes (cell membrane localization). The goal is a bigger-picture view of each epitope linking peptide level scores to tumor systems biology so that selected targets are immunologically plausible, and evolutionarily durable. EGG is available from GitHub [28], registered in bio.tools (biotoolsID: epitope_generation_gateway) [29] and SciCrunch (RRID: SCR_028792) [30], and archived as workflow in WorkflowHub [31].

## Material and methods

### Data acquisition and preprocessing

Matched tumor RNA-seq, tumor DNA (WES), and normal DNA samples were retrieved from The Cancer Genome Atlas (TCGA) [32] for 30 patients diagnosed with cutaneous melanoma (TCGA-SKCM study code). Patients were randomly selected from the subset with available primary tumor RNA-seq data, corresponding tumor DNA, and matched normal DNA, explicitly excluding metastatic tissue. Datasets were retrieved using the Genomic Data Commons (GDC) client (gdc-client) [33], leveraging unique patient identifiers (UUIDs).

Sequencing alignment files in BAM format were downloaded and converted to paired-end FASTQ format to facilitate compatibility with the EGG pipeline. Conversion was performed using SAMtools (v1.21) [34, 35], which involved: splitting BAM files by read group (RG), sorting reads by name and extracting paired reads as compressed FASTQ files. These steps ensured the correct input data format for subsequent processing modules.

### Read quality control and trimming

Raw sequencing reads from tumor RNA-seq, tumor DNA, and matched normal DNA samples (all in FASTQ format) were processed using FastQC (v0.11.9) [36] and fastp (v 0.23.2) [37] to assess and improve read quality. Quality assessment was performed with FastQC to identify issues such as base quality degradation. Read trimming and filtering were subsequently carried out using fastp, which automatically detected and removed adapter sequences and excluded reads with poor quality metrics. Specifically, reads containing bases with Phred scores below 20 or with more than 30% unqualified bases were discarded. Additionally, reads shorter than 30 base pairs were removed. A sliding window quality trimming approach with a mean quality threshold of 20 was applied. All trimming settings were specified in a centralized YAML configuration file (QC_filtering_config_0.yaml), allowing users to easily adapt the preprocessing pipeline to different requirements.

### RNA-seq processing, fusion detection, and HLA typing

Trimmed cancer RNA-seq reads were processed using STAR-Fusion (v1.15.0) [19], which aligned reads and detected gene fusion events using the GRCh38/GENCODE v37 CTAT genome reference [38, 39]. This tool generated both fusion transcript calls and alignment files for downstream quantification. Reference genomes and transcript annotations (GRCh38/GENCODE v37) were acquired from the CTAT genome library provided by STAR-Fusion [40]. These resources were automatically retrieved and managed by the pipeline. STAR-Fusion was used with default filters, which require at least one junction read and one spanning read (or ≥2 junction reads if no spanning support) and a minimum expression support of FFPM (fusion fragments per million total reads) ≥0.1.

Gene expression quantification was performed with the featureCounts function from the Rsubread Bioconductor package (v2.0.8) [41, 42], using the same annotation reference to compute raw read counts for each gene in paired-end mode. These count matrices served as input for sample specific co-expression network production implemented in later stages of the pipeline.

To enable personalized neoepitope prediction, HLA typing was conducted using arcasHLA (v0.5) [20], which genotypes both MHC class I and class II loci from RNA-seq data. After sorting the aligned BAM files with SAMtools (v1.21) [34, 35], unmapped reads were extracted and used to predict HLA alleles. The output was converted into a format compatible with pVACtools to support downstream MHC binding predictions.

Both STAR-Fusion and arcasHLA were executed within Docker containers, ensuring reproducibility and consistency across environments. Other steps, including featureCounts and BAM sorting, were integrated into the Snakemake [43] pipeline through dedicated Conda environments.

### Fusion epitopes prediction

Post-alignment RNA fusion calls were processed for neoepitope prediction using the pVACfuse module of pVACtools (v5.3) [22], which integrates gene fusion annotations with HLA typing and MHC binding prediction. Fusion predictions, generated by STAR-Fusion, were first annotated using AGFusion (v1.4.3) [44] with the Ensembl GRCh38 release 103 transcript database [45] and Pfam-A domain annotations [46]. AGFusion resolved coding fusion junctions and transcript models, generating protein sequences suitable for downstream analysis.

These annotated fusion sequences were then passed to pVACfuse, which identified candidate fusion-derived neoantigens using a user-defined panel of MHC class I and II binding prediction algorithms (MHCNuggetsI and MHCnuggetsII) [47]. HLA alleles were automatically parsed from ArcasHLA outputs however in the practical use of the pipeline they can be optionally provided as pre-existent files if the user wants to calculate them using different methods. Parameters such as epitope length (e1, e2), binding affinity thresholds, and the number of prediction threads were specified in the Snakemake pipeline and can be modified by the user by using different pVACtools options including different prediction algorithms.

All fusion epitope analyses were executed within Docker containers to ensure version consistency and reproducibility across environments. The containerized pipeline streamlined fusion annotation, neoantigen calling, and intermediate file handling, enabling high-throughput and customizable analysis per sample.

### SNV and indel detection in tumor and normal DNA samples

All genomic analyses were conducted using the GRCh38 reference genome [39]. The reference FASTA file, its associated dictionary, and BWA index files were downloaded from the UCSC Genome Browser [48] and generated using SAMtools (v1.16), Genome Analysis Toolkit (GATK, v4.5.0.0) [49, 50] CreateSequenceDictionary, and BWA index [51], respectively. Additional resources used in variant calling included a dbSNP [52] VCF (Broad Institute, dbSNP build 138), the gnomAD allele frequency panel [53], and the GATK-provided Panel of Normals (PON). These files were fetched from Broad Institute cloud repositories [54] and indexed with GATK and tabix [55] as needed.

Trimmed DNA-seq reads were processed following the GATK best practices pipeline for short variant discovery. Reads were aligned to the GRCh38 reference genome using BWA-MEM (v0.7.17) [56], with RG headers automatically extracted from the FASTQ file headers to include metadata including flowcell ID, lane, and barcode. These values were parsed directly from the first line of each FASTQ file to populate the RG tags used in alignment. The resulting alignments were sorted by coordinate using SAMtools.

To handle multi lane sequencing data, lane specific BAM files for each sample were merged using Picard (v2.27.4) MergeSamFiles [57]. Duplicate reads were marked using GATK MarkDuplicatesSpark to mitigate polymerase chain reaction (PCR) induced biases. Base quality scores were recalibrated using BaseRecalibrator and ApplyBQSR with known variant sites from dbSNP as training data. These steps produced high-confidence, analysis-ready BAM files per sample.

Alignment metrics, including overall mapping quality and insert size distribution, were collected using GATK CollectAlignmentSummaryMetrics and CollectInsertSizeMetrics respectively. BAM files were indexed using SAMtools to enable efficient access during variant calling and downstream analysis. This preprocessing pipeline was applied to tumor and matched normal DNA samples for each patient, ensuring consistency and compatibility for downstream somatic variant calling with tools such as Mutect2 and HaplotypeCaller.

Germline variants were called on normal DNA samples using GATK HaplotypeCaller, which performs local de novo assembly of haplotypes in regions showing evidence of variation. Raw variant calls were separated into SNPs and indels, followed by hard filtering based on standard GATK quality metrics (e.g. QD, FS, MQ, and SOR). Filter-passing variants were selected, sorted, compressed, and concatenated into a single filtered germline VCF file per sample using BCFtools (v1.16) [34] and tabix utilities. These high-confidence germline calls were retained for downstream annotation and comparison against somatic variants.

Somatic variants were identified using GATK Mutect2 by jointly analyzing tumor and matched-normal BAM files per sample. The analysis incorporated a germline resource (gnomAD) and a PON to reduce false positives. Mutect2 output included both raw variant calls and strand orientation artifact data (F1R2).

To refine variant calls, contamination estimates were calculated using GATK GetPileupSummaries and CalculateContamination, followed by orientation bias modeling with LearnReadOrientationModel. Final variant filtering was carried out using FilterMutectCalls, integrating all calculated metrics as additional flags in the VCF for each sample.

### Genomic variant annotation and epitope prediction

To enable downstream neoepitope prediction using pVACtools, we performed additional annotation and preprocessing of somatic and germline variant calls. Variant effect predictor (VEP, Ensembl release 103) [58] was used to functionally annotate VCF files from somatic and germline variant calling workflows. VEP was configured in offline mode using the GRCh38 cache and a subset of plugins (e.g. Frameshift, Wildtype) downloaded from the Ensembl GitHub repository. Annotations were run via command-line within a Docker environment using the griffithlab/pvactools container (v5.3). The pipeline was configured to automatically pull the Docker image if not already present. Somatic VCFs (from Mutect2) and filtered germline VCFs (from HaplotypeCaller) were annotated with VEP, producing functionally enriched VCF files for subsequent pVACseq input. While downstream neoepitope analysis focused on somatic mutations, filtered germline variants were obtained to support phasing. These variants were merged with somatic calls and jointly phased using GATK ReadBackedPhasing. This step allowed identification of compound heterozygous or cis/trans configured variants that could influence peptide sequences derived from cancer genomes.

In parallel, gene expression levels were quantified from tumor RNA-seq data using Kallisto (v0.46.1) [59], a lightweight pseudoalignment-based transcript quantifier. A Kallisto index was built from Ensembl cDNA reference transcripts (release 103), and tumor RNA reads from multiple sequencing lanes were jointly quantified. The resulting expression data was integrated into the annotated somatic VCF using vcf-expression-annotator (vatools package v5.2.0), aligning expression values with corresponding variants.

To generate VCFs compatible with pVACtools, tumor-only variants were extracted using GATK SelectVariants. To enable inclusion of germline variants in neoepitope screening, sample identifiers in the germline VCFs were harmonized with the tumor VCF using BCFtools (v1.17). Germline and tumor variants were then merged using GATK CombineVariants and sorted using Picard SortVcf. These combined VCFs were phased using GATK ReadBackedPhasing and re-annotated with VEP to account for phasing effects on coding consequence.

Human leukocyte antigen (HLA) types were obtained via ArcasHLA as described earlier. HLA types were automatically extracted from ArcasHLA genotype JSON files and formatted into a pVACtools compatible input structure. Alternatively, users have the option to supply their own HLA typing externally.

The primary pVACseq analysis was then performed using the pvacseq run command inside the griffithlab/pVACtools Docker container (v5.3). The workflow supported all pVACseq-compatible MHC binding prediction algorithms, and for this analysis specifically used MHC_nuggetsI, and MHCnuggetsII. Both phased variant calls and Kallisto derived RNA expression data were supplied to enhance neoepitope prioritization. All pVACseq parameters are user configurable by modifying the Snakemake script directly. For this analysis, we used the following key parameters: –epitope-lengths 8–15 for class I predictions and –epitope-lengths 11–30 for class II, along with –depth 1000, and –phased-proximal-variants-vcf to incorporate phasing effects. For tumor-purity-dependent tiering, we retained the pVACseq default tumor purity value of 1.0; this parameter is user-configurable and can be adjusted when sample-specific tumor purity estimates are available. In this case study, retaining tumor purity = 1.0 provided a conservative variant allele frequency (VAF) based parameter for identifying tumor-relevant somatic candidates. Somatic SNV and indel calls were generated with Mutect2 followed by Mutect2 post-filtering. For the proof-of-concept analysis presented here, both PASS and non-PASS Mutect2 records were retained as pVACseq input to preserve a broad candidate mutation pool before downstream prioritization. To assess the impact of this choice, we annotated final somatic epitope candidates with the VCF FILTER status of the originating variant and repeated the prioritization using PASS-only somatic pVACseq inputs. This sensitivity analysis showed that non-PASS calls contributed minimally to the final prioritized candidates and did not materially affect the prioritized gene set. PASS-only filtering can be applied before pVACseq when a higher-specificity input set is desired. Full details of the PASS-only sensitivity analysis are provided in Additional file 3.Mutect2 and STAR-Fusion were selected as pragmatic single-caller defaults for somatic SNV/indel and fusion detection, respectively. This choice was intended to keep the case-study workflow lightweight, reproducible, and computationally tractable, while relying on established tools with broad use in cancer genomics. Mutect2 is used in the GATK somatic short-variant discovery workflow for SNV and indel detection, and is widely regarded as an established, reliable somatic variant caller in cancer genomics [18, 60]. STAR-Fusion is used in the NCI GDC RNA-seq harmonization workflow for transcript-fusion detection and has been reported among the most accurate and fastest fusion callers in benchmark analyses of cancer transcriptomes [61]. We acknowledge that consensus calling strategies can further improve input precision and reduce dependence on caller-specific thresholds; however, benchmarking alternative caller combinations was outside the scope of the present study, which focuses on evaluating EGG as an integrated neoantigen prediction and prioritization framework.

### Alternative splicing-derived neoepitope prediction

Tumor-specific alternative splicing events were identified and processed for neoepitope prediction using a combination of RegTools (v1.0.0) [62] and pVACsplice from the pVACtools suite. First, splice junction annotations were extracted from tumor RNA-seq alignments using RegTools, which identifies novel splice sites and exon-skipping events by comparing observed junctions against reference transcript models.

The analysis was based on the GRCh38/GENCODE v37 reference annotation to identify putative tumor-specific splicing variants. Junction files obtained in output were filtered to retain high-confidence events based on read support thresholds and junction quality metrics.

Identified splicing events were then exploited for neoepitope prediction using pVACsplice (from package pVACtools v5.3), which generates peptide sequences spanning novel exon-exon junctions and predicts their MHC binding potential. The workflow required both the RegTools junction output and the corresponding genomic VCF file containing somatic variants to account for potential interactions between splicing alterations and point mutations.

HLA alleles were provided either from ArcasHLA outputs or user-specified files, maintaining consistency with other neoepitope prediction modules. pVACsplice analysis employed the same MHC binding prediction algorithms used in pVACseq (MHCnuggetsI and MHCnuggetsII) to ensure comparable scoring across different neoantigen sources. Epitope lengths were set to 8–15 amino acids for MHC class I and 11–30 for MHC class II predictions.

All splicing-derived neoepitope analyses were executed within the griffithlab/pvactools Docker container to maintain version consistency and reproducibility. Parameters, including minimum junction read support, binding affinity thresholds, and prediction algorithms were configurable through the centralized Snakemake workflow.

### Patient specific networks generation

Gene level raw read count matrices previously produced in the RNA-seq processing step (described in the RNA-Seq processing, fusion detection, and HLA typing paragraph) were carried forward for patient-specific co-expression network generation. Genes were retained for network construction only if they had at least 15 raw reads in at least 20% of samples; all other genes were removed as a low-expression preprocessing step (in our cohort of 30 samples, this corresponds to requiring detectable expression in at least 6 samples). This filtering step was applied to reduce noise from genes with very low or infrequent expression, which are less likely to support stable correlation estimates in co-expression-based network inference. similar low-expression filtering steps are commonly used in RNA-seq network analysis workflows, including LIONESS-based analyses of TCGA and GTEx data [63, 64].

For downstream network analysis, variance stabilizing transformation (VST) was applied to the expression count using the DESeq2 package (v1.46.0) [65]. This transformation adjusts for the strong relationship between gene expression level and variability, producing a matrix in which genes with different expression ranges can be more reliably compared. The resulting normalized expression matrix was used to construct patient-specific co-expression networks.

To estimate individual level gene co-expression patterns, we applied the LIONESS (Linear Interpolation to Obtain Network Estimates for Single Samples) framework [66]. For a set of S samples, the aggregate network​ was computed using Pearson correlation between all gene pairs $( {i,j} )$ across the full expression matrix.

To estimate the network specific to a sample *q*, we first denote the aggregate network computed across all samples as $e_{ij}^{( \alpha )}$, and the corresponding leave-one-out aggregate network excluding sample *q*as $e_{ij}^{( {\alpha q} )}$. The individual sample network $e_{ij}^{( q )}$was then derived using the LIONESS formula:


\begin{eqnarray*}
e_{ij}^{\left( q \right)} = S\left( {e_{ij}^{\left( \alpha \right)} - e_{ij}^{\left( {\alpha - q} \right)}} \right) + e_{ij}^{\left( {\alpha - q} \right)}.
\end{eqnarray*}

This equation quantifies the contribution of sample *q* to each edge $( {i,j} )$ by interpolating its influence relative to the full dataset. All sample-specific adjacency matrices were stored in sparse matrix format to facilitate downstream graph based analyses.

Following LIONESS based reconstruction, we filtered the sample-specific networks to prioritize biologically interpretable and functionally relevant edges. First, we intersected each network with an extended PPI network the HumanNet-XN v2, an integrated human functional interaction network anchored in PPIs and extended with co-expression, pathway, interolog, and co-citation evidence [67]. HumanNet edges were mapped from Entrez to Ensembl gene identifiers using the org.Hs.eg.db library (v3.18.0) [68].

To construct the filtered networks, we applied a two-step edge selection strategy that combined the extended PPI informed filtering with membrane-focused expansion:

1. PPI-informed edge selection: For each gene, we retain edges with absolute correlation weights ranking in the top 20% among all edges connecting that gene to its PPI-linked neighbors. This approach prioritized patient-specific co-expression patterns that aligned with known PPIs.
2. Membrane-enriched network expansion: To supplement the PPI-filtered network, we incorporated additional edges from the gene co-expression network that involved membrane-associated genes. In the default configuration, candidate membrane-associated edges were defined as edges in which at least one endpoint gene was annotated as membrane associated. These candidate edges were ranked by absolute correlation weight, and the top-ranked edges were added to the PPI-filtered network. The number of membrane-associated edges added was controlled by a configurable proportion, set to 5% by default. In the analysis presented here, this proportion was calculated relative to the number of weighted HumanNet/PPI edges before the per-gene top-20% PPI filtering step. This strategy enriched the network for membrane localized genes while maintaining focus on the strongest co-expression relationships. Membrane associated genes were identified using GO annotations from the following terms and their descendant terms in the GO hierarchy:
  - GO:0005886 (*plasma membrane*).
  - GO:0005887 (*integral component of plasma membrane*).
  - GO:0046658 (*anchored component of membrane*).
  - GO:0009897 (*external side of plasma membrane*).
  - GO:0009986 (*cell surface*).

Membrane gene annotations were obtained from the GAF file provided by the GO Consortium and mapped to Ensembl IDs via UniProt [69] and BioMart (v2.58.0) [70].

The pFINs exhibited a characteristic topology consisting of a dominant largest connected component (LCC) alongside smaller, isolated subnetworks. The LCC encompassed the vast majority of edges and nodes, forming a cohesive structure where genes were interconnected through direct or indirect relationships. The remaining nodes formed small, disconnected clusters or isolated pairs that did not connect to the main component. This topology, with most network connectivity concentrated in a single large component, provided a natural framework for assessing gene importance: genes critical to network integrity would be those whose removal disrupted the LCC structure most severely.

Both the PPI informed edge selection and the membrane-enriched network expansion are optional; the corresponding percentages can be modified via the configuration files or effectively switched off. The final output consisted of sparse adjacency matrices representing filtered, patient-specific co-expression networks enriched for interactions with known biological support and immunological relevance. We have named these networks: Patient Specific Functional Interaction Networks (pFINs) These network matrices were saved in Matrix Market format to ensure compatibility with external graph analysis frameworks.

### Centrality metrics for gene prioritization

To evaluate the functional relevance and network prominence of candidate neoantigen source genes, we calculated node level centrality scores within each sample-specific co-expression network.

To compare the relative connectivity of genes, we computed the Weighted Connectivity Impact (WCI) for each node *v*, defined as the fraction of total network edge weights attributable to its immediate connections.

Where each edge is normalized as


\begin{eqnarray*}
\widetilde {{{w}_{uv}}} = \frac{{\left| {{{w}_{uv}}} \right|}}{{\mathop {\max }\limits_{i,j} \left| {{{w}_{ij}}} \right|}}.
\end{eqnarray*}

The numerator is the sum of normalized absolute weights of all edges incident to *v*:


\begin{eqnarray*}
{\mathrm{strength}}(v) = \sum \limits_{u\in N(v)} \widetilde {{{w}_{uv}}}.
\end{eqnarray*}

WCI calculated as


\begin{eqnarray*}
{\mathrm{WCI}}\left( v \right) = \frac{{{\mathrm{strength}}\left( v \right)}}{{\mathop \sum \nolimits_{e\in E} \widetilde {{{w}_e}}}}.
\end{eqnarray*}

This yields a sample-scaled centrality score between 0 and 1, allowing for cross-sample comparisons of gene connectivity despite differences in total edge weight. Genes with high WCI constitute a disproportionately large proportion of overall network strength.

### PFINs centrality metrics

To assess the topological relevance of neoantigen-associated genes within the pFINs networks, we computed several centrality metrics.

The first centrality metric is the Largest Component Impact (LCI) quantifies a gene’s contribution to the integrity of the network’s LCC obtained from the PPI filtering steps explained above. It is a simple way to combine strength and degree in a comparable way between different networks. For each gene *v*, the LCI was computed by measuring the change in both node connectivity and edge weight upon virtual deletion of that gene:


\begin{eqnarray*}
{\mathrm{Largest\ Component\ Impact}}\left( v \right) = \frac{1}{2}\left[ {\frac{{{{S}_0} - {{S}_v}}}{{{{S}_0}}}} \right] + \frac{1}{2}\left[ {\frac{{{{W}_0} - {{W}_v}}}{{{{W}_0}}}} \right],
\end{eqnarray*}

where:

-
${{S}_0}$ is the number of nodes in the LCC of the original network,
-
${{S}_v}$ is the size of the LCC after removing node *v*,
-
${{W}_0}$ is the sum of normalized absolute edge weights within the original LCC,
-
${{W}_v}$ is the corresponding sum after removing *v*.

This metric captures both structural fragmentation and, in turn, functional disconnection, identifying nodes whose removal disproportionately disrupts network integrity. Conceptually, LCI relates to percolation theory, where the removal of critical nodes can trigger a phase transition that fragments a network into disconnected components. By quantifying the impact of individual gene removals on the LCC, LCI identifies genes that act as critical connectors maintaining network cohesion.

The second centrality metric computed was betweenness centrality, a dynamic node centrality measure that reflects how information propagates through the networks. In addition, we calculated node degree and weighted strength for each gene, with strength defined as the sum of the absolute weights of its incident edges using igraph (v2.0.3) [71].

### Pan-cancer gene essentiality scoring

To incorporate gene biological relevance into neoantigen prioritization, we obtained pan-cancer essentiality scores from the DepMap Public 24Q4 dataset [24]. The CRISPR gene effect matrix (CRISPRGeneEffect.csv) was downloaded from the Broad DepMap Figshare repository (article ID 27993248) and processed in R. This matrix contains Chronos-derived gene effect values that represent copy number corrected knockout fitness effects across cancer cell lines, where more negative values indicate greater cellular dependency [72].

For each gene, we computed the median Chronos gene effect across all available cell lines to derive a single pan-cancer essentiality score (PanCancer_Median_Chronos). Following DepMap guidelines, genes with scores <−0.5 indicate depletion in most cell lines, while scores <−1.0 represent strong essentiality. These pan-cancer essentiality scores were subsequently integrated into the epitopes prioritization workflow to favor epitopes derived from functionally necessary genes.

### Cancer driver gene annotation

To identify neoantigens derived from cancer driver genes, we obtained driver annotations from the IntOGen Drivers compendium (version 20230531) [27]. The compendium file was processed to retain all genes classified as either “activating” (oncogenic; Act) or “loss-of-function" (tumor suppressor; LoF) roles across cancer types. This annotation was integrated into the neoantigen prioritization framework to identify neoantigens originating from genes with established roles in cancer pathogenesis, as mutations in driver genes may be particularly relevant for immune targeting strategies.

### Consensus prioritization

Candidate neoantigens were prioritized using a weighted Borda rank aggregation implemented in R. For each feature, epitopes were ranked according to expected directionality: ascending for Median.MT.IC50.Score and Depmap_survivability_score, and descending for network metrics (Network_Betweenness, Network_Degree, Network_Impact, Network_Strength, and Network_WCI). Feature-specific ranks were converted to Borda points and combined using predefined weights (default: 0.25 each for Median.MT.IC50.Score and Depmap_survivability_score; 0.10 for each network metric). The aggregate Borda score determined the final ordering, with ties resolved by average rank. Missing feature values were ignored and remaining weights renormalized per epitope. The prioritization (features, weights, rank direction, tie rule, and NA handling) is configurable via a YAML file without code changes, allowing emphasis on binding affinity, network centrality, or gene essentiality as needed.

## Results and discussion

### EGG analytical flow

The complexity of cancer immunotherapy requires sophisticated computational approaches that can identify not just any neoantigens, but those most likely able to remain resistant to evolutionary changes in the cancer. To address this challenge, we developed a comprehensive pipeline that integrates both established algorithms to extrapolate tumor specific neoantigens and novel network-based methodologies to improve neoantigen prioritization based on their biological context. On one hand, our pipeline includes quality control, preprocessing, extensive mutational analysis, transcriptomics data analysis with identification of fusion events, alternative splicing, and HLA typing. The additional value in this study is that we developed novel network-based methods to infer the biological context of the identified neoantigens. Such methods include the inference, manipulation and analysis of sample specific molecular networks. We envision that by integrating data driven interactional properties of network models with prior knowledge from multiple public databases, it is possible to achieve a robust and informed prioritization that improves efficacy and streamlines pre-clinical pipelines.

The EGG pipeline comprises seven modules ranging from quality control and data preprocessing to interpretable ranking of neoantigens. The diagram in Fig. 1 provides a detailed overview of the pipeline structure as well as the included tools. The first module, Module 0, is dedicated to quality control and filtering of the raw sequencing reads using fastp [37] for read trimming and fastqc for quality assessment. The subsequent mutation detection module (Module 1A) analyzes paired tumor-normal DNA samples, utilizing Mutect2 [18] for somatic variant identification and HaplotypeCaller [49] for germline variant detection Module 1B, on the other hand, processes trimmed RNA-seq cancer data through three analytical pathways: STAR-Fusion [19] identifies structural variants, arcasHLA [20] determines patient-specific HLA allotypes, and FeatureCounts (from Rsubread) [41] generates expression profiles which are then used in network-based downstream analysis. Somatic variants identified in Module 1A undergo allele-specific epitope prediction in Module 2A, where the pVACseq [23] framework evaluates SNVs and indels for MHC-binding potential. This module also performs preprocessing steps required by pVACseq, including quantification of transcripts with Kallisto [59] and phasing variants by integrating somatic and germline calls to preserve haplotype context. Fusion transcripts detected by STAR-Fusion are analyzed in Module 2B where pVACfuse [22] is used to predict neo-antigens spanning fusion junctions.

**Figure 1:**
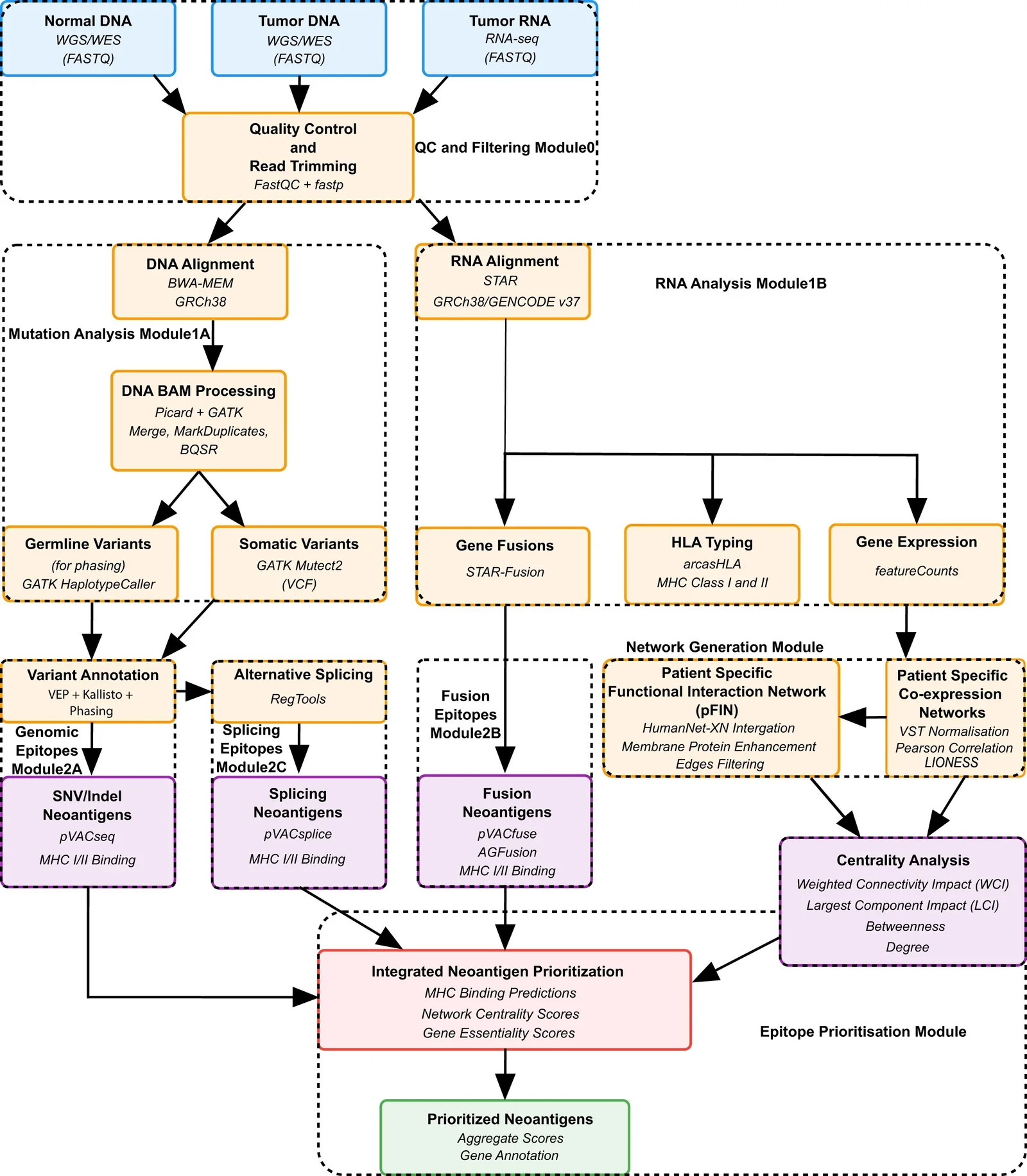
EGG Workflow. The EGG pipeline processes matched normal/tumor DNA (WGS/WES) and tumor RNA-seq data through seven integrated modules. Following quality control, DNA undergoes BWA-MEM [56] alignment and GATK processing for germline and somatic variant calling. RNA processing includes STAR alignment, gene expression quantification, fusion detection (STAR-Fusion), and alternative splicing analysis. HLA typing and variant annotation are performed in parallel. Sample-specific co-expression networks are constructed using filtered HumanNet-XN and LIONESS methodology. Neoantigen prediction targets three categories: SNV/indel, fusion, and splicing neoantigens, all with MHC class I/II binding predictions. Network centrality analysis computes connectivity metrics for integrated prioritization combining MHC binding, centrality scores, and immunogenicity to generate prioritized neoantigens with biological context. Dashed boxes represent pipeline modules subdivision.

Module 2C extracts alternative splicing events by validating variants occurring at splicing sites obtained from Module 1A, via Mutect2, by applying RegTools [62] identifying tumor-specific splicing events, and subsequently processing these through pVACsplice [21] to predict splice junction derived epitopes.

Finally, the single-sample network module constructs personalized co-expression networks using the LIONESS [66] methodology on Pearson gene co-expression matrices derived from the RNA-seq counts generated in Module 1B. The resulting networks are filtered using HumanNet-XN [67] PPI data, and cellular membrane proteins are identified via GO [25]. Network centrality metrics are computed for both filtered and unfiltered patient-specific networks and are used, together with relevant biological information, to prioritize candidates with high potential therapeutic relevance. The network related calculation and generation is carried out by the network generation module while the final prioritization is carried out by the epitope prioritization module. Additional information for the execution of the pipeline, including details on the implementation (e.g. Snakemake workflow [43]), retrieval of required references and tools, and execution of individual modules, is provided in Additional file 1 (Supplementary Material). Additional file 1 also includes an overview of the main third-party tools integrated in EGG and a computational resource benchmark of the main EGG-specific components, focusing on patient-specific network generation and epitope prioritization rather than upstream third-party tools whose resource requirements depend strongly on sequencing depth, library size, and mutation burden.

### Inter sample variability in somatic mutations relevant to neoantigens

To demonstrate EGG’s comprehensive neoantigen prediction capabilities, we analyzed the mutational landscape of 30 TCGA skin cutaneous melanoma (SKCM) samples [32]. As a descriptive view of the mutational landscape of the samples, we highlight the subset of somatic variants identified by Mutect2 that passed GATK’s contamination and quality filters and were annotated as protein-coding, cancer-specific alterations (Fig. 2).

**Figure 2:**
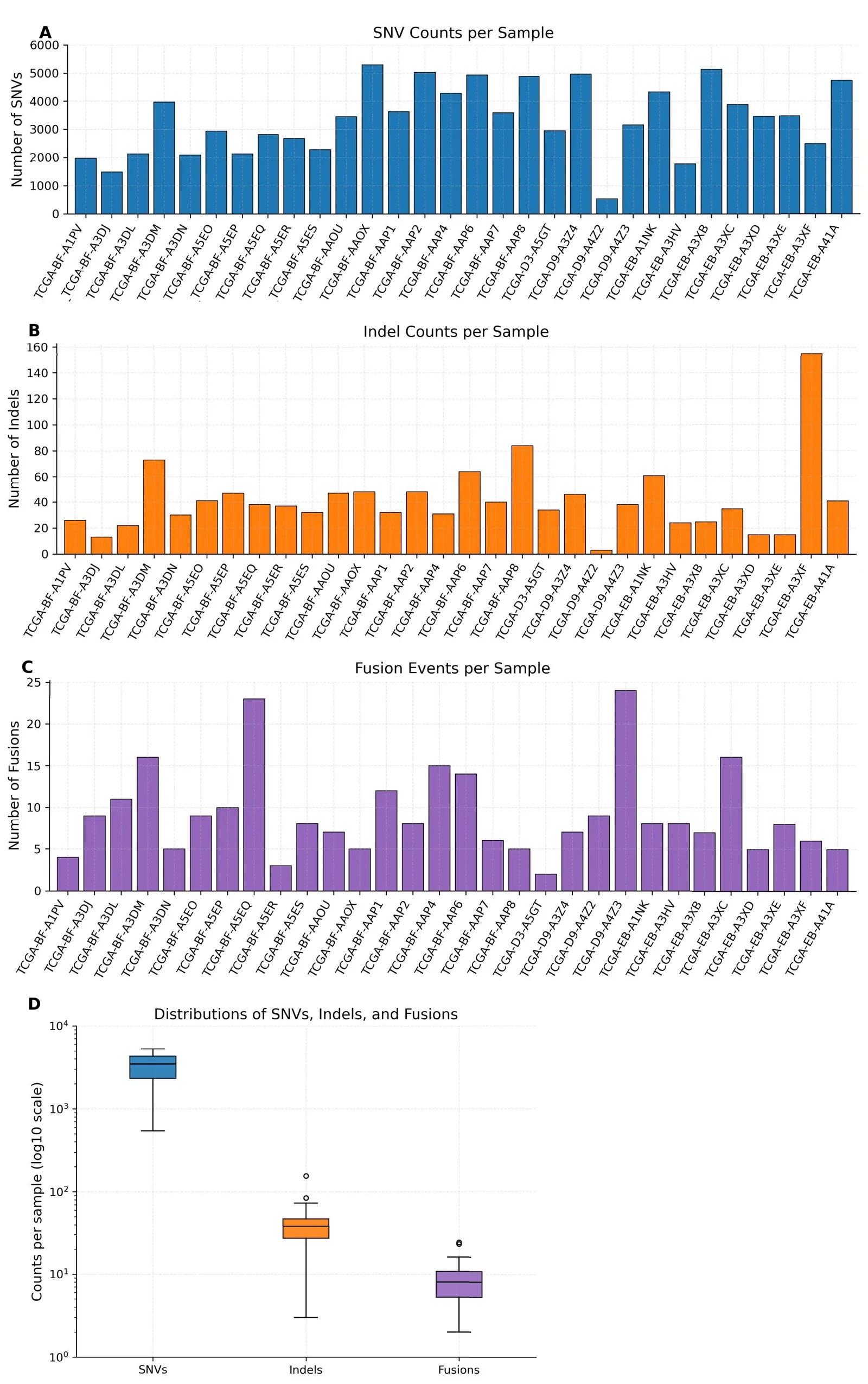
Mutational landscape analysis across TCGA samples processed by the EGG pipeline. (A) SNV counts per sample, showing variable mutational burden with the sample TCGA-BF-AAOX being highly mutated (∼5,300 SNVs). (B) Indel distribution, with most samples displaying fewer than 100 events and the sample TCGA-EB-A3XF showing the highest 155 indels. (C) Gene fusion events, range between 2 and 24 per sample. (D) Proportion of SNVs, Indels, and Fusion events per sample displayed as boxplots.

Specifically, we identified three classes of mutations potentially giving rise to cancer neoantigens, including SNVs (including splicing site modifiers), indels, and gene fusions. Our analysis revealed substantial inter-sample variability in mutational burden across all these three genomic alteration categories. As widely reported, including TCGA SKCM and subsequent WGS studies, SNVs are the predominant mutation class in melanoma reflecting UV-driven mutagenesis [73, 74]. This is consistent with our results where we found 1,000–5,000 somatic SNVs, though one sample (TCGA-BF-AAOX) exhibited the highest burden at ∼5,300 variants (Fig. 2A). While indel frequencies were generally lower, ranging from <15 to ∼100 events per sample, with TCGA-EB-A3XF displaying the highest burden at 155 indels (Fig. 2B). Gene fusion events, detected through STAR-Fusion analysis of tumor RNA-seq data, showed the lowest absolute frequencies with 2–24 fusion events identified per sample (Fig. 2C). These results confirmed that while SNVs dominate the mutational landscape (around 90% of all variants), also indels (around 5–10%) and gene fusions (usually less than 2–3% per sample) contribute meaningfully to the total neoantigen repertoire (Fig. 2D). EGG integrates several tools to carry out a comprehensive mutational profiling, allowing the users to process multiple classes of genomic alterations relevant to downstream neoantigen discovery[LM3.1].The tools embedded in the EGG workflow are reported in Additional File 1 Supplementary Material.

### Neoantigen burden distribution across mutation types

Neoantigen discovery modules of EGG, implemented via the pVACtools [22] suite, identified candidate epitopes derived from somatic variants (pVACseq), gene fusions (pVACfuse with STAR-Fusion input), and aberrant splicing events (pVACsplice with RegTools input) across the 30 samples selected from the TCGA melanoma cohort. Within the considered sample cohort, we identified up to 224 epitopes in a single sample across the three mutational classes (Fig. 3A–C). The binding affinity of epitopes to the MHC class I and class II complexes were computed. MHC class II slightly outnumbered class I predictions, consistently with the broader binding motif diversity of class II molecules. These results highlight the heterogeneity of neoantigen repertoires across patients and mutation types. To determine whether this heterogeneity simply reflected differences in input event burden, we correlated candidate neoantigen counts with the corresponding numbers of somatic variants and fusion events across the 30 TCGA-SKCM samples (Additional file 3, Supplementary Fig. S1A–C). For somatic variant-derived candidates, the total neoantigen count showed only a weak and non-significant positive correlation with SNV count (Spearman ρ = 0.26, *P*-value = 0.159) and with combined SNV plus indel count (ρ = 0.29, *P*-value = 0.124). Similar weak, non-significant associations were observed when MHC classes were analyzed separately, with SNV plus indel burden correlating weakly with both MHC class I candidates (ρ = 0.27, *P*-value = 0.143) and MHC class II candidates (ρ = 0.31, *P*-value = 0.100). Thus, the variability in mutation-derived neoantigen yield was not explained by SNV burden alone. In contrast, fusion-derived neoantigen counts were strongly associated with the number of detected fusion events. Total fusion-derived candidate counts correlated positively with fusion event burden (ρ = 0.72, *P*-value = 7.29 × 10⁻⁶), and this relationship was observed for both MHC class I (ρ = 0.65, *P*-value = 1.05 × 10⁻⁴) and MHC class II candidates (ρ = 0.73, *P*-value = 5.37 × 10⁻⁶). Together, these analyses show that EGG’s neoantigen output depends on both the number and type of underlying genomic alterations, but is not a simple linear consequence of SNV burden. Candidate peptide yield is also influenced by alteration consequence, altered protein sequence length and context, patient-specific HLA genotype and MHC class.

**Figure 3:**
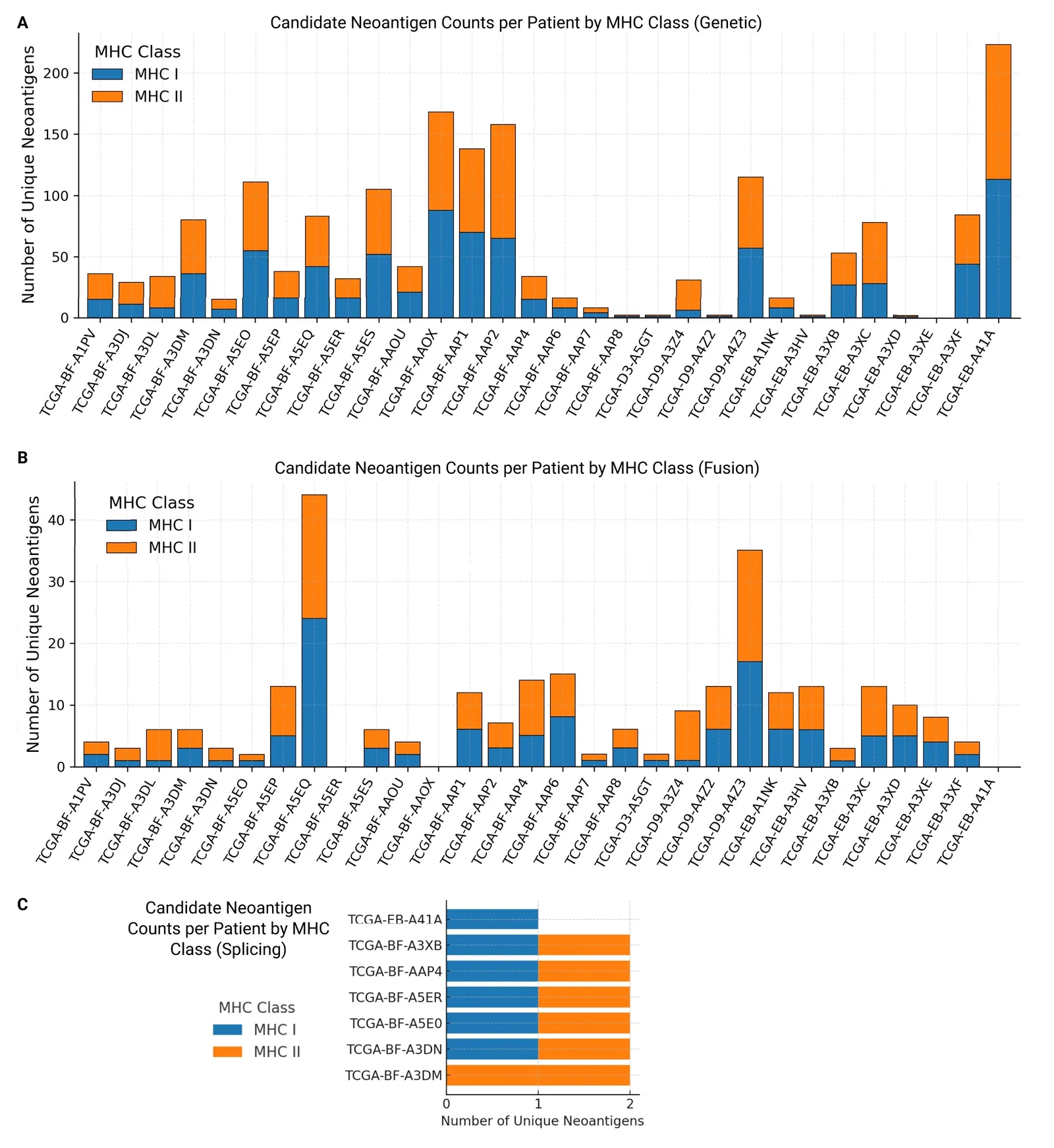
Predicted neoantigen landscape across TCGA melanoma samples processed by EGG. (A) Candidate neoantigens derived from somatic variants using pVACseq, stratified by MHC class (I and II). Samples yielded up to 224 epitopes, with somatic mutations contributing the largest overall pool. (B) Candidate neoantigens derived from gene fusions using pVACfuse with STAR-Fusion input, showing smaller but variable contributions across samples. (C) Candidate neoantigens derived from aberrant splicing events.

Since EGG jointly considers peptides predicted to bind HLA class I and HLA class II alleles, it captures candidate neoantigens that could contribute to cytotoxic CD8⁺ T-cell responses (class I) as well as helper CD4⁺ T-cell responses (class II), two complementary arms of anti-tumor immunity thought to be important for durable responses. Moreover, by adding sample-specific co-expression network analysis as a second-tier prioritization, EGG reduces large candidate pools into a smaller set of biologically relevant and therapeutically promising targets, bridging the gap between computational prediction and the practical need to select a limited number of epitopes for inclusion in a vaccine formulation.

### Integrating gene essentiality signals into epitope candidate ranking

Despite current methods are capable of identifying a remarkable amount of cancer neoantigen arising from genomic events, such as somatic mutations, fusions, and alternative splicing, translating this abundance into a clinically actionable candidates remains challenging. Therapeutic cancer vaccines typically deliver only a limited epitope set per patient, most peptide or mRNA platforms administer usually between 10 and 20 epitope sequences, whereas recent DNA plasmid vaccines have encoded larger payloads (up to 40 epitopes) in combination with PD-1 blockade. These design choices reflect manufacturing constraints, immunogenicity considerations, and trial feasibility [75–77]. Although sequencing-based pipelines can yield dozens to hundreds of candidate epitopes per tumor after standard HLA complex binding filters, current vaccine platforms can only accommodate a small subset of these in a single formulation. As a result, personalized vaccine design is not only an epitope level selection problem but also a gene level one: when only 10–40 epitopes can be used, choosing which genes they represent becomes necessary [2, 80]. Prioritizing epitopes from genes that are embedded in central, functionally indispensable positions within tumor networks offers a principled way to bias these limited slots toward targets that may be harder for the tumor to downregulate or eliminate under immune pressure [4, 81, 82].

In this study, we hypothesize that an additional layer of biological contextualization is required to rationally select effective epitopes that are, in turn, clinically relevant for the specific patient. To address this, EGG integrates a network based filtering strategy that captures patient specific gene expression patterns and exploits several sources of prior knowledge to refine the search for cancer epitopes. Specifically, EGG builds co-expression networks that are representative of the patient-specific gene expression combinations. Such a network is modeled and refined by using experimentally validated PPIs and membrane protein annotations in order to leverage the biological relevance of the co-expression relationships. We call this integrated network, pFIN. pFINs serve as a context-aware framework for refining neoantigen candidates toward those most likely to exert meaningful therapeutic impact (Fig. 4). pFINs isn’t simply a gene co-expression network filtered by PPI; we also enhanced it with the most confident connection related to membrane localized proteins. This membrane focused expansion is motivated by recent evidence that neoantigens derived from membrane localized proteins are enriched among immunogenic peptides and correlate with improved response to immune checkpoint blockade [14, 15]. To clarify the scale of the pruned patient-specific functional interaction networks used for prioritization, we also quantified the number of retained genes, edges, LCC size, and isolated genes across all 30 melanoma samples; these network-size summaries are provided in Additional file3, Supplementary Table S2.

**Figure 4:**
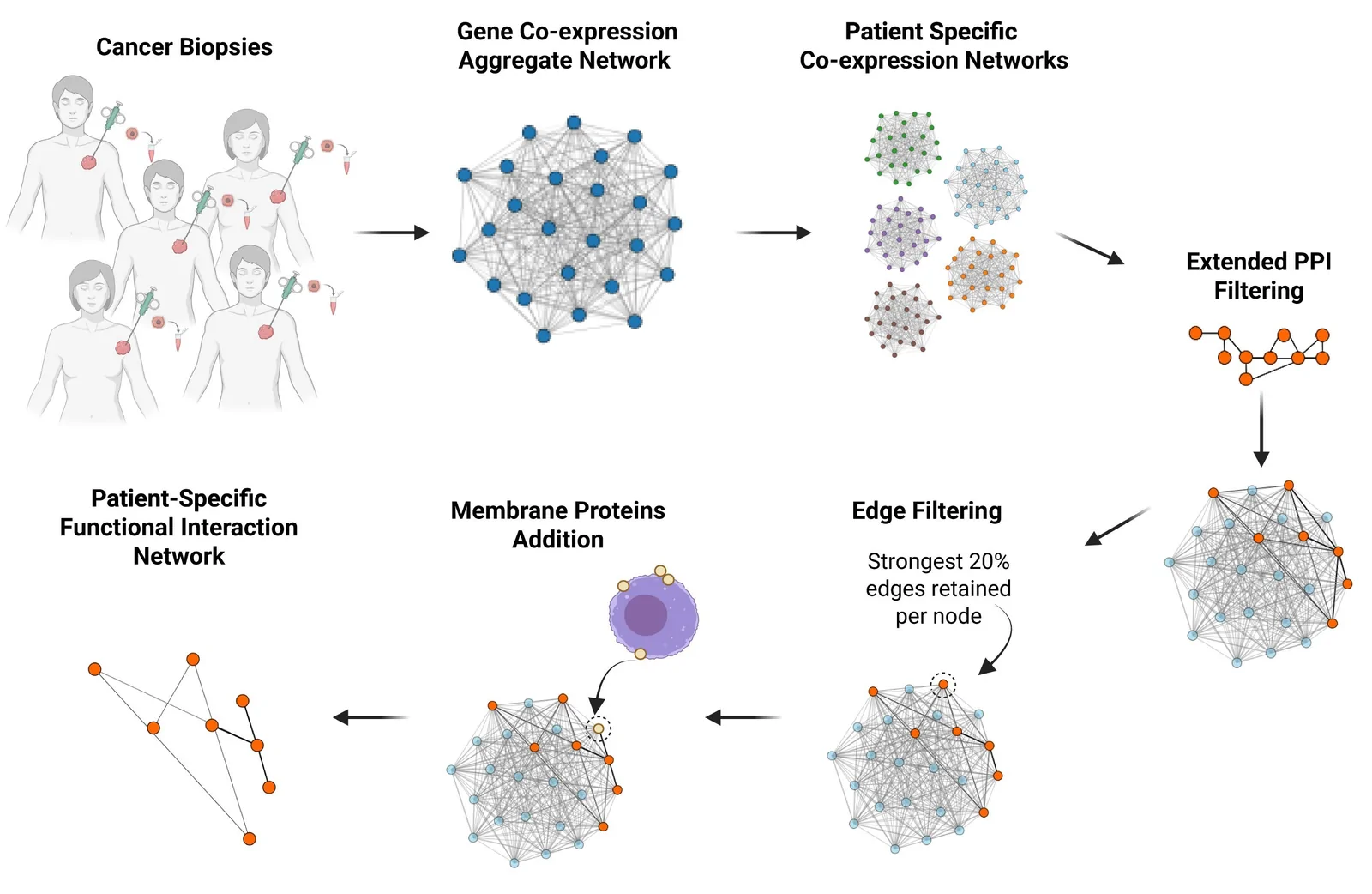
Network-based integration framework for epitope prioritization. RNA-seq data from patient specific cancer biopsies were processed within the EGG pipeline to generate raw gene expression counts. Pearson correlation across the normalized matrix was used to construct an aggregate gene co-expression network, from which patient-specific networks were derived. Each patient-specific co-expression network was subsequently filtered and refined for biological relevance. Networks were first constrained to interactions present in an extended PPI reference (HumanNet-XN). Edges were then filtered to retain the strongest connections per node. To enhance immunological relevance, edges involving membrane associated genes (annotated by GO terms) were added. The final output consisted of weighted, patient-specific functional interaction networks enriched for biologically supported and immunologically prioritized interactions. The steps illustrated in panels A and B are implemented by the sample_networks_generation; further usage details are provided are available as Additional file 1 (Supplementary Material).

EGG exploits the interactional properties of the pFINs predicting the functional relevance of each epitope’s gene of origin using topological metrics computed on the sample specific network degree, strength, betweenness centrality, and measures of network perturbation such as WCI and LCI. In this manner, it captures local connectivity, control of information flow, redundancy, and resilience. These gene level features are integrated with orthogonal evidence: CRISPR dependency scores from DepMap [24] as an experimental proxy for essentiality, and epitope level sequence metrics from pVACtools (e.g. predicted HLA class I/II binding), which assess the intrinsic immunogenic potential of the peptide independently of its gene of origin. Since these inputs derive from different experiments and sources, they are, per se, non-integrable. EGG converts each data source to ranks and aggregates them via a Borda procedure to produce a consensus ranked epitope list per sample (Fig. 5). To aid in the biological interpretation, prioritized genes are annotated with GO terms, MSigDB Hallmarks of Cancer processes [26], and IntOGen driver labels [27]. We incorporated these network topological features including degree, strength, betweenness centrality, and perturbation-based scores such as LCI since it has been shown that the placement of a gene within interaction networks is informative about its essentiality. The removal of highly connected and central proteins disproportionately fragments PPI networks (“centrality-lethality” rule) [83–85]. Percolation style measures that quantify the impact of node removal on the size and weight of the LCC, similar in spirit to our LCI, further highlight nodes whose loss compromises network integrity and the function of core pathways [12]. By prioritizing epitopes derived from genes with high LCI and high centrality, EGG explicitly favors targets whose deletion would incur a substantial systems-level fitness cost, and are therefore less likely to be suppressed through immune editing. This design directly addresses a limitation of current neoantigen vaccines: tumors frequently evade immune pressure by losing, downregulating, or epigenetically silencing immunogenic but dispensable neoantigens. Longitudinal studies in lung cancer under immune checkpoint blockade have documented selective loss of neoantigens and reshaping of the neoantigen landscape during therapy [16]. More broadly, neoantigen depletion, via copy-number loss or transcriptional repression is considered a major immune escape route that could reduce the efficacy of personalized vaccines [16]. Recent work further argues that vaccines targeting passenger neoepitopes will drive immune editing of those mutations, whereas antigens derived from oncogenic drivers or other functionally indispensable genes are more durable because their loss carries a higher fitness penalty [86].

**Figure 5:**
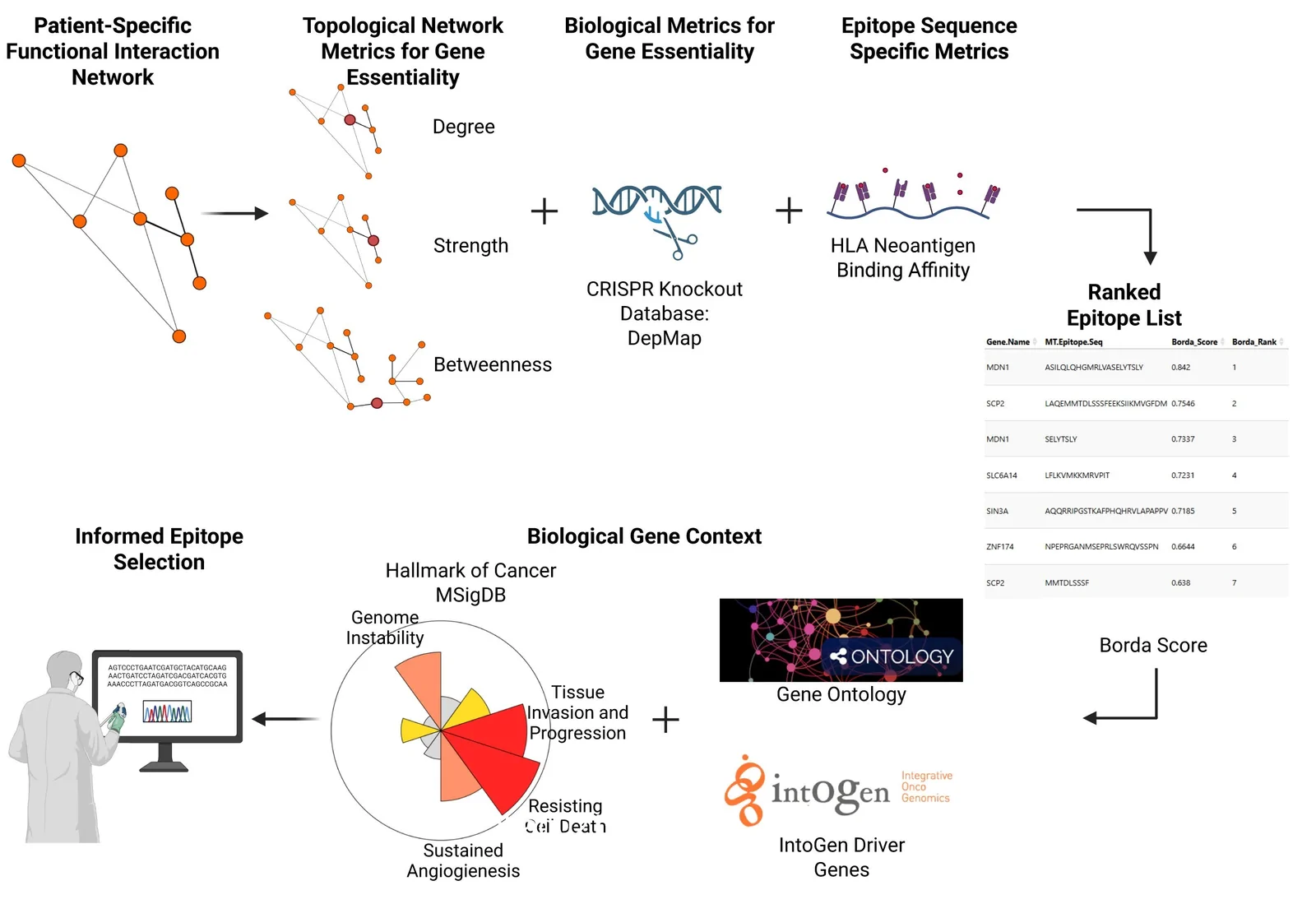
Epitope prioritization scheme. pFINs were used as the basis for epitope prioritization. Topological network metrics obtained from degree, edges strength, and betweenness (including LCI, WCI) were computed to establish a measure of gene essentiality specific to individual patients. These network-derived features were combined with external biological evidence, including CRISPR-based gene essentiality scores (DepMap), epitope specific sequence metrics such as predicted HLA binding affinity. The integrated information was aggregated into a ranked epitope list, ensuring robust consensus prioritization. To provide additional biological context, candidate genes were annotated with cancer hallmarks, GO terms, and IntOGen driver gene information. The resulting framework enables informed selection of epitopes by linking immunogenic potential to system-level relevance and cancer biology. The steps illustrated in this figure are implemented by the epitopes_prioritization module with details of usage are provided in Additional file 1 (Supplementary Material).

In addition, Al Bakir et al. showed that even a clonal EGFR driver neoantigen can be lost under combined TKI and neoantigen vaccine pressure, illustrating that oncogenic drivers themselves may be affected by immune escape [11]. This indicates that simply labeling genes as drivers or non-drivers is insufficient and supports incorporating different measures of functional indispensability to select the pool of candidate cancer vaccine epitopes. To complement topology-derived measures of indispensability with experimental evidence, EGG includes pan-cancer CRISPR dependency data from DepMap. Integrative analyses have shown that, given that network based features and genome scale CRISPR knockout screens capture non-identical sets of essential genes, their combination can widen the search space for neoantigen-deriving genes [72, 83, 87]. In this framework, the experimental dependency layer provides a broad cancer-cell fitness prior, while patient specificity is retained through the pFIN-derived topology computed from each sample. This multi-criteria scheme allows a rational prioritization of epitopes from genes that are both central in patient-specific pFINs and strongly essential across cancer cell lines, thereby increasing the chance that selected vaccine targets will remain expressed as the tumor evolves.

We further assessed the robustness and contribution of the pFIN network layer through several sensitivity analyses. Specifically, we evaluated pFIN stability using leave-one-background-sample-out jackknife analyses, top-edge consistency testing, and two GDC TCGA-SKCM benchmarks using larger *n* = 103 and *n* = 469 background cohorts. We also performed a membrane-edge expansion ablation, which showed that the membrane-focused augmentation used in this case study had a modest overall effect: it promoted selected membrane-associated candidates but did not globally dominate the final prioritization ranks. Users who wish to apply stronger membrane-focused prioritization can increase the membrane-edge expansion parameter in their own analyses. These analyses are provided in Additional file 3, Supplementary Tables S3–S7.

Our strategy explicitly mitigates immune escape via deletion or silencing of dispensable neoantigen source genes. However, this focus also defines its main limitation: EGG does not model escape mechanisms that operate through impaired antigen presentation, nor does it capture several immune-side determinants of neoantigen immunogenicity beyond peptide-HLA binding, such as antigen processing efficiency, T-cell receptor repertoire constraints, and immunodominance. EGG should therefore be interpreted as a prioritization framework centered on the tumor-side evolutionary durability of neoantigen source genes, rather than as a complete predictor of anti-tumor immune recognition. Consistent with this limitation, genetic and epigenetic alterations in HLA class I/II, β2-microglobulin, antigen processing machinery, and IFNγ signaling are frequent in clinical tumors and represent dominant routes of immune evasion under T cell pressure [88–90]. In such settings, neoantigen source gene essentiality becomes largely irrelevant, as tumor cells can evade recognition despite maintaining expression of the targeted gene.

### Determinants of epitope prioritization

By applying our multi-step prioritization approach described above across our TCGA melanoma samples, we identified correlation patterns among features utilized in the epitope prioritization. Such pairwise relationships are summarized in Fig. 6A. As expected, network centrality-based metrics are strongly intercorrelated and align closely with the Borda score, indicating that candidates from highly connected or influential genes tend to occupy top positions in the rank. DepMap survivability and predicted binding affinity showed a mild negative correlation with the Borda scores (negative values imply greater essentiality), and only limited correlation with network centralities. While DepMap highlights experimentally observed dependencies and provides biologically informed metrics, network centrality measures capture data-driven patterns of connectivity based on the unique molecular landscape of each patient. Exploiting both levels of information yields a more robust prioritization compared to single layer prioritization methods. We hypothesize that the prioritization of epitopes according to essentiality and network centrality will constrain immune escape, as the elimination of such genes is expected to impair tumor fitness. EGG’s ranking aligns most strongly with network centrality measures, while also retaining contributions from sequence-dependent features like HLA binding affinity and gene essentiality from CRISPR knockout studies. HLA expression was not included as a ranking criterion in the analysis presented here, explaining its limited correlation with the Borda score. This reflects EGG’s emphasis on complementing sequence-based epitope prioritization with gene-level functional information, including database-derived essentiality and patient-specific network topology. Nevertheless, HLA expression is retained in the final output tables and can be considered during downstream interpretation or included by the user as an additional ranking criterion in the borda score, together with other optional pVACtools algorithm outputs. We also evaluated the contribution of membrane-associated genes to the final prioritized epitope lists, given that membrane-associated edge expansion was included as an optional network refinement step. Across the 30 patients, membrane-associated genes accounted for a median of 22.3% of all candidate epitopes. Among top-ranked candidates, the median number of membrane-associated epitopes was 2 in the top 10, 4 in the top 20, and 10 in the top 50. Thus, membrane-derived epitopes were recovered among prioritized candidates, but they did not dominate the top-ranked sets. This is consistent with the intentionally light membrane-edge expansion used in the default analysis; stronger prioritization effects would be expected only if users deliberately increase the membrane-edge expansion parameter.

**Figure 6:**
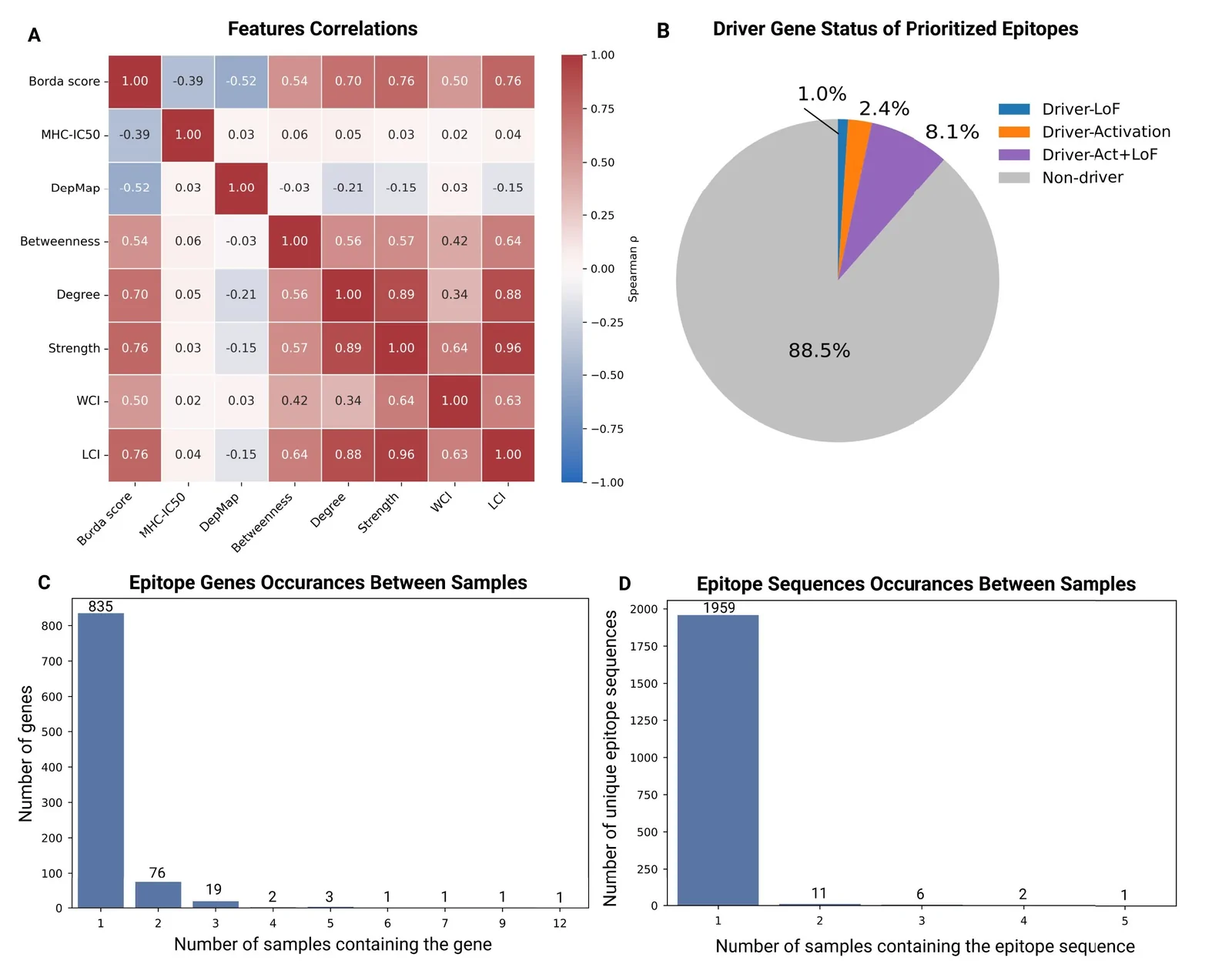
Exploratory analysis of epitope features. (A) Spearman correlation between features used in the Borda ranking procedure, highlighting expected dependencies among network-based measures and binding affinity scores. (B) Fraction of epitopes derived from driver vs. non-driver genes within the top-10 prioritized epitopes per sample, showing contributions from both loss-of-function (inactivating alterations disabling tumor suppressors) and activating (gain-of-function mutations activating oncogenes) drivers. (C) Distribution of genes shared between samples, showing that most candidate genes are unique to individual samples. A value of 1 indicates that the gene was observed in one sample only. (D) Distribution of epitope sequences shared between samples, with the majority being patient specific. A value of 1 indicates that the epitope sequence was observed in one sample only. Most genes and epitope sequences were patient-specific.

Driver-associated epitopes showed a modest but significant overrepresentation among the top prioritized candidates compared with the remaining candidate pool. As shown in Fig. 6B, driver-derived epitopes accounted for 34/296 top-10 candidates across samples (11.5%), compared with 133/1992 non-top-10 candidates (6.7%; one-sided Fisher’s exact test, odds ratio = 1.81, *P*-value = 0.0034). These driver-derived candidates included loss-of-function, activating, and mixed activating/loss-of-function driver annotations. Although most top-ranked epitopes were still derived from non-driver genes, the relative overrepresentation of driver-derived candidates is consistent with the inclusion of functional and network-context features in the prioritization framework. One possible explanation is that some cancer driver genes occupy more central or functionally important positions in the network, although this interpretation remains exploratory.To further evaluate potential determinants of prioritization, we tested whether peptide length or tumor DNA VAF influenced final epitope rank. Neither feature showed a meaningful association with prioritization outcome, supporting that EGG ranking was not primarily driven by peptide-length bias or VAF differences in this dataset (Additional file 3, Supplementary Figs 2 and 3).

In order to ascertain whether overrepresented epitopes across the population exist, we then assessed the occurrence of epitope candidates across patients. We observed that exact peptides are unique to individual patients (Fig. 6D), while the genes that give rise to epitopes show a small recurrence across cases (Fig. 6C). Altogether, these descriptive analyses illustrate the intended behavior of EGG in this case study: the pipeline defines the candidate space onto epitopes anchored in tumor-relevant biology, enriches for driver-gene targets, and produces shortlists that remain largely patient-specific at the peptide level while still revealing a small cohort of recurrent genes and epitope sequences that could inform shared targets or follow-up functional studies. Building on this, our data pinpoints a small set of recurrent targets. Among fusion derived events, KANSL1 is the most frequently shared gene (12 samples; Fig. 6C), and DIYKQIRANKVSVWRQ the most recurrent exact epitope (5 patients; Fig. 6D). These KANSL1-associated events were primarily linked to the KANSL1–ARL17/KANSARL fusion locus, which has been described previously in cancer transcriptomic studies [91]. A comparable pattern emerges for genomic mutations: exact mutation-derived peptides remain patient-specific, but the gene NRAS shows the highest recurrence (7 samples; Fig. 6C), with the epitope LDILDTAGREEYSAMRDQYMRTGEGFLCV shared across 3 patients (Fig. 6D). Since NRAS is a known recurrent melanoma driver, we compared this observation with the reported TCGA-SKCM NRAS mutation frequency of 28%. The observed frequency of NRAS-derived epitope candidates in the selected cohort was not higher than expected relative to this background rate (one-sided binomial test, *P*-value = 0.776), indicating that NRAS recurrence is consistent with the known melanoma mutational landscape rather than evidence of unexpected overrepresentation [74]. In both fusion and genomic contexts, the presence of recurrent epitopes highlights the potential for “off-the-shelf” vaccination strategies, an approach already explored for shared/public neoantigens such as KRAS hotspots and frameshift peptides in microsatellite instability (MSI) tumors. Early trials of shared neoantigen vaccines (including KRAS focused mRNA platforms and multi epitope regimens) demonstrate feasibility and immunogenicity, underscoring the translational value of the recurrent sequences identified by epitope generation pipelines [92].

Fundamentally, the main outputs of EGG, are interactive prioritization tables for each sample that integrate epitope and gene level features into a unified view. These tables report the HLA allele predicted to present each epitope together with corresponding transcript abundance, peptide sequence, mutation source, and class I/II designation. For each candidate, network-derived features (degree, strength, betweenness, LCI, and WCI) and external evidence (DepMap survivability, IntOGen driver status, cancer hallmark annotation, and GO terms) are displayed alongside the consensus Borda score and rank. This design provides investigators with a transparent overview of how multiple layers of information contribute to prioritization, linking peptide level immunogenicity with the biological relevance of the source gene. Tables for each patient that include prioritized epitopes and their topological and biological features obtained in our analysis can be found in Additional file 2 (Supplementary Material). To provide orthogonal bioinformatic support for the prioritization strategy, we compared the final EGG Borda ranking with an IC50 MHC binding affinity only ranking using external experimentally supported epitope evidence from the Immune Epitope Database [93], the Cancer Epitope Database and Analysis Resource [94], and TSNAdb v2.0 [95]. Because exact full-length peptide matches were rare, we used a relaxed min-8 containment match to identify candidates containing experimentally supported presented or immunogenic peptide cores; this analysis showed that Borda ranking improved recovery of externally supported candidates relative to IC50-only ranking at the most selective top-ranked cutoffs (Additional file 3, Supplementary Figs S6–S8 and Supplementary Tables S8–S9). Although EGG is designed to prioritize epitopes derived from genes with greater functional and network relevance, the proposed evolutionary durability of such targets remains a hypothesis in the present study. The external epitope-database analysis supports the potential presentation and immunogenicity of selected candidates, but does not directly test whether their source genes are less likely to be lost or silenced under immune pressure.

### Comparison of currently available neoantigen prediction pipelines

EGG adds a co-expression-network based prioritization step that ranks neoantigens by the centrality and indispensability of their source genes, with the goal of reducing immune evasion risk. The pipeline is implemented in Snakemake and automates resource retrieval and environment setup for local execution. Compared with prominent publicly available pipelines surveyed in Table 1, EGG’s network-aware prioritization constitutes a distinctive feature while keeping installation and execution simple. To contextualize EGG within the current landscape, we note the following pipelines: pVACtools stands out as a modular ecosystem (pVACseq/bind/fuse/splice) with extensive algorithm support and integrated visualization (pVACview) and vaccine design utilities (pVACvector); pVACtools also includes comprehensive workflows, such as immuno.wdl on the Terra platform, to automate end-to-end analyses, including upstream preprocessing. TSNAD v2.0 is an end-to-end pipeline for tumor-normal WES/WGS (with optional RNA-seq) that incorporates expression and gene-fusion filters and uses the deep-learning predictor DeepHLApan to score both HLA binding and immunogenicity [96, 97]. ScanNeo2 provides an end-to-end workflow from raw DNA/RNA data and uniquely broadens variant coverage to include exitron splicing and gene fusions alongside SNVs/indels, enabling wider candidate recovery [9]. INTEGRATE-neo is purpose-built for fusion-derived neoantigens, offering an automated pipeline from fusion calling through HLA binding specifically for gene-fusion events [5]. nextNEOpi [98] is a Nextflow [99] based, fully automated pipeline that starts from raw WES/WGS in combination with RNA-seq and additionally quantifies patient and neoepitope level features linked to immunogenicity and response to checkpoint blockade. NeoHunter emphasizes flexible input handling and systematically detects and prioritizes neoantigens not only from SNVs/indels but also from gene fusions and aberrant splicing within a single framework [10]. NeoDisc is an end-to-end clinical proteogenomic pipeline that integrates immunopeptidomics with genomics and transcriptomics to prioritize both canonical and noncanonical tumor specific antigens [100]. SNAF focuses on splicing-derived antigens, predicting MHC-bound T-cell epitopes and altered-surface B-cell targets and using machine-learning prioritization to enrich for immunogenic events [8]. Seq2Neo provides a one-stop workflow from raw data and incorporates a convolutional neural network immunogenicity model to rank candidates derived from SNVs, indels, and gene fusions [101]. TransHLA is a recent deep-learning model for HLA-presented epitope detection rather than a full sequencing-to-neoantigen workflow; it uses Transformer and Residue CNN architectures with ESM2-derived sequence and structure embeddings to predict HLA class I and class II epitopes, and notably performs peptide-level screening without requiring HLA alleles as input [102].

**Table 1:** Feature matrix for neoantigen prediction pipelines and epitope-prediction tools.

Feature	EGG	pVACtools	ScanNeo2	INTEGRATE-neo	nextNEOpi	TSNAD v2.0	NeoHunter	NeoDisc	SNAF	Seq2Neo	TransHLA
End-to-end sequencing pipeline	✓	✓	✓	✓	✓	✓	✓	✓	✓	✓	—
Modular tool suite	✓	✓	—	—	—	—	—	—	—	—	—
SNV/indel neoantigens	✓	✓	✓	—	✓	✓	✓	✓	—	✓	—
Fusion neoantigens	✓	✓	✓	✓	✓	✓	✓	✓	—	✓	—
Alternative splicing neoantigens	✓	✓	✓	—	—	—	✓	✓	✓	—	—
Noncanonical/proteogenomic antigens	—	—	—	—	—	—	—	✓	—	—	—
MHC class I	✓	✓	✓	✓	✓	✓	✓	✓	✓	✓	✓
MHC class II	✓	✓	✓	—	✓	—	—	✓	✓	✓	✓
HLA typing included/supported	✓	✓	✓	—	✓	✓	✓	✓	✓	✓	Not required
Agretopicity/mutant-vs-WT comparison	✓	✓	—	—	—	—	✓	—	—	—	—
Immunogenicity/presentation prediction	✓	✓	✓	—	✓	✓	✓	✓	✓	✓	✓
DepMap-based gene essentiality	✓	—	—	—	—	—	—	—	—	—	—
Patient-specific source-gene network context	✓	—	—	—	—	—	—	—	—	—	—
PPI-informed network context	✓	—	—	—	—	—	—	—	—	—	—
Network topology metrics	✓	—	—	—	—	—	—	—	—	—	—
Membrane/surface-gene enhancement	✓	—	—	—	—	—	—	—	✓	—	—
Gene labels (eg. IntOGen and GO)	✓	✓	—	—	✓	—	—	✓	—	—	—
Multivariable prioritization	✓	✓	✓	—	✓	✓	✓	✓	✓	✓	—
Configurable weighted consensus ranking	✓	—	—	—	—	—	—	—	—	—	—
Ranked final neoepitope table	✓	✓	✓	✓	✓	✓	✓	✓	✓	✓	—
Prediction score output	✓	✓	✓	✓	✓	✓	✓	✓	✓	✓	✓

This table compares the EGG with selected public neoantigen prediction workflows and standalone epitope-prediction tools. For each tool, we summarize supported somatic alteration classes, input requirements, MHC class coverage, HLA typing or HLA input requirements, immunogenicity or presentation prediction, and prioritization features. Because some entries, such as TransHLA, are prediction models rather than full end-to-end sequencing pipelines, not all workflow-level features are applicable. Checkmarks indicate that the feature is included or supported by the tool or tool suite; dashes indicate that the feature is not part of the standard reported functionality or is not applicable. EGG is distinguished by combining neoantigen discovery with patient-specific source-gene co-expression context, PPI-informed network metrics, DepMap-based gene essentiality, and configurable weighted consensus ranking.

Crucially, the prioritization module of EGG is flexible and fully customizable by the user. Optional features can be enabled or disabled and thresholds adjusted, e.g. using different algorithm for peptide MHC binding, immunogenicity scores, gene essentiality, and multiple network-based centrality metrics. The relative weights of these features used during prioritization are also modifiable by the users, allowing researchers to emphasize specific criteria for a customizable ranking process.

## Conclusion

EGG can bridge the gap between abundant raw neoantigen predictions and the practical need for biologically meaningful ordered results. By embedding candidate epitopes within patient-specific functional interaction networks and incorporating orthogonal evidence of essentiality and driver status, EGG prioritizes epitopes more likely to be therapeutically relevant by prioritizing more essential genes less likely to be subject to immune evasion while also offering the user with alternative prioritization methods and important gene related biological information. In the case study presented in this work, we highlight that the majority of epitopes remain unique between patients at the sequence level, whereas gene level recurrence is more common. These results show that EGG can serve as a flexible framework for integrating diverse data sources, balancing immunological plausibility with tumor systems biology, and informing the rational design of personalized cancer vaccines.

## Additional files

Additional file 1 (.docx): Supplementary Methods/Supplementary Protocol.

Description: Step-by-step instructions for execution of individual pipeline modules.

Additional file 2 (.xlsx): Supplementary Tables.

Description: Patient-level tables of prioritized epitopes and associated topological and biological features.

Additional file 3 (.docx): Supplementary Analyses.

Description: Additional sensitivity, robustness, benchmarking, and validation analyses supporting the prioritization framework, including network stability, ranking-weight sensitivity, membrane-expansion ablation, peptide-length and VAF assessments, external evidence matching, and genomic-event burden correlations.

Supplementary Figures 1–8 (.pdf). Supplementary Analyses Figures.

Figures included in Additional file 3, also provided as individual PDF files.

## Abbreviations

EGG: Epitope Generation Gateway; GO: gene ontology; HLA: human leukocyte antigen; LCC: largest connected component; LCI: Largest Component Impact; MHC: Major Histocompatibility Complex; MSI: microsatellite instability; pFIN: Patient Specific Functional Interaction Network; PPI: protein–protein interaction; SKCM: skin cutaneous melanoma; SNV: single nucleotide variation; TCGA: The Cancer Genome Atlas; VAF: variant allele frequency; VST: variance stabilizing transformation; and WCI: Weighted Connectivity Impact.

## Availability of source code and requirements

Project name: epitope_generation_gateway.

Project home page: https://github.com/fhaive/epitope_generation_gateway.

License: GPL-3.0 license. Operating system(s): Linux/Unix-like systems.

Programming language: Snakemake, Python, R, Bash/shell, and YAML.

Other requirements: Conda or Mamba, Snakemake ≥6.0, Docker, and standard command-line bioinformatics dependencies installed through the workflow-managed Conda environments and Docker containers.

Recommended computational resources depend on dataset size, sequencing depth, mutation burden, and the number of samples.

For the 30-sample TCGA melanoma benchmark, the most memory-intensive EGG-specific step was membrane-edge enrichment, requiring approximately 75 GB peak RAM per sample, while LIONESS reconstruction required approximately 36 GB peak RAM for the 30-sample cohort.

RRID:SCR_028792

bio.tools: pitope_generation_gateway WorkflowHub: http://workflowhub.eu/workflows/2165?version=2.

## Supplementary Material

giag095_Supplemental_Files

giag095_Authors_Response_To_Reviewer_Comments_original_submission

giag095_Authors_Response_To_Reviewer_Comments_revision_1

giag095_GIGA-D-26-00020_original_submission

giag095_GIGA-D-26-00020_Revision_1

giag095_GIGA-D-26-00020_Revision_2

giag095_Reviewer_1_Report_original_submission Reviewer 1 -- 2/25/2026

giag095_Reviewer_1_Report_revision_1 Reviewer 1 -- 6/25/2026

giag095_Reviewer_2_Report_original_submission Reviewer 2 -- 3/3/2026

giag095_Reviewer_2_Report_revision_1 Reviewer 2 -- 6/15/2026

## Data Availability

Raw data: Matched tumor RNA-seq, tumor DNA (WES), and normal DNA samples were retrieved from The Cancer Genome Atlas (TCGA) for 30 patients diagnosed with cutaneous melanoma (TCGA-SKCM study code) [32]. The 30 corresponding UUIDs are listed in Additional file 2. Output data: Output data is provided in Additional file 2. Supplementary Methods: Pipeline Implementation is described in Additional file 1. Supplementary Analyses: Supplementary analyses are described in Additional file 3. Supplementary figures: Supplementary Figures 1–8 are provided as separate PDF files.
