# Supplementary material for: Epitope Generation Gateway: A biological context-dependent approach to cancer vaccine development
Source: Gigascience. 2026 Oct 1;15:giag095. doi: 10.1093/gigascience/giag095 (PMC13679409; doi:10.1093/gigascience/giag095)
Supplement: Supplementary file 1 [file giag095_authors_response_to_reviewer_comments_original_submission.pdf]

Author's Response To Reviewer Comments

Dear Editor and Reviewers,
We sincerely appreciate your consideration evaluating our manuscript and for the constructive comments that helped us improve it. Below, we provide a detailed point-by-point response to each reviewer comment. Revised PDF versions of the manuscript and supplementary information, with all modifications indicated using track changes, have been included with the resubmission. The main revisions to the manuscript are also summarized in our responses below and can be viewed in the attached "Manuscript with track changes" PDF file, while updates to the supplementary information are shown in the corresponding "Supplementary Information with track changes" PDF file.

We have also provided the response-to-reviewers document as supplementary material in both Word and PDF formats, named Answers_to_Reviewer_Comments.docx and Answers_to_Reviewer_Comments.pdf, respectively. The response text in these files is identical to that provided in this separate response document; the Word and PDF versions additionally include the relevant tables and figures embedded directly to facilitate review.

In addition to the reviewer-requested revisions, we carefully rechecked the analysis outputs and updated the manuscript to correct inconsistencies identified during this process. First, we corrected a file-handling issue in the mutational landscape analysis underlying Figure 2, where both compressed and uncompressed versions of the same VCF files had been included in the mutation-counting step. This affected the reported SNV and indel counts in Figure 2A–B. Second, we identified that the variant-effect annotation reference used in the previous run did not include four chromosomes. We therefore regenerated the VEP-annotated mutation inputs and downstream epitope predictions after including the missing chromosomes in the updated analysis. As a result, the final mutation and epitope counts are not a simple halving of the previous values: the duplicate-file counting correction reduced the inflated mutation counts, while inclusion of the previously missing chromosomes added back variants and epitope candidates that had not been annotated in the earlier run.
The affected mutation counts, epitope summaries, figures, supplementary tables, and related result descriptions have therefore been regenerated from the updated analysis. This includes the mutation landscape in Figure 2, the epitope-yield summaries in Figure 3, the final epitope supplementary tables, and downstream plots based on the final prioritized candidate tables, including Figure 6. In Figure 6B, we also refined the parsing of IntOGen driver-role annotations so that compact labels such as "Act", "LoF", and "Act;LoF" are represented more accurately as activating, loss-of-function, or mixed activating/loss-of-function driver annotations. These corrections updated the numerical values reported in the affected results, but did not alter the overall conclusions of the study.
We also improved the description of the membrane-enrichment step in the Methods section. In the original text, the membrane-edge expansion was described as adding 5% of the number of edges remaining after PPI filtering. We have corrected this explanation to reflect the implemented workflow: in the default analysis, the number of additional membrane-associated edges is calculated as 5% of the weighted HumanNet/PPI edge set before the per-gene top-20% PPI filtering step, and these top-ranked membrane-touched edges are then added to the PPI-filtered network. We also clarified that this step is configurable and can be disabled directly through the updated configuration file.

The scripts used to perform the additional analyses requested during peer review have been made available in the GitHub repository under epitope_generation_gateway/Reviewer_questions_scripts at revision_scripts · fhaive/epitope_generation_gateway in the revision_scripts branch. We have also included the analyses carried out in response to the reviewers' comments, together with the corresponding response text and supporting tables/figures, in Additional Files 1 and 3. These supplementary analyses and figures are referenced at the relevant points in the revised manuscript text. Where text was added to the main document of the manuscript, the exact added or modified wording is reported in this response document in italicized quotation marks.

Editors comments:

We thank the editors for these helpful suggestions. We have registered the EGG computational workflow in WorkflowHub and added the corresponding DOI to the revised manuscript. We have attempted to register EGG in bio.tools and SciCrunch.org to obtain the requested biotoolsID and RRID identifiers. However, at the time of revision, both registration processes were blocked by account-creation issues that prevented us from completing the submission. We have contacted the relevant support channels (and opened github Issue: Signup page hangs indefinitely when creating a new account · Issue #733 · bio-tools/biotoolsRegistry) and will complete these registrations and add the identifiers to the manuscript as soon as account creation becomes available.

Reviewer #1: This manuscript presents EGG, a computational framework designed to prioritize neoantigen candidates by integrating conventional epitope prediction with gene-level measures of functional indispensability derived from co-expression networks, protein-protein interaction data, and CRISPR dependency scores. The authors aim to address the problem of immune escape driven by loss of dispensable tumor antigens under immune pressure.

Overall, the manuscript describes a thoughtfully engineered and reproducible computational pipeline. The integration of mutation calling, HLA typing, epitope prediction, patient-specific network construction, and multi-criteria ranking using Borda aggregation is technically solid and clearly implemented. Great attention to reproducibility is shown through well documented repositories, containerization and workflow management.

Conceptually, the attempt to incorporate tumor systems biology into neoantigen prioritization represents a meaningful step beyond binding affinity-only approaches.
However, several conceptual and methodological issues should be addressed before the manuscript is suitable for publication.

****

A - Major comments and questions:

A1: I am wondering why the hardcoded membrane skew is necessary. I see that two papers are cited and one seems to report better survival for tumors with higher number of membrane-derived neoantigens. I can't help but wonder, however, if there is a potential bias here, especially in low-mutational burden tumors. Perhaps this membrane-targeting should be optional or extended to other localisations? So that the used can define what they want to prioritize?
Also, I have noticed that there is no mention of the membrane-derived peptides in the results. Has your method recovered any? This warrants investigation since the authors make a point of mentioning it four times and then this disappears later on in the manuscript

We thank the reviewer for this comment that helps us clarifying aspects related to the prioritization of membrane-derived peptides. We agree that a membrane-focused expansion step could introduce a user-defined prior toward membrane-associated genes if presented as mandatory or fixed. We would like to clarify that the membrane enrichment step does not directly add Borda points to membrane proteins; instead, it adds a configurable fraction of membrane-associated edges to the PPI-filtered pFIN before network metrics are calculated. Thus, it expands the network context available to membrane-associated candidates rather than imposing a hard filter or requiring membrane localization for prioritization.
In the original implementation, the contribution of this step could already be adjusted by changing the percentage of membrane-associated edges added to the filtered PPI network, and setting this contribution to zero effectively disables the step. However, we acknowledge that this was not sufficiently clear to users. Following the reviewer’s suggestion, we have updated the pipeline and documentation to make the membrane expansion explicitly optional and user-controlled. We have also clarified in the Methods that this step represents an optional prioritization prior that can be disabled or tuned depending on the biological question and cancer context.
We also agree that the presence and impact of membrane-derived prioritized candidates should be reported. We therefore quantified membrane-associated source genes among the final ranked epitopes using the same membrane gene set used for the network expansion. Across the 30 patients, membrane-associated genes accounted for a median of 22.3% of all candidate epitopes. Among top-ranked

candidates, the median number of membrane-associated epitopes was 2 in the top 10, 4 in the top 20, and 10 in the top 50. Thus, membrane-derived epitopes were recovered among prioritized candidates, but they did not dominate the top-ranked sets.

To further test whether the membrane expansion materially changed prioritization, we performed a membrane-expansion ablation. We recomputed the final weighted Borda ranking using the same feature weights and directions as in the original workflow, but replaced the PPI + membrane-expanded pFIN metrics with metrics calculated from the PPI-filtered networks before membrane-edge addition. All non-network features, including IC50, DepMap survivability, IntOGen annotations, and WCI, were kept unchanged. The recomputed original weighted Borda ranking closely reproduced the final ranking, confirming that the ablation reproduced the ranking procedure, with median Spearman ρ = 0.9998 and median Kendall τ = 0.993.

Removing the membrane expansion had only a modest effect on the global final ranking. The final PPI + membrane ranking and the PPI-only ablation ranking had a median Spearman ρ of 0.979, a minimum Spearman ρ of 0.885, and a median Kendall τ of 0.901. The top-ranked candidates were also largely preserved, with a median top-10 overlap of 9/10 epitopes and a median top-20 overlap of 18/20 epitopes. The number of membrane-derived epitopes in the top-ranked sets was essentially unchanged, with a median of 2 membrane-derived epitopes in the top 10 and 4 in the top 20 in both the original and PPI-only rankings.

At the same time, the membrane expansion had the intended local effect in selected cases. Across 2,288 candidate epitopes, 523 were derived from membrane-associated genes. Among these, 236 membrane-derived epitopes across 25 patients showed some upward rank shift when the membrane expansion was included. More substantially, 78 membrane-derived epitopes across 17 patients improved by at least 5 rank positions, and 26 epitopes across 9 patients improved by at least 10 positions. In 8 patients, the membrane expansion moved a membrane-derived epitope into the top 10, and in 12 patients it moved at least one membrane-derived epitope into the top 20. These results indicate that the membrane expansion acts as a limited, optional network augmentation rather than a dominant driver of the final ranking.

We have added this analysis to the Additional file 3 in the supplementary Materials and revised the Results section to clarify the contribution of membrane-associated genes to the final epitope rankings by adding the following paragraph:

"We also evaluated the contribution of membrane-associated genes to the final prioritized epitope lists, given that membrane-associated edge expansion was included as an optional network refinement step. Across the 30 patients, membrane-associated genes accounted for a median of 22.3% of all candidate epitopes. Among top-ranked candidates, the median number of membrane-associated epitopes was 2 in the top 10, 4 in the top 20, and 10 in the top 50. Thus, membrane-derived epitopes were recovered among prioritized candidates, but they did not dominate the top-ranked sets. This is consistent with the intentionally light membrane-edge expansion used in the default analysis; stronger prioritization effects would be expected only if users deliberately increase the membrane-edge expansion parameter."

We have also revised the Methods section to clarify how membrane-associated edges are added. Specifically, we now state that the default membrane expansion selects additional membrane-touched edges from the full sample-specific co-expression network, ranks them by absolute correlation weight, and adds a configurable fraction to the PPI-filtered network before network metrics are calculated.

" In the default configuration, candidate membrane-associated edges were defined as edges in which at least one endpoint gene was annotated as membrane associated. These candidate edges were ranked by absolute correlation weight, and the top-ranked edges were added to the PPI-filtered network. The number of membrane-associated edges added was controlled by a configurable proportion, set to 5% by default. In the analysis presented here, this proportion was calculated relative to the number of weighted HumanNet/PPI edges before the per-gene top-20% PPI filtering step."

In addition, we improved the configuration file controlling membrane-edge addition. The membrane-enrichment step now has a dedicated configuration block with an explicit enabled option and an add_prop parameter. This makes it clearer for users to disable the step directly, rather than indirectly setting the membrane-edge percentage to zero, while still allowing users to tune the fraction of membrane-associated edges added.

file filter_config.yaml:

"

```
# Configuration for sample-specific network filtering.
#
# The final filtered network is built in two explicit steps:
```

```
# 1. PPI filtering using HumanNet edges.
# 2. Optional enrichment with high-weight edges touching membrane genes.

ppi_filter:
# For each gene/node, keep this fraction of its strongest incident HumanNet/PPI edges.
# The strength is based on absolute sample-specific LIONESS edge weight.
# 0.20 keeps the top 20% of PPI edges per gene.
top_prop: 0.20

membrane_enrichment:
# If true, add additional high-weight raw-network edges that touch membrane genes.
# If false, the final filtered network will be the PPI-only filtered network.
enabled: true

# Fraction of additional membrane-touched edges to add.
# For example, add_prop: 0.05 adds 5% additional membrane-touched edges.
add_prop: 0.05

# Denominator used to calculate how many membrane-touched edges are added.
# Options:
# "unfiltered_ppi_edges":
# n_added = ceiling(add_prop * number_of_weighted_unfiltered_HumanNet_edges)
# "ppi_filtered_edges":
# n_added = ceiling(add_prop * number_of_edges_in_the_PPI_only_filtered_network)
denominator: "unfiltered_ppi_edges"

# Candidate membrane-enrichment edges must touch at least one membrane gene.
# This should remain true. Setting it to false is not currently supported because
# it would change the biological meaning from membrane enrichment to general edge enrichment.
require_membrane_endpoint: true

# Candidate membrane-touched edges are ranked by absolute LIONESS edge weight.
# Currently, only "absolute_weight" is supported.
edge_selection_metric: "absolute_weight"
‟
```


A2: Major issues with flow. In the section "Integrating gene essentiality signals into epitope candidate ranking " the text needs tightening. There is a lot of redundancy, some parts read more like a discussion. Borda as well as DepMap explanations are mentioned several (two, three) times and are redundant and are explained in different ways with no new information. This section would gain in clarity from a text tightening.

We have modified the section accordingly to make the text clear and less redundant, the modified text reads as following:
“To complement topology-derived measures of indispensability with experimental evidence, EGG includes pan-cancer CRISPR dependency data from DepMap. Integrative analyses have shown that, given that network based features and genome scale CRISPR knockout screens capture non identical sets of essential genes, their combination can widen the search space for neoantigen-deriving genes [62,73,77]. In this framework, the experimental dependency layer provides a broad cancer-cell fitness prior, while patient specificity is retained through the pFIN-derived topology computed from each sample. This multi-criteria scheme allows a rational prioritisation of epitopes from genes that are both central in patient-specific pFINs and strongly essential across cancer cell lines, thereby increasing the chance that selected vaccine targets will remain expressed as the tumor evolves.”

A3: In the section around Figure 2, there is a sentence: "This comprehensive mutational profiling demonstrates the ability of EGG to capture the full spectrum of genomic alterations that can generate immunologically relevant neoantigens, positioning it as a robust tool for personalized cancer

immunotherapy target identification. "
From what I understand, this section simply shows the mutational burden and indeed and fusion recovered by the tools. I don't think at this stage this results demonstrates anything about EGG, considering there is no peptide prioritization at this stage and it's simply applying the tools. If this is correct, authors should soften the conclusion of that sentence to reflect what the result truly shows.

We thank the reviewer for this comment and agree that the original sentence overstated the interpretation of this result. The original sentence was intended to explain the ability of the tool to decompose the per-sample molecular landscape, extrapolating the different classes of genomic alterations that could give origin to therapeutically valid neoantigens. In fact, EGG carries out such a characterization by integrating existing methods and tools, that have been previously utilised and benchmarked in other studies such as the pvactools suite and mutect2 and starfusion for mutation calling. In order to downstate our sentence and better clarify the actual contribution of EGG to the mutational profiling, we modified the text accordingly:

“EGG integrates several tools to carry out a comprehensive mutational profiling, allowing the users to process multiple classes of genomic alterations relevant to downstream neoantigen discovery.The tools embedded in the EGG workflow are reported in Additional File 1 Supplementary Material.”

A4: Could authors comment on the large variability in peptides detected in Figure 3A and 3B? Is there a correlation with the number of SNV per sample? This would add precious information about EGG's behavior and limits

We thank the reviewer for this important observation. We acknowledge that a remarkable variability in the number of identified neoantigens between genomic and fusion events exists and that it deserves a more thorough investigation. To assess whether the variability in candidate peptide counts in Figure 3A and 3B reflects the underlying genomic event burden, we correlated the neoantigen counts with the corresponding mutation and fusion event counts across the 30 TCGA-SKCM samples.
For mutation-derived candidates in Figure 3A, the total genomic neoantigen count showed only a weak, non-significant positive correlation with SNV count (Spearman $\rho = 0.26$, p-value = 0.159) and with the combined SNV + indel count ($\rho = 0.29$, p-value = 0.124). Although indel count alone showed a modest positive correlation with total somatic neoantigen count ($\rho = 0.38$, p-value = 0.039), indels represented only a small fraction of the total genomic variant burden in these samples; therefore, this association should be interpreted cautiously and does not indicate that overall mutation-derived neoantigen variability is primarily driven by indel burden. When MHC classes were considered separately, the associations between genomic neoantigen count and SNV + indel count remained weak and non-significant for both MHC class I candidates ($\rho = 0.27$, p-value = 0.143) and MHC class II candidates ($\rho = 0.31$, p-value = 0.100). These results indicate that the variability in Figure 3A is not explained by SNV burden alone.
For fusion-derived candidates in Figure 3B, we observed a strong positive correlation between the number of fusion events and the total number of fusion-derived neoantigens ($\rho = 0.72$, p-value = 7.29×10^{-6}). This relationship was present for both MHC class I fusion candidates ($\rho = 0.65$, p-value = 1.05×10^{-4}) and MHC class II fusion candidates ($\rho = 0.73$, p-value = 5.37×10^{-6}). In contrast, fusion-derived neoantigen counts were not correlated with SNV count ($\rho = -0.03$, p-value = 0.883) or combined SNV + indel count ($\rho = -0.04$, p-value = 0.853), indicating that the variability in Figure 3B is driven by fusion burden rather than by overall SNV/indel mutation burden.
Overall, these analyses show that EGG’s neoantigen yield reflects both the number and the type of underlying genomic events, but is not a simple linear consequence of SNV burden This is expected because different genomic alterations have unequal neoantigen-generating potential: some variants are synonymous or otherwise do not substantially alter the encoded protein, whereas others, such as frameshift indels, can generate longer altered peptide sequences with greater potential to produce candidate epitopes. Candidate peptide yield is also shaped by the length and sequence context of the altered protein product, patient-specific HLA genotype and MHC class. The full correlation analysis is now reported in Additional File 3, including supplementary figure 1 reported below. The main manuscript has been updated with the following summary:
“To determine whether this heterogeneity simply reflected differences in input event burden, we correlated candidate neoantigen counts with the corresponding numbers of somatic variants and fusion events across the 30 TCGA-SKCM samples (Supplementary Material 3, Supplementary Figure S3A–C). For somatic

variant-derived candidates, the total neoantigen count showed only a weak and non-significant positive correlation with SNV count (Spearman ρ = 0.26, p-value = 0.159) and with combined SNV plus indel count (ρ = 0.29, p-value = 0.124). Similar weak, non-significant associations were observed when MHC classes were analyzed separately, with SNV plus indel burden correlating weakly with both MHC class I candidates (ρ = 0.27, p-value = 0.143) and MHC class II candidates (ρ = 0.31, p-value = 0.100). Thus, the variability in mutation-derived neoantigen yield was not explained by SNV burden alone.
By contrast, fusion-derived neoantigen counts were strongly associated with the number of detected fusion events. Total fusion-derived candidate counts correlated positively with fusion event burden (ρ = 0.72, p-value = 7.29×10^{-6}), and this relationship was observed for both MHC class I (ρ = 0.65, p-value = 1.05×10^{-4}) and MHC class II candidates (ρ = 0.73, p-value = 5.37×10^{-6}). Together, these analyses show that EGG's neoantigen output depends on both the number and type of underlying genomic alterations, but is not a simple linear consequence of SNV burden. Candidate peptide yield is also influenced by alteration consequence, altered protein sequence length and context, patient-specific HLA genotype and MHC class."

Supplementary Figure S1. Relationship between input genomic event burden and predicted neoantigen yield across TCGA-SKCM samples.
(A) Total genomic variant-derived neoantigen yield compared with combined SNV + indel burden. (B) Total genomic variant-derived neoantigen yield compared with SNV burden alone. (C) Total fusion-derived neoantigen yield compared with fusion event burden. Each point represents one TCGA-SKCM sample. Spearman correlation coefficients and p-values were calculated across the 30 samples and are reported in Supplementary Table S1. Genomic variant-derived neoantigens refer to candidates generated from somatic SNV and indel calls using pVACseq, whereas fusion-derived neoantigens refer to candidates generated from STAR-Fusion calls using pVACfuse.

#unfortunately we are struggling to add tables and figures to the sumbmission system but we have also provided this document as a pdf in the supplementary so it might be clearer.

response response label predictor label samples spearman rho p_value
genomic total neoantigens Genomic total neoantigens SNV count 30 0.264 0.1595
genomic total neoantigens Genomic total neoantigens Indel count 30 0.378 0.0392
genomic total neoantigens Genomic total neoantigens SNV + indel count 30 0.287 0.1238
genomic mhcI neoantigens Genomic MHC I neoantigens SNV + indel count 30 0.274 0.1429
genomic mhcII neoantigens Somatic MHC II neoantigens SNV + indel count 30 0.306 0.0997
fusion total neoantigens Fusion total neoantigens Fusion event count 30 0.720 7.286e-06
fusion mhcI neoantigens Fusion MHC I neoantigens Fusion event count 30 0.649 1.053e-4
fusion mhcII neoantigens Fusion MHC II neoantigens Fusion event count 30 0.727 5.366e-06
fusion_total_neoantigens Fusion total neoantigens SNV count 30 -0.028 0.8826
fusion_total_neoantigens Fusion total neoantigens SNV + indel count 30 -0.035 0.8531
Supplementary Table S1. Spearman correlations between predicted neoantigen yield and input genomic event burden across TCGA-SKCM samples. The table reports Spearman correlation coefficients and p-values calculated across the 30 TCGA-SKCM samples to assess whether variability in predicted candidate neoantigen counts was associated with the number of underlying genomic events. Genomic neoantigens correspond to somatic SNV- and indel-derived candidates generated with pVACseq. Fusion neoantigens correspond to fusion-derived candidates generated with pVACfuse using STAR-Fusion input. Correlations were evaluated for total candidate counts and, where indicated, separately for MHC class I and MHC class II candidates. SNV + indel count represents the combined burden of somatic SNVs and indels per sample.

A5: PPI are typically one of things that are used for the construction of gene ontologies and gene pathway annotations. It actually looks like this relationship is partially recovered in the heat map in Figure 6A, where Borda correlates with the graph metrics. Could the authors comments on the actual impact of adding or removing the gene ontologies in the final antigen prioritization? Is it necessary?

In the current implementation of EGG, GO, Hallmark, and related pathway annotations are not directly used in the Borda ranking. Instead, they are added after prioritization as biological annotations to support interpretation of the final candidate list. Therefore, adding or removing these annotation fields does not alter Borda scores or ranks.

To address the reviewer's broader question about the contribution of PPI-derived information, we performed a PPI-topology ablation analysis. In this analysis, we removed the topology-derived PPI/network metrics from the ranking (Net_Betweenness, Net_Degree, Net_Impact/LCI, and Net_Strength) while retaining MHC-IC50, DepMap survivability score, and WCI. WCI was retained because it derives only from the patient-specific cancer co-expression network rather than from the direct PPI/topology. To isolate the effect of removing the PPI-topology metrics, we kept the weights of the remaining components unchanged and reassigned the removed network-topology weight to WCI. The resulting PPI-topology ablated ranking remained only moderately correlated with the original rank. Across the 30 selected TCGA melanoma samples, the median Spearman correlation between the original and ablated rankings was 0.79, with an interquartile range of 0.72–0.86. The median top-10 overlap was 7/10 candidates, with an interquartile range of 6-8/10, and the median absolute rank shift was 5 positions. These results indicate that PPI-topology metrics have a measurable effect on candidate ordering and on the composition of the highest-priority shortlist. Despite this analysis was aimed at fulfilling the reviewer's question, we kindly remark that the usage of GO annotation is not involved in the construction of the rank of candidate neoantigens, and its use is limited to the biological contextualization of candidates. Below, we summarize the results of the PPI-topology ablation analysis.

Supplementary Figure S4. Effect of removing PPI/topology-derived metrics from the final Borda ranking. (A) Distribution of sample-level Spearman correlations between the original ranking and the PPI/topology-ablated ranking across the 30 TCGA-SKCM samples. (B) Distribution of top-10 overlap counts between the original ranking and the PPI/topology-ablated ranking. In this ablation, Betweenness, Degree, LCI, and Strength were removed, while MHC-IC50, DepMap survivability score, and WCI were retained. The weight previously assigned to the removed topology metrics was reassigned to WCI to preserve the overall balance between antigen-prediction, essentiality, and patient-specific network context. The moderate rank concordance and partial top-10 overlap indicate that PPI/topology-derived metrics have a measurable impact on final prioritisation.

A6: Authors write: "The fraction of epitopes deriving from known cancer drivers is enriched among the top prioritized epitopes compared to the overall pool" and refer to the pie chart. To me, enrichment is usually quantified with a statistical test. Looking at values alone, I wouldn't say it's particularly enriched, considering the largest proportion is the overall pool. Could authors clarify what they mean by enriched (and possibly perform a statistical test to quantify enrichment)?

We recognize that the term "enriched" should be supported by statistical testing rather than inferred by purely observational hypothesis on the pie chart alone. Therefore, we have performed a Fisher's exact test to evaluate the statistical overrepresentation of neoantigens deriving from cancer driver genes within the top-10 prioritized set against the remaining candidate pool.
In this analysis, we considered as "driver-derived epitopes" all the epitopes beloning to the Driver-LoF, Driver-Activation, and Driver-Act+LoF categories and we compare them against "non-driver epitopes". Driver-derived epitopes remained a minority of the top-ranked candidates, but they were significantly overrepresented relative to the remaining candidate pool. Specifically, driver-derived epitopes represented 34/296 top-10 candidates (11.5%), compared with 133/1992 non-top-10 candidates (6.7%). This corresponds to an odds ratio equal to 1.81 with a one-sided Fisher's exact p-value = 0.0034. To showcase these results, we have updated the text in the Results section clarifying these aspects as requested by the reviewer. The text was modified as follows:

"Driver-associated epitopes showed a modest but significant overrepresentation among the top prioritized candidates compared with the remaining candidate pool. As shown in Figure 6B, driver-derived epitopes accounted for 34/296 top-10 candidates across samples (11.5%), compared with 133/1992 non-top-10 candidates (6.7%; one-sided Fisher's exact test, odds ratio = 1.81, p-value = 0.0034). These driver-derived candidates included loss-of-function, activating, and mixed activating/loss-of-function driver annotations. Although most top-ranked epitopes were still derived from non-driver genes, the relative overrepresentation of driver-derived candidates is consistent with the inclusion of functional and network-context features in the prioritization framework. One possible explanation is that some cancer driver genes occupy more central or functionally important positions in the network, although this interpretation remains

exploratory."

A7- A key limitation of the current study is the absence of benchmarking against experimentally validated neoantigens. Several melanoma epitopes have been previously identified and functionally validated, including targets derived from TCGA-associated datasets and follow-up immunological studies. Comparing EGG-prioritized candidates against such known immunogenic epitopes would provide an essential test of the framework's central premise, namely, that integrating gene indispensability improves the biological relevance of predicted targets. Even a retrospective analysis demonstrating that validated epitopes rank higher under the EGG framework than under conventional binding-based approaches would substantially strengthen the manuscript and provide empirical support for its proposed advantage.

We agree that benchmarking against experimentally validated neoantigens is crucial to test whether EGG prioritization improves the biological relevance of predicted targets beyond conventional binding-based ranking. In response, we added a new retrospective validation analysis comparing the final EGG/Borda ranking with an IC50-only ranking within each TCGA melanoma patient.
To achieve this goal we interrogated external datasets such as IEDB, CEDAR, and TSNAdb V2. To achieve this goal we interrogated external datasets such as IEDB, CEDAR, and TSNAdb V2. The corresponding database resources were accessed through the IEDB export page (https://www.iedb.org/database_export_v3.php), the CEDAR export page (https://cedar.iedb.org/database_export_v3.php), the CEDAR MHC ligand full export (https://cedar.iedb.org/downloader.php?file_name=doc/mhc_ligand_full_single_file.zip), and the TSNAdb v2.0 resource page (https://pgx.zju.edu.cn/tsnadb/). Specifically, we compared the final EGG/Borda ranking against an IC50-only ranking within each patient. Therefore, we assessed whether candidates ranked near the top by each method were enriched for external evidence from IEDB, CEDAR, and TSNAdb by extrapolating only the most reliable epitopes with experimental evidence of immunogenicity.
The selected experimental evidence categories included in our investigation are reported in the Table below:
Harmonized category Source evidence included Rationale
presentation Positive MHC ligand / antigen-presentation evidence from IEDB or CEDAR; TSNAdb tier 3 validated entries Supports experimental peptide presentation or presentation-associated validation
strong_immunogenicity Positive T-cell assay evidence from IEDB or CEDAR; TSNAdb tier 2 validated entries Supports functional immunogenicity / T-cell recognition
strong_immunogenicity_and_presentation TSNAdb tier 1 validated entries Highest-confidence category combining immunogenicity and presentation/validation support
Binding-only evidence, prediction-only concordance, negative assays, and uncategorized ligand records were excluded because we considered as reliable for this validation only endpoints that showed an experimental evidence of both presentation and immunogenic reaction, by disregarding entities supported by a binding-only evidence or a mere computational prediction.
We first evaluated exact peptide matching to test whether the full EGG candidate peptide was identical to a validated peptide in the reference databases. As expected, exact full-length matches were extremely rare: only one EGG candidate exactly matched a validated peptide in IEDB/CEDAR, despite more than one million unique experimentally supported reference peptides in each database. This confirms that exact matching alone is too sparse to support a meaningful ranking comparison.
Database Experimentally supported reference rows Unique experimentally supported reference peptides EGG candidates with exact peptide match EGG candidates with relaxed min-8 peptide match

CEDAR 4,346,554 1,223,992 1 542
IEDB 4,562,446 1,329,895 1 545
TSNAdb_v2 1,854 1,666 0 5

We therefore used a relaxed min-8 containment match as the main analysis. In this approach, a candidate was counted as supported if either the EGG candidate peptide contained the database peptide or the database peptide contained the EGG candidate peptide, requiring the contained sequence to be at least 8 amino acids long. This is appropriate because experimentally reported MHC ligands and T-cell epitopes often represent shorter presented or reactive peptide cores, whereas EGG candidate sequences can be longer, particularly for class-II or window-based predictions.
Using this relaxed min-8 endpoint, Borda ranking improved recovery of externally supported candidates

compared with IC50-only ranking at the most selective top-N cutoffs. In the top-10 candidates per patient, Borda increased the median hit rate relative to IC50-only ranking, and the same trend was observed in the top-20 candidates per patient. Exact-match analyses were also performed, but the number of exact matches was too small to distinguish ranking methods reliably.
These results provide orthogonal database-supported evidence that EGG prioritization can promote candidates containing experimentally supported presented or immunogenic peptide cores beyond IC50 ranking alone. We have framed this as a bioinformatic support for the biological relevance of the prioritization framework rather than as direct functional validation or proof of clinical durability. We have also clarified this limitation in the revised manuscript.

Supplementary Figure S6. Recovery of experimentally supported external epitope evidence by EGG/Borda ranking compared with IC50-only ranking. (A) Median difference in hit rate between each ranking method and IC50-only ranking across top-N candidate cutoffs within each patient, using relaxed min-8 containment matching against experimentally supported external evidence from IEDB, CEDAR, and TSNAdb. Positive values indicate higher recovery of externally supported candidates than IC50-only ranking. (B) Median difference in hit rate between each ranking method and IC50-only ranking across top-percentile cutoffs within each patient, using the same relaxed min-8 containment endpoint. Exact full-length peptide matching was also evaluated but was too sparse to support robust ranking comparisons. The relaxed min-8 analysis indicates that EGG/Borda ranking improves recovery of candidates containing experimentally supported presented or immunogenic peptide cores relative to IC50-only ranking at the most selective top-ranked cutoffs.

These results support that the EGG/Borda ranking can enrich top-ranked TCGA melanoma candidates for experimentally supported presented or immunogenic peptide cores relative to IC50-only ranking.

A8- Another point that warrants clarification is the apparent enrichment of longer peptide sequences among the top-ranked candidates (e.g., a 29-amino acid peptide). Antigen binding prediction algorithms (particularly for HLA class II) are known to exhibit length-related biases, often favoring longer peptides due to the flexibility of binding cores and scoring heuristics. It would therefore be important to determine whether peptide length is inadvertently influencing the EGG ranking. Providing a comparison of peptide length distributions before and after prioritization, and assessing whether length correlates with final rank, would help rule out unintended algorithmic bias and ensure that prioritization reflects biological relevance rather than systematic properties of the prediction tools.

To assess whether peptide length was inadvertently influencing EGG prioritization, we compared peptide length distributions before and after prioritization and tested whether peptide length correlated with final rank, analyzing MHC class I and class II candidates separately.
We did not observe evidence of systematic peptide-length bias in the final ranking. For MHC class I candidates, the median peptide length was 11 amino acids in the full candidate pool and 11 amino acids among the top 10 prioritized epitopes per sample, with no significant difference between top-ranked and non-top-ranked candidates (Mann-Whitney U test, p-value = 0.246). For MHC class II candidates, the median peptide length was 24 amino acids in the full pool and 25 amino acids among the top 10 candidates per sample, again with no significant difference (p-value = 0.362).
We also tested whether peptide length correlated with final rank percentile. Using class-specific rank percentiles, the correlation was weak and non-significant for both MHC class I candidates (Spearman $\rho = -0.031$, p-value = 0.313) and MHC class II candidates ($\rho = 0.021$, p-value = 0.467). Similar results were obtained when using overall rank percentiles across each sample (MHC class I: $\rho = -0.055$, p-value = 0.075; MHC class II: $\rho = 0.028$, p-value = 0.326).
These analyses indicate that the presence of occasional long peptides among highly ranked candidates, including the example noted by the reviewer, does not reflect a general tendency of EGG to favor longer peptides. Rather, peptide length does not appear to be a systematic driver of prioritization in our framework. We have added both the peptide length distribution plot and the peptide length versus rank correlation plot in the Additional file 3 to clarify this point.

Supplementary Figure S2. Peptide length distribution and association with final prioritisation rank.
(A) Peptide length distribution before and after prioritisation, shown separately for MHC class I and MHC

class II candidates. “All” indicates the full candidate pool before final top-ranked selection, while “Top 10” indicates the top 10 prioritised candidates per sample. Differences between top-ranked and non-top-ranked candidates were evaluated using the Mann-Whitney U test. (B) Relationship between peptide length and final prioritisation rank percentile, shown separately for MHC class I and MHC class II candidates. Spearman correlation was used to assess whether peptide length was associated with final rank. The analyses showed no significant enrichment of longer peptides among top-ranked candidates and no meaningful correlation between peptide length and final prioritisation rank.

This analysis is now mentioned in the main text:
“To further evaluate potential determinants of prioritisation, we tested whether peptide length or tumor DNA VAF influenced final epitope rank. Neither feature showed a meaningful association with prioritisation outcome, supporting that EGG ranking was not primarily driven by peptide-length bias or VAF differences in this dataset (Additional File 3, Supplementary Figures 2 and 3).”

A9- Since the framework is motivated by evolutionary durability, it would be informative to assess whether top-ranked epitopes preferentially derive from clonal versus subclonal mutations. Incorporating or reporting cancer cell fraction estimates would help align the prioritization with the stated goal of evolutionary stability.

To address this point, we used tumor DNA variant allele frequency (VAF) as a simple proxy for cancer cell fraction (CCF) and clonality in the mutation-derived epitope set. Because formal CCF estimation would require sample-specific purity, copy-number, and local allelic imbalance correction, we used a conservative approximation assuming tumor purity of 1. Under this approximation, variants with tumor DNA VAF ≥ 0.25 were considered broadly compatible with clonal or near-clonal events, whereas lower-VAF variants would be more compatible with subclonal events.
We were able to match tumor DNA VAF values from the pVACseq outputs to all final somatic epitope candidates (1,737/1,737). Under the VAF ≥ 0.25 approximation, all matched somatic candidates were compatible with clonality, including all 185 somatic candidates that appeared within the top 10 ranked epitopes per sample. Therefore, within this dataset, the final somatic epitope pool did not contain a meaningful low-VAF/subclonal subset against which enrichment among top-ranked candidates could be tested.
Consistent with this, we did not observe evidence that the highest-ranked somatic epitopes preferentially derived from mutations with higher tumor DNA VAF. Across all matched somatic candidates, the median tumor DNA VAF was 0.383, compared with 0.385 among somatic epitopes falling within the top 10 overall candidates per sample, and this difference was not significant (Mann–Whitney p-value = 0.935). Likewise, tumor DNA VAF did not significantly correlate with final rank percentile overall (Spearman ρ = -0.020, p-value = 0.411). Similar results were observed when MHC classes were analyzed separately: MHC class I showed no correlation between VAF and rank percentile (ρ = -0.007, p-value = 0.839), and MHC class II also showed no significant correlation (ρ = -0.032, p-value = 0.335).
These results suggest that, in the current analysis, EGG prioritization was not strongly driven by tumor DNA VAF as a clonality proxy. Rather, the matched somatic candidates already fell within a VAF range compatible with clonal or near-clonal mutations under the simplified threshold used here. We have added this analysis to Additional File 3 to clarify the relationship between VAF and prioritization. For applications in which explicit clonality weighting is desired, tumor DNA VAF or formal CCF estimates can be incorporated as an additional user-defined feature in the Borda ranking framework. As suggested, we added the Tumor VAF in the final table results, in the Additional file 3 as well as we modified the pipeline now to include this as default.

Supplementary Figure S3. Tumor DNA VAF distribution and association with final prioritisation rank among somatic epitope candidates. (A) Tumor DNA variant allele frequency (VAF) distribution among all final somatic/genomic variant-derived epitope candidates compared with somatic candidates present among the top 10 ranked epitopes per sample. Tumor DNA VAF values were obtained from the pVACseq-derived candidate tables and were matched to all final somatic epitope candidates. The dashed threshold at VAF = 0.25 indicates the simplified cutoff used to classify variants as broadly compatible with clonal or near-clonal events under an assumed tumor purity of 1. (B) Relationship between tumor DNA VAF and final

prioritisation rank percentile for somatic epitope candidates, shown separately for MHC class I and MHC class II candidates. Spearman correlation was used to test whether tumor DNA VAF was associated with final prioritisation rank. No significant difference in VAF distribution was observed between all somatic candidates and top-10 somatic candidates, and no significant correlation was observed between VAF and final rank percentile.

This analysis is now mentioned in the main text:
"To further evaluate potential determinants of prioritisation, we tested whether peptide length or tumor DNA VAF influenced final epitope rank. Neither feature showed a meaningful association with prioritisation outcome, supporting that EGG ranking was not primarily driven by peptide-length bias or VAF differences in this dataset (Additional File 3, Supplementary Figures 2 and 3)."

A10- To me the merge between DepMap and pFINs is non-trivial. Since DepMap is constructed over a multitude of tumors and pFINs are patient-specific, how do authors justify using pan-cancer dependency scores as proxies for patient-specific tumor essentiality?

We thank the reviewer for this important point. We agree that the integration of DepMap with patient-specific functional interaction networks (pFINs) is non-trivial, (and we have clarified this rationale in the revised manuscript). In EGG, DepMap is not used as a direct substitute for patient-specific tumor essentiality. Rather, the patient-specific component is provided by the pFINs, which are derived from each sample's expression/network context, whereas DepMap contributes an orthogonal, experimentally measured fitness signal obtained from large-scale CRISPR dependency screens. In this sense, pan-cancer DepMap scores are used as a proxy for general cancer-cell indispensability, while pFIN topology captures the patient-specific context in which that gene is embedded. The two signals are therefore complementary rather than equivalent, and in EGG they are combined only at the consensus-ranking stage rather than merged into a single unified essentiality estimate.
Our justification for including DepMap is that large-scale dependency datasets capture recurrent survival constraints that are difficult to infer from one patient alone, including both broadly conserved and lineage-associated cancer vulnerabilities. At the same time, we agree with the reviewer that pan-cancer dependency scores cannot fully represent the exact essentiality landscape of an individual tumor, because they do not account for all patient-specific factors such as microenvironment, treatment history, or subtype-specific states. For this reason, we interpret DepMap as a complementary external prior that helps regularize prioritization, not as patient-specific ground truth.
Added this clarification in the main text:
"In this framework, the experimental dependency layer provides a broad cancer-cell fitness prior, while patient specificity is retained through the pFIN-derived topology computed from each sample."

A11- While the manuscript acknowledges tumor-intrinsic escape mechanisms, it does not explicitly discuss the limitation that immune-side determinants of immunogenicity beyond HLA binding (e.g., antigen processing efficiency, TCR repertoire constraints, immunodominance) are not modeled. I understand this is likely out of scope, since the authors sought to tackle the immune evasion problem from immune pressure, but I believe that clarifying the intended scope of the framework would strengthen the conceptual positioning of the study.

We answered the reviewer's comment by making the scope of the EGG framework clearer in the result and discussion section, the modified paragraph reads:

"Our strategy explicitly mitigates immune escape via deletion or silencing of dispensable neoantigen source genes. However, this focus also defines its main limitation: EGG does not model escape mechanisms that operate through impaired antigen presentation, nor does it capture several immune-side determinants of neoantigen immunogenicity beyond peptide-HLA binding, such as antigen processing efficiency, T-cell receptor repertoire constraints, and immunodominance. EGG should therefore be interpreted as a prioritization framework centered on the tumor-side evolutionary durability of neoantigen source genes, rather than as a complete predictor of anti-tumor immune recognition. Consistent with this limitation, genetic and epigenetic alterations in HLA class I/II, β2-microglobulin, antigen processing machinery, and

IFNγ signaling are frequent in clinical tumors and represent dominant routes of immune evasion under T cell pressure [80–82]. In such settings, neoantigen source gene essentiality becomes largely irrelevant, as tumor cells can evade recognition despite maintaining expression of the targeted gene.”

****

B- Minor comments and typos:

B1: There needs to be consistency in the formula letters used. For instance, N is used for the number of samples, number of genes as well as the scaling factor from the LIONESS algorithm. Alpha is not defined in the LIONESS-derived formula. It would make it a lot easier to follow if there is consistency.
We thank the reviewer for this comment. As suggested we improved the clarity of the formulas, we are now using S instead on N for the samples and explained the formula more clearly in the text:
“To estimate individual level gene co-expression patterns, we applied the LIONESS (Linear Interpolation to Obtain Network Estimates for Single Samples) framework [58]. For a set of S samples, the aggregate network was computed using Pearson correlation between all gene pairs (i,j) across the full expression matrix.
To estimate the network specific to a sample q, we first denote the aggregate network computed across all samples as $e_{ij}^{\left(\alpha\right)}$, and the corresponding leave-one-out aggregate network excluding sample q as eijαqThe individual sample network $e_{ij}^{\left(q\right)}$\ was then derived using the LIONESS formula:

$$e_{ij}^{\left(q\right)}=S\left(e_{ij}^{\left(\alpha\right)}-e_{ij}^{\left(\alpha-q\right)}\right)+e_{ij}^{\left(\alpha-q\right)}$$

"
B2- Authors should mention the number of genes they retain once they prune them out to give reader a sense of the network size.

We thank the reviewer for this helpful suggestion. We have now added a network-size summary for the pruned patient-specific functional interaction networks to the Additional file 3 in the supplementary material. Across the 30 melanoma pFINs, the filtered networks retained a median of 21,888 genes per patient, corresponding to 93.4% of the 23,446 VST-normalized genes, and a median of 181,255 edges. After pruning, the networks consisted of a large connected core together with a substantial number of isolated vertices: the largest connected component contained a median of 16,414 genes, corresponding to 75.0% of retained vertices, and contained nearly all retained edges. To make the effective network size clear, we now report both total retained genes and largest connected component statistics at the cohort level and per patient.

Samples	Original VST genes	Retained genes, median [min–max]	Retained genes, IQR	Retained genes, median % of VST	Edges, median [min–max]	LCC genes, median [min–max]	LCC genes, median % of retained	LCC edges, median [min–max]	Isolated genes, median [max]
30	23,446	21,888 [21,327–23,136]	21,657–22,066	93.4%	181,255 [170,853–185,124]	16,414 [15,869–17,667]	75.0%	181,243 [170,831–185,112]	5,454 [5,469]

To clarify the effective size of the pruned pFINs used for downstream prioritisation, we added a per-sample network-size summary. Across the 30 melanoma samples, the filtered pFINs retained a median of 21,888 genes [range: 21,327–23,136] from 23,446 VST-normalized genes, corresponding to a median retention of 93.4%. The filtered networks contained a median of 181,255 edges [range: 170,853–185,124]. Edge pruning produced a large connected core together with isolated vertices: the largest connected component contained a median of 16,414 genes [range: 15,869–17,667], representing 75.0% of retained vertices, and contained nearly all retained edges. Cohort-level and per-patient network-size statistics are provided in Supplementary Table S2.
We added this mention in the main text:
“To clarify the scale of the pruned patient-specific functional interaction networks used for prioritisation, we also quantified the number of retained genes, edges, largest connected component size, and isolated genes across all 30 melanoma samples; these network-size summaries are provided in Additional File 3, Supplementary Table S2.”

B3- In the gene co-expression graph, authors mention constructing a patient specific graph and they filter genes with less than 15 reads in 20% of samples. Am I understanding correctly that authors want genes included in the graph to be expressed in at least 24 people out of 30? Could authors comment on how stringent this is in then influencing their personalized network constructions?

We thank the reviewer for this comment. Genes were retained if they showed at least 15 reads in at least 20% of samples, which in our cohort corresponds to 6 of 30 samples. We agree that the original wording was ambiguous and have revised the manuscript to clarify this point.
This point has been clarified in the Methods section with the following added text:
"Gene level raw read count matrices previously produced in the RNA-seq processing step (described in the RNA-Seq Processing, Fusion Detection and HLA typing paragraph) were carried forward for patient-specific co-expression network generation. Genes were retained for network construction only if they had at least 15 raw reads in at least 20% of samples; all other genes were removed as a low-expression preprocessing step (in our cohort of 30 samples, this corresponds to requiring detectable expression in at least 6 samples). This filtering step was applied to reduce noise from genes with very low or infrequent expression, which are less likely to support stable correlation estimates in co-expression-based network inference. similar low-expression filtering steps are commonly used in RNA-seq network analysis workflows, including LIONESS-based analyses of TCGA and GTEx data [55,56]."

B4- Authors write "(..) can identify not just any neoantigens, but those most likely able to elicit appropriate immune responses and to remain resistant to evolutionary changes in the cancer. To address this challenge, we developed ". That sentence should be rephrased because authors have not addressed immune response, they only tackle evolutionary pressure.

We agree that the original wording overstated the scope of the current framework by implying that immune response was directly addressed. Our analysis prioritizes neoantigen candidates using predicted antigen presentation, functional relevance, and network/evolutionary context, but it does not experimentally assess T-cell immunogenicity.
We have therefore revised the sentence to remove the claim about eliciting immune responses and to focus on the aspect directly addressed by EGG:
"The complexity of cancer immunotherapy requires sophisticated computational approaches that can identify not just any neoantigens, but those most likely able to remain resistant to evolutionary changes in the cancer."
This revised wording better reflects the intended contribution of the framework and avoids implying direct validation of immune reactivity.

B5- In the results section, all tools used should be properly cited.

We have revised the Results section to ensure that all software tools mentioned there are properly cited at their first relevant occurrence. We also checked the Methods and reference list to ensure that each tool includes the appropriate software citation, version information, and, where available, the associated software paper or persistent identifier, in line with GigaScience software citation guidance.

B6- "with other optional pvactools algorithms outputs ", pVACtools should be capitalized properly
We have corrected the capitalization from "pvactools" to "pVACtools" throughout the manuscript.

B7- When authors mention "Across the TCGA melanoma cohort", is this the entire melanoma cohort, or the 30 samples they selected? This sentence should be edited to clarify.

The phrase referred to the selected TCGA-SKCM subset used in this analysis, not the entire TCGA melanoma cohort. We have revised the wording to clarify this:
"across the 30 selected samples from the TCGA melanoma cohort."
B8- When authors write: "bridging the gap between computational prediction and the practical constraints of vaccine formulation." I'm not sure I follow what the practical constraint is. Once the reader finishes the entire manuscript, my best guess is the practical constraint is the number of peptides to deliver in the vaccine. Perhaps clarifying that sentence would help dispel any ambiguity.

We agree that the original wording was ambiguous. We have revised the sentence to make this explicit: “Moreover, by adding sample-specific co-expression network analysis as a second-tier prioritization, EGG reduces large candidate pools into a smaller set of biologically relevant and therapeutically promising targets, bridging the gap between computational prediction and the practical need to select a limited number of epitopes for inclusion in a vaccine formulation.”

B9- Authors should always label the corresponding figure number when writing about a specific panel. Example "(12 samples; panel C)" should be Figure XC etc.

Thank you for noticing this. We modified the figure labels throughout the manuscript according to the reviewer’s suggestion,

B10- Authors should add the MSI abbreviation to their list of abbreviations and define it in the text.

We have defined microsatellite instability as microsatellite instability (MSI) at first mention in the text and added it to the abbreviation list.

****

C- Figure-related comments.

C1- In Figure 1, the Modules 0,1 etc. should be numbered for better reference for readers.
We have modified the image by adding the relevant module numbers..

C2- Figure 6 needs polishing. The y and x ticks are merging with the negative signs. The labels still have underscores in them and are hard to read. C and D are ambiguous: does 1 mean 1 other person has the same peptide (2 occurences) or 1 means only 1 person has the peptide (1 occurence)?

We thank the reviewer for this helpful comment. We have revised Figure 6 to improve readability by correcting the tick formatting, removing underscores from the labels, and using clearer axis and panel titles. In particular, for panels C and D we clarified that the x-axis indicates the number of samples containing the gene or epitope sequence. Therefore, a value of 1 means that the gene or epitope was observed in one sample only, not in one additional sample. We also updated the wording of the panel labels/titles accordingly to make this interpretation explicit.

Reviewer 2:

Reviewer #2: The manuscript introduces the Epitope Generation Gateway (EGG), a containerized Snakemake pipeline designed to bridge the gap between raw neoantigen discovery and systems-level tumor biology. By integrating established variant-calling workflows with a novel Patient-Specific Functional Interaction Network (pFIN) layer, the authors address a critical limitation in current pipelines: the tendency to prioritize epitopes based on sequence-level affinity while overlooking the evolutionary durability and functional essentiality of the source genes.
The methodological framework—leveraging single-sample co-expression inference (LIONESS) and multi-feature Borda rank aggregation—is both well-motivated and timely. We appreciate the authors' commitment to open science by providing a transparent, containerised workflow and for providing a clear proof-of-concept application using 30 TCGA patients with cutaneous melanoma.. This work has significant potential to enhance the rational selection of durable vaccine targets. However, to transition from a conceptual framework to a robust clinical utility, the following points regarding statistical stability, ranking logic, and validation should be addressed.

Major Revisions
1. Functional Validation of the Prioritization Framework
The hypothesis that network-central, functionally essential genes yield more immune-escape-resistant neoantigen targets is biologically compelling and well-supported by the cited literature. While we appreciate the "proof-of-concept" framing, the claims related to durability made throughout the introduction and

discussion require more direct support to move beyond speculation.
* Recommendation: Ideally, the authors would provide evidence that network-central epitopes are enriched for biologically meaningful targets. Immunogenicity testing of a subset of top-ranked versus lower-ranked epitopes — through T-cell reactivity assays or MHC-eluted peptide confirmation — would provide the most direct evidence that the network-based prioritization identifies biologically meaningful targets beyond what MHC binding prediction alone achieves.
* A more feasible bioinformatic approach would be to perform an enrichment analysis against high-quality immunopeptidomics datasets (e.g., MHC ligands identified by MS from melanoma cell lines) to determine whether epitopes that are specifically elevated in rank by the network-centrality layer — relative to their MHC-binding-only rank — are more likely to correspond to experimentally presented peptides. This would directly isolate the contribution of the network prioritization module from the baseline pVACtools prediction.

We thank the reviewer for this important comment. We agree that direct T-cell reactivity testing or MHC-eluted peptide validation would provide the strongest functional validation of the prioritization framework. These experiments are beyond the scope of the current study, but we have added a new computational validation to address the reviewer's feasible suggested analysis: whether EGG prioritization recovers candidates with independent experimental support more effectively than MHC binding prediction alone.
To achieve this goal, we interrogated external datasets such as IEDB, CEDAR, and TSNAdb V2. The corresponding database resources were accessed through the IEDB export page (https://www.iedb.org/database_export_v3.php), the CEDAR export page (https://cedar.iedb.org/database_export_v3.php), the CEDAR MHC ligand full export (https://cedar.iedb.org/downloader.php?file_name=doc/mhc_ligand_full_single_file.zip), and the TSNAdb v2.0 resource page (https://pgx.zju.edu.cn/tsnadb/). Specifically, we compared the final EGG/Borda ranking against an IC50-only ranking within each patient. Therefore, we assessed whether candidates ranked near the top by each method were enriched for external evidence from IEDB, CEDAR, and TSNAdb by extrapolating only the most reliable epitopes with experimental evidence of immunogenicity.
The selected experimental evidence categories included in our investigation are reported in the Table below:

Harmonized category	Source evidence included	Rationale
presentation	Positive MHC ligand / antigen-presentation evidence from IEDB or CEDAR; TSNAdb tier 3 validated entries	Supports experimental peptide presentation or presentation-associated validation
strong_immunogenicity	Positive T-cell assay evidence from IEDB or CEDAR; TSNAdb tier 2 validated entries	Supports functional immunogenicity / T-cell recognition
strong_immunogenicity_and_presentation	TSNAdb tier 1 validated entries	Highest-confidence category combining immunogenicity and presentation/validation support

Binding-only evidence, prediction-only concordance, negative assays, and uncategorized ligand records were excluded because we considered as reliable for this validation only endpoints that showed an experimental evidence of both presentation and immunogenic reaction, by disregarding entities supported by a binding-only evidence or a mere computational prediction.
We first evaluated exact peptide matching to test whether the full EGG candidate peptide was identical to a validated peptide in the reference databases. As expected, exact full-length matches were extremely rare: only one EGG candidate exactly matched a validated peptide in IEDB/CEDAR, despite more than one million unique experimentally supported reference peptides in each database. This confirms that exact matching alone is too sparse to support a meaningful ranking comparison.

Database	Experimentally supported reference rows	Unique experimentally supported reference peptides	EGG candidates with exact peptide match	EGG candidates with relaxed min-8 peptide match
CEDAR	4,346,554	1,223,992	1	542
IEDB	4,562,446	1,329,895	1	545
TSNAdb_v2	1,854	1,666	0	5

We therefore used a relaxed min-8 containment match as the main analysis. In this approach, a candidate was counted as supported if either the EGG candidate peptide contained the database peptide or the database peptide contained the EGG candidate peptide, requiring the contained sequence to be at least 8 amino acids long. This is appropriate because experimentally reported MHC ligands and T-cell epitopes

often represent shorter presented or reactive peptide cores, whereas EGG candidate sequences can be longer, particularly for class-II or window-based predictions.
Using this relaxed min-8 endpoint, Borda ranking improved recovery of externally supported candidates compared with IC50-only ranking at the most selective top-N cutoffs. In the top-10 candidates per patient, Borda increased the median hit rate relative to IC50-only ranking, and the same trend was observed in the top-20 candidates per patient. Exact-match analyses were also performed, but the number of exact matches was too small to distinguish ranking methods reliably.
These results provide orthogonal database-supported evidence that EGG prioritization can promote candidates containing experimentally supported presented or immunogenic peptide cores beyond IC50 ranking alone. We have framed this as a bioinformatic support for the biological relevance of the prioritization framework rather than as direct functional validation or proof of clinical durability. We have also clarified this limitation in the revised manuscript.

Supplementary Figure S6. Recovery of experimentally supported external epitope evidence by EGG/Borda ranking compared with IC50-only ranking. (A) Median difference in hit rate between each ranking method and IC50-only ranking across top-N candidate cutoffs within each patient, using relaxed min-8 containment matching against experimentally supported external evidence from IEDB, CEDAR, and TSNAdb. Positive values indicate higher recovery of externally supported candidates than IC50-only ranking. (B) Median difference in hit rate between each ranking method and IC50-only ranking across top-percentile cutoffs within each patient, using the same relaxed min-8 containment endpoint. Exact full-length peptide matching was also evaluated but was too sparse to support robust ranking comparisons. The relaxed min-8 analysis indicates that EGG/Borda ranking improves recovery of candidates containing experimentally supported presented or immunogenic peptide cores relative to IC50-only ranking at the most selective top-ranked cutoffs.

These results support that the EGG/Borda ranking can enrich top-ranked TCGA melanoma candidates for experimentally supported presented or immunogenic peptide cores relative to IC50-only ranking.

2. Evaluation of Network Stability and Limit of Detection (n=30)
Integrating the LIONESS framework to estimate patient-specific networks is a compelling methodological feature. However, at a sample size of n = 30, the mathematical stability of the co-expression manifold requires closer examination. In smaller cohorts, leave-one-out interpolations can be sensitive to sampling variance and transcriptomic outliers.
* Network Volatility: Since the standard error of Pearson correlation at n = 30 is high (~0.19), the authors should provide a bootstrapping or jackknife analysis. This would demonstrate that the centrality ranks (WCI/LCI) of the prioritized genes are robust and not driven by stochastic noise.
* Edge Consistency: Reporting the Jaccard similarity of the top-ranked edges across the 30 samples would clarify whether the pFINs reflect a unique biological signal or the variance inherent to small-sample interpolation.
* External Benchmarking: To demonstrate that EGG captures meaningful tumor biology rather than baseline noise, the authors should compare their centrality ranks against a stable baseline. This could be achieved by using either the full TCGA-SKCM cohort (~470) or a large external melanoma dataset (e.g., from GEO or EGA).

We thank the reviewer for this important comment. To evaluate whether patient-specific networks inferred from the n = 30 cohort were sensitive to sampling variance or individual transcriptomic outliers, we performed a leave-one-background-sample-out jackknife analysis using the same Pearson-LIONESS framework as in the original workflow. For each target patient, we retained the target sample and recomputed the patient-specific network after removing each of the other 29 samples from the background cohort, yielding 870 same-patient jackknife comparisons. For each comparison, we recalculated the network metrics and compared the resulting gene-level ranks against the original full-cohort ranks for the same target patient.
The resulting centrality ranks were highly stable. WCI showed a median Spearman rank correlation of 0.997 relative to the original full-cohort ranks, with a minimum of 0.990. On the PPI/membrane-filtered networks used for downstream prioritisation, degree, strength, and LCI also showed high stability, with

median Spearman correlations of 0.998, 0.997, and 0.996, respectively. Betweenness was more sensitive, as expected for a shortest-path-based metric, but remained concordant with the original ranks, with median Spearman $\rho = 0.933$ and minimum $\rho = 0.862$. The top-ranked genes were also largely preserved, with median top-1% overlap ranging from 91.8% to 95.0% across metrics.
To address edge consistency, we additionally quantified Jaccard similarity of top-ranked pFIN edge sets. For the same patient, original-vs-jackknife top-edge sets were stable, with median Jaccard values of 0.784, 0.800, 0.808, and 0.839 for the top 1%, 5%, 10%, and 20% edges, respectively. These corresponded to median overlaps of 88.0 to 91.3% relative to the smaller top-edge set. In contrast, pairwise comparisons between the 30 original patient pFINs showed very low overlap of top-ranked edges, with median Jaccard values of 0.000 for the top 1–10% edge sets and 0.005 for the top 20% edge set. Thus, top-ranked edges were stable within a patient under jackknife perturbation but were not simply shared across all patients.

To address the reviewer’s request for external benchmarking, we also performed a matched public-data benchmark using TCGA-SKCM primary-tumor RNA-seq data downloaded from GDC STAR-count files. Because the public benchmark was based on primary-tumor samples with available open-access gene-level counts, the benchmark cohort contained 103 TCGA-SKCM primary-tumor samples rather than the full approximately 470-sample TCGA-SKCM cohort. To separate cohort-size effects from preprocessing/source effects, we first rebuilt the same 30 target-patient networks using the public GDC primary-tumor count data alone, and then recomputed the same 30 target-patient networks after embedding those samples in the larger $n = 103$ TCGA-SKCM primary-tumor background. Thus, this comparison evaluates the effect of using a larger TCGA-derived background while keeping the public GDC preprocessing source constant.

In this matched-GDC benchmark, WCI ranks were strongly concordant between the $n = 30$ and $n = 103$ backgrounds, with median Spearman $\rho = 0.942$ and minimum Spearman $\rho = 0.910$. On the PPI/membrane-filtered networks, degree, strength, and LCI also showed high concordance, with median Spearman correlations of 0.950, 0.937, and 0.929, respectively. Betweenness again showed lower concordance, with median Spearman $\rho = 0.675$, consistent with its greater sensitivity to changes in shortest-path structure. For top-ranked genes, the exact top 1% boundary was more sensitive to the background cohort, but broader high-ranked gene sets were more stable. For example, WCI top-gene overlap increased from 56.7% at the top 1% to 82.2% at the top 20%, and degree top-gene overlap increased from 72.7% at the top 1% to 89.3% at the top 20%. These results support broad stability of the centrality signal under a larger TCGA-derived background, while also showing that the most extreme top-ranked genes remain partly cohort-background dependent.

Together, these analyses indicate that the pFIN-derived centrality features used by EGG are robust to leave-one-sample perturbation of the $n = 30$ cohort, retain patient-specific edge structure, and show substantial concordance with a larger TCGA-SKCM primary-tumor benchmark. We have added these analyses and summary statistics to the revised Additional file 3 in the supplemententray material.

Metric	Network level	N	Median Spearman ρ	Minimum Spearman ρ	Median Kendall τ	Top 1% median overlap	Top 1% minimum overlap	Top 1% median Jaccard
WCI	Full LIONESS network	870	0.997	0.990	0.957	93.6%	64.7%	0.880
Degree	PPI/membrane-filtered network	870	0.998	0.994	0.984	95.0%	79.5%	0.905
Strength	PPI/membrane-filtered network	870	0.997	0.989	0.973	94.0%	72.6%	0.884
LCI	PPI/membrane-filtered network	870	0.996	0.987	0.970	94.0%	72.6%	0.884
Betweenness	PPI/membrane-filtered network	870	0.933	0.862	0.900	91.8%	74.4%	0.848

Supplementary Table S3. Jackknife stability of pFIN-derived centrality ranks. For each target patient, patient-specific networks were recomputed after removing each of the other 29 background samples, yielding 870 same-patient jackknife comparisons across the 30 TCGA-SKCM samples. For each comparison, gene-level centrality ranks from the jackknife network were compared with the corresponding ranks from the original full-cohort network. The table reports Spearman rank correlation, Kendall τ, top-1% overlap, and top-1% Jaccard similarity for WCI, degree, strength, LCI, and betweenness.

Analysis	Top edge fraction considered	Median Jaccard	Median overlap / smaller top set
Same patient: original vs jackknife	1%	0.784	88.0%

Same patient: original vs jackknife	5%	0.800	88.9%
Same patient: original vs jackknife	10%	0.808	89.4%
Same patient: original vs jackknife	20%	0.839	91.3%
Between patients: original pFINs	1%	0.000	0.0%
Between patients: original pFINs	5%	0.000	0.0%
Between patients: original pFINs	10%	0.000	0.0%
Between patients: original pFINs	20%	0.005	0.9%

Supplementary Table S4. Edge consistency of pFIN top-ranked edge sets. Top-ranked edge sets were compared using Jaccard similarity and overlap relative to the smaller top-edge set. Same-patient comparisons evaluate the stability of top edges between the original pFIN and jackknife-derived pFINs. Between-patient comparisons evaluate the overlap of top-ranked edges across the 30 original patient pFINs. The results distinguish within-patient edge stability from shared edge structure across different patients.

Metric	Median Spearman ρ	Minimum Spearman ρ	Median Kendall τ	Top 1% median overlap	Top 1% minimum overlap	Top 1% median Jaccard
WCI	0.942	0.910	0.786	56.7%	42.4%	0.365
Degree	0.950	0.935	0.890	72.7%	63.1%	0.569
Strength	0.937	0.917	0.850	61.5%	48.1%	0.444
LCI	0.929	0.912	0.840	61.5%	47.7%	0.444
Betweenness	0.675	0.619	0.616	63.4%	53.3%	0.465

Supplementary Table S5. Matched GDC benchmark comparing pFIN centrality ranks generated using n = 30 and n = 103 TCGA-SKCM primary-tumor backgrounds. The 30 target-sample networks were reconstructed using public GDC STAR-count RNA-seq data and compared with corresponding networks generated after embedding the same target samples in a larger TCGA-SKCM primary-tumor background of 103 samples. This benchmark was designed to evaluate the effect of increasing the background cohort size while keeping the data source and preprocessing framework consistent. The table reports rank concordance and top-gene overlap metrics for WCI, degree, strength, LCI, and betweenness.

To address the reviewer’s request for external benchmarking, we also performed a matched public-data benchmark using TCGA-SKCM RNA-seq data downloaded from GDC STAR-count files [https://docs.gdc.cancer.gov/Encyclopedia/pages/STAR_Counts/]. In this analysis, the 30 target sample networks included in our study were reconstructed from public GDC data and compared with the entire TCGA-SKCM cohort, including 469 samples. In this analysis, we utilized only genes in common between the two groups. In this way, we evaluated the effect of replacing the smaller background composed by 30 samples with a larger TCGA-derived background, which is expected to provide a better representation of the patient population.

In our results, WCI showed substantial concordance between the n = 30 and n = 469 backgrounds, with median Spearman ρ = 0.824. On the PPI/membrane-filtered networks, degree and strength also showed moderate-to-substantial concordance, with median Spearman ρ = 0.771 and 0.772, respectively. Betweenness showed moderate concordance, with median Spearman ρ = 0.707. LCI was more sensitive to the larger background cohort, with median Spearman ρ = 0.465, indicating that this metric is more dependent on the inferred network background. The top-gene overlap analysis showed that broader high-ranked gene sets were more stable than the exact top 1% boundary. At the top 1% threshold, median overlap was 52.6% for WCI, 73.2% for degree, 66.5% for strength, 43.5% for LCI, and 57.4% for betweenness. At the broader top 20% threshold, median overlap increased to 74.8%, 77.7%, 76.2%, 78.2%, and 67.5%, respectively. These results support broad concordance of the main centrality signals under a larger background, while also showing that the most extreme top-ranked genes and LCI rank ordering are more cohort-background dependent.

Metric	Median Spearman ρ	Minimum Spearman ρ	Median Kendall τ	Top 1% median overlap	Top 1% minimum overlap	Top 1% median Jaccard	Top 20% median overlap	Top 20% median Jaccard
WCI	0.824	0.686	0.628	52.6%	30.4%	0.357	74.8%	0.597
Degree	0.771	0.700	0.623	73.2%	57.4%	0.578	77.7%	0.635
Strength	0.772	0.702	0.594	66.5%	52.8%	0.498	76.2%	0.616
LCI	0.465	0.285	0.456	43.5%	30.8%	0.278	78.2%	0.642
Betweenness	0.707	0.587	0.582	57.4%	46.1%	0.403	67.5%	0.510

Supplementary Table S6. Extended GDC benchmark comparing pFIN centrality ranks generated using n = 30 and n = 469 TCGA-SKCM backgrounds. The 30 target-sample networks were compared with corresponding networks generated using a larger TCGA-SKCM background of 469 samples, restricting the analysis to genes common to both network reconstructions. This benchmark evaluates the stability of pFIN-derived centrality ranks under a substantially larger TCGA-derived background. The table reports rank concordance and top-gene overlap metrics for WCI, degree, strength, LCI, and betweenness, including top-1% and top-20% overlap statistics.

In the main text we added:
"We further assessed the robustness and contribution of the pFIN network layer through several sensitivity analyses. Specifically, we evaluated pFIN stability using leave-one-background-sample-out jackknife analyses, top-edge consistency testing, and two GDC TCGA-SKCM benchmarks using larger n = 103 and n = 469 background cohorts. We also performed a membrane-edge expansion ablation, which showed that the membrane-focused augmentation used in this case study had a modest overall effect: it promoted selected membrane-associated candidates but did not globally dominate the final prioritisation ranks. Users who wish to apply stronger membrane-focused prioritisation can increase the membrane-edge expansion parameter in their own analyses. These analyses are provided in Additional File 3, Supplementary Tables S3–S7."

3. Empirical Justification of Borda Weights and Sensitivity Analysis
The Borda aggregation uses a "default" weighting scheme: 0.25 for MHC/DepMap and 0.10 for each network metric. While this system's flexibility is a strength, the manuscript would benefit from a more detailed rationale for these specific values.
* Sensitivity Analysis: The authors should perform a sensitivity analysis to demonstrate how the final ranking changes with different weighting scenarios (e.g., weighting MHC binding at 1.0 versus the current multi-layered approach).
* Addressing Feature Redundancy: Figure 6A shows a high correlation between several network metrics. Please discuss whether using five separate centrality measures leads to an inadvertent "overweighting" of the network component.

We thank the reviewer for this important point. We agree that the default Borda weights should be empirically justified and that correlated network metrics could, in principle, overweight the network component. To address this, we performed a sensitivity analysis using the features included in the Borda ranking: MHC-IC50, DepMap survivability score, and the five network metrics (Net_Betweenness, Net_Degree, Net_Impact, Net_Strength, and Net_WCI). HLA expression was not included in this sensitivity analysis because it was not part of the Borda score. We recomputed rankings under multiple alternative weighting scenarios and compared each to the default ranking across the 30 TCGA-SKCM samples. The scenarios included MHC-IC50-only, DepMap-only, network-only, no-network, no-MHC-IC50, no-DepMap, a network-weight sweep from 0% to 100% total network contribution, and leave-one-network-metric-out analyses. Ranking stability was assessed using Spearman correlation across the full ranked candidate list, as well as top-10 and top-20 overlap with the default ranking.
The sensitivity analysis showed that the default ranking was not simply recapitulating any single input feature. The MHC-IC50-only ranking differed substantially from the default ranking, with median Spearman correlation of 0.45 and median top-10 overlap of 0.40. DepMap-only ranking also differed from the default, with median Spearman correlation of 0.55 and median top-10 overlap of 0.45. Removing the full network component also altered the ranking, with median Spearman correlation of 0.66 and median top-10 overlap of 0.50. These results indicate that MHC binding, DepMap survivability, and network context each contribute to the final prioritization rather than one feature alone determining the ranking. At the same time, the ranking was not highly fragile to moderate weight perturbations. When the total network contribution was reduced to 25%, the median Spearman correlation with the default ranking was 0.88 and the median top-10 overlap was 0.70. When the network contribution was increased to 75%, the median Spearman correlation was 0.91 and the median top-10 overlap was again 0.70. Thus, moderate changes in the relative weight of the network layer changed the ranking, as expected, but did not completely reshuffle the highest-priority candidates.
To address feature redundancy, we also quantified pairwise correlations among the Borda features. As expected, several network metrics were correlated, particularly Net_Impact and Net_Strength (median Spearman $\rho = 0.99$), Net_Degree and Net_Strength ($\rho = 0.92$), and Net_Degree and Net_Impact ($\rho =$

0.90). However, the network metrics were not perfectly interchangeable: correlations involving Net_WCI and Net_Betweenness were lower, and leave-one-network-metric-out analysis showed small but measurable effects on the top-ranked set. Removing each network metric one at a time produced high full-rank similarity to the default ranking (median Spearman ρ = 0.98–1.00), but the median top-10 overlap ranged from 0.90 to 1.00 and the median top-20 overlap was approximately 0.95. This indicates that individual network metrics have overlapping information, but are not completely redundant.
Overall, these analyses support the interpretation that the default Borda weights provide a balanced, user-adjustable prioritization scheme. The ranking is sensitive to the inclusion of major biological information layers, particularly when MHC-IC50 or when the full network block is removed, but it is not dominated by one individual centrality metric. We have added the full Borda-weight sensitivity analysis to Additional File 3, including the alternative weighting scenarios, leave-one-network-metric-out analysis, and the corresponding summary plots shown in Supplementary Figure S5.

Supplementary Figure S5. Sensitivity of final Borda ranking to alternative feature-weighting scenarios. (A) Leave-one-network-metric-out full-rank similarity with the default Borda ranking. Bars show the median Spearman correlation across the 30 TCGA-SKCM samples after removing one network metric at a time. (B) Leave-one-network-metric-out overlap with the default ranking for the top 10 and top 20 candidates. Bars show the median overlap across samples. (C) Feature- and block-level sensitivity analysis showing median Spearman correlation between the default Borda ranking and alternative ranking scenarios, including MHC-IC50-only, DepMap-only, network-only, no-network, no-MHC-IC50, no-DepMap, and leave-one-network-metric-out scenarios. Blue bars indicate feature/block-level sensitivity analyses, while orange bars indicate leave-one-network-metric-out analyses. Error bars indicate variability across samples. (D) Distribution of top-10 overlap with the default ranking under selected alternative weighting scenarios. (E) Effect of changing the total network weight from 0% to 100% on similarity to the default ranking, measured by Spearman correlation and top-10 overlap. Overall, the analyses show that the default ranking is not reproduced by any single feature class, is affected by removal of major information layers, and remains relatively stable under moderate changes in total network weighting. Leave-one-network-metric-out analyses indicate that no individual centrality metric alone dominates the final ranking.

4. Impact of Non-PASS Variant Retention and Single-Caller Somatic Variant Calling
The authors deliberately retained non-PASS Mutect2 variants to maximize candidate epitope diversity, and rely on a single tool for both somatic SNV/indel detection (Mutect2) and fusion calling (STAR-Fusion). While both choices are disclosed and the tools themselves are industry standards, their combined effect is a candidate epitope pool of uncertain precision that directly affects all downstream prioritization results.
The impact of non-PASS variant retention is never quantified. It is unclear what fraction of top-ranked epitopes per patient derive from non-PASS variants, and whether the reported driver gene enrichment in the top 10 (Figure 6B) holds when the analysis is restricted to PASS-only variants. Furthermore, consensus somatic calling — for example adding Strelka2 for SNVs or Arriba for fusions — is known to improve precision and would reduce dependence on a single caller's filter thresholds. If the authors prefer to maintain a lightweight single-caller approach for practical reasons, a brief empirical justification should be provided, such as showing that the prioritization results are not materially sensitive to the non-PASS variant inclusion.
* Recommendation: The authors should quantify the contribution of non-PASS variants to the top-ranked epitope lists and demonstrate that key findings — particularly driver gene enrichment — are robust to PASS-only filtering. If consensus calling is not feasible within the scope of this work, a clear justification for the single-caller design should be included in the Methods.
To quantify the impact of retaining non-PASS Mutect2 variants, we annotated the somatic pVACseq candidates and final prioritised epitope tables with the FILTER status of the originating VCF record. PASS variants were defined as VCF FILTER equal to PASS, while all other FILTER values were treated as non-PASS. Fusion and splicing candidates were reported separately because Mutect2 FILTER status is not applicable to these classes.
This analysis showed that non-PASS Mutect2 calls contributed minimally to the final candidate set. In the final prioritised tables, 1,737/2,288 candidates were somatic, while 551/2,288 were fusion or splicing candidates for which Mutect2 FILTER status was not applicable. Among the somatic candidates, 1,714/1,737 were PASS and 23/1,737 were non-PASS, corresponding to 98.68% and 1.32% of somatic

final epitopes, respectively. Non-PASS somatic candidates represented only 1.01% of all final prioritised epitopes.
We then assessed whether non-PASS variants contributed to the highest-ranked epitopes. Across the top 10 ranked candidates per sample, only one somatic epitope derived from a non-PASS variant, corresponding to 0.34% of all top-10 candidates and 0.54% of somatic top-10 candidates. Thus, non-PASS Mutect2 calls made a negligible contribution to the highest-priority epitope set.
We also repeated the prioritisation using PASS-only somatic pVACseq inputs while retaining the same fusion, splicing, network, and annotation layers. Driver-gene representation among top-10 candidates was highly similar between the complete analysis and the PASS-only sensitivity analysis: 34/296 top-10 candidates in the complete analysis versus 33/296 in the PASS-only analysis. Thus, the driver-gene result highlighted in Figure 6B was not materially dependent on inclusion of non-PASS Mutect2 calls, but rather marginally improved once non-pass variants were removed.
Moreover, we agree that consensus calling can improve the specificity of somatic variant detection, and we have clarified this point as a limitation and possible future extension of the workflow. In the present study, Mutect2 and STAR-Fusion were selected to maintain a lightweight and reproducible implementation based on established tools for somatic SNV/indel and fusion detection, respectively [Mutect2 reference; STAR-Fusion reference]. This design choice reflects the scope of the present work, which is to evaluate EGG as an integrated neoantigen prediction and prioritisation framework, rather than to benchmark alternative variant- or fusion-calling strategies.
Importantly, our sensitivity analysis showed that the main prioritisation results were not materially dependent on retaining non-PASS Mutect2 calls. Non-PASS somatic variants contributed only 23/1,737 somatic final candidates, corresponding to 1.32% of somatic final epitopes and 1.01% of all final prioritised epitopes.
We have incorporated these clarifications into the Methods section. Specifically, we added the following paragraphs describing the PASS/non-PASS sensitivity analysis and the rationale for using Mutect2 and STAR-Fusion as pragmatic single-caller defaults:

"Somatic SNV and indel calls were generated with Mutect2 followed by Mutect2 post-filtering. For the proof-of-concept analysis presented here, both PASS and non-PASS Mutect2 records were retained as pVACseq input to preserve a broad candidate mutation pool before downstream prioritisation. To assess the impact of this choice, we annotated final somatic epitope candidates with the VCF FILTER status of the originating variant and repeated the prioritisation using PASS-only somatic pVACseq inputs. This sensitivity analysis showed that non-PASS calls contributed minimally to the final prioritised candidates and did not materially affect the prioritised gene set. PASS-only filtering can be applied before pVACseq when a higher-specificity input set is desired. Full details of the PASS-only sensitivity analysis are provided in Additional File 3."
and this paragraph also was added to the Method section:
"Mutect2 and STAR-Fusion were selected as pragmatic single-caller defaults for somatic SNV/indel and fusion detection, respectively. This choice was intended to keep the case-study workflow lightweight, reproducible, and computationally tractable, while relying on established tools with broad use in cancer genomics. Mutect2 is used in the GATK somatic short-variant discovery workflow for SNV and indel detection, and is widely regarded as an established, reliable somatic variant caller in cancer genomics [18,55]. STAR-Fusion is used in the NCI Genomic Data Commons RNA-seq harmonization workflow for transcript-fusion detection and has been reported among the most accurate and fastest fusion callers in benchmark analyses of cancer transcriptomes[56]. We acknowledge that consensus calling strategies can further improve input precision and reduce dependence on caller-specific thresholds; however, benchmarking alternative caller combinations was outside the scope of the present study, which focuses on evaluating EGG as an integrated neoantigen prediction and prioritisation framework."

5. Impact of Membrane Enrichment Step
The addition of 5% membrane-associated gene edges to the pFINs is motivated by references showing membrane-localized neoantigen enrichment in immunotherapy responders. However, the actual impact of this step on epitope prioritization is never evaluated.
* Empirical Assessment: How many of the top-ranked epitopes per patient derive from membrane genes? Does the membrane expansion materially change the rankings compared to those of PPI-filtered networks alone? Although this step is presented as a key design choice, it requires empirical evaluation.
We quantified the representation of membrane-associated source genes among the final prioritized

epitopes using the same membrane-gene set used for pFIN expansion. Across the 30 patients, membrane-associated genes accounted for a median of 22.3% of all candidate epitopes. Among the top-ranked epitopes, the median membrane-associated fraction was 20.0% for the top 10 epitopes, 20.0% for the top 20 epitopes, 22.0% for the top 50 epitopes, and 23.8% for the top 100 epitopes. Thus, membrane-associated epitopes were present among prioritized candidates but were not strongly represented compared to the overall pool of candidates. This suggests that the membrane-edge expansion does not by itself dominate final epitope prioritization.

Cutoff	Background membrane	Mean membrane count, original ranking	Mean membrane count, original ranking without membrane enhancement	Top membrane % median
Top 10 epitopes	22.3%	2	2	20.0%
Top 20 epitopes	22.3%	4	4	20.0%
Top 50 epitopes	22.3%	10	10	22.0%

To directly evaluate whether the membrane-edge expansion materially changed prioritisation, we additionally performed a membrane-expansion ablation. In this analysis, we recomputed the final weighted Borda ranking using the same feature weights and directions as in the original workflow, but replaced the PPI + membrane-expanded pFIN metrics with metrics calculated from the PPI-filtered networks before membrane-edge addition. All features, but not network metrics, including IC50, DepMap survivability, IntOGen annotations, and WCI, were kept unchanged. As a validation step, we first recomputed the original weighted Borda ranking from the final feature columns; this reproduced the final ranks closely, with median Spearman ρ = 0.9998 and median Kendall τ = 0.993 across patients.

Removing the membrane-edge expansion had only a modest effect on the final prioritisation. The final PPI + membrane ranking and the PPI-only ablation ranking had a median Spearman ρ of 0.979, minimum Spearman ρ of 0.885, and median Kendall τ of 0.901. The top-ranked candidates were also largely preserved: the median top-10 overlap was 9/10 epitopes and the median top-20 overlap was 18/20 epitopes. The number of membrane-derived epitopes in the top-ranked sets was essentially unchanged, with a median of 2 membrane-derived epitopes in the top 10 and 4 in the top 20 in both the original and PPI-only rankings as seen in the reported table above.

Although the global ranking impact was limited, the membrane expansion did have the intended local effect in selected cases. Across 2,288 candidate epitopes detected in our cohort, 523 were derived from membrane-associated genes. Among these, 236 membrane-derived epitopes across 25 patients showed some upward rank shift when the membrane expansion was included. More substantially, 78 membrane-derived epitopes across 17 patients improved by at least 5 rank positions, and 26 epitopes across 9 patients improved by at least 10 positions. In 8 patients, the membrane expansion moved a membrane-derived epitope into the top 10, and in 12 patients it moved at least one membrane-derived epitope into the top 20. Thus, the membrane-edge expansion acts as a limited, targeted network augmentation rather than a dominant driver of the final ranking.

We have added the membrane-edge expansion analysis to Additional File 3, including both the representation of membrane-associated epitopes among top-ranked candidates and the PPI-only ablation analysis. We also clarified in the main text that the membrane expansion used in this case study acts as a modest augmentation step: it can promote selected membrane-associated candidates, while leaving the overall prioritisation largely stable.

In the main text, we added the following:

"We further assessed the robustness and contribution of the pFIN network layer through several sensitivity analyses. Specifically, we evaluated pFIN stability using leave-one-background-sample-out jackknife analyses, top-edge consistency testing, and two GDC TCGA-SKCM benchmarks using larger n = 103 and n = 469 background cohorts. We also performed a membrane-edge expansion ablation, which showed that the membrane-focused augmentation used in this case study had a modest overall effect: it promoted selected membrane-associated candidates but did not globally dominate the final prioritisation ranks. Users who wish to apply stronger membrane-focused prioritisation can increase the membrane-edge expansion parameter in their own analyses. These analyses are provided in Additional File 3, Supplementary Tables S3–S7."

Minor Revisions

6. Quantitative Tool Comparison

Table 1 provides a helpful feature matrix, but a quantitative comparison would better substantiate the

claim that EGG represents a concrete advance over existing tools.
* Direct Comparison: A direct comparison — such as the overlap between EGG's top-ranked epitopes and those of other tools on the same 30 patients — would concretely demonstrate what the additional prioritization layer contributes.
We thank the reviewer for this helpful suggestion. We agree that quantitative comparisons across tools are valuable when the methods address the same prioritization task and use comparable scoring criteria. However, a direct top-ranked overlap analysis would be difficult to interpret here, because EGG introduces several conceptually different prioritization layers. Most existing workflows rank candidates mainly using epitope- or sequence-derived features, such as peptide–MHC binding affinity, binding percentile, expression support, agretopicity, presentation, or immunogenicity-related scores. EGG can include these standard epitope-level features, but its main contribution is to add source-gene biological context to the ranking.
In particular, EGG incorporates DepMap-derived gene essentiality and patient-specific co-expression network topology, allowing candidate epitopes to be prioritized partly according to the functional importance of their source genes in the individual tumor context. Thus, differences in top-ranked candidates would be expected and would not necessarily reflect disagreement in neoantigen prediction, but rather the contribution of a non-sequence-based, gene-context-aware prioritization strategy.
To clarify this distinction, we have modified table 1 in the manuscript comparing EGG with representative neoantigen prediction and prioritization tools. The table summarizes workflow scope, alteration classes considered, inputs, MHC coverage, HLA typing, epitope-level prioritization features, source-gene essentiality/context, patient-specific network context, membrane/surface enhancement, additional source-gene annotations, and the type of ranking or prioritization output provided by each tool.

7. Computational Resource Requirements
No benchmarking of runtime or computational requirements is provided.
* Recommendation: Given that EGG processes three data modalities and constructs single-sample networks, prospective users evaluating EGG for clinical or large-scale research use need runtime and memory information.
We agree that runtime and memory information is useful for prospective users, particularly because EGG combines multiple data modalities and constructs patient-specific functional interaction networks. To address this, we added a new computational resource benchmark for the two EGG-specific downstream modules: network generation and epitope prioritisation. We focused on these modules because the earlier steps of the workflow rely largely on established third-party tools, including commonly used mutation-calling and neoantigen-prediction software, for which resource requirements are already documented by the respective developers. In addition, the runtime and memory requirements of these upstream steps are strongly influenced by sample-specific factors such as sequencing depth, library size, and mutation burden, making them less directly informative as a benchmark of EGG-specific computational requirements. For the network-generation benchmark, we used the same 30 TCGA melanoma samples analysed in the manuscript.
The benchmark was run throug the script included in the sample_networks_generation.smk file. Such a script included function for RNA count normalisation, LIONESS single-sample network reconstruction, PPI-based network filtering, membrane-edge enrichment, network centrality metrics, largest-component impact, full-network WCI/strength calculation, and QC plot generation. The benchmark was run with Snakemake using single-threaded rules, with BLAS/OpenMP threading fixed to one thread per job. Because membrane-edge enrichment was the main memory-intensive step, this rule was assigned a Snakemake thread reservation to limit concurrent execution while allowing lighter rules to run in parallel.
We now report per-rule runtime and peak memory usage in Supplementary Table S1 in Additional file 1. In brief, the most memory-intensive step was membrane-edge enrichment, with a median peak resident memory of 75.36 GB per sample and a median runtime of 24.3 min per sample. The longest-running per-sample step was PPI-only filtering, with a median runtime of 2.30 h per sample and a median peak resident memory of 11.03 GB. LIONESS reconstruction was run once for the 30-sample cohort and required 4.09 h and 36.11 GB peak resident memory. These results identify membrane enrichment and PPI filtering as the main computational bottlenecks and provide practical guidance for users to adjust Snakemake concurrency according to available memory.

Rule / step	n	Median runtime	Max runtime	Median peak RAM	Max peak RAM
RNA normalisation	1	19.3 s	19.3 s	0.73 GB	0.73 GB
LIONESS reconstruction	1	4.09 h	4.09 h	36.11 GB	36.11 GB
PPI-only filtering	30	2.30 h	2.47 h	11.03 GB	11.04 GB

Membrane-edge enrichment 30 24.3 min 26.6 min 75.36 GB 75.37 GB
Degree/strength/betweenness metrics 30 3.49 min 3.82 min 0.16 GB 0.17 GB
Largest-component impact 30 51.0 min 56.6 min 0.29 GB 0.33 GB
Full-network WCI 30 55.8 s 1.92 min 11.11 GB 14.55 GB

Rule / step n Median runtime Max runtime Median peak RAM Max peak RAM
Merge epitopes / IC50 harmonisation 30 16.0 s 16.1 s 0.08 GB 0.11 GB
DepMap/IntOGen annotation 30 5.3 s 6.8 s NA NA
Add network metrics 30 1.85 min 2.95 min 0.99 GB 1.26 GB
Compute Borda rank 30 5.2 s 8.5 s NA NA
GO/Hallmark annotation 30 36.9 s 60.5 s 0.95 GB 1.05 GB
Build interactive HTML 30 6.6 s 9.9 s NA NA

We have also clarified this in the manuscript text by specifying that network-related calculations are carried out by the network generation module, while final candidate ranking is performed by the epitope prioritisation module. We further added that Additional file 1 now includes implementation details for pipeline execution, retrieval of required references and tools, execution of individual modules, an overview of the main third-party tools integrated in EGG, and a computational resource benchmark of the main EGG-specific components. The benchmark focuses on patient-specific network generation and epitope prioritisation rather than upstream third-party tools, whose resource requirements depend strongly on sequencing depth, library size, and mutation burden.
"The network-related calculations and network generation are carried out by the network generation module, while final candidate ranking is performed by the epitope prioritisation module. Additional information for pipeline execution, including implementation details such as the Snakemake workflow [39], retrieval of required references and tools, and execution of individual modules, is provided in Additional file 1 (Supplementary Material). Additional file 1 also includes an overview of the main third-party tools integrated in EGG and a computational resource benchmark of the main EGG-specific components, focusing on patient-specific network generation and epitope prioritisation rather than upstream third-party tools whose resource requirements depend strongly on sequencing depth, library size, and mutation burden."
8. Statistical Context for Recurrent Epitopes
The recurrence of KANSL1 (12/30) and NRAS (7/30) is interesting, but no statistical comparison to background recurrence rates is provided.
* Clarification: Are these frequencies significantly higher than expected, given the baseline mutation rates of these genes in TCGA-SKCM? Without a null model, it is unclear whether the recurrence reflects a specific biological signal or simply the known mutational frequency in melanoma.
We thank the reviewer for this important clarification. We agree that recurrence across the selected 30-sample cohort should not be interpreted as evidence of statistically unexpected enrichment without an appropriate background model. We therefore revised the text to present recurrent genes and exact epitope sequences as descriptive and hypothesis-generating observations.
For NRAS, we performed a simple background comparison using the TCGA-SKCM NRAS mutation frequency reported in the TCGA melanoma study. NRAS somatic mutations were reported in 28% of TCGA-SKCM tumors, while NRAS-derived epitope candidates were observed in 7/30 selected samples in our analysis. A one-sided binomial test did not support enrichment above this background rate ($p = 0.776$). Thus, we now interpret NRAS recurrence as consistent with the known melanoma mutational landscape rather than as evidence of unexpectedly high recurrence.
For KANSL1, the recurrent fusion-derived candidates in our output were primarily linked to the KANSL1-ARL17/KANSARL fusion locus, which has been reported previously in cancer transcriptomic studies. We have therefore revised the manuscript to identify this as a known fusion locus rather than presenting it as an uncharacterized melanoma-specific recurrence signal.
We report the modified text below:
"These KANSL1-associated events were primarily linked to the KANSL1–ARL17/KANSARL fusion locus, which has been described previously in cancer transcriptomic studies [84]. A comparable pattern emerges for genomic mutations: exact mutation-derived peptides remain patient-specific, but the gene NRAS shows the highest recurrence (7 samples; figure 6C), with the epitope LDILDTAGREEYSAMRDQYMRTGEGFLCV shared across 3 patients (figure 6D). Since NRAS is a known recurrent melanoma driver, we compared this observation with the reported TCGA-SKCM NRAS mutation frequency of 28%. The observed frequency of

NRAS-derived epitope candidates in the selected cohort was not higher than expected relative to this background rate (one-sided binomial test, p-value = 0.776), indicating that NRAS recurrence is consistent with the known melanoma mutational landscape rather than evidence of unexpected overrepresentation [85].”

9. Exclusion of HLA Expression from Ranking
Because HLA expression levels are important for neoantigen presentation probability and expression data is already processed within EGG, the rationale for excluding it from the default ranking should be explicitly stated.
We thank the reviewer for this helpful comment. We have expanded the Results paragraph discussing HLA expression, which noted that it was not included as a ranking criterion in the analysis presented here, to further explain the rationale for this choice. Specifically, we now clarify that this reflects EGG’s emphasis on complementing sequence-based epitope prioritisation with gene-level functional information, including database-derived essentiality and patient-specific network topology. We have also clarified that HLA expression is retained in the final output tables and can therefore be considered during downstream interpretation or incorporated by the user as an additional Borda score ranking criterion, together with other optional pVACtools algorithm outputs.
This clarification has been incorporated into the manuscript as follows:

“HLA expression was not included as a ranking criterion in the analysis presented here, explaining its limited correlation with the Borda score. This reflects EGG’s emphasis on complementing sequence-based epitope prioritisation with gene-level functional information, including database-derived essentiality and patient-specific network topology. Nevertheless, HLA expression is retained in the final output tables and can be considered during downstream interpretation or included by the user as an additional ranking criterion in the borda score, together with other optional pVACtools algorithm outputs.”

Overall, EGG represents a thoughtful and timely contribution to the neoantigen discovery landscape. The integration of patient-specific network biology with established prediction tools is a conceptually strong advance, and the authors have built a flexible, well-documented pipeline that the community will find useful. The concerns raised in this review are intended constructively and primarily center on strengthening the empirical foundation of the prioritization framework, clarifying several methodological choices, and ensuring that the scope of the conclusions is proportionate to the current evidence. We are confident that addressing these points will substantially strengthen the manuscript and its impact, and we encourage the authors to consider experimental validation as a particularly powerful path forward. We look forward to reviewing a revised version.

Editorial Manager®

Cookie Preference Center

We use cookies which are necessary to make our site work. We may also use additional cookies to analyze, improve and personalize our content and your digital experience. For more information, see our Cookie Policy.

You may choose not to allow some types of cookies. However, blocking some types may impact your experience of our site and the services we are able to offer. See the different category headings below to find out more or change your settings.

Allow all

Manage Consent Preferences

Strictly Necessary Cookies

Always active

These cookies are necessary for the website to function and cannot be switched off in our systems. They are usually only set in response to actions made by you which amount to a request for services, such as setting your privacy preferences, logging in or filling in forms. You can set your browser to block or alert you about these cookies, but some parts of the site will not then work.

Functional Cookies

These cookies enable the website to provide enhanced functionality and personalisation. They may be set by us or by third party providers whose services we have added to our pages. If you do not allow these cookies then some or all of these services may not function properly.

Performance Cookies

These cookies allow us to count visits and traffic sources so we can measure and improve the performance of our site. They help us to know which pages are the most and least popular and see how visitors move around the site.

Targeting Cookies

These cookies may be set through our site by our advertising partners. They may be used by those companies to build a profile of your interests and show you relevant adverts on other sites. If you do not allow these cookies, you will experience less targeted advertising.

Cookie List

Clear

Apply Cancel

Consent Leg.Interest

Confirm my choices

Powe
