# Supplementary material for: Epitope Generation Gateway: A biological context-dependent approach to cancer vaccine development
Source: Gigascience. 2026 Oct 1;15:giag095. doi: 10.1093/gigascience/giag095 (PMC13679409; doi:10.1093/gigascience/giag095)
Supplement: Supplementary file 2 [file giag095_authors_response_to_reviewer_comments_revision_1.pdf]

Author's Response To Reviewer Comments

Dear Editor and Reviewers,
We sincerely thank the Editor and both reviewers for their continued consideration of our manuscript. We are grateful to Reviewer 2 for their positive assessment and to Reviewer 1 for the remaining constructive comments, which helped us further clarify the scope of the study and align our claims more closely with the evidence presented.

As requested by the Editor, we have registered EGG in bio.tools (biotoolsID: epitope_generation_gateway; https://bio.tools/epitope_generation_gateway) and SciCrunch (RRID: SCR_028792). We have also archived the computational workflow in WorkflowHub (DOI: 10.48546/workflowhub.workflow.2165.2; https://doi.org/10.48546/workflowhub.workflow.2165.2). These identifiers and their corresponding links have been added to the revised manuscript.

Below, we provide a detailed point-by-point response to Reviewer 1's comments. Revised versions of the manuscript and supplementary information, with all modifications indicated using track changes, have been included with the resubmission. The principal revisions are also summarised in our responses below and can be reviewed in the accompanying "Manuscript with track changes" and "Supplementary Information with track changes" PDF files.

A file was also added to the submission named Answers to reviewer comments 2.docx which consists of these answers but in a word document so that it might be more readable as it includes the figures.
Reviewer reports:
Reviewer #1: I thank the authors for a thorough and largely responsive revision. The pipeline is well engineered and the rebuttal resolves most of my methodological concerns. A few points remain, centered on the validation analysis and on scoping the claims to match the evidence.

1. I'm still having trouble reconciling that presentation and immunogenicity are not durability.
The paper's central thesis is that ranking by source-gene essentiality and network centrality yields targets the tumor is less able to silence under immune pressure.
However, I think matching against IEDB/CEDAR/TSNAdb, at best, tells us a candidate peptide is more likely to be presented or T-cell-reactive.
It says nothing about whether that candidate's source gene is harder to lose during tumor evolution.
So the entire durability claim (the reason the network layer exists) is untouched by this analysis.
I suggest authors frame the durability advantage as a hypothesis rather than something that has been validated.
We agree that presentation and immunogenicity are not equivalent to evolutionary durability. The IEDB/CEDAR/TSNAdb analysis provides support that EGG/Borda-ranked candidates can recover peptides with experimental evidence of presentation and/or immune recognition more effectively than an IC50-only ranking, but it does not demonstrate that the corresponding source gene are less likely to be lost during tumour evolution.
We have therefore revised the manuscript to downstate the immune escape resistance of the identified neoantigen as a hypothesis rather than as a validated outcome of the present study. Accordingly, we have revised the relevant sections of the manuscript to better distinguish the level of support.
Therefore, we added the following text to the result/discussion section of the manuscript:
"Although EGG is designed to prioritise epitopes derived from genes with greater functional and network relevance, the proposed evolutionary durability of such targets remains a hypothesis in the present study. The external epitope-database analysis supports the potential presentation and immunogenicity of selected candidates, but does not directly test whether their source genes are less likely to be lost or silenced under immune pressure."

2. I'm also wondering if the minimum 8 containment is a good threshold given peptides vary by length. Wouldn't a % match be a better threshold? I fear that some of the result is coming from peptides that are of a longer size and for which 8 amino acid match brings them somewhere very different. Could the

authors clarify if this is affected?

We thank the reviewer for raising this important point. The min-8 containment endpoint was intended as a sensitive core-overlap criterion rather than as evidence of full-length peptide identity. We used this approach because exact full-length peptide matching was extremely sparse. Nevertheless, we confirm that peptide length can affect the interpretation of a fixed 8-amino-acid containment threshold, and that a peptide length-normalised analysis provides an important sensitivity check.
To address this, we performed an additional coverage sensitivity analysis, now included in Additional file 3_Supplementary_Material. For each 8amino acids containment match between an EGG candidate peptide and an experimentally supported external reference peptide from CEDAR, IEDB, or TSNAdb v2.0, we calculated the fraction of the longer peptide covered by the matched sequence. We then repeated the analysis using the original min-8 containment endpoint and stricter criteria requiring min-8 containment plus ≥50%, ≥70%, or ≥80% coverage of the longer peptide.
Increasing the required longer-peptide coverage substantially reduced database-specific support. CEDAR retained 542, 189, 40, and 14 candidates; IEDB retained 545, 192, 41, and 14 candidates; and TSNAdb v2.0 retained 5, 1, 0, and 0 candidates across the min-8, ≥50%, ≥70%, and ≥80% thresholds, respectively (Additional file 3_Supplementary_Material, Supplementary Figure S7). We also repeated the ranking comparison using these progressively stricter coverage thresholds. The original relaxed min-8 endpoint produced the clearest recovery signal relative to IC50-only ranking at selective cutoffs, whereas this signal was attenuated under the ≥50% longer-peptide coverage threshold and became sparse at the ≥70% and ≥80% thresholds (Additional file 3_Supplementary_Material, Supplementary Figure S8). These results indicate that the min-8 endpoint is useful for detecting shared experimentally supported peptide cores, but is sensitive to peptide length-normalised stringency.

To help clarify we have added the following paragraph in the supplementary file 3:
"To assess whether the relaxed min-8 containment endpoint was influenced by peptide length, we performed an additional peptide length-normalised coverage sensitivity analysis. For each relaxed containment match between an EGG candidate peptide and an experimentally supported external reference peptide from CEDAR, IEDB, or TSNAdb v2.0, we calculated the fraction of the longer peptide covered by the matched sequence. We then repeated the external-evidence comparison using min-8 containment alone and min-8 containment combined with ≥50%, ≥70%, and ≥80% longer-peptide coverage thresholds.
Increasing the required longer-peptide coverage substantially reduced database-specific support. CEDAR retained 542, 189, 40, and 14 candidates; IEDB retained 545, 192, 41, and 14 candidates; and TSNAdb v2.0 retained 5, 1, 0, and 0 candidates across the min-8, ≥50%, ≥70%, and ≥80% thresholds, respectively (Supplementary Figure S7).
We then repeated the ranking comparison using these progressively stricter coverage thresholds. The original relaxed min-8 endpoint produced the clearest recovery signal relative to IC50-only ranking at selective cutoffs, whereas this signal was attenuated under the ≥50% longer-peptide coverage threshold and became sparse at the ≥70% and ≥80% thresholds (Supplementary Figure S8). These results indicate that relaxed min-8 containment is useful for detecting shared experimentally supported peptide cores, but that it is sensitive to peptide length-normalised stringency and should not be interpreted as full-length peptide validation.

Supplementary Figure S7. Effect of peptide length-normalised coverage thresholds on database-specific external evidence support. The number of EGG candidates with experimentally supported external evidence is shown for each matching criterion, stratified by evidence database. Candidate support was assessed using the original relaxed min-8 containment endpoint and stricter criteria requiring the matched segment to cover at least 50%, 70%, or 80% of the longer peptide. Counts are shown separately for CEDAR, IEDB, and TSNAdb v2.0. Increasing the coverage threshold substantially reduced the number of supported candidates, indicating that relaxed min-8 containment should be interpreted as sensitive core-level support rather than full-length peptide validation.

Supplementary Figure S8. Effect of peptide length-normalised coverage thresholds on ranking-based recovery of external evidence. Median difference in hit rate between each ranking method and IC50-only

ranking is shown across top-N and top-percentile cutoffs within each patient. The analysis used experimentally supported peptide matches pooled across CEDAR, IEDB, and TSNAdb v2.0. The original relaxed min-8 containment endpoint produced the clearest recovery signal relative to IC50-only ranking at selective cutoffs. This signal was attenuated under the ≥50% longer-peptide coverage threshold and became sparse at the ≥70% and ≥80% thresholds, where median differences were zero. These results indicate that the relaxed min-8 endpoint captures shared experimentally supported peptide cores, however stricter full-length-like coverage thresholds leave too few supported candidates for robust ranking comparison."

3. Is assuming purity =1 a good measure? I wonder if this inflates the VAF interpretation.
Ex: melanomas are 0.3-0.7. Have the authors tested with other purity values?
We agree that tumour purity is an important consideration when interpreting tumour DNA variant allele fraction. We recognise that tumour samples are unlikely to consist exclusively of tumour cells and may also contain adjacent normal tissue and infiltrating immune cells. Nevertheless, assuming purity = 1 is conservative in this context and avoids inflating the inferred clonality. Under a simplified diploid heterozygous model, cancer cell fraction is approximately proportional to 2 × variant allele fraction divided by tumour purity. Therefore, for a fixed observed variant allele fraction, lowering the assumed tumour purity from 1.0 to values such as 0.7, 0.5, or 0.3 would increase, rather than decrease, the inferred cancer cell fraction.
In our analysis, all matched somatic final candidates, including all somatic top-10 candidates, had tumour DNA variant allele fraction ≥ 0.25. Under the conservative purity = 1 assumption, this corresponds approximately to cancer cell fraction ≥ 0.5. Using lower melanoma-relevant purity values would risk yielding variants that are less compatible with clonal or near-clonal status. Thus, additional testing across lower purity values would not change the qualitative conclusion that we did not identify a low-variant-allele-fraction or clearly subclonal subset driving the final prioritisation.
Finally, the relevant purity- and variant-allele-fraction-related parameters are user-configurable in the pVACtools-based component of the EGG workflow, allowing users to apply different thresholds if needed.
We also added this paragraph in the method section to clarify what tumor purity value was used and that in can be modified by the user if needed.
"For tumour-purity-dependent tiering, we retained the pVACseq default tumour purity value of 1.0; this parameter is user-configurable and can be adjusted when sample-specific tumour purity estimates are available. In this case study, retaining tumour purity = 1.0 provided a conservative variant allele frequency based parameter for identifying tumour-relevant somatic candidates."

4. Maybe a very minor point: The workflow is described as "lightweight," but the new benchmark reports ~75 GB peak memory for the membrane-enrichment step; the framing should be tempered.

We agree with the reviewer. Our use of "lightweight" was intended to refer to reduced workflow complexity and runtime for the case-study single-caller setup and downstream epitope-prioritisation steps, not to imply that all components have low memory requirements. The benchmark shows that some patient-specific network-generation steps, particularly membrane-edge enrichment, can require substantial RAM. To avoid ambiguity, we have replaced "lightweight" with "streamlined" in the main text and clarified in the supplementary benchmark that the epitope-prioritisation steps are comparatively lightweight only after candidate neoantigen tables and network metrics have been generated. We also now explicitly note that network generation, especially membrane-edge enrichment, represents the main memory-intensive component of the workflow.

Revised supplementary paragraph:
"The epitope prioritisation benchmark was run across the same 30 samples and included candidate merging and IC50 harmonisation, DepMap/IntOGen annotation, addition of network metrics, Borda rank calculation, GO/Hallmark annotation, and generation of interactive HTML outputs. These downstream prioritisation steps were comparatively lightweight once candidate neoantigen tables and network metrics had been generated. Merging epitopes and harmonising IC50 values required a median of 16.0 s per sample, DepMap/IntOGen annotation required 5.3 s, addition of network metrics required 1.85 min, Borda rank calculation required 5.2 s, GO/Hallmark annotation required 36.9 s, and interactive HTML generation required 6.6 s. Peak memory was below approximately 1.3 GB for steps where reliable memory estimates were available, except that some very short rules did not produce stable peak-memory estimates and are

therefore reported as not available.
Overall, this benchmark shows that EGG-specific epitope prioritisation is computationally modest after upstream candidate generation and patient-specific network metrics are available. In contrast, the main computational burden is associated with patient-specific network generation, particularly LIONESS reconstruction, PPI filtering, and membrane-edge enrichment; the latter reached approximately 75 GB peak RAM in this benchmark. These results provide practical guidance for adjusting Snakemake concurrency and memory allocation according to available compute resources.”

A few remaining items I noticed in the revised text:
* Figure 3: the body states "up to 2240 epitopes in a single sample" while the Figure 3 caption states "up to 224 epitopes." Please reconcile — this looks like a residual error from the recount.
* A sentence in the Patient2-Specific Networks Methods ("Gene level raw read count matrices previously produced…") is duplicated.
* "Supplementray" is misspelled in several places, including the Additional file 3 description.

We thank the reviewer for identifying these remaining editorial issues. We have revised the text accordingly by reconciling the Figure 3 value between the main text and figure caption, removing the duplicated sentence in the Patient-Specific Networks Methods section, and correcting the misspelling of “Supplementary” throughout the manuscript and supplementary-file descriptions, including the description of Additional file 3.

Final point:
The Abstract and title still state that EGG prioritizes "durable cancer vaccine targets" and reduces "the likelihood of immune escape." These are efficacy claims that the current validations do not establish and so given the appropriately scoped limitation now in the Discussion, please bring the Abstract (and ideally the title framing) into line so the claims match the evidence.

We agree with the reviewer and have revised the Abstract to avoid presenting evolutionary durability or reduced immune escape as validated outcomes of the current study. Specifically, we replaced sentences stating that EGG prioritizes candidates “resilient to tumor immune escape,” “reduces the likelihood of immune escape,” and identifies “durable cancer vaccine targets” with more appropriately scoped wording describing EGG as a framework that integrates peptide-level prediction with gene-level and network-context features relevant to tumour fitness and immune escape.

We modified the text of the abstract as follows:

“Abstract
Background
Personalized neoantigen discovery requires tools that not only identify putative epitopes but also prioritize candidates using biological features relevant to tumor fitness and immune escape. Here, we present Epitope Generation Gateway (EGG), a containerized pipeline that streamlines discovery and prioritization across variant types and human leukocyte antigen (HLA) contexts. EGG coordinates established components, Mutect2 for somatic single nucleotide variations (SNVs) and indels, STAR-Fusion for fusion events, ArcasHLA for HLA typing, and pVACtools for epitope sequence prediction, then augments peptide-level scores with biological context. For each patient, EGG builds co-expression and protein-protein interaction networks, uses centrality measures and CRISPR dependency data (DepMap) to flag genes important for tumor fitness, and annotates source genes with Gene Ontology terms, Hallmarks of Cancer processes, and IntOGen driver status.
Results
This multi-layer information supports ranking of candidates that are predicted to be presented and that arise from genes essential to tumor fitness or embedded in key pathways, providing a framework to nominate epitopes whose source genes may be less dispensable under immune pressure. As a case study, we applied EGG to 30 TCGA skin cutaneous melanoma (SKCM) patients, demonstrating end-to-end execution and producing prioritized epitopes that emphasize peptides and alterations in high-centrality, dependency-supported essential genes.

Conclusions
Overall, EGG links epitope prioritization to tumor systems biology and evolutionary rationale, prioritizing biologically contextualized cancer vaccine candidates from SNVs, indels, fusions, and splicing-derived epitopes while streamlining dataset analysis. EGG is available from GitHub at https://github.com/fhaive/epitope_generation_gateway, registered in bio.tools (biotoolsID: epitope_generation_gateway; https://bio.tools/epitope_generation_gateway) and SciCrunch (RRID: SCR_028792), and archived as a citable workflow in WorkflowHub (DOI: 10.48546/workflowhub.workflow.2165.2).
"

Editorial Manager®

Cookie Preference Center

We use cookies which are necessary to make our site work. We may also use additional cookies to analyze, improve and personalize our content and your digital experience. For more information, see our Cookie Policy.

You may choose not to allow some types of cookies. However, blocking some types may impact your experience of our site and the services we are able to offer. See the different category headings below to find out more or change your settings.

Allow all

Manage Consent Preferences

Strictly Necessary Cookies

Always active

These cookies are necessary for the website to function and cannot be switched off in our systems. They are usually only set in response to actions made by you which amount to a request for services, such as setting your privacy preferences, logging in or filling in forms. You can set your browser to block or alert you about these cookies, but some parts of the site will not then work.

Functional Cookies

These cookies enable the website to provide enhanced functionality and personalisation. They may be set by us or by third party providers whose services we have added to our pages. If you do not allow these cookies then some or all of these services may not function properly.

Performance Cookies

These cookies allow us to count visits and traffic sources so we can measure and improve the performance of our site. They help us to know which pages are the most and least popular and see how visitors move around the site.

Targeting Cookies

These cookies may be set through our site by our advertising partners. They may be used by those companies to build a profile of your interests and show you relevant adverts on other sites. If you do not allow these cookies, you will experience less targeted advertising.

Cookie List

Clear

Apply Cancel
Consent Leg.Interest

Confirm my choices

Power
