# Supplementary material for: Epitope Generation Gateway: A biological context-dependent approach to cancer vaccine development
Source: Gigascience. 2026 Oct 1;15:giag095. doi: 10.1093/gigascience/giag095 (PMC13679409; doi:10.1093/gigascience/giag095)
Supplement: Supplementary file 3 [file giag095_giga-d-26-00020_original_submission.pdf]

GigaScience

Epitope Generation Gateway (EGG):

A biological context-dependent approach to cancer vaccine development

--Manuscript Draft--

**Manuscript Number:**	GIGA-D-26-00020
**Full Title:**	Epitope Generation Gateway (EGG):<br><br>A biological context-dependent approach to cancer vaccine development
**Article Type:**	Research
**Funding Information:**	Jane ja Aatos Erkon Säätiö — Prof Seppo Ylä-Herttuala<br>Prof Vincenzo Cerullo<br>Prof Dario Greco
**Abstract:**	Abstract<br>Background<br>Personalized neoantigen discovery requires tools that not only identify putative epitopes but also prioritize candidates resilient to tumor immune escape. Here, we present Epitope Generation Gateway (EGG), a containerized pipeline that streamlines discovery and prioritization across variant types and human leukocyte antigen (HLA) contexts. EGG coordinates established components, Mutect2 for somatic single nucleotide variations (SNVs) and indels, STAR-Fusion for fusion events, ArcasHLA for HLA typing, and pVACtools for epitope sequence prediction, then augments peptide-level scores with biological context. For each patient, EGG builds co-expression and protein-protein interaction networks, uses centrality measures and CRISPR dependency data (DepMap) to flag genes important for tumor fitness, and annotates source genes with Gene Ontology terms, Hallmarks of Cancer processes, and IntOGen driver status.<br>Results<br>This multi-layer information supports ranking of candidates that are predicted to be presented and that arise from genes essential to tumor fitness or embedded in key pathways, thereby reducing the likelihood of immune escape by mutations caused by treatment pressure. As a case study, we applied EGG to 30 TCGA skin cutaneous melanoma (SKCM) patients, demonstrating end-to-end execution and producing prioritized epitopes that emphasize peptides and alterations in high-centrality, dependency-supported essential genes.<br>Conclusions<br>Overall, EGG links epitope prioritization to tumor systems biology and evolutionary dynamics, prioritizing durable cancer vaccine targets from SNVs, indels, fusions, and splicing derived epitopes while streamlining dataset analysis. EGG is available to download at https://github.com/fhaive/epitope_generation_gateway.
**Corresponding Author:**	Dario Greco<br>Tampereen Yliopisto<br>Tampere, FINLAND
**Corresponding Author Secondary Information:**	
**Corresponding Author's Institution:**	Tampereen Yliopisto
**Corresponding Author's Secondary Institution:**	
**First Author:**	Luca Mannino
**First Author Secondary Information:**	
**Order of Authors:**	Luca Mannino
	Michele Fratello
	Jacopo Chiaro
	Federica D'Alessio

Powered by Editorial Manager® and ProduXion Manager® from Aries Systems Corporation

	Emanuele Di Lieto
	Minna Niittykoski
	Angela Serra
	Seppo Ylä-Herttuala
	Vincenzo Cerullo
	Antonio Federico
	Dario Greco
**Order of Authors Secondary Information:**	
**Additional Information:**	
**Question**	**Response**
Are you submitting this manuscript to a special series or article collection?	No
**Experimental design and statistics**<br><br>Full details of the experimental design and statistical methods used should be given in the Methods section, as detailed in our Minimum Standards Reporting Checklist. Information essential to interpreting the data presented should be made available in the figure legends.<br><br>Have you included all the information requested in your manuscript?	Yes
**Resources**<br><br>A description of all resources used, including antibodies, cell lines, animals and software tools, with enough information to allow them to be uniquely identified, should be included in the Methods section. Authors are strongly encouraged to cite Research Resource Identifiers (RRIDs) for antibodies, model organisms and tools, where possible.<br><br>Have you included the information requested as detailed in our Minimum Standards Reporting Checklist?	Yes
**Availability of data and materials**	Yes

All datasets and code on which the conclusions of the paper rely must be either included in your submission or deposited in publicly available repositories (where available and ethically appropriate), referencing such data using a unique identifier in the references and in the "Availability of Data and Materials" section of your manuscript.<br><br>Have you have met the above requirement as detailed in our Minimum Standards Reporting Checklist?	
GigaScience has policies and guidelines in place for the use of generative AI-writing tools such as ChatGPT. If you have used such writing tools to assist with writing the manuscript this must be declared and cited in the text. Authors should not list AI-writing tools and other AI-assisted technologies as an author or co-author and should acknowledge that they are fully responsible for text generated or refined by AI-writing tools.<p><br><br>A summary of use (particularly in the introduction or among methods) needs to be included at the end of the paper, and the outputs should also be included as a supplementary file hosted in GigaDB or other open repositories. Please read our guidelines for more information. <p><br><br>By submitting to GigaScience, you are aware of the journal's AI-writing tools policy, and if you have declared use of such tools below, you have acknowledged this where appropriate in your manuscript and have made a summary of use and outputs available. <p><br><b>AI-assisted writing tools have been used in the preparation of this manuscript?	Yes

Manuscript Click here to access/download;Manuscript;EpitopeGenerationGateway.docx

Epitope Generation Gateway (EGG):

A biological context-dependent approach to cancer vaccine development

Luca Mannino[a], Michele Fratello[a], Jacopo Chiaro[bcde], Federica D'Alessio[bcde], Emanuele Di Lieto[a], Minna Niittykoski[f], Angela Serra[agh], Seppo Ylä-Herttuala[fi], Vincenzo Cerullo[bcdej], Antonio Federico[agh], Dario Greco[aht]

† Corresponding Author: dario.greco@helsinki.fi

Affiliations:

a: Faculty of Medicine and Health Technology, Tampere University, Tampere, Finland

b: Department of Pharmaceutical Biosciences, University of Helsinki, Faculty of Pharmacy ImmunoViroTherapy Lab, Drug Research Program, Helsinki, Finland

c: Helsinki Institute of Life Science (HiLIFE), University of Helsinki, Helsinki, Finland

d: Translational Immunology Program (TRIMM), Faculty of Medicine Helsinki University, University of Helsinki, Helsinki, Finland

e: Digital Precision Cancer Medicine Flagship (iCAN), University of Helsinki, Helsinki, Finland

f: A.I. Virtanen Institute for Molecular Sciences, University of Eastern Finland, Kuopio, Finland

g: Tampere Institute for Advanced Study, Tampere University, Tampere, Finland

h: Division of Pharmaceutical Biosciences, Faculty of Pharmacy, University of Helsinki, Helsinki, Finland

i: Heart Center and Gene Therapy Unit, Kuopio University Hospital, Kuopio, Finland

j: Department of Molecular Medicine and Medical Biotechnology and CEINGE, Naples University Federico II, Naples, Italy

Abstract

Background

Personalized neoantigen discovery requires tools that not only identify putative epitopes but also prioritize candidates resilient to tumor immune escape. Here, we present Epitope Generation Gateway (EGG), a containerized pipeline that streamlines discovery and prioritization across variant types and human leukocyte antigen (HLA) contexts. EGG coordinates established components, Mutect2 for somatic single nucleotide variations (SNVs) and indels, STAR-Fusion for fusion events, ArcasHLA for HLA typing, and pVACtools for epitope sequence prediction, then augments peptide-level scores with biological context. For each patient, EGG builds co-expression and protein-protein interaction networks, uses centrality measures and CRISPR dependency data (DepMap) to flag genes important

for tumor fitness, and annotates source genes with Gene Ontology terms, Hallmarks of Cancer processes, and IntOGen driver status.

Results

This multi-layer information supports ranking of candidates that are predicted to be presented and that arise from genes essential to tumor fitness or embedded in key pathways, thereby reducing the likelihood of immune escape by mutations caused by treatment pressure. As a case study, we applied EGG to 30 TCGA skin cutaneous melanoma (SKCM) patients, demonstrating end-to-end execution and producing prioritized epitopes that emphasize peptides and alterations in high-centrality, dependency-supported essential genes.

Conclusions

Overall, EGG links epitope prioritization to tumor systems biology and evolutionary dynamics, prioritizing durable cancer vaccine targets from SNVs, indels, fusions, and splicing derived epitopes while streamlining dataset analysis. EGG is available to download at https://github.com/fhaive/epitope_generation_gateway.

Introduction

Cancer immunotherapy has emerged as a dominant therapeutic modality in modern oncology, fundamentally transforming treatment paradigms across multiple malignancies. Among the most promising immunotherapeutic approaches, personalized neoantigen vaccines exploit tumor-specific mutations to generate highly targeted immune responses, offering unprecedented precision in cancer treatment [1,2]. Unlike conventional cytotoxic therapies such as chemotherapy and radiotherapy, which broadly target rapidly dividing cells, neoantigen vaccines provide enhanced tumor specificity and may mitigate the systemic toxicity associated with these standard interventions [3].

The therapeutic efficacy of cancer vaccines essentially depends on their capacity to trigger robust immune responses while maintaining tumor specificity, minimizing off-target effects that could compromise patient safety [4]. Current computational pipelines can identify candidate neo-antigens from SNVs, small insertions or deletions (indels), gene fusions, and aberrant splicing events. Early generation tools primarily targeted SNVs and indels with exclusive focus on Major Histocompatibility Complex (MHC) class I ligand prediction, addressing only CD8$^+$ T-cell responses [5–7]. Subsequent frameworks expanded to include gene-fusion breakpoints and now commonly support both class I and class II molecule prediction, engaging both cytotoxic and helper T-cell responses. The most recently developed tools further capture epitopes from aberrant splicing events [8–10]. However, existing

approaches largely overlook the functional context of neoantigens, failing to integrate them within their broader transcriptomic landscape or consider the biological significance of their source genes. Moreover, vaccine efficacy is challenged by the tumor intrinsic capacity for immune evasion and the functional importance of targeted mutations. Recent findings by Al Bakir et al. demonstrate that tumors can eliminate immunogenic targets through clonal deletion under selective pressure, emphasizing the critical need to prioritize mutations in functionally indispensable genes [11]. Effective neoantigen selection must therefore consider gene essentiality: alterations in genes essential for malignant fitness or in hub genes anchoring critical signaling pathways are less likely to be purged during tumor evolution, thus representing more durable vaccine targets. Gene co-expression networks provide a practical framework for inferring such functional importance, as network centrality metrics correlate with gene essentiality and the evolutionary cost of mutational escape [12].

Systems biology treats cancer as a dynamic, complex disease, where phenotype arises from perturbed interactions rather than isolated genes. Network models are widespread tools for representing and analyzing complex systems such as tumor biology. Yet, most neoantigen pipelines still emphasize sequence level features for epitope prioritization (such as peptide MHC binding) and give limited weight to the broader context that governs whether a candidate peptide is generated, displayed and retained by the tumor. Evidence shows that subcellular origin influences both presentation and outcome: neoantigens from plasma membrane proteins are disproportionately presented and have been associated with improved responses to immune checkpoint blockade [13,14]. At the same time, tumors can evade immunity by purging dispensable targets during evolution, underscoring the value of prioritizing alterations in vital genes, which are less likely to be lost [11,15,16].

To address these gaps, we developed Epitope Generation Gateway (EGG), an automated workflow that integrates standard callers and predictors: Mutect2 [17] for somatic SNVs/indels, STAR-Fusion [18] for fusion events, ArcasHLA [19] for HLA typing, and pVACtools [20–22] for class I/II epitope prediction. EGG augments sequence level scoring with essentiality aware features and places each candidate in its biological setting by building patient specific co-expression/protein-protein-interaction (PPI) networks. For prioritization, EGG integrates multiple layers of evidence regarding the source gene. These include patient specific co-expression/PPI centrality measures as network derived indicators of gene importance or essentiality, complemented by CRISPR knockout based dependency scores from DepMap [23] that provide experimentally grounded, though not patient-specific, gene essentiality information. Candidate genes are further annotated with Gene Ontology [24] terms and MSigDB Cancer Hallmarks [25] gene sets to capture pathway-level context, as well as with IntOGen [26] annotations to identify established cancer driver genes. Taken together, these data-driven and knowledge based features help to prioritize neoantigens that are not only predicted to be presented

but also arise from genes whose functions are critical to tumor fitness and therefore less likely to be lost through immune evasion. EGG's design is intentionally holistic: it integrates patient specific networks with curated biological knowledge (GO, MSigDB Cancer Hallmarks, DepMap) and presentation relevant attributes (cell membrane localization). The goal is a bigger-picture view of each epitope linking peptide level scores to tumor systems biology so that selected targets are immunologically plausible, and evolutionarily durable. EGG has been made available for download from https://github.com/fhaive/epitope_generation_gateway.

Material and Methods:

Data Acquisition and Preprocessing

Matched tumor RNA-seq, tumor DNA (WES), and normal DNA samples were retrieved from The Cancer Genome Atlas (TCGA) [27] for 30 patients diagnosed with cutaneous melanoma (TCGA-SKCM study code). Patients were randomly selected from the subset with available primary tumor RNA-seq data, corresponding tumor DNA, and matched normal DNA, explicitly excluding metastatic tissue. Datasets were retrieved using the Genomic Data Commons (GDC) client (gdc-client) [28], leveraging unique patient identifiers (UUIDs).

Sequencing alignment files in BAM format were downloaded and converted to paired-end FASTQ format to facilitate compatibility with the EGG pipeline. Conversion was performed using SAMtools (v1.21) [29,30], which involved: splitting BAM files by read group, sorting reads by name and extracting paired reads as compressed FASTQ files. These steps ensured the correct input data format for subsequent processing modules.

Read Quality Control and Trimming

Raw sequencing reads from tumor RNA-seq, tumor DNA, and matched normal DNA samples (all in FASTQ format) were processed using FastQC (v0.11.9) [31] and fastp (v 0.23.2) [32] to assess and improve read quality. Quality assessment was performed with FastQC to identify issues such as base quality degradation. Read trimming and filtering were subsequently carried out using fastp, which automatically detected and removed adapter sequences and excluded reads with poor quality metrics. Specifically, reads containing bases with Phred scores below 20 or with more than 30% unqualified bases were discarded. Additionally, reads shorter than 30 base pairs were removed. A sliding window quality trimming approach with a mean quality threshold of 20 was applied. All trimming settings were specified in a centralized YAML configuration file (QC_filtering_config_0.yaml), allowing users to easily adapt the preprocessing pipeline to different requirements.

RNA-Seq Processing, Fusion Detection and HLA typing

Trimmed cancer RNA-seq reads were processed using STAR-Fusion (v1.15.0) [18], which aligned reads and detected gene fusion events using the GRCh38 / GENCODE v37 CTAT genome reference [33,34]. This tool generated both fusion transcript calls and alignment files for downstream quantification. Reference genomes and transcript annotations (GRCh38 / GENCODE v37) were acquired from the CTAT genome library provided by STAR-Fusion [35]. These resources were automatically retrieved and managed by the pipeline. STAR-Fusion was used with default filters, which require at least one junction read and one spanning read (or ≥2 junction reads if no spanning support) and a minimum expression support of FFPM (fusion fragments per million total reads) ≥0.1.

Gene expression quantification was performed with the featureCounts function from the Rsubread Bioconductor package (v2.0.8) [36,37], using the same annotation reference to compute raw read counts for each gene in paired-end mode. These count matrices served as input for sample specific co-expression network production implemented in later stages of the pipeline.

To enable personalized neoepitope prediction, HLA typing was conducted using arcasHLA (v0.5) [19], which genotypes both MHC class I and class II loci from RNA-seq data. After sorting the aligned BAM files with SAMtools (v1.21) [29,30], unmapped reads were extracted and used to predict HLA alleles. The output was converted into a format compatible with pVACtools to support downstream MHC binding predictions.

Both STAR-Fusion and arcasHLA were executed within Docker containers, ensuring reproducibility and consistency across environments. Other steps, including featureCounts and BAM sorting, were integrated into the Snakemake [38] pipeline through dedicated Conda environments.

Fusion Epitopes Prediction

Post-alignment RNA fusion calls were processed for neoepitope prediction using the pVACfuse module of pVACtools (v5.3) [21], which integrates gene fusion annotations with HLA typing and MHC binding prediction. Fusion predictions, generated by STAR-Fusion, were first annotated using AGFusion (v1.4.3) [39] with the Ensembl GRCh38 release 103 transcript database [40] and Pfam-A domain annotations [41]. AGFusion resolved coding fusion junctions and transcript models, generating protein sequences suitable for downstream analysis.

These annotated fusion sequences were then passed to pVACfuse, which identified candidate fusion-derived neoantigens using a user-defined panel of MHC class I and II binding prediction algorithms (MHCNuggetsI and MHCnuggetsII) [42]. HLA alleles were automatically parsed from ArcasHLA outputs however in the practical use of the pipeline they can be optionally provided as pre-existent files if the

user wants to calculate them using different methods. Parameters such as epitope length (e1, e2), binding affinity thresholds, and the number of prediction threads were specified in the Snakemake pipeline and can be modified by the user by using different pVACtools options including different prediction algorithms.

All fusion epitope analyses were executed within Docker containers to ensure version consistency and reproducibility across environments. The containerized pipeline streamlined fusion annotation, neoantigen calling, and intermediate file handling, enabling high-throughput and customizable analysis per sample.

SNV and Indel Detection in Tumor and Normal DNA Samples:

All genomic analyses were conducted using the GRCh38 reference genome [34]. The reference FASTA file, its associated dictionary, and BWA index files were downloaded from the UCSC Genome Browser [43] and generated using SAMtools (v1.16), Genome Analysis Toolkit (GATK, v4.5.0.0) [44,45] CreateSequenceDictionary, and BWA index [46], respectively. Additional resources used in variant calling included a dbSNP [47] VCF (Broad Institute, dbSNP build 138), the gnomAD allele frequency panel [48], and the GATK-provided Panel of Normals (PON). These files were fetched from Broad Institute cloud repositories (https://gatk.broadinstitute.org/hc/en-us/articles/360035890811-Resource-bundle) and indexed with GATK and tabix [49] as needed.

Trimmed DNA-seq reads were processed following the GATK best practices pipeline for short variant discovery. Reads were aligned to the GRCh38 reference genome using BWA-MEM (v0.7.17) [50], with read group headers automatically extracted from the FASTQ file headers to include metadata including flowcell ID, lane, and barcode. These values were parsed directly from the first line of each FASTQ file to populate the read group (RG) tags used in alignment. The resulting alignments were sorted by coordinate using SAMtools.

To handle multi lane sequencing data, lane specific BAM files for each sample were merged using Picard (v2.27.4) MergeSamFiles [51] Duplicate reads were marked .using GATK MarkDuplicatesSpark to mitigate PCR induced biases. Base quality scores were recalibrated using BaseRecalibrator and ApplyBQSR with known variant sites from dbSNP as training data. These steps produced high-confidence, analysis-ready BAM files per sample.

Alignment metrics, including overall mapping quality and insert size distribution, were collected using GATK CollectAlignmentSummaryMetrics and CollectInsertSizeMetrics respectively. BAM files were indexed using SAMtools to enable efficient access during variant calling and downstream analysis. This preprocessing pipeline was applied to tumor and matched normal DNA samples for each patient,

ensuring consistency and compatibility for downstream somatic variant calling with tools such as Mutect2 and HaplotypeCaller.

Germline variants were called on normal DNA samples using GATK HaplotypeCaller, which performs local de novo assembly of haplotypes in regions showing evidence of variation. Raw variant calls were separated into SNPs and indels, followed by hard filtering based on standard GATK quality metrics (e.g., QD, FS, MQ, SOR). Filter-passing variants were selected, sorted, compressed, and concatenated into a single filtered germline VCF file per sample using BCFtools (v1.16) [29] and tabix utilities. These high-confidence germline calls were retained for downstream annotation and comparison against somatic variants.

Somatic variants were identified using GATK Mutect2 by jointly analyzing tumor and matched-normal BAM files per sample. The analysis incorporated a germline resource (gnomAD) and a panel of normals to reduce false positives. Mutect2 output included both raw variant calls and strand orientation artifact data (F1R2).

To refine variant calls, contamination estimates were calculated using GATK GetPileupSummaries and CalculateContamination, followed by orientation bias modeling with LearnReadOrientationModel. Final variant filtering was carried out using FilterMutectCalls, integrating all calculated metrics as additional flags in the VCF for each sample.

Genomic Variant Annotation and Epitope Prediction

To enable downstream neoepitope prediction using pVACtools, we performed additional annotation and preprocessing of somatic and germline variant calls. Variant Effect Predictor (VEP, Ensembl release 103) [52] was used to functionally annotate VCF files from somatic and germline variant calling workflows. VEP was configured in offline mode using the GRCh38 cache and a subset of plugins (e.g., Frameshift, Wildtype) downloaded from the Ensembl GitHub repository. Annotations were run via command-line within a Docker environment using the griffithlab/pvactools container (v5.3). The pipeline was configured to automatically pull the Docker image if not already present. Somatic VCFs (from Mutect2) and filtered germline VCFs (from HaplotypeCaller) were annotated with VEP, producing functionally enriched VCF files for subsequent pVACseq input. While downstream neoepitope analysis focused on somatic mutations, filtered germline variants were obtained to support phasing. These variants were merged with somatic calls and jointly phased using GATK ReadBackedPhasing. This step allowed identification of compound heterozygous or cis/trans configured variants that could influence peptide sequences derived from cancer genomes.

In parallel, gene expression levels were quantified from tumor RNA-seq data using Kallisto (v0.46.1) [53], a lightweight pseudoalignment-based transcript quantifier. A Kallisto index was built from Ensembl cDNA reference transcripts (release 103), and tumor RNA reads from multiple sequencing lanes were jointly quantified. The resulting expression data was integrated into the annotated somatic VCF using vcf-expression-annotator (vatools package v5.2.0), aligning expression values with corresponding variants.

To generate VCFs compatible with pVACtools, tumor-only variants were extracted using GATK SelectVariants. To enable inclusion of germline variants in neoepitope screening, sample identifiers in the germline VCFs were harmonized with the tumor VCF using BCFtools (v1.17). Germline and tumor variants were then merged using GATK CombineVariants and sorted using Picard SortVcf. These combined VCFs were phased using GATK ReadBackedPhasing and re-annotated with VEP to account for phasing effects on coding consequence.

Human leukocyte antigen (HLA) types were obtained via ArcasHLA as described earlier. HLA types were automatically extracted from ArcasHLA genotype JSON files and formatted into a pVACtools compatible input structure. Alternatively, users have the option to supply their own HLA typing externally.

The primary pVACseq analysis was then performed using the pvacseq run command inside the griffithlab/pVACtools Docker container (v5.3). The workflow supported all pVACseq-compatible MHC binding prediction algorithms, and for this analysis specifically used MHC_nuggetsI, and MHCnuggetsII. Both phased variant calls and Kallisto derived RNA expression data were supplied to enhance neoepitope prioritization. All pVACseq parameters are user configurable by modifying the Snakemake script directly. For this analysis, we used the following key parameters: --epitope-lengths 8-15 for class I predictions and --epitope-lengths 11-30 for class II, along with --depth 1000, and --phased-proximal-variants-vcf to incorporate phasing effects. Somatic Mutect2 calls were filtered (via Mutect2's post-filtering), but variants flagged by filters (i.e. non-PASS) were not excluded both flagged and PASS variants were retained. This decision was made deliberately to capture the broadest possible mutation pool as a proof-of-concept, enabling EGG's downstream prioritization framework to demonstrate its capacity to refine even heterogeneous candidate sets arising from a relatively small (n=30) patient cohort.

Alternative Splicing-Derived Neoepitope Prediction

Tumor-specific alternative splicing events were identified and processed for neoepitope prediction using a combination of RegTools (v1.0.0) [54] and pVACsplice from the pVACtools suite. First, splice

junction annotations were extracted from tumor RNA-seq alignments using RegTools, which identifies novel splice sites and exon-skipping events by comparing observed junctions against reference transcript models.

The analysis was based on the GRCh38/GENCODE v37 reference annotation to identify putative tumor-specific splicing variants. Junction files obtained in output were filtered to retain high-confidence events based on read support thresholds and junction quality metrics.

Identified splicing events were then exploited for neoepitope prediction using pVACsplice (from package pVACtools v5.3), which generates peptide sequences spanning novel exon-exon junctions and predicts their MHC binding potential. The workflow required both the RegTools junction output and the corresponding genomic VCF file containing somatic variants to account for potential interactions between splicing alterations and point mutations.

HLA alleles were provided either from ArcasHLA outputs or user-specified files, maintaining consistency with other neoepitope prediction modules. pVACsplice analysis employed the same MHC binding prediction algorithms used in pVACseq (MHCnuggetsI and MHCnuggetsII) to ensure comparable scoring across different neoantigen sources. Epitope lengths were set to 8-15 amino acids for MHC class I and 11-30 for MHC class II predictions.

All splicing-derived neoepitope analyses were executed within the griffithlab/pvactools Docker container to maintain version consistency and reproducibility. Parameters including minimum junction read support, binding affinity thresholds, and prediction algorithms were configurable through the centralized Snakemake workflow.

Patient Specific Networks Generation

Gene level raw read count matrices previously produced in the RNA-seq processing step (described in the RNA-Seq Processing, Fusion Detection and HLA typing paragraph) were carried forward for patient-specific co-expression network generation. Genes with low expression (fewer than 15 reads in at least in 20% of samples) were filtered out to reduce noise.

For downstream network analysis, variance stabilizing transformation (VST) was applied to the expression count using the DESeq2 package (v1.46.0) [55]. This transformation adjusts for the strong relationship between gene expression level and variability, producing a matrix in which genes with different expression ranges can be more reliably compared. The resulting normalized expression matrix was used to construct patient-specific co-expression networks.

To estimate individual level gene co-expression patterns, we applied the LIONESS (Linear Interpolation to Obtain Network Estimates for Single Samples) framework [56]. For a set of N samples, the aggregate network was computed using Pearson correlation between all gene pairs (i, j) across the full expression matrix.

To estimate the network specific to a sample q, we first computed the leave-one-out network $e_{ij}^{(\alpha-q)}$, constructed from all samples except q. The individual sample network $e_{ij}^{(q)}$ was then derived using the LIONESS formula:

$$e_{ij}^{(q)} = N\left(e_{ij}^{(\alpha)} - e_{ij}^{(\alpha-q)}\right) + e_{ij}^{(\alpha-q)}$$

This equation quantifies the contribution of sample q. to each edge (i, j) by interpolating its influence relative to the full dataset. All sample-specific adjacency matrices were stored in sparse matrix format to facilitate downstream graph based analyses.

Following LIONESS based reconstruction, we filtered the sample-specific networks to prioritize biologically interpretable and functionally relevant edges. First, we intersected each network with an extended PPI network the HumanNet-XN v2, an integrated human functional interaction network anchored in protein-protein interactions and extended with co-expression, pathway, interolog and co-citation evidence [57]. HumanNet edges were mapped from Entrez to Ensembl gene identifiers using the org.Hs.eg.db library (v3.18.0) [58].

To construct the filtered networks, we applied a two-step edge selection strategy that combined the extended PPI informed filtering with membrane-focused expansion:

1. PPI-informed edge selection: For each gene, we retained edges with absolute correlation weights ranking in the top 20% among all edges connecting that gene to its PPI-linked neighbors. This approach prioritized patient-specific co-expression patterns that aligned with known protein-protein interactions.
2. Membrane-enriched network expansion: To supplement the PPI-filtered network, we incorporated additional edges from the gene co-expression network that involved membrane-associated genes. Specifically, we first applied the PPI filtering described above, which yielded N edges. We then calculated 5% of N to determine the target number of additional edges to include. From the full gene co-expression network, we selected the top-ranked edges (by absolute correlation weight) where at least one gene was annotated as membrane associated, up to this 5% threshold. This strategy enriched the network for membrane localized genes

while maintaining focus on the strongest co-expression relationships. Membrane associated genes were identified using Gene Ontology (GO) annotations from the following terms and their descendant terms in the GO hierarchy:

- GO:0005886 (*plasma membrane*)
- GO:0005887 (*integral component of plasma membrane*)
- GO:0046658 (*anchored component of membrane*)
- GO:0009897 (*external side of plasma membrane*)
- GO:0009986 (*cell surface*)

Membrane gene annotations were obtained from the GAF file provided by the GO Consortium and mapped to Ensembl IDs via UniProt [59] and BioMart (v2.58.0) [60].

Both the PPI informed edge selection and the membrane-enriched network expansion are optional; the corresponding percentages can be modified via the configuration files or effectively switched off. The final output consisted of sparse adjacency matrices representing filtered, patient-specific co-expression networks enriched for interactions with known biological support and immunological relevance. We have named these networks: Patient Specific Functional Interaction Networks (pFINs) These network matrices were saved in Matrix Market format to ensure compatibility with external graph analysis frameworks.

The pFINs exhibited a characteristic topology consisting of a dominant largest connected component (LCC) alongside smaller, isolated subnetworks. The LCC encompassed the vast majority of edges and nodes, forming a cohesive structure where genes were interconnected through direct or indirect relationships. The remaining nodes formed small, disconnected clusters or isolated pairs that did not connect to the main component. This topology, with most network connectivity concentrated in a single large component, provided a natural framework for assessing gene importance: genes critical to network integrity would be those whose removal disrupted the LCC structure most severely.

Centrality Metrics for Gene Prioritization

To evaluate the functional relevance and network prominence of candidate neoantigen source genes, we calculated node level centrality scores within each sample-specific co-expression network.

To compare the relative connectivity of genes, we computed the Weighted Connectivity Impact (WCI) for each node v, defined as the fraction of total network edge weights attributable to its immediate connections.

Where each edge is normalized as:

$$\widetilde{w_{uv}} = \frac{|w_{uv}|}{\max_{i,j}|w_{ij}|}$$

The numerator is the sum of normalized absolute weights of all edges incident to *v*:

$$\text{strength}(v) = \sum_{u \in N(v)} \widetilde{w_{uv}}$$

Weighted Connectivity Impact calculated as:

$$\text{WCI}(v) = \frac{\text{strength}(v)}{\sum_{e \in E} \widetilde{w_e}}$$

This yields a sample-scaled centrality score between 0 and 1, allowing for cross-sample comparisons of gene connectivity despite differences in total edge weight. Genes with high WCI constitute a disproportionately large proportion of overall network strength.

pFINs Centrality Metrics

To assess the topological relevance of neoantigen-associated genes within the pFINs networks, we computed several centrality metrics.

The first centrality metric is the Largest Component Impact (LCI) quantifies a gene's contribution to the integrity of the network's Largest Connected Component (LCC) obtained from the PPI filtering steps explained above. It is a simple way to combine strength and degree in a comparable way between different networks. For each gene *v*, the LCI was computed by measuring the change in both node connectivity and edge weight upon virtual deletion of that gene:

$$\text{Largest Component Impact}(v) = \frac{1}{2}\left[\frac{S_0 - S_v}{S_0}\right] + \frac{1}{2}\left[\frac{W_0 - W_v}{W_0}\right]$$

Where:

- S_0 is the number of nodes in the LCC of the original network,
- $S_v$is the size of the LCC after removing node *v*,
- W_0 is the sum of normalized absolute edge weights within the original LCC,
- W_v is the corresponding sum after removing *v*.

This metric captures both structural fragmentation and, in turn, functional disconnection, identifying nodes whose removal disproportionately disrupts network integrity. Conceptually, LCI relates to percolation theory, where the removal of critical nodes can trigger a phase transition that fragments a network into disconnected components. By quantifying the impact of individual gene removals on the LCC, LCI identifies genes that act as critical connectors maintaining network cohesion.

The second centrality metric computed was betweenness centrality, a dynamic node centrality measure that reflects how information propagates through the networks. In addition, we calculated node degree and weighted strength for each gene, with strength defined as the sum of the absolute weights of its incident edges using igraph (v2.0.3) [61].

Pan-cancer Gene Essentiality Scoring

To incorporate gene biological relevance into neoantigen prioritization, we obtained pan-cancer essentiality scores from the DepMap Public 24Q4 dataset [23]. The CRISPR gene effect matrix (CRISPRGeneEffect.csv) was downloaded from the Broad DepMap Figshare repository (article ID 27993248) and processed in R. This matrix contains Chronos-derived gene effect values that represent copy number corrected knockout fitness effects across cancer cell lines, where more negative values indicate greater cellular dependency [62].

For each gene, we computed the median Chronos gene effect across all available cell lines to derive a single pan-cancer essentiality score (PanCancer_Median_Chronos). Following DepMap guidelines, genes with scores < -0.5 indicate depletion in most cell lines, while scores < -1.0 represent strong essentiality. These pan-cancer essentiality scores were subsequently integrated into the epitopes prioritization workflow to favor epitopes derived from functionally necessary genes.

Cancer Driver Gene Annotation

To identify neoantigens derived from cancer driver genes, we obtained driver annotations from the IntOGen Drivers compendium (version 20230531) [26]. The compendium file was processed to retain all genes classified as either “activating” (oncogenic; Act) or “loss-of-function" (tumor suppressor; LoF) roles across cancer types. This annotation was integrated into the neoantigen prioritization framework to identify neoantigens originating from genes with established roles in cancer pathogenesis, as mutations in driver genes may be particularly relevant for immune targeting strategies.

Consensus Prioritization

Candidate neoantigens were prioritized using a weighted Borda rank aggregation implemented in R. For each feature, epitopes were ranked according to expected directionality: ascending for Median.MT.IC50.Score and Depmap_survivability_score, and descending for network metrics

(Network_Betweenness, Network_Degree, Network_Impact, Network_Strength, Network_WCI). Feature-specific ranks were converted to Borda points and combined using predefined weights (default: 0.25 each for Median.MT.IC50.Score and Depmap_survivability_score; 0.10 for each network metric). The aggregate Borda score determined the final ordering, with ties resolved by average rank. Missing feature values were ignored and remaining weights renormalized per epitope. The prioritization (features, weights, rank direction, tie rule, and NA handling) is configurable via a YAML file without code changes, allowing emphasis on binding affinity, network centrality, or gene essentiality as needed.

Results and Discussion

EGG analytical flow

The complexity of cancer immunotherapy requires sophisticated computational approaches that can identify not just any neoantigens, but those most likely able to elicit appropriate immune responses and to remain resistant to evolutionary changes in the cancer. To address this challenge, we developed a comprehensive pipeline that integrates both established algorithms to extrapolate tumor specific neoantigens and novel network-based methodologies to improve neoantigen prioritization based on their biological context. On one hand, our pipeline includes quality control, preprocessing, extensive mutational analysis, transcriptomics data analysis with identification of fusion events, alternative splicing, and HLA typing. The additional value in this study is that we developed novel network-based methods to infer the biological context of the identified neoantigens. Such methods include the inference, manipulation and analysis of sample specific molecular networks. We envision that by integrating data driven interactional properties of network models with prior knowledge from multiple public databases, it is possible to achieve a robust and informed prioritization that improves efficacy and streamlines pre-clinical pipelines.

The EGG pipeline comprises seven modules ranging from quality control and data preprocessing to interpretable ranking of neoantigens. The diagram in Figure 1 provides a detailed overview of the pipeline structure as well as the included tools. The first module, Module 0, is dedicated to quality control and filtering of the raw sequencing reads using fastp for read trimming and fastqc for quality assessment. The subsequent mutation detection module (Module 1A) analyzes paired tumor-normal DNA samples, utilizing Mutect2 for somatic variant identification and HaplotypeCaller for germline variant detection Module 1B, on the other hand, processes trimmed RNA-seq cancer data through three analytical pathways: STAR-Fusion identifies structural variants, arcasHLA determines patient-specific HLA allotypes, and FeatureCounts (from Rsubread) generates expression profiles which are then used in network-based downstream analysis. Somatic variants identified in Module 1A undergo

allele-specific epitope prediction in Module 2A, where the pVACseq framework evaluates SNVs and indels for MHC-binding potential. This module also performs preprocessing steps required by pVACseq, including quantification of transcripts with Kallisto and phasing variants by integrating somatic and germline calls to preserve haplotype context. Fusion transcripts detected by STAR-Fusion are analyzed in Module 2B where pVACfuse is used to predict neo-antigens spanning fusion junctions.

Module 2C extracts alternative splicing events by validating variants occurring at splicing sites obtained from Module 1A, via Mutect2, by applying RegTools identifying tumor-specific splicing events, and subsequently processing these through pVACsplice to predict splice junction derived epitopes.

Finally, the single-sample network module constructs personalized co-expression networks using the LIONESS methodology on Pearson gene co-expression matrices derived from the RNA-seq counts generated in Module 1B. The resulting networks are filtered using HumanNet-XN protein-protein interaction data, and cellular membrane proteins are identified via Gene Ontology. Network centrality metrics are computed for both filtered and unfiltered patient-specific networks and are used, together with relevant biological information, to prioritize candidates with high potential therapeutic relevance. The network related calculation and generation is carried out by the network generation module while the final prioritization is carried out by the epitope prioritization module. Additional information for the execution of the pipeline, including details on the implementation (e.g., Snakemake workflow), the retrieval of required references and tools, as well as the execution of individual modules, are provided in Additional file 1 (Supplementary Material).

Figure 1. Epitope Generation Gateway Workflow. The EGG pipeline processes matched normal/tumor DNA (WGS/WES) and tumor RNA-seq data through seven integrated modules. Following quality control, DNA undergoes BWA-MEM alignment and GATK processing for germline and somatic variant calling. RNA processing includes STAR alignment, gene expression quantification, fusion detection (STAR-Fusion), and alternative splicing analysis. HLA typing and variant annotation are performed in parallel. Sample-specific co-expression networks are constructed using filtered HumanNet-XN and LIONESS methodology. Neoantigen prediction targets three categories: SNV/indel, fusion, and splicing neoantigens, all with MHC class I/II binding predictions. Network centrality analysis computes connectivity metrics for integrated prioritization combining MHC binding, centrality scores, and immunogenicity to generate prioritized neoantigens with biological context. Dashed boxes represent pipeline modules subdivision. Color scheme: blue boxes represent input data, orange boxes represent intermediate analyses or results, purple boxes denote analyses whose outputs contribute to the consensus scoring, the red box represents the consensus prioritization step, and the green box indicates the final output of prioritized epitope tables.

Inter sample variability in somatic mutations relevant to neoantigens

To demonstrate EGG's comprehensive neoantigen prediction capabilities, we analyzed the mutational landscape of 30 TCGA skin cutaneous melanoma (SKCM) samples. As a descriptive view of the

mutational landscape of the samples, we highlight the subset of somatic variants identified by Mutect2 that passed GATK's contamination and quality filters and were annotated as protein-coding, cancer-specific alterations (Figure 2).

Specifically, we identified three classes of mutations potentially giving rise to cancer neoantigens, including SNVs (including splicing site modifiers), indels, and gene fusions. Our analysis revealed substantial inter-sample variability in mutational burden across all these three genomic alteration categories. As widely reported, including TCGA SKCM and subsequent WGS studies, SNVs are the predominant mutation class in melanoma reflecting UV-driven mutagenesis [63,64]. This is consistent with our results where we found 2,000-9,000 somatic SNVs, though one sample (TCGA-BF-AAOX) exhibited the highest burden at ~9,400 variants (Figure 2A). While indel frequencies were generally lower, ranging from <50 to ~300 events per sample, with TCGA-EB-A3XF displaying the highest burden at 286 indels (Figure 2B). Gene fusion events, detected through STAR-Fusion analysis of tumor RNA-seq data, showed the lowest absolute frequencies with 2-24 fusion events identified per sample (Figure 2C). These results confirmed that while SNVs dominate the mutational landscape (around 90% of all variants), also indels (around 5-10%) and gene fusions (usually less than 2-3% per sample) contribute meaningfully to the total neoantigen repertoire (Figure 2D). This comprehensive mutational profiling demonstrates the ability of EGG to capture the full spectrum of genomic alterations that can generate immunologically relevant neoantigens, positioning it as a robust tool for personalized cancer immunotherapy target identification.

Figure 2. Mutational landscape analysis across TCGA samples processed by the EGG pipeline. (A) Single nucleotide variant (SNV) counts per sample, showing variable mutational burden with the sample TCGA-BF-AAOX being highly mutated (~9,400 SNVs). (B) Indel distribution, with most samples displaying fewer than 100 events and the sample TCGA-EB-A3XF showing the highest 286 indels. (C) Gene fusion events, range between 2-24 per sample. (D) Proportion of SNVs, Indels and Fusion events per sample displayed as boxplots.

Neoantigen burden distribution across mutation types

Neoantigen discovery modules of EGG, implemented via the pVACtools suite, identified candidate epitopes derived from somatic variants (pVACseq), gene fusions (pVACfuse with STAR-Fusion input), and aberrant splicing events (pVACsplice with RegTools input) across the TCGA melanoma cohort. Within the considered sample cohort, we identified up to 200 epitopes in a single sample across the three mutational classes (Figure 3A–3C). The binding affinity of epitopes to the MHC class I and class II complexes were computed. MHC class II slightly outnumbered class I predictions, consistently with the broader binding motif diversity of class II molecules. These results highlight the heterogeneity of neoantigen repertoires across patients and mutation types. Since EGG jointly considers peptides

predicted to bind HLA class I and HLA class II alleles, it captures candidate neoantigens that could contribute to cytotoxic $CD8^+$ T-cell responses (class I) as well as helper $CD4^+$ T-cell responses (class II), two complementary arms of anti-tumor immunity thought to be important for durable responses. Moreover, by adding sample-specific co-expression network analysis as a second-tier prioritization, EGG reduces large candidate pools into a smaller set of biologically relevant and therapeutically promising targets, bridging the gap between computational prediction and the practical constraints of vaccine formulation.

Figure 3. Predicted neoantigen landscape across TCGA melanoma samples processed by EGG. (A) Candidate neoantigens derived from somatic variants using pVACseq, stratified by MHC class (I and II). Samples yielded up to 200 epitopes, with somatic mutations contributing the largest overall pool. (B) Candidate neoantigens derived from gene fusions using pVACfuse with STAR-Fusion input, showing smaller but variable contributions across samples. (C) Candidate neoantigens derived from aberrant splicing events.

Integrating gene essentiality signals into epitope candidate ranking

Despite current methods are capable of identifying a remarkable amount of cancer neoantigen arising from genomic events, such as somatic mutations, fusions, and alternative splicing, translating this abundance into a clinically actionable candidates remains challenging. Therapeutic cancer vaccines typically deliver only a limited epitope set per patient, most peptide or mRNA platforms administer usually between 10 to 20 epitope sequences, whereas recent DNA plasmid vaccines have encoded larger payloads (up to 40 epitopes) in combination with PD-1 blockade. These design choices reflect manufacturing constraints, immunogenicity considerations, and trial feasibility [65–67]. Although sequencing-based pipelines can yield dozens to hundreds of candidate epitopes per tumour after standard HLA complex binding filters, current vaccine platforms can only accommodate a small subset of these in a single formulation. As a result, personalized vaccine design is not only an epitope level selection problem but also a gene level one: when only 10 to 40 epitopes can be used, choosing which genes they represent becomes necessary [2,70]. Prioritising epitopes from genes that are embedded in central, functionally indispensable positions within tumour networks offers a principled way to bias these limited slots toward targets that may be harder for the tumour to downregulate or eliminate under immune pressure [4,71,72].

In this study, we hypothesise that an additional layer of biological contextualisation is required to rationally select effective epitopes that are, in turn, clinically relevant for the specific patient. To address this, EGG integrates a network based filtering strategy that captures patient specific gene

expression patterns and exploits several sources of prior knowledge to refine the search for cancer epitopes. Specifically, EGG builds co-expression networks that are representative of the patient-specific gene expression combinations. Such a network is modelled and refined by using experimentally validated protein-protein interactions and membrane protein annotations in order to leverage the biological relevance of the co-expression relationships. We call this integrated network, Patient Specific Functional Interaction Network (pFIN). pFINs serve as a context-aware framework for refining neoantigen candidates toward those most likely to exert meaningful therapeutic impact (Figure 4). pFINs isn't simply a gene co-expression network filtered by PPI we also enhanced it with the most confident connection related to membrane localized proteins. This membrane focused expansion is motivated by recent evidence that neoantigens derived from membrane localized proteins are enriched among immunogenic peptides and correlate with improved response to immune checkpoint blockade [13,14].

Figure 4. Network-based integration framework for epitope prioritization. RNA-seq data from patient specific cancer biopsies were processed within the EGG pipeline to generate raw gene expression counts. Pearson correlation across the normalized matrix was used to construct an aggregate gene co-expression network, from which patient-specific networks were derived. Each patient-specific co-expression network was subsequently filtered and refined for biological relevance. Networks were first constrained to interactions present in an extended protein-protein interaction (PPI) reference (HumanNet-XN). Edges were then filtered to retain the strongest connections per node. To enhance immunological relevance, edges involving membrane associated genes (annotated by Gene Ontology terms) were added. The final output consisted of weighted, patient-specific functional interaction networks enriched for biologically supported and immunologically prioritized interactions. The steps illustrated in panels A and B are implemented by the sample_networks_generation; further usage details are provided are available as Additional file 1 (Supplementary Material).

EGG exploits the interactional properties of the pFINs predicting the functional relevance of each epitope's gene of origin using topological metrics computed on the sample specific network degree, strength, betweenness centrality, and measures of network perturbation such as Weighted Connectivity Impact (WCI) and Largest Component Impact (LCI). In this manner it captures local connectivity, control of information flow, redundancy and resilience. These gene level features are integrated with orthogonal evidence: CRISPR dependency scores from DepMap as an experimental proxy for essentiality, and epitope level sequence metrics from pVACtools (e.g., predicted HLA class I/II binding), which assess the intrinsic immunogenic potential of the peptide independently of its gene of origin. Since these inputs derive from different experiments and sources, they are, per se, non-integrable. EGG converts each data source to ranks and aggregates them via a Borda procedure to produce a consensus ranked epitope list per sample (Figure 5). To aid in the biological interpretation,

prioritized genes are annotated with Gene Ontology terms, MSigDB Hallmarks of Cancer processes, and IntOGen driver labels. We incorporated these network topological features including degree, strength, betweenness centrality, and perturbation-based scores such as LCI since it has been shown that the placement of a gene within interaction networks is informative about its essentiality. The removal of highly connected and central proteins disproportionately fragments protein-protein interaction networks ("centrality-lethality" rule) [73–75]. Percolation style measures that quantify the impact of node removal on the size and weight of the largest connected component, similar in spirit to our LCI, further highlight nodes whose loss compromises network integrity and the function of core pathways [12]. By prioritizing epitopes derived from genes with high LCI and high centrality, EGG explicitly favors targets whose deletion would incur a substantial systems-level fitness cost, and are therefore less likely to be suppressed through immune editing. This design directly addresses a limitation of current neoantigen vaccines: tumors frequently evade immune pressure by losing, downregulating, or epigenetically silencing immunogenic but dispensable neoantigens. Longitudinal studies in lung cancer under immune checkpoint blockade have documented selective loss of neoantigens and reshaping of the neoantigen landscape during therapy [15]. More broadly, neoantigen depletion, via copy-number loss or transcriptional repression is considered a major immune escape route that could reduce the efficacy of personalized vaccines [15]. Recent work further argues that vaccines targeting passenger neoepitopes will drive immune editing of those mutations, whereas antigens derived from oncogenic drivers or other functionally indispensable genes are more durable because their loss carries a higher fitness penalty [76].

In addition, Al Bakir et al. showed that even a clonal EGFR driver neoantigen can be lost under combined TKI and neoantigen vaccine pressure, illustrating that oncogenic drivers themselves may be affected by immune escape [11]. This indicates that simply labelling genes as drivers or non-drivers is insufficient and supports incorporating different measures of functional indispensability to select the pool of candidate cancer vaccine epitopes. By prioritising for network-central cancer and patient specific essential genes, EGG is designed to bias neoantigen selection toward targets that should be harder to lose under evolutionary pressure caused by the immune system and the cancer treatment. To complement these topology derived measures of indispensability with experimental evidence, we include pan-cancer CRISPR dependency data from DepMap, using Chronos gene effect scores as a quantitative measure of gene essentiality. Chronos models cell population dynamics to infer knockout fitness effects; more negative gene effect values indicate stronger depletion, with scores below -0.5 typically marking genes required for viability in most cell lines and scores near -1 characteristic of pan-essential genes [62]. These CRISPR knockout dependencies provide an alternative measure of gene essentiality that is independent of patient specific co-expression structure but is instead derived from

thousands of in vitro cancer cell lines. Integrative analyses have shown that network based features and genome scale CRISPR knockout screens capture overlapping but non identical sets of essential genes, and combining them can improve the selection of essential genes [73,77]. In EGG, network centrality metrics (degree, strength, betweenness, WCI, LCI) and DepMap derived essentiality are therefore aggregated in the Borda consensus. This multi-criteria scheme allows epitopes from genes that are both structurally central in patient-specific pFINs and strongly essential across cancer cell lines, while still capturing context-specific vulnerabilities seen only in one data source, thereby increasing the chance that selected vaccine targets will remain expressed as the tumor evolves.

Our strategy explicitly mitigates immune escape via deletion or silencing of dispensable neoantigen source genes, however it's main limitation is that it does not address escape mechanisms that circumvent antigen presentation altogether. Genetic and epigenetic alterations in HLA class I/II, β2-microglobulin, antigen processing machinery, and IFNγ signaling are frequent in clinical tumors and represent dominant routes of immune evasion under T cell pressure [78–80]. In such settings, neoantigen source gene essentiality becomes largely irrelevant, as tumor cells can evade recognition despite maintaining expression of the targeted gene.

Figure 5. Epitope prioritisation scheme. Patient Specific Functional Interaction Networks were used as the basis for epitope prioritization. Topological network metrics obtained from degree, edges strength, betweenness (including LCI, WCI) were computed to establish a measure of gene essentiality specific to individual patients. These network-derived features were combined with external biological evidence, including CRISPR-based gene essentiality scores (DepMap), epitope specific sequence metrics such as predicted HLA binding affinity. The integrated information was aggregated into a ranked epitope list, ensuring robust consensus prioritization. To provide additional biological context, candidate genes were annotated with cancer hallmarks, Gene Ontology terms, and IntOGen driver gene information. The resulting framework enables informed selection of epitopes by linking immunogenic potential to system-level relevance and cancer biology. The steps illustrated in this figure are implemented by the epitopes_prioritisation module with details of usage are provided in Additional file 1 (Supplementary Material).

Determinants of epitope prioritization

By applying our multi-step prioritisation approach described above across our TCGA melanoma samples, we identified correlation patterns among features utilised in the epitope prioritisation. Such pairwise relationships are summarised in Figure 6A. As expected, network centrality-based metrics are strongly intercorrelated and align closely with the Borda score, indicating that candidates from highly connected or influential genes tend to occupy top positions in the rank. DepMap survivability and predicted binding affinity showed a mild negative correlation with the Borda scores (negative values

imply greater essentiality), and only limited correlation with network centralities. While DepMap highlights experimentally observed dependencies and provides biologically informed metrics, network centrality measures capture data-driven patterns of connectivity based on the unique molecular landscape of each patient. Exploiting both levels of information yields a more robust prioritization compared to single layer prioritisation methods. We hypothesize that the prioritization of epitopes according to essentiality and network centrality will constrain immune escape, as the elimination of such genes is expected to impair tumor fitness. EGG's ranking aligns most strongly with network centrality measures, while also retaining contributions from sequence-dependent features like HLA binding affinity and gene essentiality from CRISPR knockout studies. HLAs expression were not included as ranking criterion, hence their lack of correlation to the Borda score. This variable, together with other optional pvactools algorithms outputs can be easily included by the user as a ranking criterion.

The fraction of epitopes deriving from known cancer drivers is enriched among the top prioritized epitopes compared to the overall pool. As shown in Figure 6, panel B, within the top 10 per sample, both loss-of-function and activating drivers contribute to this enrichment. The presence of driver genes toward higher scores may reflect the tendency of cancer driver genes to act as network hubs.

In order to ascertain whether overrepresented epitopes across the population exist, we then assessed the occurrence of epitope candidates across patients. We observed that exact peptides are unique to individual patients (panel D), while the genes that give rise to epitopes show a small recurrence across cases (panel C). Altogether, these descriptive analyses illustrate the intended behavior of EGG in this case study: the pipeline defines the candidate space onto epitopes anchored in tumor-relevant biology, enriches for driver-gene targets, and produces shortlists that remain largely patient-specific at the peptide level while still revealing a small cohort of recurrent genes and epitope sequences that could inform shared targets or follow-up functional studies. Building on this, our data pinpoints a small set of recurrent targets. Among fusion derived events, KANSL1 is the most frequently shared gene (12 samples; panel C), and DIYKQIRANKVSVWRQ the most recurrent exact epitope (5 patients; panel D). A comparable pattern emerges for genomic mutations: exact mutation-derived peptides remain patient-specific, but the gene NRAS shows the highest recurrence (7 samples; panel C), with the epitope LDILDTAGREEYSAMRDQYMRTGEGFLCV shared across 3 patients (panel D). In both fusion and genomic contexts, the presence of recurrent epitopes highlights the potential for "off-the-shelf" vaccination strategies, an approach already explored for shared/public neoantigens such as KRAS hotspots and frameshift peptides in MSI tumors. Early trials of shared neoantigen vaccines (including KRAS focused mRNA platforms and multi epitope regimens) demonstrate feasibility and immunogenicity,

underscoring the translational value of the recurrent sequences identified by epitope generation pipelines [81].

Fundamentally, the main outputs of EGG, are interactive prioritization tables for each sample that integrate epitope and gene level features into a unified view. These tables report the HLA allele predicted to present each epitope together with corresponding transcript abundance, peptide sequence, mutation source, and class I/II designation. For each candidate, network-derived features (degree, strength, betweenness, LCI, WCI) and external evidence (DepMap survivability, IntOGen driver status, cancer hallmark annotation, GO terms) are displayed alongside the consensus Borda score and rank. This design provides investigators with a transparent overview of how multiple layers of information contribute to prioritization, linking peptide level immunogenicity with the biological relevance of the source gene. Tables for each patient that include prioritised epitopes and their topological and biological features obtained in our analysis can be found in Additional file 2 (Supplementary Material).

Figure 6. Exploratory analysis of epitope features. (A) Spearman correlation between features used in the Borda ranking procedure, highlighting expected dependencies among network-based measures and binding affinity scores. (B) Fraction of epitopes derived from driver versus non-driver genes within the top-10 prioritized epitopes per sample, showing contributions from both loss-of-function (inactivating alterations disabling tumor suppressors) and activating (gain-of-function mutations activating oncogenes) drivers. (C) Distribution of genes shared between samples, showing that most candidate genes are unique to individual samples. (D) Distribution of epitope sequences shared between samples, with the majority being patient specific.

Comparison of currently available neoantigen prediction pipelines

EGG adds a co-expression-network based prioritization step that ranks neoantigens by the centrality and indispensability of their source genes, with the goal of reducing immune evasion risk. The pipeline is implemented in Snakemake and automates resource retrieval and environment setup for local execution. Compared with prominent publicly available pipelines surveyed in Table 1, EGG's network-aware prioritization constitutes a distinctive feature while keeping installation and execution simple. To contextualize EGG within the current landscape, we note the following pipelines: pVACtools stands out as a modular ecosystem (pVACseq/bind/fuse/splice) with extensive algorithm support and integrated visualization (pVACview) and vaccine design utilities (pVACvector); pVACtools also includes comprehensive workflows, such as immuno.wdl on the Terra platform, to automate end-to-end analyses, including upstream preprocessing. TSNAD v2.0 is an end-to-end pipeline for tumor-normal WES/WGS (with optional RNA-seq) that incorporates expression and gene-fusion filters and uses the deep-learning predictor DeepHLApan to score both HLA binding and immunogenicity [82,83].

ScanNeo2 provides an end-to-end workflow from raw DNA/RNA data and uniquely broadens variant coverage to include exitron splicing and gene fusions alongside SNVs/indels, enabling wider candidate recovery [9]. INTEGRATE-neo is purpose-built for fusion-derived neoantigens, offering an automated pipeline from fusion calling through HLA binding specifically for gene-fusion events [5]. nextNEOpi [84]is a Nextflow [85] based, fully automated pipeline that starts from raw WES/WGS in combination with RNA-seq and additionally quantifies patient and neoepitope level features linked to immunogenicity and response to checkpoint blockade. NeoHunter emphasizes flexible input handling and systematically detects and prioritizes neoantigens not only from SNVs/indels but also from gene fusions and aberrant splicing within a single framework [10]. NeoDisc is an end-to-end clinical proteogenomic pipeline that integrates immunopeptidomics with genomics and transcriptomics to prioritize both canonical and noncanonical tumor specific antigens [86]. SNAF focuses on splicing-derived antigens, predicting MHC-bound T-cell epitopes and altered-surface B-cell targets and using machine-learning prioritization to enrich for immunogenic events [8]. Seq2Neo provides a one-stop workflow from raw data and incorporates a convolutional neural network immunogenicity model to rank candidates derived from SNVs, indels, and gene fusions [87].

Crucially, the prioritization module of EGG is flexible and fully customizable by the user. Optional features can be enabled or disabled and thresholds adjusted, for example using different algorithm for peptide MHC binding, immunogenicity scores, gene essentiality, and multiple network-based centrality metrics. The relative weights of these features used during prioritization are also modifiable by the users, allowing researchers to emphasize specific criteria for a customizable ranking process.

Tool	Mutations considered	Input	MHC class	HLA typing	Co-expression context
EGG (Epitope Generation Getaway)	SNVs, indels, gene fusions, alt splicing	Fastqs	I, II	yes	yes
ScanNeo2	SNVs, indels, gene fusions, alt splicing	Fastqs, BAM, VCF	I, II	yes	no
INTEGRATE-neo	Gene fusions	GenePred, BEDPE	I	no	no
nextNEOpi	SNVs, indels, gene fusions	Fastqs, BAM	I, II	yes	no
pVACtools	SNVs, indels, gene fusions, alt splicing	Fastqs, VCF	I, II	yes	no
TSANAD v2.0	SNVs, indels	Fastqs, VCF	I	yes	no
NeoHunter	SNVs, indels, gene fusions, alt splicing	Fastqs, BAM, VCF	I	yes	no
NeoDisc	SNVs, indels, gene fusions, alt splicing	Fastqs, BAM, VCF, MS	I, II	yes	no
SNAF	Alt splicing	Fastqs	I, II	yes	no
Seq2Neo	SNVs, indels, gene fusions	Fastqs	I, II	yes	no

Table 1. Feature matrix for neo-antigen prediction pipelines. This table benchmarks the Epitope Generation Gateway (EGG) against leading public workflows, summarizing for each tool the classes of somatic alterations analyzed (SNVs, indels, gene fusions, splicing), accepted input formats, MHC coverage (class I and/or II), built-in HLA typing, and use of patient-specific gene co-expression data to provide biological context.

Conclusion

Epitope Generation Gateway (EGG) can bridge the gap between abundant raw neoantigen predictions and the practical need for biologically meaningful ordered results. By embedding candidate epitopes

within patient-specific functional interaction networks and incorporating orthogonal evidence of essentiality and driver status, EGG prioritizes epitopes more likely to be therapeutically relevant by prioritising more essential genes less likely to be subject to immune evasion while also offering the user with alternative prioritisation methods and important gene related biological information. In the case study presented in this work, we highlight that the majority of epitopes remain unique between patients at the sequence level, whereas gene level recurrence is more common. These results show that EGG can serve as a flexible framework for integrating diverse data sources, balancing immunological plausibility with tumor systems biology, and informing the rational design of personalized cancer vaccines.

Abbreviations

EGG: Epitope Generation Gateway, GO: Gene Ontology, HLA: Human Leukocyte Antigen, LCC: Largest Connected Component, LCI: Largest Component Impact, MHC: Major Histocompatibility Complex, pFIN: Patient Specific Functional Interaction Network, PPI: Protein Protein Interaction, SKCM: Skin Cutaneous Melanoma, SNV: Single Nucleotide Variation, TCGA: The Cancer Genome Atlas, vst: Variance Stabilizing Transformation, WCI: Weighted Connectivity Impact.

Data availability

The source code is publicly available at GitHub:
https://github.com/fhaive/epitope_generation_gateway

Competing interests

The authors declare that they have no competing interests that could have influenced the work reported in this paper.

Authors contributions

LM: Data Analysis, Investigation, Methodology, Software, Visualisation, Conceptualisation, Writing – original draft, Writing – review and editing, MF: Software, Methodology, Investigation, JC: Methodology, Writing – review and editing, FD: Methodology, Writing – review and editing, ED: Software, Writing – review and editing, MN: Writing – review and editing, AS: Methodology, Investigation, Writing – review and editing, SY: Conceptualisation, Funding Acquisition, Writing – review and editing, VC: Conceptualisation, Funding Acquisition, Writing – review and editing, AF: Conceptualisation, Investigation, Methodology, Supervision, Writing – original draft, Writing – review and editing, DG: Conceptualisation, Funding Acquisition, Investigation, Methodology, Supervision, Writing – review and editing

Additional files

Additional file 1 (.docx): Supplementary Methods / Supplementary Protocol.

Description: Step-by-step instructions for execution of individual pipeline modules.

Additional file 2 (.xlsx): Supplementary Tables.

Description: Patient-level tables of prioritised epitopes and associated topological and biological features.

Use of AI assisted tools

During manuscript preparation, we used AI assisted tools to improve clarity and readability of text and as a coding aid. AI-assisted suggestions were reviewed and edited by the authors, who take full responsibility for the final content.

Funding

This work was supported by the Jane and Aatos Erkko Foundation.

References

1. Sahin U, Derhovanessian E, Miller M, Kloke B-P, Simon P, Löwer M, et al.. Personalized RNA mutanome vaccines mobilize poly-specific therapeutic immunity against cancer. *Nature*. 2017; doi: 10.1038/nature23003.

2. Ott PA, Hu Z, Keskin DB, Shukla SA, Sun J, Bozym DJ, et al.. An immunogenic personal neoantigen vaccine for patients with melanoma. *Nature*. 2017; doi: 10.1038/nature22991.

3. Li X, You J, Hong L, Liu W, Guo P, Hao X. Neoantigen cancer vaccines: a new star on the horizon. *Cancer Biol Med*. 2023; doi: 10.20892/j.issn.2095-3941.2023.0395.

4. Bang H, Park JS, Kim JY, Sung C, An J, Cho D, et al.. Gene essentiality for tumour growth influences neoantigen-directed immunoediting. *Clinical & Translational Med*. 2022; doi: 10.1002/ctm2.714.

5. Zhang J, Mardis ER, Maher CA. INTEGRATE-neo: a pipeline for personalized gene fusion neoantigen discovery. Hancock J, editor. *Bioinformatics*. 2017; doi: 10.1093/bioinformatics/btw674.

6. Nielsen M, Lundegaard C, Blicher T, Lamberth K, Harndahl M, Justesen S, et al.. NetMHCpan, a Method for Quantitative Predictions of Peptide Binding to Any HLA-A and -B Locus Protein of Known Sequence. Kallas E, editor. *PLoS ONE*. 2007; doi: 10.1371/journal.pone.0000796.

7. Zhou Z, Lyu X, Wu J, Yang X, Wu S, Zhou J, et al.. TSNAD: an integrated software for cancer somatic mutation and tumour-specific neoantigen detection. *R Soc Open Sci*. 2017; doi: 10.1098/rsos.170050.

8. Li G, Mahajan S, Ma S, Jeffery ED, Zhang X, Bhattacharjee A, et al.. Splicing neoantigen discovery with SNAF reveals shared targets for cancer immunotherapy. *Sci Transl Med*. 2024; doi: 10.1126/scitranslmed.ade2886.

9. Schäfer RA, Guo Q, Yang R. ScanNeo2: a comprehensive workflow for neoantigen detection and immunogenicity prediction from diverse genomic and transcriptomic alterations. Kendziorski C, editor. *Bioinformatics*. 2023; doi: 10.1093/bioinformatics/btad659.

10. Ma T, Zhao Z, Li H, Wei L, Zhang X. NeoHunter: Flexible software for systematically detecting neoantigens from sequencing data. *Quant Biol*. 2024; doi: 10.1002/qub2.28.

11. Al Bakir M, Reading JL, Gamble S, Rosenthal R, Uddin I, Rowan A, et al.. Clonal driver neoantigen loss under EGFR TKI and immune selection pressures. *Nature*. 2025; doi: 10.1038/s41586-025-08586-y.

12. Li X, Li W, Zeng M, Zheng R, Li M. Network-based methods for predicting essential genes or proteins: a survey. *Briefings in Bioinformatics*. 2020; doi: 10.1093/bib/bbz017.

13. Goldberger Z, Hauert S, Chang K, Kurtanich T, Alpar AT, Repond G, et al.. Membrane-localized neoantigens predict the efficacy of cancer immunotherapy. *Cell Reports Medicine*. 2023; doi: 10.1016/j.xcrm.2023.101145.

14. Castro A, Kaabinejadian S, Yari H, Hildebrand W, Zanetti M, Carter H. Subcellular location of source proteins improves prediction of neoantigens for immunotherapy. *EMBO J*. 2022; doi: 10.15252/embj.2022111071.

15. Xie N, Shen G, Gao W, Huang Z, Huang C, Fu L. Neoantigens: promising targets for cancer therapy. *Sig Transduct Target Ther*. 2023; doi: 10.1038/s41392-022-01270-x.

16. Anagnostou V, Smith KN, Forde PM, Niknafs N, Bhattacharya R, White J, et al.. Evolution of Neoantigen Landscape during Immune Checkpoint Blockade in Non–Small Cell Lung Cancer. *Cancer Discovery*. 2017; doi: 10.1158/2159-8290.CD-16-0828.

17. Benjamin D, Sato T, Cibulskis K, Getz G, Stewart C, Lichtenstein L. Calling Somatic SNVs and Indels with Mutect2. Bioinformatics;

18. Haas BJ, Dobin A, Stransky N, Li B, Yang X, Tickle T, et al.. STAR-Fusion: Fast and Accurate Fusion Transcript Detection from RNA-Seq. Bioinformatics;

19. Orenbuch R, Filip I, Comito D, Shaman J, Pe’er I, Rabadan R. arcasHLA: high-resolution HLA typing from RNAseq. Ponty Y, editor. *Bioinformatics*. 2020; doi: 10.1093/bioinformatics/btz474.

20. Hoang M, Richters M, Kiwala S, Griffith O, Griffith M. 32. pVACsplice: A Computational tool for predicting and prioritizing alternative splicing neoantigens. *Cancer Genetics*. 2024; doi: 10.1016/j.cancergen.2024.08.034.

21. Hundal J, Kiwala S, McMichael J, Miller CA, Xia H, Wollam AT, et al.. pVACtools: A Computational Toolkit to Identify and Visualize Cancer Neoantigens. *Cancer Immunology Research*. 2020; doi: 10.1158/2326-6066.CIR-19-0401.

22. Hundal J, Carreno BM, Petti AA, Linette GP, Griffith OL, Mardis ER, et al.. pVAC-Seq: A genome-guided in silico approach to identifying tumor neoantigens. *Genome Med*. 2016; doi: 10.1186/s13073-016-0264-5.

23. DepMap B. DepMap 24Q4 Public. Figshare+;

24. The Gene Ontology Consortium, Carbon S, Douglass E, Good BM, Unni DR, Harris NL, et al.. The Gene Ontology resource: enriching a GOld mine. *Nucleic Acids Research*. 2021; doi: 10.1093/nar/gkaa1113.

25. Liberzon A, Birger C, Thorvaldsdóttir H, Ghandi M, Mesirov JP, Tamayo P. The Molecular Signatures Database Hallmark Gene Set Collection. *Cell Systems*. 2015; doi: 10.1016/j.cels.2015.12.004.

26. Martínez-Jiménez F, Muiños F, Sentís I, Deu-Pons J, Reyes-Salazar I, Arnedo-Pac C, et al.. A compendium of mutational cancer driver genes. *Nat Rev Cancer*. 2020; doi: 10.1038/s41568-020-0290-x.

27. The Cancer Genome Atlas Research Network, Weinstein JN, Collisson EA, Mills GB, Shaw KRM, Ozenberger BA, et al.. The Cancer Genome Atlas Pan-Cancer analysis project. *Nat Genet*. 2013; doi: 10.1038/ng.2764.

28. Zhang Z, Hernandez K, Savage J, Li S, Miller D, Agrawal S, et al.. Uniform genomic data analysis in the NCI Genomic Data Commons. *Nat Commun*. 2021; doi: 10.1038/s41467-021-21254-9.

29. Danecek P, Bonfield JK, Liddle J, Marshall J, Ohan V, Pollard MO, et al.. Twelve years of SAMtools and BCFtools. *GigaScience*. 2021; doi: 10.1093/gigascience/giab008.

30. Li H, Handsaker B, Wysoker A, Fennell T, Ruan J, Homer N, et al.. The Sequence Alignment/Map format and SAMtools. *Bioinformatics*. 2009; doi: 10.1093/bioinformatics/btp352.

31. : Babraham Bioinformatics - FastQC A Quality Control tool for High Throughput Sequence Data. https://www.bioinformatics.babraham.ac.uk/projects/fastqc/ Accessed 2025 Dec 17.

32. Chen S, Zhou Y, Chen Y, Gu J. fastp: an ultra-fast all-in-one FASTQ preprocessor. *Bioinformatics*. 2018; doi: 10.1093/bioinformatics/bty560.

33. Mudge JM, Carbonell-Sala S, Diekhans M, Martinez JG, Hunt T, Jungreis I, et al.. GENCODE 2025: reference gene annotation for human and mouse. *Nucleic Acids Research*. 2025; doi: 10.1093/nar/gkae1078.

34. Schneider VA, Graves-Lindsay T, Howe K, Bouk N, Chen H-C, Kitts PA, et al.. Evaluation of GRCh38 and de novo haploid genome assemblies demonstrates the enduring quality of the reference assembly. *Genome Res*. 2017; doi: 10.1101/gr.213611.116.

35. Ticke T, Bankapur A, Ganote C, Fulton B, Tirosh I, Chen J, et al.. Trinity CTAT: a community resource for *de novo* and reference-based RNA-Seq analysis.

36. Liao Y, Smyth GK, Shi W. The R package Rsubread is easier, faster, cheaper and better for alignment and quantification of RNA sequencing reads. *Nucleic Acids Research*. 2019; doi: 10.1093/nar/gkz114.

37. Liao Y, Smyth GK, Shi W. featureCounts: an efficient general purpose program for assigning sequence reads to genomic features. *Bioinformatics*. 2014; doi: 10.1093/bioinformatics/btt656.

38. Köster J, Rahmann S. Snakemake—a scalable bioinformatics workflow engine. *Bioinformatics*. 2012; doi: 10.1093/bioinformatics/bts480.

39. Murphy C, Elemento O. AGFusion: annotate and visualize gene fusions. Bioinformatics;

40. Cunningham F, Allen JE, Allen J, Alvarez-Jarreta J, Amode MR, Armean IM, et al.. Ensembl 2022. *Nucleic Acids Research*. 2022; doi: 10.1093/nar/gkab1049.

41. Mistry J, Chuguransky S, Williams L, Qureshi M, Salazar GA, Sonnhammer ELL, et al.. Pfam: The protein families database in 2021. *Nucleic Acids Research*. 2021; doi: 10.1093/nar/gkaa913.

42. Shao XM, Bhattacharya R, Huang J, Sivakumar IKA, Tokheim C, Zheng L, et al.. High-Throughput Prediction of MHC Class I and II Neoantigens with MHCnuggets. *Cancer Immunology Research*. 2020; doi: 10.1158/2326-6066.CIR-19-0464.

43. Kent WJ, Sugnet CW, Furey TS, Roskin KM, Pringle TH, Zahler AM, et al.. The Human Genome Browser at UCSC. *Genome Res*. 2002; doi: 10.1101/gr.229102.

44. McKenna A, Hanna M, Banks E, Sivachenko A, Cibulskis K, Kernytsky A, et al.. The Genome Analysis Toolkit: A MapReduce framework for analyzing next-generation DNA sequencing data. *Genome Res*. 2010; doi: 10.1101/gr.107524.110.

45. Van Der Auwera GA, Carneiro MO, Hartl C, Poplin R, Del Angel G, Levy-Moonshine A, et al.. From FastQ Data to High-Confidence Variant Calls: The Genome Analysis Toolkit Best Practices Pipeline. *CP in Bioinformatics*. 2013; doi: 10.1002/0471250953.bi1110s43.

46. Li H, Durbin R. Fast and accurate short read alignment with Burrows–Wheeler transform. *Bioinformatics*. 2009; doi: 10.1093/bioinformatics/btp324.

47. Sherry ST. dbSNP: the NCBI database of genetic variation. *Nucleic Acids Research*. 2001; doi: 10.1093/nar/29.1.308.

48. Karczewski KJ, Francioli LC, Tiao G, Cummings BB, Alföldi J, Wang Q, et al.. The mutational constraint spectrum quantified from variation in 141,456 humans. *Nature*. 2020; doi: 10.1038/s41586-020-2308-7.

49. Li H. Tabix: fast retrieval of sequence features from generic TAB-delimited files. *Bioinformatics*. 2011; doi: 10.1093/bioinformatics/btq671.

50. Li H. Aligning sequence reads, clone sequences and assembly contigs with BWA-MEM. arXiv;

51. : Picard Tools - By Broad Institute. https://broadinstitute.github.io/picard/ Accessed 2025 Dec 17.

52. McLaren W, Gil L, Hunt SE, Riat HS, Ritchie GRS, Thormann A, et al.. The Ensembl Variant Effect Predictor. *Genome Biol*. 2016; doi: 10.1186/s13059-016-0974-4.

53. Bray NL, Pimentel H, Melsted P, Pachter L. Near-optimal probabilistic RNA-seq quantification. *Nat Biotechnol*. 2016; doi: 10.1038/nbt.3519.

54. Cotto KC, Feng Y-Y, Ramu A, Richters M, Freshour SL, Skidmore ZL, et al.. Integrated analysis of genomic and transcriptomic data for the discovery of splice-associated variants in cancer. *Nat Commun*. 2023; doi: 10.1038/s41467-023-37266-6.

55. Love MI, Huber W, Anders S. Moderated estimation of fold change and dispersion for RNA-seq data with DESeq2. *Genome Biol*. 2014; doi: 10.1186/s13059-014-0550-8.

56. Kuijjer ML, Hsieh P-H, Quackenbush J, Glass K. lionessR: single sample network inference in R. *BMC Cancer*. 2019; doi: 10.1186/s12885-019-6235-7.

57. Hwang S, Kim CY, Yang S, Kim E, Hart T, Marcotte EM, et al.. HumanNet v2: human gene networks for disease research. *Nucleic Acids Research*. 2019; doi: 10.1093/nar/gky1126.

58. Carlson M. org.Hs.eg.db. Bioconductor;

59. Conin B, Billault-Chaumartin I, El Sayyed H, Quenech'Du N, Cockram C, Koszul R, et al.. Extended sister-chromosome catenation leads to massive reorganization of the *E. coli* genome. *Nucleic Acids Research*. 2022; doi: 10.1093/nar/gkac105.

60. Durinck S, Spellman PT, Birney E, Huber W. Mapping identifiers for the integration of genomic datasets with the R/Bioconductor package biomaRt. *Nat Protoc*. 2009; doi: 10.1038/nprot.2009.97.

61. Csárdi G, Nepusz T, Müller K, Horvát S, Traag V, Zanini F, et al.. igraph for R: R interface of the igraph library for graph theory and network analysis. Zenodo;

62. Dempster JM, Boyle I, Vazquez F, Root DE, Boehm JS, Hahn WC, et al.. Chronos: a cell population dynamics model of CRISPR experiments that improves inference of gene fitness effects. *Genome Biol*. 2021; doi: 10.1186/s13059-021-02540-7.

63. Hayward NK, Wilmott JS, Waddell N, Johansson PA, Field MA, Nones K, et al.. Whole-genome landscapes of major melanoma subtypes. *Nature*. 2017; doi: 10.1038/nature22071.

64. Akbani R, Akdemir KC, Aksoy BA, Albert M, Ally A, Amin SB, et al.. Genomic Classification of Cutaneous Melanoma. *Cell*. 2015; doi: 10.1016/j.cell.2015.05.044.

65. Yarchoan M, Gane EJ, Marron TU, Perales-Linares R, Yan J, Cooch N, et al.. Personalized neoantigen vaccine and pembrolizumab in advanced hepatocellular carcinoma: a phase 1/2 trial. *Nat Med*. 2024; doi: 10.1038/s41591-024-02894-y.

66. Biswas N, Chakrabarti S, Padul V, Jones LD, Ashili S. Designing neoantigen cancer vaccines, trials, and outcomes. *Front Immunol*. 2023; doi: 10.3389/fimmu.2023.1105420.

67. Wu D-W, Jia S-P, Xing S-J, Ma H, Wang X, Tang Q-Y, et al.. Personalized neoantigen cancer vaccines: current progression, challenges and a bright future. *Clin Exp Med*. 2024; doi: 10.1007/s10238-024-01436-7.

68. Cai Y, Li D, Lv D, Yu J, Ma Y, Jiang T, et al.. MHC-I-presented non-canonical antigens expand the cancer immunotherapy targets in acute myeloid leukemia. *Sci Data*. 2024; doi: 10.1038/s41597-024-03660-y.

69. Ye L, Creaney J, Redwood A, Robinson B. The Current Lung Cancer Neoantigen Landscape and Implications for Therapy. *Journal of Thoracic Oncology*. 2021; doi: 10.1016/j.jtho.2021.01.1624.

70. Li J, Xiao Z, Wang D, Jia L, Nie S, Zeng X, et al.. The screening, identification, design and clinical application of tumor-specific neoantigens for TCR-T cells. *Mol Cancer*. 2023; doi: 10.1186/s12943-023-01844-5.

71. Buonaguro L, Tagliamonte M. Selecting Target Antigens for Cancer Vaccine Development. *Vaccines*. 2020; doi: 10.3390/vaccines8040615.

72. Cavallo F, Calogero RA, Forni G. Are oncoantigens suitable targets for anti-tumour therapy? *Nat Rev Cancer*. 2007; doi: 10.1038/nrc2208.

73. Jiang P, Wang H, Li W, Zang C, Li B, Wong YJ, et al.. Network analysis of gene essentiality in functional genomics experiments. *Genome Biol*. 2015; doi: 10.1186/s13059-015-0808-9.

74. Jeong H, Mason SP, Barabási A-L, Oltvai ZN. Lethality and centrality in protein networks. *Nature*. 2001; doi: 10.1038/35075138.

75. Ning K, Ng HK, Srihari S, Leong HW, Nesvizhskii AI. Examination of the relationship between essential genes in PPI network and hub proteins in reverse nearest neighbor topology. *BMC Bioinformatics*. 2010; doi: 10.1186/1471-2105-11-505.

76. Crosby EJ, Hartman ZC, Lyerly HK. Beyond Neoantigens: Antigens Derived from Tumor Drivers as Cancer Vaccine Targets. *Clin Cancer Res*. 2023; doi: 10.1158/1078-0432.CCR-23-1244.

77. Azhagesan K, Ravindran B, Raman K. Network-based features enable prediction of essential genes across diverse organisms. Mande SC, editor. *PLoS ONE*. 2018; doi: 10.1371/journal.pone.0208722.

78. Hazini A, Fisher K, Seymour L. Deregulation of HLA-I in cancer and its central importance for immunotherapy. *J Immunother Cancer*. 2021; doi: 10.1136/jitc-2021-002899.

79. Taylor BC, Balko JM. Mechanisms of MHC-I Downregulation and Role in Immunotherapy Response. *Front Immunol*. 2022; doi: 10.3389/fimmu.2022.844866.

80. Zaretsky JM, Garcia-Diaz A, Shin DS, Escuin-Ordinas H, Hugo W, Hu-Lieskovan S, et al.. Mutations Associated with Acquired Resistance to PD-1 Blockade in Melanoma. *N Engl J Med*. 2016; doi: 10.1056/NEJMoa1604958.

81. Merck Sharp & Dohme LLC. A Phase 1, Open-Label, Multicenter Study to Assess the Safety and Tolerability of mRNA-5671/V941 as a Monotherapy and in Combination With Pembrolizumab in Participants With KRAS Mutant Advanced or Metastatic Non-Small Cell Lung Cancer, Colorectal Cancer or Pancreatic Adenocarcinoma. clinicaltrials.gov; 2025 Jan. Report No.: NCT03948763.

82. Zhou Z, Wu J, Ren J, Chen W, Zhao W, Gu X, et al.. TSNAD v2.0: A one-stop software solution for tumor-specific neoantigen detection. *Computational and Structural Biotechnology Journal*. 2021; doi: 10.1016/j.csbj.2021.08.016.

83. Wu J, Wang W, Zhang J, Zhou B, Zhao W, Su Z, et al.. DeepHLApan: A Deep Learning Approach for Neoantigen Prediction Considering Both HLA-Peptide Binding and Immunogenicity. *Front Immunol*. 2019; doi: 10.3389/fimmu.2019.02559.

84. Rieder D, Fotakis G, Ausserhofer M, René G, Paster W, Trajanoski Z, et al.. nextNEOpi: a comprehensive pipeline for computational neoantigen prediction. Valencia A, editor. *Bioinformatics*. 2022; doi: 10.1093/bioinformatics/btab759.

85. Di Tommaso P, Chatzou M, Floden EW, Barja PP, Palumbo E, Notredame C. Nextflow enables reproducible computational workflows. *Nat Biotechnol*. 2017; doi: 10.1038/nbt.3820.

86. Huber F, Arnaud M, Stevenson BJ, Michaux J, Benedetti F, Thevenet J, et al.. A comprehensive proteogenomic pipeline for neoantigen discovery to advance personalized cancer immunotherapy. *Nat Biotechnol*. 2025; doi: 10.1038/s41587-024-02420-y.

87. Diao K, Chen J, Wu T, Wang X, Wang G, Sun X, et al.. Seq2Neo: A Comprehensive Pipeline for Cancer Neoantigen Immunogenicity Prediction. *Int J Mol Sci*. 2022; doi: 10.3390/ijms231911624.

Click here to access/download;Figure;Figure4.pdf

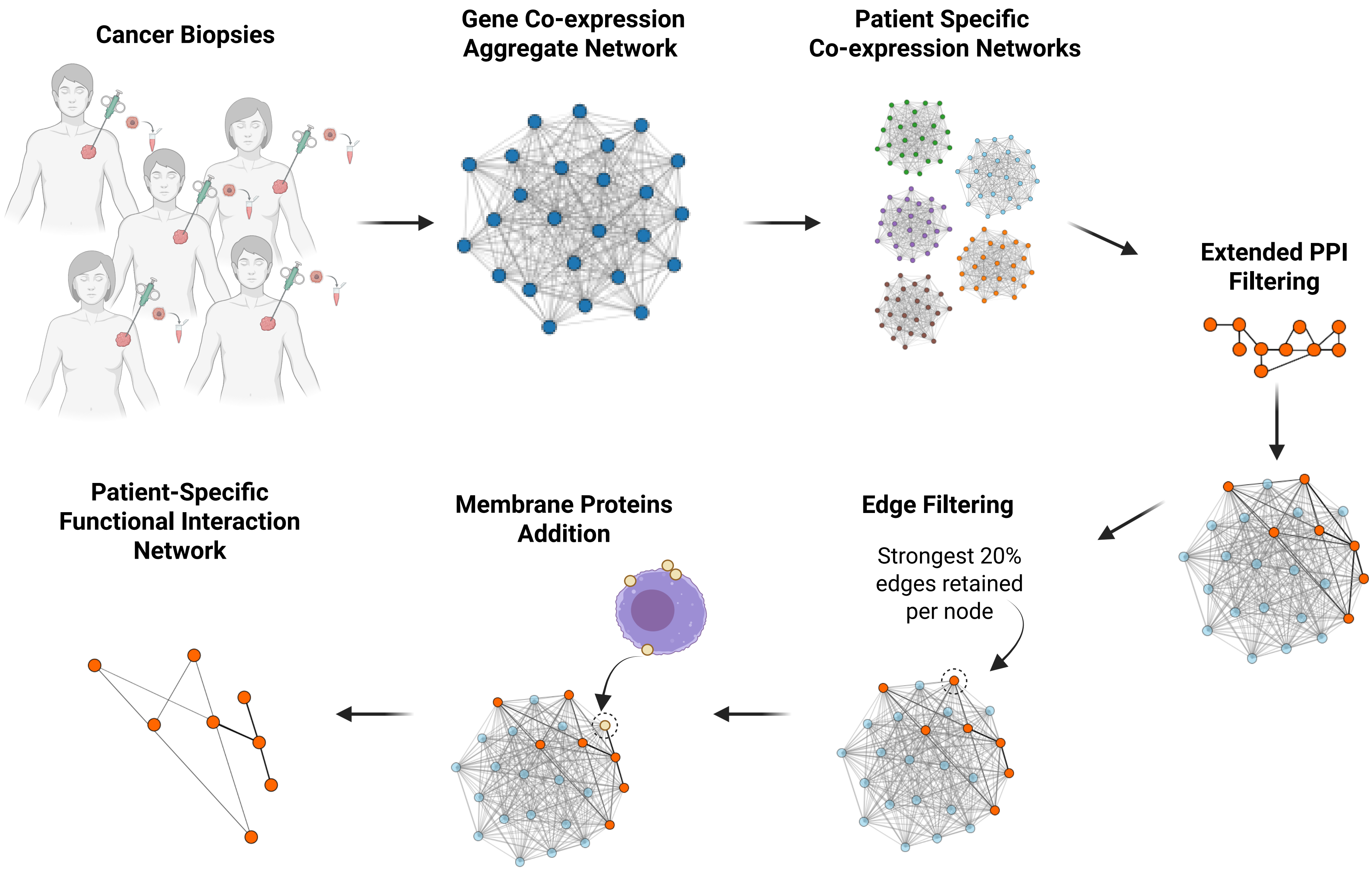

Click here to access/download;Figure;Figure5.pdf

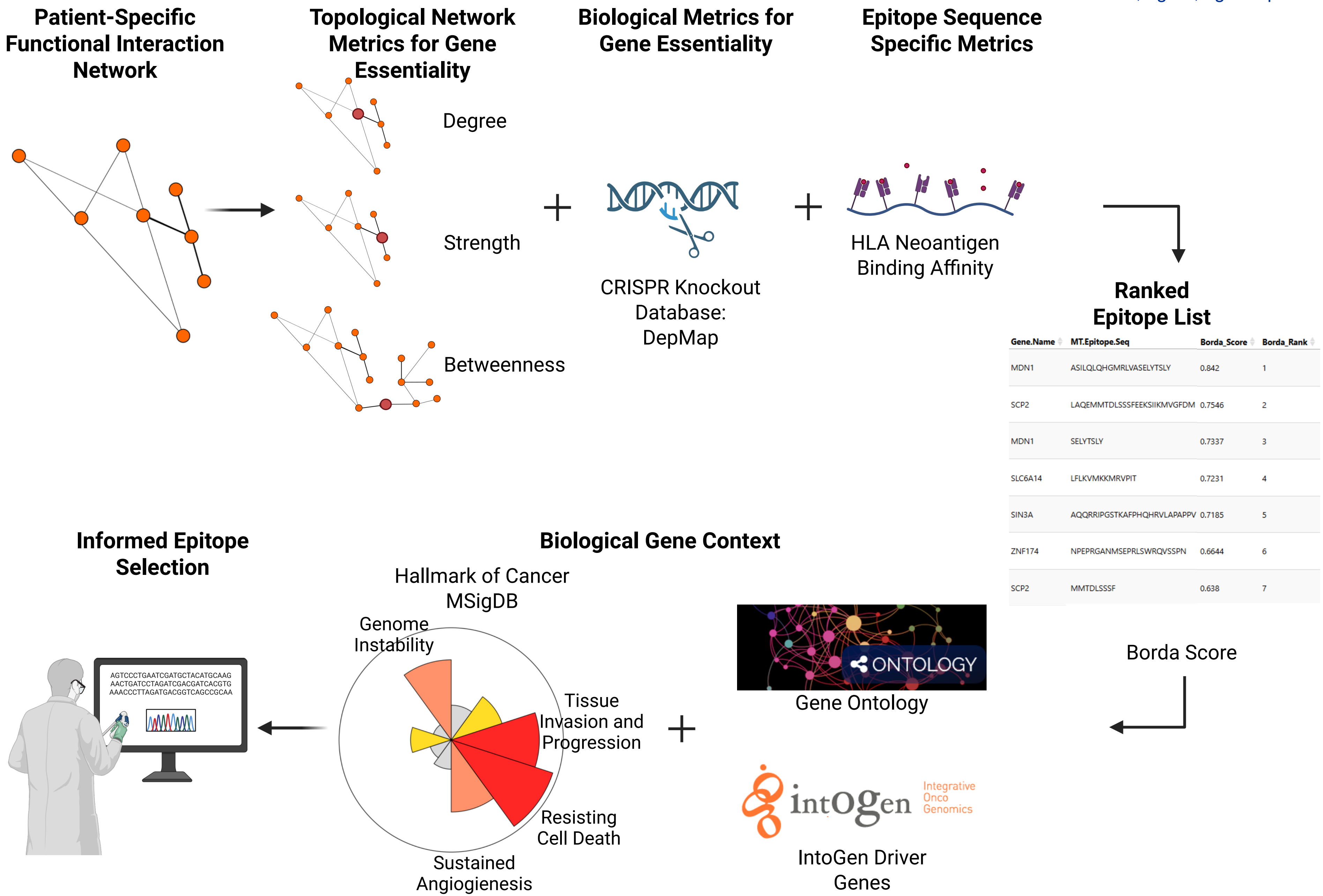

Click here to access/download;Figure;Figure6.pdf

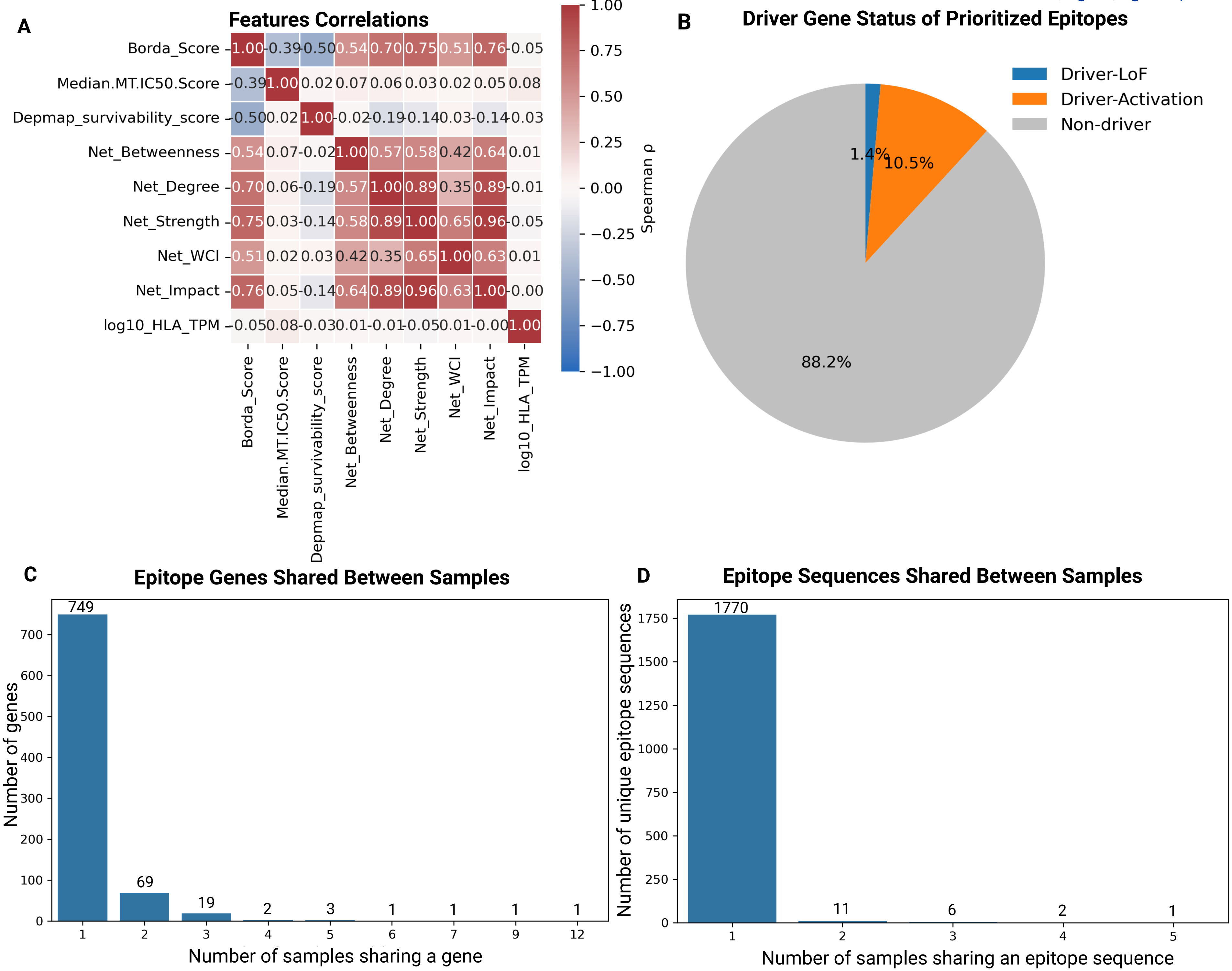

Click here to access/download;Figure;Figure1.pdf

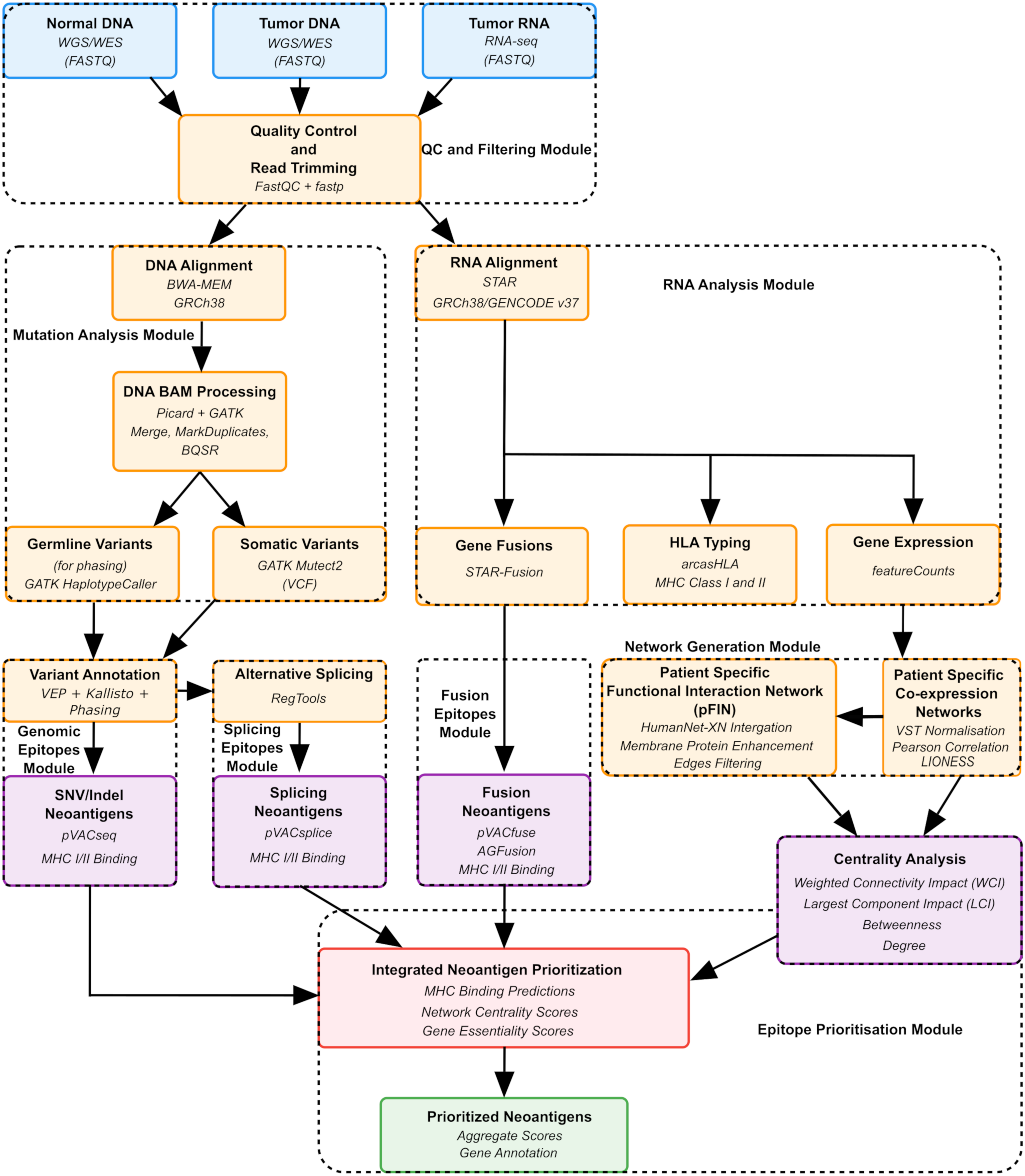

Click here to access/download;Figure;Figure2.pdf

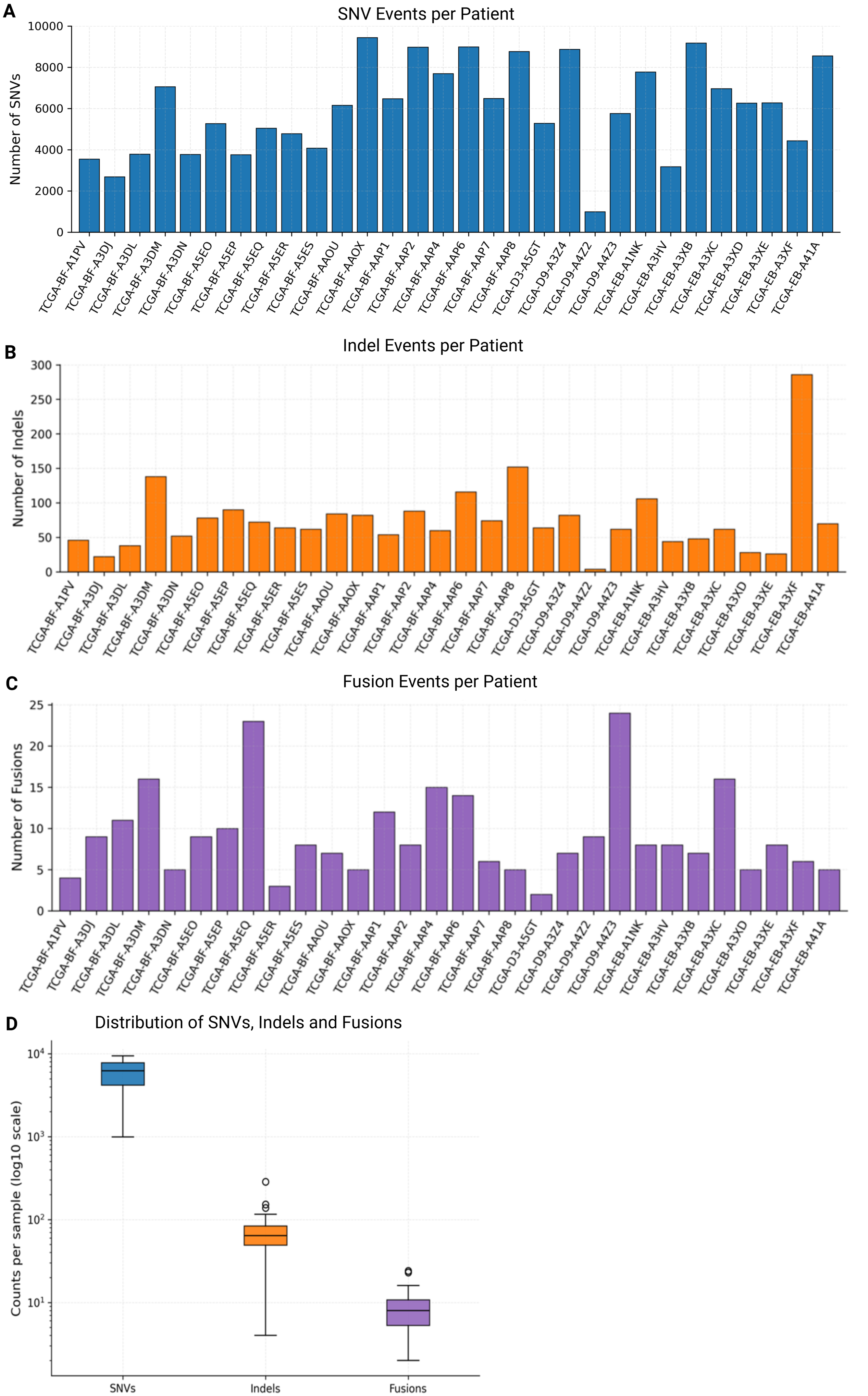

Click here to access/download;Figure;Figure3.pdf

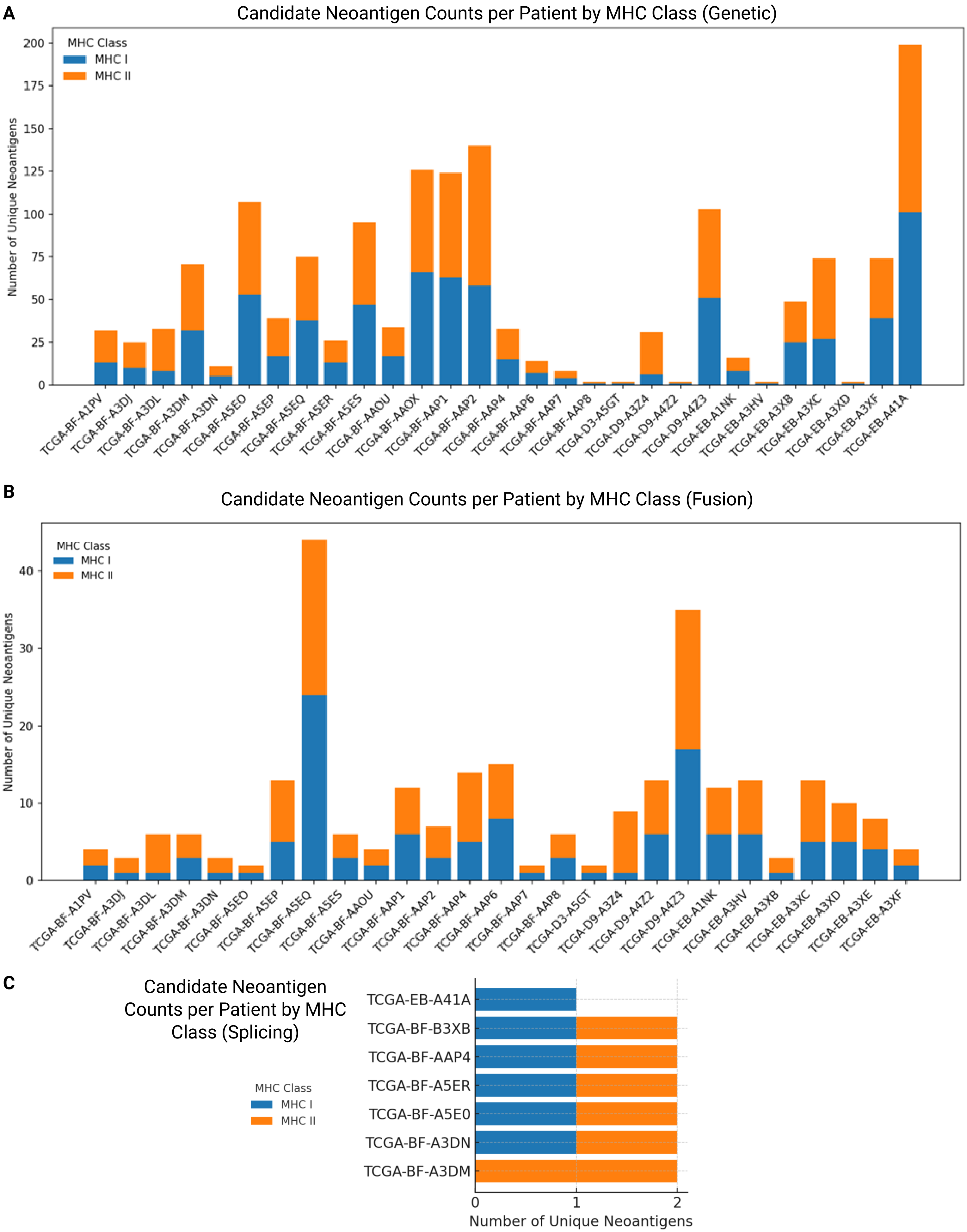

Epitope Prioritisation Tables, one sheet per patient

Click here to access/download
Supplementary Material
Additional file 2_Supplementary Material.xlsx

Click here to access/download
Supplementary Material
Additional file 1_Supplementary Material.docx

Cover Letter Click here to access/download;Personal Cover;Cover Letter.docx

Tampere University

Dr. Xun Xu,
Editor in Chief
GigaScience

January, 22nd 2026

Dear Dr. Xun Xu,

Please find enclosed our manuscript entitled "Epitope Generation Gateway (EGG): A biological context-dependent approach to cancer vaccine development" by Mannino et al., for consideration for publication as a Research Article in *GigaScience*.

Personalized neoantigen discovery has matured in its ability to find candidate epitopes, yet prioritization remains a key bottleneck, particularly when aiming to identify vaccine targets that are resilient to tumor immune escape. In this work, we introduce Epitope Generation Gateway (EGG), a containerized, end-to-end pipeline that streamlines epitope discovery across SNVs, indels, gene fusions, and splicing-derived candidates while explicitly incorporating biological context to prioritize more evolutionary durable targets.

Key advance delivered by Epitope Generation Gateway (EGG) beyond sequence-centric neoantigen pipelines is that it embeds each candidate epitope in patient-specific tumor functional context to support prioritization of targets that are less likely to be eliminated under selective pressure. To achieve this, EGG coordinates widely used, well supported components for core steps (Mutect2 for somatic SNVs/indels, STAR-Fusion for fusion detection, ArcasHLA for HLA typing, and pVACtools for MHC class I/II epitope prediction), and then enhances peptide-level predictions with systems-level evidence derived from the cancer gene expression context. For each patient, EGG reconstructs cancer specific co-expression and protein-protein interaction networks and derives network-informed features (including centrality and perturbation related metrics), integrates CRISPR dependency gene essentiality evidence from DepMap, and annotates candidate source genes using Gene Ontology, Hallmarks of Cancer processes, and IntOGen driver status. These heterogeneous datapoints are integrated into a consensus prioritization using a rank-based procedure, producing an interpretable, ordered epitope list per sample that favors targets less likely to be eliminated under selective pressure. As a case study, we applied EGG to 30 TCGA SKCM patients, demonstrating reproducible execution from raw data inputs to prioritized outputs. Collectively, the results show how embedding neoantigen candidates in patient specific functional context can strengthen prioritization beyond sequence scoring alone.

We believe this manuscript is well aligned with *GigaScience*'s emphasis on open, reproducible, data-driven biomedical research and the linking of publications to reusable research objects such as software and workflows. In keeping with the journal's expectations for transparency and reuse, EGG is openly available and fully documented at: https://github.com/fhaive/epitope_generation_gateway. We also confirm that the manuscript is original, has not been published elsewhere, and is not under consideration by another journal.

Thank you for your consideration. We would be pleased to address any questions from you or the reviewers, and we believe this work will be of strong interest to readers developing computational methods and resources for cancer immunology.

Sincerely,

Dario Greco
PhD, Professor
Tampere University, Faculty of Medicine and Health Technology
