# Supplementary material for: Epitope Generation Gateway: A biological context-dependent approach to cancer vaccine development
Source: Gigascience. 2026 Oct 1;15:giag095. doi: 10.1093/gigascience/giag095 (PMC13679409; doi:10.1093/gigascience/giag095)
Supplement: Supplementary file 5 [file giag095_giga-d-26-00020_revision_2.pdf]

GigaScience

Epitope Generation Gateway (EGG):

A biological context-dependent approach to cancer vaccine development

--Manuscript Draft--

**Manuscript Number:**	GIGA-D-26-00020R2
**Full Title:**	Epitope Generation Gateway (EGG):<br><br>A biological context-dependent approach to cancer vaccine development
**Article Type:**	Research
**Funding Information:**	Jane ja Aatos Erkon Säätiö — Prof Seppo Ylä-Herttuala<br>Prof Vincenzo Cerullo<br>Prof Dario Greco
**Abstract:**	Abstract<br>Background<br>Personalized neoantigen discovery requires tools that not only identify putative epitopes but also prioritize candidates using biological features relevant to tumor fitness and immune escape. Here, we present Epitope Generation Gateway (EGG), a containerized pipeline that streamlines discovery and prioritization across variant types and human leukocyte antigen (HLA) contexts. EGG coordinates established components, Mutect2 for somatic single nucleotide variations (SNVs) and indels, STAR-Fusion for fusion events, ArcasHLA for HLA typing, and pVACtools for epitope sequence prediction, then augments peptide-level scores with biological context. For each patient, EGG builds co-expression and protein-protein interaction networks, uses centrality measures and CRISPR dependency data (DepMap) to flag genes important for tumor fitness, and annotates source genes with Gene Ontology terms, Hallmarks of Cancer processes, and IntOGen driver status.<br>Results<br>This multi-layer information supports ranking of candidates that are predicted to be presented and that arise from genes essential to tumor fitness or embedded in key pathways, providing a framework to nominate epitopes whose source genes may be less dispensable under immune pressure. As a case study, we applied EGG to 30 TCGA skin cutaneous melanoma (SKCM) patients, demonstrating end-to-end execution and producing prioritized epitopes that emphasize peptides and alterations in high-centrality, dependency-supported essential genes.<br>Conclusions<br>Overall, EGG links epitope prioritization to tumor systems biology and evolutionary rationale, prioritizing biologically contextualized cancer vaccine candidates from SNVs, indels, fusions, and splicing-derived epitopes while streamlining dataset analysis. EGG is available from GitHub at https://github.com/fhaive/epitope_generation_gateway, registered in bio.tools (biotoolsID: epitope_generation_gateway; https://bio.tools/epitope_generation_gateway) and SciCrunch (RRID: SCR_028792), and archived as a citable workflow in WorkflowHub (DOI: 10.48546/workflowhub.workflow.2165.2).
**Corresponding Author:**	Dario Greco<br>Tampereen Yliopisto<br>Tampere, FINLAND
**Corresponding Author Secondary Information:**	
**Corresponding Author's Institution:**	Tampereen Yliopisto
**Corresponding Author's Secondary Institution:**	
**First Author:**	Luca Mannino
**First Author Secondary Information:**	

Powered by Editorial Manager® and ProduXion Manager® from Aries Systems Corporation

**Order of Authors:**	Luca Mannino
	Michele Fratello
	Jacopo Chiaro
	Federica D'Alessio
	Emanuele Di Lieto
	Minna Niittykoski
	Angela Serra
	Seppo Ylä-Herttuala
	Vincenzo Cerullo
	Antonio Federico
	Dario Greco
**Order of Authors Secondary Information:**	
**Response to Reviewers:**	Dear Editor and Reviewers,<br>We sincerely thank the Editor and both reviewers for their continued consideration of our manuscript. We are grateful to Reviewer 2 for their positive assessment and to Reviewer 1 for the remaining constructive comments, which helped us further clarify the scope of the study and align our claims more closely with the evidence presented.<br><br>As requested by the Editor, we have registered EGG in bio.tools (biotoolsID: epitope_generation_gateway; https://bio.tools/epitope_generation_gateway) and SciCrunch (RRID: SCR_028792). We have also archived the computational workflow in WorkflowHub (DOI: 10.48546/workflowhub.workflow.2165.2; https://doi.org/10.48546/workflowhub.workflow.2165.2). These identifiers and their corresponding links have been added to the revised manuscript.<br><br>Below, we provide a detailed point-by-point response to Reviewer 1's comments. Revised versions of the manuscript and supplementary information, with all modifications indicated using track changes, have been included with the resubmission. The principal revisions are also summarised in our responses below and can be reviewed in the accompanying "Manuscript with track changes" and "Supplementary Information with track changes" PDF files.<br><br>A file was also added to the submission named Answers to reviewer comments 2.docx which consists of these answers but in a word document so that it might be more readable as it includes the figures.<br>Reviewer reports:<br>Reviewer #1: I thank the authors for a thorough and largely responsive revision. The pipeline is well engineered and the rebuttal resolves most of my methodological concerns. A few points remain, centered on the validation analysis and on scoping the claims to match the evidence.<br><br>1. I'm still having trouble reconciling that presentation and immunogenicity are not durability.<br>The paper's central thesis is that ranking by source-gene essentiality and network centrality yields targets the tumor is less able to silence under immune pressure.<br>However, I think matching against IEDB/CEDAR/TSNAdb, at best, tells us a candidate peptide is more likely to be presented or T-cell-reactive.<br>It says nothing about whether that candidate's source gene is harder to lose during tumor evolution.<br>So the entire durability claim (the reason the network layer exists) is untouched by this analysis.<br>I suggest authors frame the durability advantage as a hypothesis rather than something that has been validated.<br>We agree that presentation and immunogenicity are not equivalent to evolutionary durability. The IEDB/CEDAR/TSNAdb analysis provides support that EGG/Borda-ranked candidates can recover peptides with experimental evidence of presentation and/or immune recognition more effectively than an IC50-only ranking, but it does not

demonstrate that the corresponding source gene are less likely to be lost during tumour evolution.
We have therefore revised the manuscript to downstate the immune escape resistance of the identified neoantigen as a hypothesis rather than as a validated outcome of the present study. Accordingly, we have revised the relevant sections of the manuscript to better distinguish the level of support.
Therefore, we added the following text to the result/discussion section of the manuscript:
"Although EGG is designed to prioritise epitopes derived from genes with greater functional and network relevance, the proposed evolutionary durability of such targets remains a hypothesis in the present study. The external epitope-database analysis supports the potential presentation and immunogenicity of selected candidates, but does not directly test whether their source genes are less likely to be lost or silenced under immune pressure."

2. I'm also wondering if the minimum 8 containment is a good threshold given peptides vary by length. Wouldn't a % match be a better threshold? I fear that some of the result is coming from peptides that are of a longer size and for which 8 amino acid match brings them somewhere very different. Could the authors clarify if this is affected?

We thank the reviewer for raising this important point. The min-8 containment endpoint was intended as a sensitive core-overlap criterion rather than as evidence of full-length peptide identity. We used this approach because exact full-length peptide matching was extremely sparse. Nevertheless, we confirm that peptide length can affect the interpretation of a fixed 8-amino-acid containment threshold, and that a peptide length-normalised analysis provides an important sensitivity check.
To address this, we performed an additional coverage sensitivity analysis, now included in Additional file 3_Supplementary_Material. For each 8amino acids containment match between an EGG candidate peptide and an experimentally supported external reference peptide from CEDAR, IEDB, or TSNAdb v2.0, we calculated the fraction of the longer peptide covered by the matched sequence. We then repeated the analysis using the original min-8 containment endpoint and stricter criteria requiring min-8 containment plus ≥50%, ≥70%, or ≥80% coverage of the longer peptide.
Increasing the required longer-peptide coverage substantially reduced database-specific support. CEDAR retained 542, 189, 40, and 14 candidates; IEDB retained 545, 192, 41, and 14 candidates; and TSNAdb v2.0 retained 5, 1, 0, and 0 candidates across the min-8, ≥50%, ≥70%, and ≥80% thresholds, respectively (Additional file 3_Supplementary_Material, Supplementary Figure S7). We also repeated the ranking comparison using these progressively stricter coverage thresholds. The original relaxed min-8 endpoint produced the clearest recovery signal relative to IC50-only ranking at selective cutoffs, whereas this signal was attenuated under the ≥50% longer-peptide coverage threshold and became sparse at the ≥70% and ≥80% thresholds (Additional file 3_Supplementary_Material, Supplementary Figure S8). These results indicate that the min-8 endpoint is useful for detecting shared experimentally supported peptide cores, but is sensitive to peptide length-normalised stringency.

To help clarify we have added the following paragraph in the supplementary file 3:
"To assess whether the relaxed min-8 containment endpoint was influenced by peptide length, we performed an additional peptide length-normalised coverage sensitivity analysis. For each relaxed containment match between an EGG candidate peptide and an experimentally supported external reference peptide from CEDAR, IEDB, or TSNAdb v2.0, we calculated the fraction of the longer peptide covered by the matched sequence. We then repeated the external-evidence comparison using min-8 containment alone and min-8 containment combined with ≥50%, ≥70%, and ≥80% longer-peptide coverage thresholds.
Increasing the required longer-peptide coverage substantially reduced database-specific support. CEDAR retained 542, 189, 40, and 14 candidates; IEDB retained 545, 192, 41, and 14 candidates; and TSNAdb v2.0 retained 5, 1, 0, and 0 candidates across the min-8, ≥50%, ≥70%, and ≥80% thresholds, respectively (Supplementary Figure S7).
We then repeated the ranking comparison using these progressively stricter coverage

thresholds. The original relaxed min-8 endpoint produced the clearest recovery signal relative to IC50-only ranking at selective cutoffs, whereas this signal was attenuated under the ≥50% longer-peptide coverage threshold and became sparse at the ≥70% and ≥80% thresholds (Supplementary Figure S8). These results indicate that relaxed min-8 containment is useful for detecting shared experimentally supported peptide cores, but that it is sensitive to peptide length-normalised stringency and should not be interpreted as full-length peptide validation.

Supplementary Figure S7. Effect of peptide length-normalised coverage thresholds on database-specific external evidence support. The number of EGG candidates with experimentally supported external evidence is shown for each matching criterion, stratified by evidence database. Candidate support was assessed using the original relaxed min-8 containment endpoint and stricter criteria requiring the matched segment to cover at least 50%, 70%, or 80% of the longer peptide. Counts are shown separately for CEDAR, IEDB, and TSNAdb v2.0. Increasing the coverage threshold substantially reduced the number of supported candidates, indicating that relaxed min-8 containment should be interpreted as sensitive core-level support rather than full-length peptide validation.

Supplementary Figure S8. Effect of peptide length-normalised coverage thresholds on ranking-based recovery of external evidence. Median difference in hit rate between each ranking method and IC50-only ranking is shown across top-N and top-percentile cutoffs within each patient. The analysis used experimentally supported peptide matches pooled across CEDAR, IEDB, and TSNAdb v2.0. The original relaxed min-8 containment endpoint produced the clearest recovery signal relative to IC50-only ranking at selective cutoffs. This signal was attenuated under the ≥50% longer-peptide coverage threshold and became sparse at the ≥70% and ≥80% thresholds, where median differences were zero. These results indicate that the relaxed min-8 endpoint captures shared experimentally supported peptide cores, however stricter full-length-like coverage thresholds leave too few supported candidates for robust ranking comparison.“

3. Is assuming purity =1 a good measure? I wonder if this inflates the VAF interpretation.
Ex: melanomas are 0.3-0.7. Have the authors tested with other purity values?
We agree that tumour purity is an important consideration when interpreting tumour DNA variant allele fraction. We recognise that tumour samples are unlikely to consist exclusively of tumour cells and may also contain adjacent normal tissue and infiltrating immune cells. Nevertheless, assuming purity = 1 is conservative in this context and avoids inflating the inferred clonality. Under a simplified diploid heterozygous model, cancer cell fraction is approximately proportional to 2 × variant allele fraction divided by tumour purity. Therefore, for a fixed observed variant allele fraction, lowering the assumed tumour purity from 1.0 to values such as 0.7, 0.5, or 0.3 would increase, rather than decrease, the inferred cancer cell fraction.
In our analysis, all matched somatic final candidates, including all somatic top-10 candidates, had tumour DNA variant allele fraction ≥ 0.25. Under the conservative purity = 1 assumption, this corresponds approximately to cancer cell fraction ≥ 0.5. Using lower melanoma-relevant purity values would risk yielding variants that are less compatible with clonal or near-clonal status. Thus, additional testing across lower purity values would not change the qualitative conclusion that we did not identify a low-variant-allele-fraction or clearly subclonal subset driving the final prioritisation.
Finally, the relevant purity- and variant-allele-fraction-related parameters are user-configurable in the pVACtools-based component of the EGG workflow, allowing users to apply different thresholds if needed.
We also added this paragraph in the method section to clarify what tumor purity value was used and that in can be modified by the user if needed.
“For tumour-purity-dependent tiering, we retained the pVACseq default tumour purity value of 1.0; this parameter is user-configurable and can be adjusted when sample-specific tumour purity estimates are available. In this case study, retaining tumour purity = 1.0 provided a conservative variant allele frequency based parameter for identifying tumour-relevant somatic candidates.”

Powered by Editorial Manager® and ProduXion Manager® from Aries Systems Corporation

4. Maybe a very minor point:The workflow is described as "lightweight," but the new benchmark reports ~75 GB peak memory for the membrane-enrichment step; the framing should be tempered.

We agree with the reviewer. Our use of “lightweight” was intended to refer to reduced workflow complexity and runtime for the case-study single-caller setup and downstream epitope-prioritisation steps, not to imply that all components have low memory requirements. The benchmark shows that some patient-specific network-generation steps, particularly membrane-edge enrichment, can require substantial RAM. To avoid ambiguity, we have replaced “lightweight” with “streamlined” in the main text and clarified in the supplementary benchmark that the epitope-prioritisation steps are comparatively lightweight only after candidate neoantigen tables and network metrics have been generated. We also now explicitly note that network generation, especially membrane-edge enrichment, represents the main memory-intensive component of the workflow.

Revised supplementary paragraph:
“The epitope prioritisation benchmark was run across the same 30 samples and included candidate merging and IC50 harmonisation, DepMap/IntOGen annotation, addition of network metrics, Borda rank calculation, GO/Hallmark annotation, and generation of interactive HTML outputs. These downstream prioritisation steps were comparatively lightweight once candidate neoantigen tables and network metrics had been generated. Merging epitopes and harmonising IC50 values required a median of 16.0 s per sample, DepMap/IntOGen annotation required 5.3 s, addition of network metrics required 1.85 min, Borda rank calculation required 5.2 s, GO/Hallmark annotation required 36.9 s, and interactive HTML generation required 6.6 s. Peak memory was below approximately 1.3 GB for steps where reliable memory estimates were available, except that some very short rules did not produce stable peak-memory estimates and are therefore reported as not available.
Overall, this benchmark shows that EGG-specific epitope prioritisation is computationally modest after upstream candidate generation and patient-specific network metrics are available. In contrast, the main computational burden is associated with patient-specific network generation, particularly LIONESS reconstruction, PPI filtering, and membrane-edge enrichment; the latter reached approximately 75 GB peak RAM in this benchmark. These results provide practical guidance for adjusting Snakemake concurrency and memory allocation according to available compute resources.”

A few remaining items I noticed in the revised text:
* Figure 3: the body states "up to 2240 epitopes in a single sample" while the Figure 3 caption states "up to 224 epitopes." Please reconcile — this looks like a residual error from the recount.
* A sentence in the Patient2-Specific Networks Methods ("Gene level raw read count matrices previously produced…") is duplicated.
* "Supplementray" is misspelled in several places, including the Additional file 3 description.

We thank the reviewer for identifying these remaining editorial issues. We have revised the text accordingly by reconciling the Figure 3 value between the main text and figure caption, removing the duplicated sentence in the Patient-Specific Networks Methods section, and correcting the misspelling of “Supplementary” throughout the manuscript and supplementary-file descriptions, including the description of Additional file 3.

Final point:
The Abstract and title still state that EGG prioritizes "durable cancer vaccine targets" and reduces "the likelihood of immune escape." These are efficacy claims that the current validations do not establish and so given the appropriately scoped limitation now in the Discussion, please bring the Abstract (and ideally the title framing) into line so the claims match the evidence.

We agree with the reviewer and have revised the Abstract to avoid presenting evolutionary durability or reduced immune escape as validated outcomes of the current

	study. Specifically, we replaced sentences stating that EGG prioritizes candidates "resilient to tumor immune escape," "reduces the likelihood of immune escape," and identifies "durable cancer vaccine targets" with more appropriately scoped wording describing EGG as a framework that integrates peptide-level prediction with gene-level and network-context features relevant to tumour fitness and immune escape.<br><br>We modified the text of the abstract as follows:<br><br>"Abstract<br>Background<br>Personalized neoantigen discovery requires tools that not only identify putative epitopes but also prioritize candidates using biological features relevant to tumor fitness and immune escape. Here, we present Epitope Generation Gateway (EGG), a containerized pipeline that streamlines discovery and prioritization across variant types and human leukocyte antigen (HLA) contexts. EGG coordinates established components, Mutect2 for somatic single nucleotide variations (SNVs) and indels, STAR-Fusion for fusion events, ArcasHLA for HLA typing, and pVACtools for epitope sequence prediction, then augments peptide-level scores with biological context. For each patient, EGG builds co-expression and protein-protein interaction networks, uses centrality measures and CRISPR dependency data (DepMap) to flag genes important for tumor fitness, and annotates source genes with Gene Ontology terms, Hallmarks of Cancer processes, and IntOGen driver status.<br>Results<br>This multi-layer information supports ranking of candidates that are predicted to be presented and that arise from genes essential to tumor fitness or embedded in key pathways, providing a framework to nominate epitopes whose source genes may be less dispensable under immune pressure. As a case study, we applied EGG to 30 TCGA skin cutaneous melanoma (SKCM) patients, demonstrating end-to-end execution and producing prioritized epitopes that emphasize peptides and alterations in high-centrality, dependency-supported essential genes.<br>Conclusions<br>Overall, EGG links epitope prioritization to tumor systems biology and evolutionary rationale, prioritizing biologically contextualized cancer vaccine candidates from SNVs, indels, fusions, and splicing-derived epitopes while streamlining dataset analysis. EGG is available from GitHub at https://github.com/fhaive/epitope_generation_gateway, registered in bio.tools (biotoolsID: epitope_generation_gateway; https://bio.tools/epitope_generation_gateway) and SciCrunch (RRID: SCR_028792), and archived as a citable workflow in WorkflowHub (DOI: 10.48546/workflowhub.workflow.2165.2).<br>"
**Additional Information:**	
**Question**	**Response**
Are you submitting this manuscript to a special series or article collection?	No
**Experimental design and statistics**<br><br>Full details of the experimental design and statistical methods used should be given in the Methods section, as detailed in our Minimum Standards Reporting Checklist. Information essential to interpreting the data presented should be made available in the figure legends.<br><br>Have you included all the information requested in your manuscript?	Yes

Powered by Editorial Manager® and ProduXion Manager® from Aries Systems Corporation

**Resources**<br><br>A description of all resources used, including antibodies, cell lines, animals and software tools, with enough information to allow them to be uniquely identified, should be included in the Methods section. Authors are strongly encouraged to cite Research Resource Identifiers (RRIDs) for antibodies, model organisms and tools, where possible.<br><br>Have you included the information requested as detailed in our Minimum Standards Reporting Checklist?	Yes
**Availability of data and materials**<br><br>All datasets and code on which the conclusions of the paper rely must be either included in your submission or deposited in publicly available repositories (where available and ethically appropriate), referencing such data using a unique identifier in the references and in the “Availability of Data and Materials” section of your manuscript.<br><br>Have you have met the above requirement as detailed in our Minimum Standards Reporting Checklist?	Yes
GigaScience has policies and guidelines in place for the use of generative AI-writing tools such as ChatGPT. If you have used such writing tools to assist with writing the manuscript this must be declared and cited in the text. Authors should not list AI-writing tools and other AI-assisted technologies as an author or co-author and should acknowledge that they are fully responsible for text generated or refined by AI-writing tools.<p><br><br>A summary of use (particularly in the introduction or among methods) needs to be included at the end of the paper, and the outputs should also be included as a	Yes

supplementary file hosted in GigaDB or other open repositories. Please read our guidelines for more information. <p><br><br>By submitting to GigaScience, you are aware of the journal's AI-writing tools policy, and if you have declared use of such tools below, you have acknowledged this where appropriate in your manuscript and have made a summary of use and outputs available. <p><br><b>AI-assisted writing tools have been used in the preparation of this manuscript?	

Manuscript

Click here to access/download;Manuscript;EpitopeGenerationGateway_Man

Epitope Generation Gateway (EGG):

A biological context-dependent approach to cancer vaccine development

Luca Mannino[a], Michele Fratello[a], Jacopo Chiaro[bcde], Federica D'Alessio[bcde], Emanuele Di Lieto[a], Minna Niittykoski[f], Angela Serra[agh], Seppo Ylä-Herttuala[fi], Vincenzo Cerullo[bcdej], Antonio Federico[agh], Dario Greco[ah†]

† Corresponding Author: dario.greco@helsinki.fi

Affiliations:

a: Faculty of Medicine and Health Technology, Tampere University, Tampere, Finland

b: Department of Pharmaceutical Biosciences, University of Helsinki, Faculty of Pharmacy ImmunoViroTherapy Lab, Drug Research Program, Helsinki, Finland

c: Helsinki Institute of Life Science (HiLIFE), University of Helsinki, Helsinki, Finland

d: Translational Immunology Program (TRIMM), Faculty of Medicine Helsinki University, University of Helsinki, Helsinki, Finland

e: Digital Precision Cancer Medicine Flagship (iCAN), University of Helsinki, Helsinki, Finland

f: A.I. Virtanen Institute for Molecular Sciences, University of Eastern Finland, Kuopio, Finland

g: Tampere Institute for Advanced Study, Tampere University, Tampere, Finland

h: Division of Pharmaceutical Biosciences, Faculty of Pharmacy, University of Helsinki, Helsinki, Finland

i: Heart Center and Gene Therapy Unit, Kuopio University Hospital, Kuopio, Finland

j: Department of Molecular Medicine and Medical Biotechnology and CEINGE, Naples University Federico II, Naples, Italy

ORCID:

Luca Mannino: https://orcid.org/0009-0006-2549-2680

Michele Fratello: https://orcid.org/0000-0002-3997-2339

Jacopo Chiaro: https://orcid.org/0000-0001-6577-2691

Emanuele Di Lieto: https://orcid.org/0009-0000-3329-8649

Minna Niittykoski: https://orcid.org/0000-0002-2816-9874

Angela Serra: https://orcid.org/0000-0002-3374-1492

Seppo Ylä-Herttuala: https://orcid.org/0000-0001-7593-2708

Vincenzo Cerullo: https://orcid.org/0000-0003-4901-3796

Antonio Federico: https://orcid.org/0000-0003-2554-9879

Dario Greco: https://orcid.org/0000-0001-9195-9003

Abstract:

Background

Personalized neoantigen discovery requires tools that not only identify putative epitopes but also prioritize candidates using biological features relevant to tumor fitness and immune escape. Here, we present Epitope Generation Gateway (EGG), a containerized pipeline that streamlines discovery and prioritization across variant types and human leukocyte antigen (HLA) contexts. EGG coordinates established components, Mutect2 for somatic single nucleotide variations (SNVs) and indels, STAR-Fusion for fusion events, ArcasHLA for HLA typing, and pVACtools for epitope sequence prediction, then augments peptide-level scores with biological context. For each patient, EGG builds co-expression and protein-protein interaction networks, uses centrality measures and CRISPR dependency data (DepMap) to flag genes important for tumor fitness, and annotates source genes with Gene Ontology terms, Hallmarks of Cancer processes, and IntOGen driver status.

Results

This multi-layer information supports ranking of candidates that are predicted to be presented and that arise from genes essential to tumor fitness or embedded in key pathways, providing a framework to nominate epitopes whose source genes may be less dispensable under immune pressure. As a case study, we applied EGG to 30 TCGA skin cutaneous melanoma (SKCM) patients, demonstrating end-to-end execution and producing prioritized epitopes that emphasize peptides and alterations in high-centrality, dependency-supported essential genes.

Conclusions

Overall, EGG links epitope prioritization to tumor systems biology and evolutionary rationale, prioritizing biologically contextualized cancer vaccine candidates from SNVs, indels, fusions, and splicing-derived epitopes while streamlining dataset analysis. EGG is available from GitHub at https://github.com/fhaive/epitope_generation_gateway.

Introduction

Cancer immunotherapy has emerged as a dominant therapeutic modality in modern oncology, fundamentally transforming treatment paradigms across multiple malignancies. Among the most promising immunotherapeutic approaches, personalized neoantigen vaccines exploit tumor-specific mutations to generate highly targeted immune responses, offering unprecedented precision in cancer treatment [1,2]. Unlike conventional cytotoxic therapies such as chemotherapy and radiotherapy, which broadly target rapidly dividing cells, neoantigen vaccines provide enhanced tumor specificity and may mitigate the systemic toxicity associated with these standard interventions [3].

The therapeutic efficacy of cancer vaccines essentially depends on their capacity to trigger robust immune responses while maintaining tumor specificity, minimizing off-target effects that could compromise patient safety [4]. Current computational pipelines can identify candidate neo-antigens from SNVs, small insertions or deletions (indels), gene fusions, and aberrant splicing events. Early generation tools primarily targeted SNVs and indels with exclusive focus on Major Histocompatibility Complex (MHC) class I ligand prediction, addressing only $CD8^+$ T-cell responses [5–7]. Subsequent frameworks expanded to include gene-fusion breakpoints and now commonly support both class I and class II molecule prediction, engaging both cytotoxic and helper T-cell responses. The most recently developed tools further capture epitopes from aberrant splicing events [8–10]. However, existing approaches largely overlook the functional context of neoantigens, failing to integrate them within their broader transcriptomic landscape or consider the biological significance of their source genes. Moreover, vaccine efficacy is challenged by the tumor intrinsic capacity for immune evasion and the functional importance of targeted mutations. Recent findings by Al Bakir et al. demonstrate that tumors can eliminate immunogenic targets through clonal deletion under selective pressure, emphasizing the critical need to prioritize mutations in functionally indispensable genes [11]. Effective neoantigen selection must therefore consider gene essentiality: alterations in genes essential for malignant fitness or in hub genes anchoring critical signaling pathways are less likely to be purged during tumor evolution, thus representing more durable vaccine targets. Gene co-expression networks provide a practical framework for inferring such functional importance, as network centrality metrics correlate with gene essentiality and the evolutionary cost of mutational escape [12].

Systems biology treats cancer as a dynamic, complex disease, where phenotype arises from perturbed interactions rather than isolated genes. Network models are widespread tools for representing and analyzing complex systems such as tumor biology. Yet, most neoantigen pipelines still emphasize sequence level features for epitope prioritization (such as peptide MHC binding) and give limited weight to the broader context that governs whether a candidate peptide is generated, displayed and retained by the tumor. Recent spatial approaches such as SpatialSNV further illustrate that tumor SNVs can vary across local tumor regions and microenvironmental contexts [13]. Evidence shows that subcellular origin influences both presentation and outcome: neoantigens from plasma membrane proteins are disproportionately presented and have been associated with improved responses to immune checkpoint blockade [14,15]. At the same time, tumors can evade immunity by purging dispensable targets during evolution, underscoring the value of prioritizing alterations in vital genes, which are less likely to be lost [11,16,17].

To address these gaps, we developed Epitope Generation Gateway (EGG), an automated workflow that integrates standard callers and predictors: Mutect2 [18] for somatic SNVs/indels, STAR-Fusion [19] for

fusion events, ArcasHLA [20] for HLA typing, and pVACtools [21–23] for class I/II epitope prediction. EGG augments sequence level scoring with essentiality aware features and places each candidate in its biological setting by building patient specific co-expression/protein-protein-interaction (PPI) networks. For prioritization, EGG integrates multiple layers of evidence regarding the source gene. These include patient specific co-expression/PPI centrality measures as network derived indicators of gene importance or essentiality, complemented by CRISPR knockout based dependency scores from DepMap [24] that provide experimentally grounded, though not patient-specific, gene essentiality information. Candidate genes are further annotated with Gene Ontology [25] terms and MSigDB Cancer Hallmarks [26] gene sets to capture pathway-level context, as well as with IntOGen [27] annotations to identify established cancer driver genes. Taken together, these data-driven and knowledge based features help to prioritize neoantigens that are not only predicted to be presented but also arise from genes whose functions are critical to tumor fitness and therefore less likely to be lost through immune evasion. EGG's design is intentionally holistic: it integrates patient specific networks with curated biological knowledge (GO, MSigDB Cancer Hallmarks, DepMap) and presentation relevant attributes (cell membrane localization). The goal is a bigger-picture view of each epitope linking peptide level scores to tumor systems biology so that selected targets are immunologically plausible, and evolutionarily durable. EGG is available from GitHub [28], registered in bio.tools (biotoolsID: epitope_generation_gateway)[29] and SciCrunch (RRID: SCR_028792)[30], and archived as workflow in WorkflowHub [31].

Material and Methods:

Data Acquisition and Preprocessing

Matched tumor RNA-seq, tumor DNA (WES), and normal DNA samples were retrieved from The Cancer Genome Atlas (TCGA) [32] for 30 patients diagnosed with cutaneous melanoma (TCGA-SKCM study code). Patients were randomly selected from the subset with available primary tumor RNA-seq data, corresponding tumor DNA, and matched normal DNA, explicitly excluding metastatic tissue. Datasets were retrieved using the Genomic Data Commons (GDC) client (gdc-client) [33], leveraging unique patient identifiers (UUIDs).

Sequencing alignment files in BAM format were downloaded and converted to paired-end FASTQ format to facilitate compatibility with the EGG pipeline. Conversion was performed using SAMtools (v1.21) [34,35], which involved: splitting BAM files by read group, sorting reads by name and extracting

paired reads as compressed FASTQ files. These steps ensured the correct input data format for subsequent processing modules.

Read Quality Control and Trimming

Raw sequencing reads from tumor RNA-seq, tumor DNA, and matched normal DNA samples (all in FASTQ format) were processed using FastQC (v0.11.9) [36] and fastp (v 0.23.2) [37] to assess and improve read quality. Quality assessment was performed with FastQC to identify issues such as base quality degradation. Read trimming and filtering were subsequently carried out using fastp, which automatically detected and removed adapter sequences and excluded reads with poor quality metrics. Specifically, reads containing bases with Phred scores below 20 or with more than 30% unqualified bases were discarded. Additionally, reads shorter than 30 base pairs were removed. A sliding window quality trimming approach with a mean quality threshold of 20 was applied. All trimming settings were specified in a centralized YAML configuration file (QC_filtering_config_0.yaml), allowing users to easily adapt the preprocessing pipeline to different requirements.

RNA-Seq Processing, Fusion Detection and HLA typing

Trimmed cancer RNA-seq reads were processed using STAR-Fusion (v1.15.0) [19], which aligned reads and detected gene fusion events using the GRCh38 / GENCODE v37 CTAT genome reference [38,39]. This tool generated both fusion transcript calls and alignment files for downstream quantification. Reference genomes and transcript annotations (GRCh38 / GENCODE v37) were acquired from the CTAT genome library provided by STAR-Fusion [40]. These resources were automatically retrieved and managed by the pipeline. STAR-Fusion was used with default filters, which require at least one junction read and one spanning read (or ≥2 junction reads if no spanning support) and a minimum expression support of FFPM (fusion fragments per million total reads) ≥0.1.

Gene expression quantification was performed with the featureCounts function from the Rsubread Bioconductor package (v2.0.8) [41,42], using the same annotation reference to compute raw read counts for each gene in paired-end mode. These count matrices served as input for sample specific co-expression network production implemented in later stages of the pipeline.

To enable personalized neoepitope prediction, HLA typing was conducted using arcasHLA (v0.5) [20], which genotypes both MHC class I and class II loci from RNA-seq data. After sorting the aligned BAM files with SAMtools (v1.21) [34,35], unmapped reads were extracted and used to predict HLA alleles. The output was converted into a format compatible with pVACtools to support downstream MHC binding predictions.

Both STAR-Fusion and arcasHLA were executed within Docker containers, ensuring reproducibility and consistency across environments. Other steps, including featureCounts and BAM sorting, were integrated into the Snakemake [43] pipeline through dedicated Conda environments.

Fusion Epitopes Prediction

Post-alignment RNA fusion calls were processed for neoepitope prediction using the pVACfuse module of pVACtools (v5.3) [22], which integrates gene fusion annotations with HLA typing and MHC binding prediction. Fusion predictions, generated by STAR-Fusion, were first annotated using AGFusion (v1.4.3) [44] with the Ensembl GRCh38 release 103 transcript database [45] and Pfam-A domain annotations [46]. AGFusion resolved coding fusion junctions and transcript models, generating protein sequences suitable for downstream analysis.

These annotated fusion sequences were then passed to pVACfuse, which identified candidate fusion-derived neoantigens using a user-defined panel of MHC class I and II binding prediction algorithms (MHCNuggetsI and MHCnuggetsII) [47]. HLA alleles were automatically parsed from ArcasHLA outputs however in the practical use of the pipeline they can be optionally provided as pre-existent files if the user wants to calculate them using different methods. Parameters such as epitope length (e1, e2), binding affinity thresholds, and the number of prediction threads were specified in the Snakemake pipeline and can be modified by the user by using different pVACtools options including different prediction algorithms.

All fusion epitope analyses were executed within Docker containers to ensure version consistency and reproducibility across environments. The containerized pipeline streamlined fusion annotation, neoantigen calling, and intermediate file handling, enabling high-throughput and customizable analysis per sample.

SNV and Indel Detection in Tumor and Normal DNA Samples

All genomic analyses were conducted using the GRCh38 reference genome [39]. The reference FASTA file, its associated dictionary, and BWA index files were downloaded from the UCSC Genome Browser [48] and generated using SAMtools (v1.16), Genome Analysis Toolkit (GATK, v4.5.0.0) [49,50] CreateSequenceDictionary, and BWA index [51], respectively. Additional resources used in variant calling included a dbSNP [52] VCF (Broad Institute, dbSNP build 138), the gnomAD allele frequency panel [53], and the GATK-provided Panel of Normals (PON). These files were fetched from Broad Institute cloud repositories [54] and indexed with GATK and tabix [55] as needed.

Trimmed DNA-seq reads were processed following the GATK best practices pipeline for short variant discovery. Reads were aligned to the GRCh38 reference genome using BWA-MEM (v0.7.17) [56], with

read group headers automatically extracted from the FASTQ file headers to include metadata including flowcell ID, lane, and barcode. These values were parsed directly from the first line of each FASTQ file to populate the read group (RG) tags used in alignment. The resulting alignments were sorted by coordinate using SAMtools.

To handle multi lane sequencing data, lane specific BAM files for each sample were merged using Picard (v2.27.4) MergeSamFiles [57] Duplicate reads were marked .using GATK MarkDuplicatesSpark to mitigate PCR induced biases. Base quality scores were recalibrated using BaseRecalibrator and ApplyBQSR with known variant sites from dbSNP as training data. These steps produced high-confidence, analysis-ready BAM files per sample.

Alignment metrics, including overall mapping quality and insert size distribution, were collected using GATK CollectAlignmentSummaryMetrics and CollectInsertSizeMetrics respectively. BAM files were indexed using SAMtools to enable efficient access during variant calling and downstream analysis. This preprocessing pipeline was applied to tumor and matched normal DNA samples for each patient, ensuring consistency and compatibility for downstream somatic variant calling with tools such as Mutect2 and HaplotypeCaller.

Germline variants were called on normal DNA samples using GATK HaplotypeCaller, which performs local de novo assembly of haplotypes in regions showing evidence of variation. Raw variant calls were separated into SNPs and indels, followed by hard filtering based on standard GATK quality metrics (e.g., QD, FS, MQ, SOR). Filter-passing variants were selected, sorted, compressed, and concatenated into a single filtered germline VCF file per sample using BCFtools (v1.16) [34] and tabix utilities. These high-confidence germline calls were retained for downstream annotation and comparison against somatic variants.

Somatic variants were identified using GATK Mutect2 by jointly analyzing tumor and matched-normal BAM files per sample. The analysis incorporated a germline resource (gnomAD) and a panel of normals to reduce false positives. Mutect2 output included both raw variant calls and strand orientation artifact data (F1R2).

To refine variant calls, contamination estimates were calculated using GATK GetPileupSummaries and CalculateContamination, followed by orientation bias modeling with LearnReadOrientationModel. Final variant filtering was carried out using FilterMutectCalls, integrating all calculated metrics as additional flags in the VCF for each sample.

Genomic Variant Annotation and Epitope Prediction

To enable downstream neoepitope prediction using pVACtools, we performed additional annotation and preprocessing of somatic and germline variant calls. Variant Effect Predictor (VEP, Ensembl release 103) [58] was used to functionally annotate VCF files from somatic and germline variant calling workflows. VEP was configured in offline mode using the GRCh38 cache and a subset of plugins (e.g., Frameshift, Wildtype) downloaded from the Ensembl GitHub repository. Annotations were run via command-line within a Docker environment using the griffithlab/pvactools container (v5.3). The pipeline was configured to automatically pull the Docker image if not already present. Somatic VCFs (from Mutect2) and filtered germline VCFs (from HaplotypeCaller) were annotated with VEP, producing functionally enriched VCF files for subsequent pVACseq input. While downstream neoepitope analysis focused on somatic mutations, filtered germline variants were obtained to support phasing. These variants were merged with somatic calls and jointly phased using GATK ReadBackedPhasing. This step allowed identification of compound heterozygous or cis/trans configured variants that could influence peptide sequences derived from cancer genomes.

In parallel, gene expression levels were quantified from tumor RNA-seq data using Kallisto (v0.46.1) [59], a lightweight pseudoalignment-based transcript quantifier. A Kallisto index was built from Ensembl cDNA reference transcripts (release 103), and tumor RNA reads from multiple sequencing lanes were jointly quantified. The resulting expression data was integrated into the annotated somatic VCF using vcf-expression-annotator (vatools package v5.2.0), aligning expression values with corresponding variants.

To generate VCFs compatible with pVACtools, tumor-only variants were extracted using GATK SelectVariants. To enable inclusion of germline variants in neoepitope screening, sample identifiers in the germline VCFs were harmonized with the tumor VCF using BCFtools (v1.17). Germline and tumor variants were then merged using GATK CombineVariants and sorted using Picard SortVcf. These combined VCFs were phased using GATK ReadBackedPhasing and re-annotated with VEP to account for phasing effects on coding consequence.

Human leukocyte antigen (HLA) types were obtained via ArcasHLA as described earlier. HLA types were automatically extracted from ArcasHLA genotype JSON files and formatted into a pVACtools compatible input structure. Alternatively, users have the option to supply their own HLA typing externally.

The primary pVACseq analysis was then performed using the pvacseq run command inside the griffithlab/pVACtools Docker container (v5.3). The workflow supported all pVACseq-compatible MHC binding prediction algorithms, and for this analysis specifically used MHC_nuggetsI, and MHCnuggetsII. Both phased variant calls and Kallisto derived RNA expression data were supplied to

enhance neoepitope prioritization. All pVACseq parameters are user configurable by modifying the Snakemake script directly. For this analysis, we used the following key parameters: --epitope-lengths 8-15 for class I predictions and --epitope-lengths 11-30 for class II, along with --depth 1000, and --phased-proximal-variants-vcf to incorporate phasing effects. For tumour-purity-dependent tiering, we retained the pVACseq default tumour purity value of 1.0; this parameter is user-configurable and can be adjusted when sample-specific tumour purity estimates are available. In this case study, retaining tumour purity = 1.0 provided a conservative variant allele frequency based parameter for identifying tumour-relevant somatic candidates. Somatic SNV and indel calls were generated with Mutect2 followed by Mutect2 post-filtering. For the proof-of-concept analysis presented here, both PASS and non-PASS Mutect2 records were retained as pVACseq input to preserve a broad candidate mutation pool before downstream prioritisation. To assess the impact of this choice, we annotated final somatic epitope candidates with the VCF FILTER status of the originating variant and repeated the prioritisation using PASS-only somatic pVACseq inputs. This sensitivity analysis showed that non-PASS calls contributed minimally to the final prioritised candidates and did not materially affect the prioritised gene set. PASS-only filtering can be applied before pVACseq when a higher-specificity input set is desired. Full details of the PASS-only sensitivity analysis are provided in Additional file 3.Mutect2 and STAR-Fusion were selected as pragmatic single-caller defaults for somatic SNV/indel and fusion detection, respectively. This choice was intended to keep the case-study workflow lightweight, reproducible, and computationally tractable, while relying on established tools with broad use in cancer genomics. Mutect2 is used in the GATK somatic short-variant discovery workflow for SNV and indel detection, and is widely regarded as an established, reliable somatic variant caller in cancer genomics [18,60]. STAR-Fusion is used in the NCI Genomic Data Commons RNA-seq harmonization workflow for transcript-fusion detection and has been reported among the most accurate and fastest fusion callers in benchmark analyses of cancer transcriptomes[61]. We acknowledge that consensus calling strategies can further improve input precision and reduce dependence on caller-specific thresholds; however, benchmarking alternative caller combinations was outside the scope of the present study, which focuses on evaluating EGG as an integrated neoantigen prediction and prioritisation framework.

Alternative Splicing-Derived Neoepitope Prediction

Tumor-specific alternative splicing events were identified and processed for neoepitope prediction using a combination of RegTools (v1.0.0) [62] and pVACsplice from the pVACtools suite. First, splice junction annotations were extracted from tumor RNA-seq alignments using RegTools, which identifies novel splice sites and exon-skipping events by comparing observed junctions against reference transcript models.

The analysis was based on the GRCh38/GENCODE v37 reference annotation to identify putative tumor-specific splicing variants. Junction files obtained in output were filtered to retain high-confidence events based on read support thresholds and junction quality metrics.

Identified splicing events were then exploited for neoepitope prediction using pVACsplice (from package pVACtools v5.3), which generates peptide sequences spanning novel exon-exon junctions and predicts their MHC binding potential. The workflow required both the RegTools junction output and the corresponding genomic VCF file containing somatic variants to account for potential interactions between splicing alterations and point mutations.

HLA alleles were provided either from ArcasHLA outputs or user-specified files, maintaining consistency with other neoepitope prediction modules. pVACsplice analysis employed the same MHC binding prediction algorithms used in pVACseq (MHCnuggetsI and MHCnuggetsII) to ensure comparable scoring across different neoantigen sources. Epitope lengths were set to 8-15 amino acids for MHC class I and 11-30 for MHC class II predictions.

All splicing-derived neoepitope analyses were executed within the griffithlab/pvactools Docker container to maintain version consistency and reproducibility. Parameters including minimum junction read support, binding affinity thresholds, and prediction algorithms were configurable through the centralized Snakemake workflow.

Patient Specific Networks Generation

Gene level raw read count matrices previously produced in the RNA-seq processing step (described in the RNA-Seq Processing, Fusion Detection and HLA typing paragraph) were carried forward for patient-specific co-expression network generation. Genes were retained for network construction only if they had at least 15 raw reads in at least 20% of samples; all other genes were removed as a low-expression preprocessing step (in our cohort of 30 samples, this corresponds to requiring detectable expression in at least 6 samples). This filtering step was applied to reduce noise from genes with very low or infrequent expression, which are less likely to support stable correlation estimates in co-expression-based network inference. similar low-expression filtering steps are commonly used in RNA-seq network analysis workflows, including LIONESS-based analyses of TCGA and GTEx data [63,64].

For downstream network analysis, variance stabilizing transformation (VST) was applied to the expression count using the DESeq2 package (v1.46.0) [65]. This transformation adjusts for the strong relationship between gene expression level and variability, producing a matrix in which genes with different expression ranges can be more reliably compared. The resulting normalized expression matrix was used to construct patient-specific co-expression networks.

To estimate individual level gene co-expression patterns, we applied the LIONESS (Linear Interpolation to Obtain Network Estimates for Single Samples) framework [66]. For a set of S samples, the aggregate network was computed using Pearson correlation between all gene pairs (i, j) across the full expression matrix.

To estimate the network specific to a sample q, we first denote the aggregate network computed across all samples as $e_{ij}^{(\alpha)}$, and the corresponding leave-one-out aggregate network excluding sample $q$as $e_{ij}^{(\alpha-q)}$The individual sample network $e_{ij}^{(q)}$ was then derived using the LIONESS formula:

$$e_{ij}^{(q)} = S\left(e_{ij}^{(\alpha)} - e_{ij}^{(\alpha-q)}\right) + e_{ij}^{(\alpha-q)}$$

This equation quantifies the contribution of sample q. to each edge (i, j) by interpolating its influence relative to the full dataset. All sample-specific adjacency matrices were stored in sparse matrix format to facilitate downstream graph based analyses.

Following LIONESS based reconstruction, we filtered the sample-specific networks to prioritize biologically interpretable and functionally relevant edges. First, we intersected each network with an extended PPI network the HumanNet-XN v2, an integrated human functional interaction network anchored in protein-protein interactions and extended with co-expression, pathway, interolog and co-citation evidence [67]. HumanNet edges were mapped from Entrez to Ensembl gene identifiers using the org.Hs.eg.db library (v3.18.0) [68].

To construct the filtered networks, we applied a two-step edge selection strategy that combined the extended PPI informed filtering with membrane-focused expansion:

1. PPI-informed edge selection: For each gene, we retain edges with absolute correlation weights ranking in the top 20% among all edges connecting that gene to its PPI-linked neighbors. This approach prioritized patient-specific co-expression patterns that aligned with known protein-protein interactions.
2. Membrane-enriched network expansion: To supplement the PPI-filtered network, we incorporated additional edges from the gene co-expression network that involved membrane-associated genes. In the default configuration, candidate membrane-associated edges were defined as edges in which at least one endpoint gene was annotated as membrane associated. These candidate edges were ranked by absolute correlation weight, and the top-ranked edges were added to the PPI-filtered network. The number of membrane-associated edges added was controlled by a configurable proportion, set to 5% by default. In the analysis presented

here, this proportion was calculated relative to the number of weighted HumanNet/PPI edges before the per-gene top-20% PPI filtering step. This strategy enriched the network for membrane localized genes while maintaining focus on the strongest co-expression relationships. Membrane associated genes were identified using Gene Ontology (GO) annotations from the following terms and their descendant terms in the GO hierarchy:

- GO:0005886 (*plasma membrane*)
- GO:0005887 (*integral component of plasma membrane*)
- GO:0046658 (*anchored component of membrane*)
- GO:0009897 (*external side of plasma membrane*)
- GO:0009986 (*cell surface*)

Membrane gene annotations were obtained from the GAF file provided by the GO Consortium and mapped to Ensembl IDs via UniProt [69] and BioMart (v2.58.0) [70].

Both the PPI informed edge selection and the membrane-enriched network expansion are optional; the corresponding percentages can be modified via the configuration files or effectively switched off. The final output consisted of sparse adjacency matrices representing filtered, patient-specific co-expression networks enriched for interactions with known biological support and immunological relevance. We have named these networks: Patient Specific Functional Interaction Networks (pFINs) These network matrices were saved in Matrix Market format to ensure compatibility with external graph analysis frameworks.

The pFINs exhibited a characteristic topology consisting of a dominant largest connected component (LCC) alongside smaller, isolated subnetworks. The LCC encompassed the vast majority of edges and nodes, forming a cohesive structure where genes were interconnected through direct or indirect relationships. The remaining nodes formed small, disconnected clusters or isolated pairs that did not connect to the main component. This topology, with most network connectivity concentrated in a single large component, provided a natural framework for assessing gene importance: genes critical to network integrity would be those whose removal disrupted the LCC structure most severely.

Centrality Metrics for Gene Prioritization

To evaluate the functional relevance and network prominence of candidate neoantigen source genes, we calculated node level centrality scores within each sample-specific co-expression network.

To compare the relative connectivity of genes, we computed the Weighted Connectivity Impact (WCI) for each node *v*, defined as the fraction of total network edge weights attributable to its immediate connections.

Where each edge is normalized as:

$$\widetilde{w_{uv}} = \frac{|w_{uv}|}{\max_{i,j}|w_{ij}|}$$

The numerator is the sum of normalized absolute weights of all edges incident to *v*:

$$\text{strength}(v) = \sum_{u \in N(v)} \widetilde{w_{uv}}$$

Weighted Connectivity Impact calculated as:

$$\text{WCI}(v) = \frac{\text{strength}(v)}{\sum_{e \in E} \widetilde{w_e}}$$

This yields a sample-scaled centrality score between 0 and 1, allowing for cross-sample comparisons of gene connectivity despite differences in total edge weight. Genes with high WCI constitute a disproportionately large proportion of overall network strength.

pFINs Centrality Metrics

To assess the topological relevance of neoantigen-associated genes within the pFINs networks, we computed several centrality metrics.

The first centrality metric is the Largest Component Impact (LCI) quantifies a gene's contribution to the integrity of the network's Largest Connected Component (LCC) obtained from the PPI filtering steps explained above. It is a simple way to combine strength and degree in a comparable way between different networks. For each gene *v*, the LCI was computed by measuring the change in both node connectivity and edge weight upon virtual deletion of that gene:

$$\text{Largest Component Impact}(v) = \frac{1}{2}\left[\frac{S_0 - S_v}{S_0}\right] + \frac{1}{2}\left[\frac{W_0 - W_v}{W_0}\right]$$

Where:

- S_0 is the number of nodes in the LCC of the original network,
- $S_v$is the size of the LCC after removing node *v*,

- W_0 is the sum of normalized absolute edge weights within the original LCC,
- W_v is the corresponding sum after removing *v*.

This metric captures both structural fragmentation and, in turn, functional disconnection, identifying nodes whose removal disproportionately disrupts network integrity. Conceptually, LCI relates to percolation theory, where the removal of critical nodes can trigger a phase transition that fragments a network into disconnected components. By quantifying the impact of individual gene removals on the LCC, LCI identifies genes that act as critical connectors maintaining network cohesion.

The second centrality metric computed was betweenness centrality, a dynamic node centrality measure that reflects how information propagates through the networks. In addition, we calculated node degree and weighted strength for each gene, with strength defined as the sum of the absolute weights of its incident edges using igraph (v2.0.3) [71].

Pan-cancer Gene Essentiality Scoring

To incorporate gene biological relevance into neoantigen prioritization, we obtained pan-cancer essentiality scores from the DepMap Public 24Q4 dataset [24]. The CRISPR gene effect matrix (CRISPRGeneEffect.csv) was downloaded from the Broad DepMap Figshare repository (article ID 27993248) and processed in R. This matrix contains Chronos-derived gene effect values that represent copy number corrected knockout fitness effects across cancer cell lines, where more negative values indicate greater cellular dependency [72].

For each gene, we computed the median Chronos gene effect across all available cell lines to derive a single pan-cancer essentiality score (PanCancer_Median_Chronos). Following DepMap guidelines, genes with scores < -0.5 indicate depletion in most cell lines, while scores < -1.0 represent strong essentiality. These pan-cancer essentiality scores were subsequently integrated into the epitopes prioritization workflow to favor epitopes derived from functionally necessary genes.

Cancer Driver Gene Annotation

To identify neoantigens derived from cancer driver genes, we obtained driver annotations from the IntOGen Drivers compendium (version 20230531) [27]. The compendium file was processed to retain all genes classified as either “activating” (oncogenic; Act) or “loss-of-function" (tumor suppressor; LoF) roles across cancer types. This annotation was integrated into the neoantigen prioritization framework to identify neoantigens originating from genes with established roles in cancer pathogenesis, as mutations in driver genes may be particularly relevant for immune targeting strategies.

Consensus Prioritization

Candidate neoantigens were prioritized using a weighted Borda rank aggregation implemented in R. For each feature, epitopes were ranked according to expected directionality: ascending for Median.MT.IC50.Score and Depmap_survivability_score, and descending for network metrics (Network_Betweenness, Network_Degree, Network_Impact, Network_Strength, Network_WCI). Feature-specific ranks were converted to Borda points and combined using predefined weights (default: 0.25 each for Median.MT.IC50.Score and Depmap_survivability_score; 0.10 for each network metric). The aggregate Borda score determined the final ordering, with ties resolved by average rank. Missing feature values were ignored and remaining weights renormalized per epitope. The prioritization (features, weights, rank direction, tie rule, and NA handling) is configurable via a YAML file without code changes, allowing emphasis on binding affinity, network centrality, or gene essentiality as needed.

Results and Discussion:

EGG analytical flow

The complexity of cancer immunotherapy requires sophisticated computational approaches that can identify not just any neoantigens, but those most likely able to remain resistant to evolutionary changes in the cancer. To address this challenge, we developed a comprehensive pipeline that integrates both established algorithms to extrapolate tumor specific neoantigens and novel network-based methodologies to improve neoantigen prioritization based on their biological context. On one hand, our pipeline includes quality control, preprocessing, extensive mutational analysis, transcriptomics data analysis with identification of fusion events, alternative splicing, and HLA typing. The additional value in this study is that we developed novel network-based methods to infer the biological context of the identified neoantigens. Such methods include the inference, manipulation and analysis of sample specific molecular networks. We envision that by integrating data driven interactional properties of network models with prior knowledge from multiple public databases, it is possible to achieve a robust and informed prioritization that improves efficacy and streamlines pre-clinical pipelines.

The EGG pipeline comprises seven modules ranging from quality control and data preprocessing to interpretable ranking of neoantigens. The diagram in Figure 1 provides a detailed overview of the pipeline structure as well as the included tools. The first module, Module 0, is dedicated to quality control and filtering of the raw sequencing reads using fastp [37] for read trimming and fastqc for quality assessment. The subsequent mutation detection module (Module 1A) analyzes paired tumor-normal DNA samples, utilizing Mutect2 [18] for somatic variant identification and HaplotypeCaller [49] for germline variant detection Module 1B, on the other hand, processes trimmed RNA-seq cancer data

through three analytical pathways: STAR-Fusion [19] identifies structural variants, arcasHLA [20] determines patient-specific HLA allotypes, and FeatureCounts (from Rsubread) [41] generates expression profiles which are then used in network-based downstream analysis. Somatic variants identified in Module 1A undergo allele-specific epitope prediction in Module 2A, where the pVACseq [23] framework evaluates SNVs and indels for MHC-binding potential. This module also performs preprocessing steps required by pVACseq, including quantification of transcripts with Kallisto [59] and phasing variants by integrating somatic and germline calls to preserve haplotype context. Fusion transcripts detected by STAR-Fusion are analyzed in Module 2B where pVACfuse [22] is used to predict neo-antigens spanning fusion junctions.

Module 2C extracts alternative splicing events by validating variants occurring at splicing sites obtained from Module 1A, via Mutect2, by applying RegTools [62] identifying tumor-specific splicing events, and subsequently processing these through pVACsplice [21] to predict splice junction derived epitopes.

Finally, the single-sample network module constructs personalized co-expression networks using the LIONESS [66] methodology on Pearson gene co-expression matrices derived from the RNA-seq counts generated in Module 1B. The resulting networks are filtered using HumanNet-XN [67] protein-protein interaction data, and cellular membrane proteins are identified via Gene Ontology [25]. Network centrality metrics are computed for both filtered and unfiltered patient-specific networks and are used, together with relevant biological information, to prioritize candidates with high potential therapeutic relevance. The network related calculation and generation is carried out by the network generation module while the final prioritization is carried out by the epitope prioritization module. Additional information for the execution of the pipeline, including details on the implementation (e.g., Snakemake workflow [43]), retrieval of required references and tools, and execution of individual modules, is provided in Additional file 1 (Supplementary Material). Additional file 1 also includes an overview of the main third-party tools integrated in EGG and a computational resource benchmark of the main EGG-specific components, focusing on patient-specific network generation and epitope prioritisation rather than upstream third-party tools whose resource requirements depend strongly on sequencing depth, library size, and mutation burden.

Figure 1. Epitope Generation Gateway Workflow. The EGG pipeline processes matched normal/tumor DNA (WGS/WES) and tumor RNA-seq data through seven integrated modules. Following quality control, DNA undergoes BWA-MEM [56] alignment and GATK processing for germline and somatic variant calling. RNA processing includes STAR alignment, gene expression quantification, fusion detection (STAR-Fusion), and alternative splicing analysis. HLA typing and variant annotation are performed in parallel. Sample-specific co-expression networks are constructed using filtered HumanNet-XN and LIONESS methodology. Neoantigen prediction targets three categories: SNV/indel, fusion, and splicing neoantigens, all with MHC class I/II binding predictions. Network centrality analysis computes connectivity metrics for integrated prioritization combining MHC binding, centrality scores,

and immunogenicity to generate prioritized neoantigens with biological context. Dashed boxes represent pipeline modules subdivision. Color scheme: blue boxes represent input data, orange boxes represent intermediate analyses or results, purple boxes denote analyses whose outputs contribute to the consensus scoring, the red box represents the consensus prioritization step, and the green box indicates the final output of prioritized epitope tables.

Inter sample variability in somatic mutations relevant to neoantigens

To demonstrate EGG's comprehensive neoantigen prediction capabilities, we analyzed the mutational landscape of 30 TCGA skin cutaneous melanoma (SKCM) samples [32]. As a descriptive view of the mutational landscape of the samples, we highlight the subset of somatic variants identified by Mutect2 that passed GATK's contamination and quality filters and were annotated as protein-coding, cancer-specific alterations (Figure 2).

Specifically, we identified three classes of mutations potentially giving rise to cancer neoantigens, including SNVs (including splicing site modifiers), indels, and gene fusions. Our analysis revealed substantial inter-sample variability in mutational burden across all these three genomic alteration categories. As widely reported, including TCGA SKCM and subsequent WGS studies, SNVs are the predominant mutation class in melanoma reflecting UV-driven mutagenesis [73,74]. This is consistent with our results where we found 1,000-5,000 somatic SNVs, though one sample (TCGA-BF-AAOX) exhibited the highest burden at ~5,300 variants (Figure 2A). While indel frequencies were generally lower, ranging from <15 to ~100 events per sample, with TCGA-EB-A3XF displaying the highest burden at 155 indels (Figure 2B). Gene fusion events, detected through STAR-Fusion analysis of tumor RNA-seq data, showed the lowest absolute frequencies with 2-24 fusion events identified per sample (Figure 2C). These results confirmed that while SNVs dominate the mutational landscape (around 90% of all variants), also indels (around 5-10%) and gene fusions (usually less than 2-3% per sample) contribute meaningfully to the total neoantigen repertoire (Figure 2D). EGG integrates several tools to carry out a comprehensive mutational profiling, allowing the users to process multiple classes of genomic alterations relevant to downstream neoantigen discovery[LM3.1].The tools embedded in the EGG workflow are reported in Additional File 1 Supplementary Material.

Figure 2. Mutational landscape analysis across TCGA samples processed by the EGG pipeline. (A) Single nucleotide variant (SNV) counts per sample, showing variable mutational burden with the sample TCGA-BF-AAOX being highly mutated (~5,300 SNVs). (B) Indel distribution, with most samples displaying fewer than 100 events and the sample TCGA-EB-A3XF showing the highest 155 indels. (C) Gene fusion events, range between 2-24 per sample. (D) Proportion of SNVs, Indels and Fusion events per sample displayed as boxplots.

Neoantigen burden distribution across mutation types

Neoantigen discovery modules of EGG, implemented via the pVACtools [22] suite, identified candidate epitopes derived from somatic variants (pVACseq), gene fusions (pVACfuse with STAR-Fusion input), and aberrant splicing events (pVACsplice with RegTools input) across the 30 samples selected from the TCGA melanoma cohort. Within the considered sample cohort, we identified up to 224 epitopes in a single sample across the three mutational classes (Figure 3A–3C). The binding affinity of epitopes to the MHC class I and class II complexes were computed. MHC class II slightly outnumbered class I predictions, consistently with the broader binding motif diversity of class II molecules. These results highlight the heterogeneity of neoantigen repertoires across patients and mutation types. To determine whether this heterogeneity simply reflected differences in input event burden, we correlated candidate neoantigen counts with the corresponding numbers of somatic variants and fusion events across the 30 TCGA-SKCM samples (Additional file 3, Supplementary Figure S1A–C). For somatic variant-derived candidates, the total neoantigen count showed only a weak and non-significant positive correlation with SNV count (Spearman $\rho = 0.26$, p-value = 0.159) and with combined SNV plus indel count ($\rho = 0.29$, p-value = 0.124). Similar weak, non-significant associations were observed when MHC classes were analyzed separately, with SNV plus indel burden correlating weakly with both MHC class I candidates ($\rho = 0.27$, p-value = 0.143) and MHC class II candidates ($\rho = 0.31$, p-value = 0.100). Thus, the variability in mutation-derived neoantigen yield was not explained by SNV burden alone. By contrast, fusion-derived neoantigen counts were strongly associated with the number of detected fusion events. Total fusion-derived candidate counts correlated positively with fusion event burden ($\rho = 0.72$, p-value = 7.29×10^{-6}), and this relationship was observed for both MHC class I ($\rho = 0.65$, p-value = 1.05×10^{-4}) and MHC class II candidates ($\rho = 0.73$, p-value = 5.37×10^{-6}). Together, these analyses show that EGG's neoantigen output depends on both the number and type of underlying genomic alterations, but is not a simple linear consequence of SNV burden. Candidate peptide yield is also influenced by alteration consequence, altered protein sequence length and context, patient-specific HLA genotype and MHC class.

Since EGG jointly considers peptides predicted to bind HLA class I and HLA class II alleles, it captures candidate neoantigens that could contribute to cytotoxic $CD8^+$ T-cell responses (class I) as well as helper $CD4^+$ T-cell responses (class II), two complementary arms of anti-tumor immunity thought to be important for durable responses. Moreover, by adding sample-specific co-expression network analysis as a second-tier prioritization, EGG reduces large candidate pools into a smaller set of biologically relevant and therapeutically promising targets, bridging the gap between computational prediction and the practical need to select a limited number of epitopes for inclusion in a vaccine formulation.

Figure 3. Predicted neoantigen landscape across TCGA melanoma samples processed by EGG. (A) Candidate neoantigens derived from somatic variants using pVACseq, stratified by MHC class (I and II). Samples yielded up to 224 epitopes, with somatic mutations contributing the largest overall pool. (B) Candidate neoantigens derived from gene fusions using pVACfuse with STAR-Fusion input, showing smaller but variable contributions across samples. (C) Candidate neoantigens derived from aberrant splicing events.

Integrating gene essentiality signals into epitope candidate ranking

Despite current methods are capable of identifying a remarkable amount of cancer neoantigen arising from genomic events, such as somatic mutations, fusions, and alternative splicing, translating this abundance into a clinically actionable candidates remains challenging. Therapeutic cancer vaccines typically deliver only a limited epitope set per patient, most peptide or mRNA platforms administer usually between 10 to 20 epitope sequences, whereas recent DNA plasmid vaccines have encoded larger payloads (up to 40 epitopes) in combination with PD-1 blockade. These design choices reflect manufacturing constraints, immunogenicity considerations, and trial feasibility [75–77]. Although sequencing-based pipelines can yield dozens to hundreds of candidate epitopes per tumour after standard HLA complex binding filters, current vaccine platforms can only accommodate a small subset of these in a single formulation. As a result, personalized vaccine design is not only an epitope level selection problem but also a gene level one: when only 10 to 40 epitopes can be used, choosing which genes they represent becomes necessary [2,80]. Prioritising epitopes from genes that are embedded in central, functionally indispensable positions within tumour networks offers a principled way to bias these limited slots toward targets that may be harder for the tumour to downregulate or eliminate under immune pressure [4,81,82].

In this study, we hypothesise that an additional layer of biological contextualisation is required to rationally select effective epitopes that are, in turn, clinically relevant for the specific patient. To address this, EGG integrates a network based filtering strategy that captures patient specific gene expression patterns and exploits several sources of prior knowledge to refine the search for cancer epitopes. Specifically, EGG builds co-expression networks that are representative of the patient-specific gene expression combinations. Such a network is modelled and refined by using experimentally validated protein-protein interactions and membrane protein annotations in order to leverage the biological relevance of the co-expression relationships. We call this integrated network, Patient Specific Functional Interaction Network (pFIN). pFINs serve as a context-aware framework for refining neoantigen candidates toward those most likely to exert meaningful therapeutic impact

(Figure 4). pFINs isn’t simply a gene co-expression network filtered by PPI we also enhanced it with the most confident connection related to membrane localized proteins. This membrane focused expansion is motivated by recent evidence that neoantigens derived from membrane localized proteins are enriched among immunogenic peptides and correlate with improved response to immune checkpoint blockade [14,15]. To clarify the scale of the pruned patient-specific functional interaction networks used for prioritisation, we also quantified the number of retained genes, edges, largest connected component size, and isolated genes across all 30 melanoma samples; these network-size summaries are provided in Additional file3, Supplementary Table S2.

Figure 4. Network-based integration framework for epitope prioritization. RNA-seq data from patient specific cancer biopsies were processed within the EGG pipeline to generate raw gene expression counts. Pearson correlation across the normalized matrix was used to construct an aggregate gene co-expression network, from which patient-specific networks were derived. Each patient-specific co-expression network was subsequently filtered and refined for biological relevance. Networks were first constrained to interactions present in an extended protein-protein interaction (PPI) reference (HumanNet-XN). Edges were then filtered to retain the strongest connections per node. To enhance immunological relevance, edges involving membrane associated genes (annotated by Gene Ontology terms) were added. The final output consisted of weighted, patient-specific functional interaction networks enriched for biologically supported and immunologically prioritized interactions. The steps illustrated in panels A and B are implemented by the sample_networks_generation; further usage details are provided are available as Additional file 1 (Supplementary Material).

EGG exploits the interactional properties of the pFINs predicting the functional relevance of each epitope’s gene of origin using topological metrics computed on the sample specific network degree, strength, betweenness centrality, and measures of network perturbation such as Weighted Connectivity Impact (WCI) and Largest Component Impact (LCI). In this manner it captures local connectivity, control of information flow, redundancy and resilience. These gene level features are integrated with orthogonal evidence: CRISPR dependency scores from DepMap [24] as an experimental proxy for essentiality, and epitope level sequence metrics from pVACtools (e.g., predicted HLA class I/II binding), which assess the intrinsic immunogenic potential of the peptide independently of its gene of origin. Since these inputs derive from different experiments and sources, they are, per se, non-integrable. EGG converts each data source to ranks and aggregates them via a Borda procedure to produce a consensus ranked epitope list per sample (Figure 5). To aid in the biological interpretation, prioritized genes are annotated with Gene Ontology terms, MSigDB Hallmarks of Cancer processes [26], and IntOGen driver labels [27]. We incorporated these network topological features including degree, strength, betweenness centrality, and perturbation-based scores such as LCI since it has been shown that the placement of a gene within interaction networks

is informative about its essentiality. The removal of highly connected and central proteins disproportionately fragments protein-protein interaction networks (“centrality-lethality” rule) [83–85]. Percolation style measures that quantify the impact of node removal on the size and weight of the largest connected component, similar in spirit to our LCI, further highlight nodes whose loss compromises network integrity and the function of core pathways [12]. By prioritizing epitopes derived from genes with high LCI and high centrality, EGG explicitly favors targets whose deletion would incur a substantial systems-level fitness cost, and are therefore less likely to be suppressed through immune editing. This design directly addresses a limitation of current neoantigen vaccines: tumors frequently evade immune pressure by losing, downregulating, or epigenetically silencing immunogenic but dispensable neoantigens. Longitudinal studies in lung cancer under immune checkpoint blockade have documented selective loss of neoantigens and reshaping of the neoantigen landscape during therapy [16]. More broadly, neoantigen depletion, via copy-number loss or transcriptional repression is considered a major immune escape route that could reduce the efficacy of personalized vaccines [16]. Recent work further argues that vaccines targeting passenger neoepitopes will drive immune editing of those mutations, whereas antigens derived from oncogenic drivers or other functionally indispensable genes are more durable because their loss carries a higher fitness penalty [86].

In addition, Al Bakir et al. showed that even a clonal EGFR driver neoantigen can be lost under combined TKI and neoantigen vaccine pressure, illustrating that oncogenic drivers themselves may be affected by immune escape [11]. This indicates that simply labelling genes as drivers or non-drivers is insufficient and supports incorporating different measures of functional indispensability to select the pool of candidate cancer vaccine epitopes. To complement topology-derived measures of indispensability with experimental evidence, EGG includes pan-cancer CRISPR dependency data from DepMap. Integrative analyses have shown that, given that network based features and genome scale CRISPR knockout screens capture non identical sets of essential genes, their combination can widen the search space for neoantigen-deriving genes [72,83,87]. In this framework, the experimental dependency layer provides a broad cancer-cell fitness prior, while patient specificity is retained through the pFIN-derived topology computed from each sample. This multi-criteria scheme allows a rational prioritisation of epitopes from genes that are both central in patient-specific pFINs and strongly essential across cancer cell lines, thereby increasing the chance that selected vaccine targets will remain expressed as the tumor evolves.

We further assessed the robustness and contribution of the pFIN network layer through several sensitivity analyses. Specifically, we evaluated pFIN stability using leave-one-background-sample-out jackknife analyses, top-edge consistency testing, and two GDC TCGA-SKCM benchmarks using larger n = 103 and n = 469 background cohorts. We also performed a membrane-edge expansion ablation,

which showed that the membrane-focused augmentation used in this case study had a modest overall effect: it promoted selected membrane-associated candidates but did not globally dominate the final prioritisation ranks. Users who wish to apply stronger membrane-focused prioritisation can increase the membrane-edge expansion parameter in their own analyses. These analyses are provided in Additional file 3, Supplementary Tables S3–S7.

Our strategy explicitly mitigates immune escape via deletion or silencing of dispensable neoantigen source genes. However, this focus also defines its main limitation: EGG does not model escape mechanisms that operate through impaired antigen presentation, nor does it capture several immune-side determinants of neoantigen immunogenicity beyond peptide-HLA binding, such as antigen processing efficiency, T-cell receptor repertoire constraints, and immunodominance. EGG should therefore be interpreted as a prioritization framework centered on the tumor-side evolutionary durability of neoantigen source genes, rather than as a complete predictor of anti-tumor immune recognition. Consistent with this limitation, genetic and epigenetic alterations in HLA class I/II, β2-microglobulin, antigen processing machinery, and IFNγ signaling are frequent in clinical tumors and represent dominant routes of immune evasion under T cell pressure [88–90]. In such settings, neoantigen source gene essentiality becomes largely irrelevant, as tumor cells can evade recognition despite maintaining expression of the targeted gene.

Figure 5. Epitope prioritisation scheme. Patient Specific Functional Interaction Networks were used as the basis for epitope prioritization. Topological network metrics obtained from degree, edges strength, betweenness (including LCI, WCI) were computed to establish a measure of gene essentiality specific to individual patients. These network-derived features were combined with external biological evidence, including CRISPR-based gene essentiality scores (DepMap), epitope specific sequence metrics such as predicted HLA binding affinity. The integrated information was aggregated into a ranked epitope list, ensuring robust consensus prioritization. To provide additional biological context, candidate genes were annotated with cancer hallmarks, Gene Ontology terms, and IntOGen driver gene information. The resulting framework enables informed selection of epitopes by linking immunogenic potential to system-level relevance and cancer biology. The steps illustrated in this figure are implemented by the epitopes_prioritisation module with details of usage are provided in Additional file 1 (Supplementary Material).

Determinants of epitope prioritization

By applying our multi-step prioritisation approach described above across our TCGA melanoma samples, we identified correlation patterns among features utilised in the epitope prioritisation. Such pairwise relationships are summarised in Figure 6A. As expected, network centrality-based metrics are strongly intercorrelated and align closely with the Borda score, indicating that candidates from highly

connected or influential genes tend to occupy top positions in the rank. DepMap survivability and predicted binding affinity showed a mild negative correlation with the Borda scores (negative values imply greater essentiality), and only limited correlation with network centralities. While DepMap highlights experimentally observed dependencies and provides biologically informed metrics, network centrality measures capture data-driven patterns of connectivity based on the unique molecular landscape of each patient. Exploiting both levels of information yields a more robust prioritization compared to single layer prioritisation methods. We hypothesize that the prioritization of epitopes according to essentiality and network centrality will constrain immune escape, as the elimination of such genes is expected to impair tumor fitness. EGG's ranking aligns most strongly with network centrality measures, while also retaining contributions from sequence-dependent features like HLA binding affinity and gene essentiality from CRISPR knockout studies. HLA expression was not included as a ranking criterion in the analysis presented here, explaining its limited correlation with the Borda score. This reflects EGG's emphasis on complementing sequence-based epitope prioritisation with gene-level functional information, including database-derived essentiality and patient-specific network topology. Nevertheless, HLA expression is retained in the final output tables and can be considered during downstream interpretation or included by the user as an additional ranking criterion in the borda score, together with other optional pVACtools algorithm outputs.We also evaluated the contribution of membrane-associated genes to the final prioritized epitope lists, given that membrane-associated edge expansion was included as an optional network refinement step. Across the 30 patients, membrane-associated genes accounted for a median of 22.3% of all candidate epitopes. Among top-ranked candidates, the median number of membrane-associated epitopes was 2 in the top 10, 4 in the top 20, and 10 in the top 50. Thus, membrane-derived epitopes were recovered among prioritized candidates, but they did not dominate the top-ranked sets. This is consistent with the intentionally light membrane-edge expansion used in the default analysis; stronger prioritization effects would be expected only if users deliberately increase the membrane-edge expansion parameter.

Driver-associated epitopes showed a modest but significant overrepresentation among the top prioritized candidates compared with the remaining candidate pool. As shown in Figure 6B, driver-derived epitopes accounted for 34/296 top-10 candidates across samples (11.5%), compared with 133/1992 non-top-10 candidates (6.7%; one-sided Fisher's exact test, odds ratio = 1.81, p-value = 0.0034). These driver-derived candidates included loss-of-function, activating, and mixed activating/loss-of-function driver annotations. Although most top-ranked epitopes were still derived from non-driver genes, the relative overrepresentation of driver-derived candidates is consistent with the inclusion of functional and network-context features in the prioritization framework. One possible

explanation is that some cancer driver genes occupy more central or functionally important positions in the network, although this interpretation remains exploratory.To further evaluate potential determinants of prioritisation, we tested whether peptide length or tumor DNA variant allele frequency (VAF) influenced final epitope rank. Neither feature showed a meaningful association with prioritisation outcome, supporting that EGG ranking was not primarily driven by peptide-length bias or VAF differences in this dataset (Additional file 3, Supplementary Figures 2 and 3).

In order to ascertain whether overrepresented epitopes across the population exist, we then assessed the occurrence of epitope candidates across patients. We observed that exact peptides are unique to individual patients (figure 6D), while the genes that give rise to epitopes show a small recurrence across cases (figure 6C). Altogether, these descriptive analyses illustrate the intended behavior of EGG in this case study: the pipeline defines the candidate space onto epitopes anchored in tumor-relevant biology, enriches for driver-gene targets, and produces shortlists that remain largely patient-specific at the peptide level while still revealing a small cohort of recurrent genes and epitope sequences that could inform shared targets or follow-up functional studies. Building on this, our data pinpoints a small set of recurrent targets. Among fusion derived events, KANSL1 is the most frequently shared gene (12 samples; figure 6C), and DIYKQIRANKVSVWRQ the most recurrent exact epitope (5 patients; figure 6D). These KANSL1-associated events were primarily linked to the KANSL1–ARL17/KANSARL fusion locus, which has been described previously in cancer transcriptomic studies [91]. A comparable pattern emerges for genomic mutations: exact mutation-derived peptides remain patient-specific, but the gene NRAS shows the highest recurrence (7 samples; figure 6C), with the epitope LDILDTAGREEYSAMRDQYMRTGEGFLCV shared across 3 patients (figure 6D). Since NRAS is a known recurrent melanoma driver, we compared this observation with the reported TCGA-SKCM NRAS mutation frequency of 28%. The observed frequency of NRAS-derived epitope candidates in the selected cohort was not higher than expected relative to this background rate (one-sided binomial test, p-value = 0.776), indicating that NRAS recurrence is consistent with the known melanoma mutational landscape rather than evidence of unexpected overrepresentation [74]. In both fusion and genomic contexts, the presence of recurrent epitopes highlights the potential for “off-the-shelf” vaccination strategies, an approach already explored for shared/public neoantigens such as KRAS hotspots and frameshift peptides in microsatellite instability (MSI) tumors. Early trials of shared neoantigen vaccines (including KRAS focused mRNA platforms and multi epitope regimens) demonstrate feasibility and immunogenicity, underscoring the translational value of the recurrent sequences identified by epitope generation pipelines [92].

Fundamentally, the main outputs of EGG, are interactive prioritization tables for each sample that integrate epitope and gene level features into a unified view. These tables report the HLA allele

predicted to present each epitope together with corresponding transcript abundance, peptide sequence, mutation source, and class I/II designation. For each candidate, network-derived features (degree, strength, betweenness, LCI, WCI) and external evidence (DepMap survivability, IntOGen driver status, cancer hallmark annotation, GO terms) are displayed alongside the consensus Borda score and rank. This design provides investigators with a transparent overview of how multiple layers of information contribute to prioritization, linking peptide level immunogenicity with the biological relevance of the source gene. Tables for each patient that include prioritised epitopes and their topological and biological features obtained in our analysis can be found in Additional file 2 (Supplementary Material). To provide orthogonal bioinformatic support for the prioritisation strategy, we compared the final EGG Borda ranking with an IC50 MHC binding affinity only ranking using external experimentally supported epitope evidence from the Immune Epitope Database (IEDB) [93], the Cancer Epitope Database and Analysis Resource (CEDAR) [94], and TSNAdb v2.0 [95]. Because exact full-length peptide matches were rare, we used a relaxed min-8 containment match to identify candidates containing experimentally supported presented or immunogenic peptide cores; this analysis showed that Borda ranking improved recovery of externally supported candidates relative to IC50-only ranking at the most selective top-ranked cutoffs (Additional file 3, Supplementary Figure S6-S8 and Supplementary Tables S8–S9). Although EGG is designed to prioritise epitopes derived from genes with greater functional and network relevance, the proposed evolutionary durability of such targets remains a hypothesis in the present study. The external epitope-database analysis supports the potential presentation and immunogenicity of selected candidates, but does not directly test whether their source genes are less likely to be lost or silenced under immune pressure.

Figure 6. Exploratory analysis of epitope features. (A) Spearman correlation between features used in the Borda ranking procedure, highlighting expected dependencies among network-based measures and binding affinity scores. (B) Fraction of epitopes derived from driver versus non-driver genes within the top-10 prioritized epitopes per sample, showing contributions from both loss-of-function (inactivating alterations disabling tumor suppressors) and activating (gain-of-function mutations activating oncogenes) drivers. (C) Distribution of genes shared between samples, showing that most candidate genes are unique to individual samples. A value of 1 indicates that the gene was observed in one sample only. (D) Distribution of epitope sequences shared between samples, with the majority being patient specific. A value of 1 indicates that the epitope sequence was observed in one sample only. Most genes and epitope sequences were patient-specific.

Comparison of currently available neoantigen prediction pipelines

EGG adds a co-expression-network based prioritization step that ranks neoantigens by the centrality and indispensability of their source genes, with the goal of reducing immune evasion risk. The pipeline is implemented in Snakemake and automates resource retrieval and environment setup for local

execution. Compared with prominent publicly available pipelines surveyed in Table 1, EGG's network-aware prioritization constitutes a distinctive feature while keeping installation and execution simple. To contextualize EGG within the current landscape, we note the following pipelines: pVACtools stands out as a modular ecosystem (pVACseq/bind/fuse/splice) with extensive algorithm support and integrated visualization (pVACview) and vaccine design utilities (pVACvector); pVACtools also includes comprehensive workflows, such as immuno.wdl on the Terra platform, to automate end-to-end analyses, including upstream preprocessing. TSNAD v2.0 is an end-to-end pipeline for tumor-normal WES/WGS (with optional RNA-seq) that incorporates expression and gene-fusion filters and uses the deep-learning predictor DeepHLApan to score both HLA binding and immunogenicity [96,97]. ScanNeo2 provides an end-to-end workflow from raw DNA/RNA data and uniquely broadens variant coverage to include exitron splicing and gene fusions alongside SNVs/indels, enabling wider candidate recovery [9]. INTEGRATE-neo is purpose-built for fusion-derived neoantigens, offering an automated pipeline from fusion calling through HLA binding specifically for gene-fusion events [5]. nextNEOpi [98]is a Nextflow [99] based, fully automated pipeline that starts from raw WES/WGS in combination with RNA-seq and additionally quantifies patient and neoepitope level features linked to immunogenicity and response to checkpoint blockade. NeoHunter emphasizes flexible input handling and systematically detects and prioritizes neoantigens not only from SNVs/indels but also from gene fusions and aberrant splicing within a single framework [10]. NeoDisc is an end-to-end clinical proteogenomic pipeline that integrates immunopeptidomics with genomics and transcriptomics to prioritize both canonical and noncanonical tumor specific antigens [100]. SNAF focuses on splicing-derived antigens, predicting MHC-bound T-cell epitopes and altered-surface B-cell targets and using machine-learning prioritization to enrich for immunogenic events [8]. Seq2Neo provides a one-stop workflow from raw data and incorporates a convolutional neural network immunogenicity model to rank candidates derived from SNVs, indels, and gene fusions [101]. TransHLA is a recent deep-learning model for HLA-presented epitope detection rather than a full sequencing-to-neoantigen workflow; it uses Transformer and Residue CNN architectures with ESM2-derived sequence and structure embeddings to predict HLA class I and class II epitopes, and notably performs peptide-level screening without requiring HLA alleles as input [102].

Crucially, the prioritization module of EGG is flexible and fully customizable by the user. Optional features can be enabled or disabled and thresholds adjusted, for example using different algorithm for peptide MHC binding, immunogenicity scores, gene essentiality, and multiple network-based centrality metrics. The relative weights of these features used during prioritization are also modifiable by the users, allowing researchers to emphasize specific criteria for a customizable ranking process.

Table 1. Feature matrix for neoantigen prediction pipelines and epitope-prediction tools. This table compares the Epitope Generation Gateway (EGG) with selected public neoantigen prediction workflows and standalone epitope-prediction tools. For each tool, we summarize supported somatic alteration classes, input requirements, MHC class coverage, HLA typing or HLA input requirements, immunogenicity or presentation prediction, and prioritization features. Because some entries, such as TransHLA, are prediction models rather than full end-to-end sequencing pipelines, not all workflow-level features are applicable. Checkmarks indicate that the feature is included or supported by the tool or tool suite; dashes indicate that the feature is not part of the standard reported functionality or is not applicable. EGG is distinguished by combining neoantigen discovery with patient-specific source-gene co-expression context, PPI-informed network metrics, DepMap-based gene essentiality, and configurable weighted consensus ranking.

Conclusion

Epitope Generation Gateway (EGG) can bridge the gap between abundant raw neoantigen predictions and the practical need for biologically meaningful ordered results. By embedding candidate epitopes within patient-specific functional interaction networks and incorporating orthogonal evidence of essentiality and driver status, EGG prioritizes epitopes more likely to be therapeutically relevant by prioritising more essential genes less likely to be subject to immune evasion while also offering the user with alternative prioritisation methods and important gene related biological information. In the case study presented in this work, we highlight that the majority of epitopes remain unique between patients at the sequence level, whereas gene level recurrence is more common. These results show that EGG can serve as a flexible framework for integrating diverse data sources, balancing immunological plausibility with tumor systems biology, and informing the rational design of personalized cancer vaccines.

Abbreviations

EGG: Epitope Generation Gateway, GO: Gene Ontology, HLA: Human Leukocyte Antigen, LCC: Largest Connected Component, LCI: Largest Component Impact, MHC: Major Histocompatibility Complex, MSI: Microsatellite Instability, pFIN: Patient Specific Functional Interaction Network, PPI: Protein Protein Interaction, SKCM: Skin Cutaneous Melanoma, SNV: Single Nucleotide Variation, TCGA: The Cancer Genome Atlas, VAF: Variant Allele Frequency, vst: Variance Stabilizing Transformation, WCI: Weighted Connectivity Impact.

Data availability

Raw data: Matched tumor RNA-seq, tumor DNA (WES), and normal DNA samples were retrieved from The Cancer Genome Atlas (TCGA) for 30 patients diagnosed with cutaneous melanoma (TCGA-SKCM study code) [32]. The 30 corresponding UUIDs are listed in Additional file 2.

Output data: Output data is provided in Additional file 2.

Supplementary Methods: Pipeline Implementation is described in Additional file 1.

Supplementary Analyses: Supplementary analyses are described in Additional file 3.

Availability of source code and requirements

Project name: epitope_generation_gateway

Project home page: https://github.com/fhaive/epitope_generation_gateway

License: GPL-3.0 license

Operating system(s): Linux / Unix-like systems

Programming language: Snakemake, Python, R, Bash/shell, and YAML

Other requirements: Conda or Mamba, Snakemake ≥6.0, Docker, and standard command-line bioinformatics dependencies installed through the workflow-managed Conda environments and Docker containers. Recommended computational resources depend on dataset size, sequencing depth, mutation burden, and the number of samples. For the 30-sample TCGA melanoma benchmark, the most memory-intensive EGG-specific step was membrane-edge enrichment, requiring approximately 75 GB peak RAM per sample, while LIONESS reconstruction required approximately 36 GB peak RAM for the 30-sample cohort.

RRID: SCR_028792

bio.tools: epitope_generation_gateway

WorkflowHub: https://workflowhub.eu/workflows/2165?version=2

Competing interests

The authors declare that they have no competing interests that could have influenced the work reported in this paper.

Authors contributions

LM: Data Analysis, Investigation, Methodology, Software, Visualisation, Conceptualisation, Writing – original draft, Writing – review and editing, MF: Software, Methodology, Investigation, JC: Methodology, Writing – review and editing, FD: Methodology, Writing – review and editing, ED: Software, Writing – review and editing, MN: Writing – review and editing, AS: Methodology, Investigation, Writing – review and editing, SY: Conceptualisation, Funding Acquisition, Writing – review and editing, VC: Conceptualisation, Funding Acquisition, Writing – review and editing, AF: Conceptualisation, Investigation, Methodology, Supervision, Writing – original draft, Writing – review and editing, DG: Conceptualisation, Funding Acquisition, Investigation, Methodology, Supervision, Writing – review and editing

Additional files

Additional file 1 (.docx): Supplementary Methods / Supplementary Protocol.

Description: Step-by-step instructions for execution of individual pipeline modules.

Additional file 2 (.xlsx): Supplementary Tables.

Description: Patient-level tables of prioritised epitopes and associated topological and biological features.

Additional file 3 (.docx): Supplementary Analyses:

Description**:** Additional sensitivity, robustness, benchmarking, and validation analyses supporting the prioritisation framework, including network stability, ranking-weight sensitivity, membrane-expansion ablation, peptide-length and VAF assessments, external evidence matching, and genomic-event burden correlations.

Acknowledgement

During manuscript preparation, we used AI assisted tools to improve clarity and readability of text and as a coding aid. AI-assisted suggestions were reviewed and edited by the authors, who take full responsibility for the final content.

Funding

This work was supported by the Jane and Aatos Erkko Foundation.

References

1. Sahin U, Derhovanessian E, Miller M, Kloke B-P, Simon P, Löwer M, et al.. Personalized RNA mutanome vaccines mobilize poly-specific therapeutic immunity against cancer. *Nature*. 2017; doi: 10.1038/nature23003.

2. Ott PA, Hu Z, Keskin DB, Shukla SA, Sun J, Bozym DJ, et al.. An immunogenic personal neoantigen vaccine for patients with melanoma. *Nature*. 2017; doi: 10.1038/nature22991.

3. Li X, You J, Hong L, Liu W, Guo P, Hao X. Neoantigen cancer vaccines: a new star on the horizon. *Cancer Biol Med*. 2023; doi: 10.20892/j.issn.2095-3941.2023.0395.

4. Bang H, Park JS, Kim JY, Sung C, An J, Cho D, et al.. Gene essentiality for tumour growth influences neoantigen-directed immunoediting. *Clinical & Translational Med*. 2022; doi: 10.1002/ctm2.714.

5. Zhang J, Mardis ER, Maher CA. INTEGRATE-neo: a pipeline for personalized gene fusion neoantigen discovery. Hancock J, editor. *Bioinformatics*. 2017; doi: 10.1093/bioinformatics/btw674.

6. Nielsen M, Lundegaard C, Blicher T, Lamberth K, Harndahl M, Justesen S, et al.. NetMHCpan, a Method for Quantitative Predictions of Peptide Binding to Any HLA-A and -B Locus Protein of Known Sequence. Kallas E, editor. *PLoS ONE*. 2007; doi: 10.1371/journal.pone.0000796.

7. Zhou Z, Lyu X, Wu J, Yang X, Wu S, Zhou J, et al.. TSNAD: an integrated software for cancer somatic mutation and tumour-specific neoantigen detection. *R Soc Open Sci*. 2017; doi: 10.1098/rsos.170050.

8. Li G, Mahajan S, Ma S, Jeffery ED, Zhang X, Bhattacharjee A, et al.. Splicing neoantigen discovery with SNAF reveals shared targets for cancer immunotherapy. *Sci Transl Med*. 2024; doi: 10.1126/scitranslmed.ade2886.

9. Schäfer RA, Guo Q, Yang R. ScanNeo2: a comprehensive workflow for neoantigen detection and immunogenicity prediction from diverse genomic and transcriptomic alterations. Kendziorski C, editor. *Bioinformatics*. 2023; doi: 10.1093/bioinformatics/btad659.

10. Ma T, Zhao Z, Li H, Wei L, Zhang X. NeoHunter: Flexible software for systematically detecting neoantigens from sequencing data. *Quant Biol*. 2024; doi: 10.1002/qub2.28.

11. Al Bakir M, Reading JL, Gamble S, Rosenthal R, Uddin I, Rowan A, et al.. Clonal driver neoantigen loss under EGFR TKI and immune selection pressures. *Nature*. 2025; doi: 10.1038/s41586-025-08586-y.

12. Li X, Li W, Zeng M, Zheng R, Li M. Network-based methods for predicting essential genes or proteins: a survey. *Briefings in Bioinformatics*. 2020; doi: 10.1093/bib/bbz017.

13. Liu Y, Zhu F, Li X, Guan X, Hou Y, Feng Y, et al.. SpatialSNV: A novel method for identifying and analyzing spatially resolved SNVs in tumor microenvironments. *GigaScience*. 2025; doi: 10.1093/gigascience/giaf065.

14. Goldberger Z, Hauert S, Chang K, Kurtanich T, Alpar AT, Repond G, et al.. Membrane-localized neoantigens predict the efficacy of cancer immunotherapy. *Cell Reports Medicine*. 2023; doi: 10.1016/j.xcrm.2023.101145.

15. Castro A, Kaabinejadian S, Yari H, Hildebrand W, Zanetti M, Carter H. Subcellular location of source proteins improves prediction of neoantigens for immunotherapy. *EMBO J*. 2022; doi: 10.15252/embj.2022111071.

16. Xie N, Shen G, Gao W, Huang Z, Huang C, Fu L. Neoantigens: promising targets for cancer therapy. *Sig Transduct Target Ther*. 2023; doi: 10.1038/s41392-022-01270-x.

17. Anagnostou V, Smith KN, Forde PM, Niknafs N, Bhattacharya R, White J, et al.. Evolution of Neoantigen Landscape during Immune Checkpoint Blockade in Non–Small Cell Lung Cancer. *Cancer Discovery*. 2017; doi: 10.1158/2159-8290.CD-16-0828.

18. Benjamin D, Sato T, Cibulskis K, Getz G, Stewart C, Lichtenstein L. Calling Somatic SNVs and Indels with Mutect2. bioRxiv. 2019. doi: 10.1101/861054

19. Haas BJ, Dobin A, Stransky N, Li B, Yang X, Tickle T, et al.. STAR-Fusion: Fast and Accurate Fusion Transcript Detection from RNA-Seq. bioRxiv. 2017. doi: 10.1101/120295

20. Orenbuch R, Filip I, Comito D, Shaman J, Pe’er I, Rabadan R. arcasHLA: high-resolution HLA typing from RNAseq. Ponty Y, editor. *Bioinformatics*. 2020; doi: 10.1093/bioinformatics/btz474.

21. Hoang M, Richters M, Kiwala S, Griffith O, Griffith M. 32. pVACsplice: A Computational tool for predicting and prioritizing alternative splicing neoantigens. *Cancer Genetics*. 2024; doi: 10.1016/j.cancergen.2024.08.034.

22. Hundal J, Kiwala S, McMichael J, Miller CA, Xia H, Wollam AT, et al.. pVACtools: A Computational Toolkit to Identify and Visualize Cancer Neoantigens. *Cancer Immunology Research*. 2020; doi: 10.1158/2326-6066.CIR-19-0401.

23. Hundal J, Carreno BM, Petti AA, Linette GP, Griffith OL, Mardis ER, et al.. pVAC-Seq: A genome-guided in silico approach to identifying tumor neoantigens. *Genome Med*. 2016; doi: 10.1186/s13073-016-0264-5.

24. DepMap, Broad. DepMap 24Q4 Public. Figshare+. 2024. doi:10.25452/figshare.plus.27993248.v1.

25. The Gene Ontology Consortium, Carbon S, Douglass E, Good BM, Unni DR, Harris NL, et al.. The Gene Ontology resource: enriching a GOld mine. *Nucleic Acids Research*. 2021; doi: 10.1093/nar/gkaa1113.

26. Liberzon A, Birger C, Thorvaldsdóttir H, Ghandi M, Mesirov JP, Tamayo P. The Molecular Signatures Database Hallmark Gene Set Collection. *Cell Systems*. 2015; doi: 10.1016/j.cels.2015.12.004.

27. Martínez-Jiménez F, Muiños F, Sentís I, Deu-Pons J, Reyes-Salazar I, Arnedo-Pac C, et al.. A compendium of mutational cancer driver genes. *Nat Rev Cancer*. 2020; doi: 10.1038/s41568-020-0290-x.

28. epitope_generation_gateway. https://github.com/fhaive/epitope_generation_gateway

29. epitope_generation_gateway. https://bio.tools/epitope_generation_gateway.

30. epitope_generation_gateway. https://scicrunch.org/resolver/SCR_028792

31. Mannino L. EGG: Epitope Generation Gateway A modular Snakemake pipeline for personalized neoantigen discovery and prioritization using patient-specific gene co-expression networks. WorkflowHub; doi:10.48546/WORKFLOWHUB.WORKFLOW.2165.2; https://workflowhub.eu/workflows/2165?version=2

32. The Cancer Genome Atlas Research Network, Weinstein JN, Collisson EA, Mills GB, Shaw KRM, Ozenberger BA, et al.. The Cancer Genome Atlas Pan-Cancer analysis project. *Nat Genet*. 2013; doi: 10.1038/ng.2764.

33. Zhang Z, Hernandez K, Savage J, Li S, Miller D, Agrawal S, et al.. Uniform genomic data analysis in the NCI Genomic Data Commons. *Nat Commun*. 2021; doi: 10.1038/s41467-021-21254-9.

34. Danecek P, Bonfield JK, Liddle J, Marshall J, Ohan V, Pollard MO, et al.. Twelve years of SAMtools and BCFtools. *GigaScience*. 2021; doi: 10.1093/gigascience/giab008.

35. Li H, Handsaker B, Wysoker A, Fennell T, Ruan J, Homer N, et al.. The Sequence Alignment/Map format and SAMtools. *Bioinformatics*. 2009; doi: 10.1093/bioinformatics/btp352.

36. : Babraham Bioinformatics - FastQC A Quality Control tool for High Throughput Sequence Data. https://www.bioinformatics.babraham.ac.uk/projects/fastqc/ Accessed 2025 Dec 17.

37. Chen S, Zhou Y, Chen Y, Gu J. fastp: an ultra-fast all-in-one FASTQ preprocessor. *Bioinformatics*. 2018; doi: 10.1093/bioinformatics/bty560.

38. Mudge JM, Carbonell-Sala S, Diekhans M, Martinez JG, Hunt T, Jungreis I, et al.. GENCODE 2025: reference gene annotation for human and mouse. *Nucleic Acids Research*. 2025; doi: 10.1093/nar/gkae1078.

39. Schneider VA, Graves-Lindsay T, Howe K, Bouk N, Chen H-C, Kitts PA, et al.. Evaluation of GRCh38 and de novo haploid genome assemblies demonstrates the enduring quality of the reference assembly. *Genome Res*. 2017; doi: 10.1101/gr.213611.116.

40. Ticke T, Bankapur A, Ganote C, Fulton B, Tirosh I, Chen J, et al.. Trinity CTAT: a community resource for *de novo* and reference-based RNA-Seq analysis. F1000Research. 2016;5:1844. doi:10.7490/f1000research.1112725.1.

41. Liao Y, Smyth GK, Shi W. The R package Rsubread is easier, faster, cheaper and better for alignment and quantification of RNA sequencing reads. *Nucleic Acids Research*. 2019; doi: 10.1093/nar/gkz114.

42. Liao Y, Smyth GK, Shi W. featureCounts: an efficient general purpose program for assigning sequence reads to genomic features. *Bioinformatics*. 2014; doi: 10.1093/bioinformatics/btt656.

43. Köster J, Rahmann S. Snakemake—a scalable bioinformatics workflow engine. *Bioinformatics*. 2012; doi: 10.1093/bioinformatics/bts480.

44. Murphy C, Elemento O. AGFusion: annotate and visualize gene fusions. bioRxiv. 2016. doi: 10.1101/080903

45. Cunningham F, Allen JE, Allen J, Alvarez-Jarreta J, Amode MR, Armean IM, et al.. Ensembl 2022. *Nucleic Acids Research*. 2022; doi: 10.1093/nar/gkab1049.

46. Mistry J, Chuguransky S, Williams L, Qureshi M, Salazar GA, Sonnhammer ELL, et al.. Pfam: The protein families database in 2021. *Nucleic Acids Research*. 2021; doi: 10.1093/nar/gkaa913.

47. Shao XM, Bhattacharya R, Huang J, Sivakumar IKA, Tokheim C, Zheng L, et al.. High-Throughput Prediction of MHC Class I and II Neoantigens with MHCnuggets. *Cancer Immunology Research*. 2020; doi: 10.1158/2326-6066.CIR-19-0464.

48. Kent WJ, Sugnet CW, Furey TS, Roskin KM, Pringle TH, Zahler AM, et al.. The Human Genome Browser at UCSC. *Genome Res*. 2002; doi: 10.1101/gr.229102.

49. McKenna A, Hanna M, Banks E, Sivachenko A, Cibulskis K, Kernytsky A, et al.. The Genome Analysis Toolkit: A MapReduce framework for analyzing next-generation DNA sequencing data. *Genome Res*. 2010; doi: 10.1101/gr.107524.110.

50. Van Der Auwera GA, Carneiro MO, Hartl C, Poplin R, Del Angel G, Levy-Moonshine A, et al.. From FastQ Data to High-Confidence Variant Calls: The Genome Analysis Toolkit Best Practices Pipeline. *CP in Bioinformatics*. 2013; doi: 10.1002/0471250953.bi1110s43.

51. Li H, Durbin R. Fast and accurate short read alignment with Burrows–Wheeler transform. *Bioinformatics*. 2009; doi: 10.1093/bioinformatics/btp324.

52. Sherry ST. dbSNP: the NCBI database of genetic variation. *Nucleic Acids Research*. 2001; doi: 10.1093/nar/29.1.308.

53. Karczewski KJ, Francioli LC, Tiao G, Cummings BB, Alföldi J, Wang Q, et al.. The mutational constraint spectrum quantified from variation in 141,456 humans. *Nature*. 2020; doi: 10.1038/s41586-020-2308-7.

54. . GATK Team. Resource bundle. https://gatk.broadinstitute.org/hc/en-us/articles/360035890811-Resource-bundle; Accessed 4 April 2025

55. Li H. Tabix: fast retrieval of sequence features from generic TAB-delimited files. *Bioinformatics*. 2011; doi: 10.1093/bioinformatics/btq671.

56. Li H. Aligning sequence reads, clone sequences and assembly contigs with BWA-MEM. arXiv. 2013. doi: 10.48550/arXiv.1303.3997

57. : Broad Institute. Picard Tools. https://broadinstitute.github.io/picard/ Accessed 2025 Dec 17.

58. McLaren W, Gil L, Hunt SE, Riat HS, Ritchie GRS, Thormann A, et al.. The Ensembl Variant Effect Predictor. *Genome Biol*. 2016; doi: 10.1186/s13059-016-0974-4.

59. Bray NL, Pimentel H, Melsted P, Pachter L. Near-optimal probabilistic RNA-seq quantification. *Nat Biotechnol*. 2016; doi: 10.1038/nbt.3519.

60. Guille A, Adélaïde J, Finetti P, Andre F, Birnbaum D, Mamessier E, et al.. A benchmarking study of individual somatic variant callers and voting-based ensembles for whole-exome sequencing. *Briefings in Bioinformatics*. 2024; doi: 10.1093/bib/bbae697.

61. Haas BJ, Dobin A, Li B, Stransky N, Pochet N, Regev A. Accuracy assessment of fusion transcript detection via read-mapping and de novo fusion transcript assembly-based methods. *Genome Biol*. 2019; doi: 10.1186/s13059-019-1842-9.

62. Cotto KC, Feng Y-Y, Ramu A, Richters M, Freshour SL, Skidmore ZL, et al.. Integrated analysis of genomic and transcriptomic data for the discovery of splice-associated variants in cancer. *Nat Commun*. 2023; doi: 10.1038/s41467-023-37266-6.

63. Saha E, Ben Guebila M, Fanfani V, Shutta KH, DeMeo DL, Quackenbush J, et al.. Aging-associated alterations in gene regulatory networks associate with risk, prognosis and response to therapy in lung adenocarcinoma. *NPJ Aging*. 2025; doi: 10.1038/s41514-025-00247-8.

64. Fanfani V, Shutta KH, Mandros P, Fischer J, Saha E, Micheletti S, et al.. Reproducible processing of TCGA regulatory networks. *GigaScience*. 2025; doi: 10.1093/gigascience/giaf126.

65. Love MI, Huber W, Anders S. Moderated estimation of fold change and dispersion for RNA-seq data with DESeq2. *Genome Biol*. 2014; doi: 10.1186/s13059-014-0550-8.

66. Kuijjer ML, Hsieh P-H, Quackenbush J, Glass K. lionessR: single sample network inference in R. *BMC Cancer*. 2019; doi: 10.1186/s12885-019-6235-7.

67. Hwang S, Kim CY, Yang S, Kim E, Hart T, Marcotte EM, et al.. HumanNet v2: human gene networks for disease research. *Nucleic Acids Research*. 2019; doi: 10.1093/nar/gky1126.

68. Carlson M. org.Hs.eg.db. Bioconductor. 2017. doi: 10.18129/B9.bioc.org.Hs.eg.db.

69. Conin B, Billault-Chaumartin I, El Sayyed H, Quenech’Du N, Cockram C, Koszul R, et al.. Extended sister-chromosome catenation leads to massive reorganization of the *E. coli* genome. *Nucleic Acids Research*. 2022; doi: 10.1093/nar/gkac105.

70. Durinck S, Spellman PT, Birney E, Huber W. Mapping identifiers for the integration of genomic datasets with the R/Bioconductor package biomaRt. *Nat Protoc*. 2009; doi: 10.1038/nprot.2009.97.

71. Csárdi G, Nepusz T, Müller K, Horvát S, Traag V, Zanini F, et al.(2025, October 29). igraph for R: R interface of the igraph library for graph theory and network analysis. Zenodo (Version v2.2.1). doi: 10.5281/ZENODO.7682609.

72. Dempster JM, Boyle I, Vazquez F, Root DE, Boehm JS, Hahn WC, et al.. Chronos: a cell population dynamics model of CRISPR experiments that improves inference of gene fitness effects. *Genome Biol*. 2021; doi: 10.1186/s13059-021-02540-7.

73. Hayward NK, Wilmott JS, Waddell N, Johansson PA, Field MA, Nones K, et al.. Whole-genome landscapes of major melanoma subtypes. *Nature*. 2017; doi: 10.1038/nature22071.

74. Akbani R, Akdemir KC, Aksoy BA, Albert M, Ally A, Amin SB, et al.. Genomic Classification of Cutaneous Melanoma. *Cell*. 2015; doi: 10.1016/j.cell.2015.05.044.

75. Yarchoan M, Gane EJ, Marron TU, Perales-Linares R, Yan J, Cooch N, et al.. Personalized neoantigen vaccine and pembrolizumab in advanced hepatocellular carcinoma: a phase 1/2 trial. *Nat Med*. 2024; doi: 10.1038/s41591-024-02894-y.

76. Biswas N, Chakrabarti S, Padul V, Jones LD, Ashili S. Designing neoantigen cancer vaccines, trials, and outcomes. *Front Immunol*. 2023; doi: 10.3389/fimmu.2023.1105420.

77. Wu D-W, Jia S-P, Xing S-J, Ma H, Wang X, Tang Q-Y, et al.. Personalized neoantigen cancer vaccines: current progression, challenges and a bright future. *Clin Exp Med*. 2024; doi: 10.1007/s10238-024-01436-7.

78. Cai Y, Li D, Lv D, Yu J, Ma Y, Jiang T, et al.. MHC-I-presented non-canonical antigens expand the cancer immunotherapy targets in acute myeloid leukemia. *Sci Data*. 2024; doi: 10.1038/s41597-024-03660-y.

79. Ye L, Creaney J, Redwood A, Robinson B. The Current Lung Cancer Neoantigen Landscape and Implications for Therapy. *Journal of Thoracic Oncology*. 2021; doi: 10.1016/j.jtho.2021.01.1624.

80. Li J, Xiao Z, Wang D, Jia L, Nie S, Zeng X, et al.. The screening, identification, design and clinical application of tumor-specific neoantigens for TCR-T cells. *Mol Cancer*. 2023; doi: 10.1186/s12943-023-01844-5.

81. Buonaguro L, Tagliamonte M. Selecting Target Antigens for Cancer Vaccine Development. *Vaccines*. 2020; doi: 10.3390/vaccines8040615.

82. Cavallo F, Calogero RA, Forni G. Are oncoantigens suitable targets for anti-tumour therapy? *Nat Rev Cancer*. 2007; doi: 10.1038/nrc2208.

83. Jiang P, Wang H, Li W, Zang C, Li B, Wong YJ, et al.. Network analysis of gene essentiality in functional genomics experiments. *Genome Biol*. 2015; doi: 10.1186/s13059-015-0808-9.

84. Jeong H, Mason SP, Barabási A-L, Oltvai ZN. Lethality and centrality in protein networks. *Nature*. 2001; doi: 10.1038/35075138.

85. Ning K, Ng HK, Srihari S, Leong HW, Nesvizhskii AI. Examination of the relationship between essential genes in PPI network and hub proteins in reverse nearest neighbor topology. *BMC Bioinformatics*. 2010; doi: 10.1186/1471-2105-11-505.

86. Crosby EJ, Hartman ZC, Lyerly HK. Beyond Neoantigens: Antigens Derived from Tumor Drivers as Cancer Vaccine Targets. *Clin Cancer Res*. 2023; doi: 10.1158/1078-0432.CCR-23-1244.

87. Azhagesan K, Ravindran B, Raman K. Network-based features enable prediction of essential genes across diverse organisms. Mande SC, editor. *PLoS ONE*. 2018; doi: 10.1371/journal.pone.0208722.

88. Hazini A, Fisher K, Seymour L. Deregulation of HLA-I in cancer and its central importance for immunotherapy. *J Immunother Cancer*. 2021; doi: 10.1136/jitc-2021-002899.

89. Taylor BC, Balko JM. Mechanisms of MHC-I Downregulation and Role in Immunotherapy Response. *Front Immunol*. 2022; doi: 10.3389/fimmu.2022.844866.

90. Zaretsky JM, Garcia-Diaz A, Shin DS, Escuin-Ordinas H, Hugo W, Hu-Lieskovan S, et al.. Mutations Associated with Acquired Resistance to PD-1 Blockade in Melanoma. *N Engl J Med*. 2016; doi: 10.1056/NEJMoa1604958.

91. Kalender Atak Z, Gianfelici V, Hulselmans G, De Keersmaecker K, Devasia AG, Geerdens E, et al.. Comprehensive Analysis of Transcriptome Variation Uncovers Known and Novel Driver Events in T-Cell Acute Lymphoblastic Leukemia. Scott HS, editor. *PLoS Genet*. 2013; doi: 10.1371/journal.pgen.1003997.

92. Merck Sharp & Dohme LLC. A Phase 1, Open-Label, Multicenter Study to Assess the Safety and Tolerability of mRNA-5671/V941 as a Monotherapy and in Combination With Pembrolizumab in Participants With KRAS Mutant Advanced or Metastatic Non-Small Cell Lung Cancer, Colorectal Cancer or Pancreatic Adenocarcinoma. clinicaltrials.gov; 2025 Jan. Report No.: NCT03948763.

93. Vita R, Mahajan S, Overton JA, Dhanda SK, Martini S, Cantrell JR, et al.. The Immune Epitope Database (IEDB): 2018 update. *Nucleic Acids Research*. 2019; doi: 10.1093/nar/gky1006.

94. Sayers EW, Cavanaugh M, Clark K, Pruitt KD, Sherry ST, Yankie L, et al.. GenBank 2023 update. *Nucleic Acids Research*. 2023; doi: 10.1093/nar/gkac1012.

95. Wu J, Chen W, Zhou Y, Chi Y, Hua X, Wu J, et al.. TSNAdb v2.0: The Updated Version of Tumor-Specific Neoantigen Database. *Genomics, Proteomics & Bioinformatics*. 2023; doi: 10.1016/j.gpb.2022.09.012.

96. Zhou Z, Wu J, Ren J, Chen W, Zhao W, Gu X, et al.. TSNAD v2.0: A one-stop software solution for tumor-specific neoantigen detection. *Computational and Structural Biotechnology Journal*. 2021; doi: 10.1016/j.csbj.2021.08.016.

97. Wu J, Wang W, Zhang J, Zhou B, Zhao W, Su Z, et al.. DeepHLApan: A Deep Learning Approach for Neoantigen Prediction Considering Both HLA-Peptide Binding and Immunogenicity. *Front Immunol*. 2019; doi: 10.3389/fimmu.2019.02559.

98. Rieder D, Fotakis G, Ausserhofer M, René G, Paster W, Trajanoski Z, et al.. nextNEOpi: a comprehensive pipeline for computational neoantigen prediction. Valencia A, editor. *Bioinformatics*. 2022; doi: 10.1093/bioinformatics/btab759.

99. Di Tommaso P, Chatzou M, Floden EW, Barja PP, Palumbo E, Notredame C. Nextflow enables reproducible computational workflows. *Nat Biotechnol*. 2017; doi: 10.1038/nbt.3820.

100. Huber F, Arnaud M, Stevenson BJ, Michaux J, Benedetti F, Thevenet J, et al.. A comprehensive proteogenomic pipeline for neoantigen discovery to advance personalized cancer immunotherapy. *Nat Biotechnol*. 2025; doi: 10.1038/s41587-024-02420-y.

101. Diao K, Chen J, Wu T, Wang X, Wang G, Sun X, et al.. Seq2Neo: A Comprehensive Pipeline for Cancer Neoantigen Immunogenicity Prediction. *Int J Mol Sci*. 2022; doi: 10.3390/ijms231911624.

102. Lu T, Wang X, Nie W, Huo M, Li S. TransHLA: a Hybrid Transformer model for HLA-presented epitope detection. *GigaScience*. 2025; doi: 10.1093/gigascience/giaf008.

Manuscript with track changes

Click here to access/download;Manuscript;EpitopeGenerationGateway_MS

Epitope Generation Gateway (EGG):

A biological context-dependent approach to cancer vaccine development

Luca Mannino[a], Michele Fratello[a], Jacopo Chiaro[bcde], Federica D'Alessio[bcde], Emanuele Di Lieto[a], Minna Niittykoski[f], Angela Serra[agh], Seppo Ylä-Herttuala[fi], Vincenzo Cerullo[bcdej], Antonio Federico[agh], Dario Greco[ah†]

† Corresponding Author: dario.greco@helsinki.fi

Affiliations:

a: Faculty of Medicine and Health Technology, Tampere University, Tampere, Finland

b: Department of Pharmaceutical Biosciences, University of Helsinki, Faculty of Pharmacy ImmunoViroTherapy Lab, Drug Research Program, Helsinki, Finland

c: Helsinki Institute of Life Science (HiLIFE), University of Helsinki, Helsinki, Finland

d: Translational Immunology Program (TRIMM), Faculty of Medicine Helsinki University, University of Helsinki, Helsinki, Finland

e: Digital Precision Cancer Medicine Flagship (iCAN), University of Helsinki, Helsinki, Finland

f: A.I. Virtanen Institute for Molecular Sciences, University of Eastern Finland, Kuopio, Finland

g: Tampere Institute for Advanced Study, Tampere University, Tampere, Finland

h: Division of Pharmaceutical Biosciences, Faculty of Pharmacy, University of Helsinki, Helsinki, Finland

i: Heart Center and Gene Therapy Unit, Kuopio University Hospital, Kuopio, Finland

j: Department of Molecular Medicine and Medical Biotechnology and CEINGE, Naples University Federico II, Naples, Italy

Abstract

Background

Personalized neoantigen discovery requires tools that not only identify putative epitopes but also prioritize candidates using biological features relevant to tumor fitness and immune escape. Here, we present Epitope Generation Gateway (EGG), a containerized pipeline that streamlines discovery and prioritization across variant types and human leukocyte antigen (HLA) contexts. EGG coordinates established components, Mutect2 for somatic single nucleotide variations (SNVs) and indels, STAR-Fusion for fusion events, ArcasHLA for HLA typing, and pVACtools for epitope sequence prediction, then augments peptide-level scores with biological context. For each patient, EGG builds co-expression and protein-protein interaction networks, uses centrality measures and CRISPR dependency data (DepMap) to flag genes important for tumor fitness, and annotates source genes with Gene Ontology

terms, Hallmarks of Cancer processes, and IntOGen driver status.Personalized neoantigen discovery requires tools that not only identify putative epitopes but also prioritize candidates resilient to tumor immune escape. Here, we present Epitope Generation Gateway (EGG), a containerized pipeline that streamlines discovery and prioritization across variant types and human leukocyte antigen (HLA) contexts. EGG coordinates established components, Mutect2 for somatic single nucleotide variations (SNVs) and indels, STAR-Fusion for fusion events, ArcasHLA for HLA typing, and pVACtools for epitope sequence prediction, then augments peptide-level scores with biological context. For each patient, EGG builds co-expression and protein-protein interaction networks, uses centrality measures and CRISPR dependency data (DepMap) to flag genes important for tumor fitness, and annotates source genes with Gene Ontology terms, Hallmarks of Cancer processes, and IntOGen driver status.

Results

This multi-layer information supports ranking of candidates that are predicted to be presented and that arise from genes essential to tumor fitness or embedded in key pathways, providing a framework to nominate epitopes whose source genes may be less dispensable under immune pressure. As a case study, we applied EGG to 30 TCGA skin cutaneous melanoma (SKCM) patients, demonstrating end-to-end execution and producing prioritized epitopes that emphasize peptides and alterations in high-centrality, dependency-supported essential genes.This multi-layer information supports ranking of candidates that are predicted to be presented and that arise from genes essential to tumor fitness or embedded in key pathways, thereby reducing the likelihood of immune escape by mutations caused by treatment pressure. As a case study, we applied EGG to 30 TCGA skin cutaneous melanoma (SKCM) patients, demonstrating end-to-end execution and producing prioritized epitopes that emphasize peptides and alterations in high-centrality, dependency-supported essential genes.

Conclusions

Overall, EGG links epitope prioritization to tumor systems biology and evolutionary rationale, prioritizing biologically contextualized cancer vaccine candidates from SNVs, indels, fusions, and splicing-derived epitopes while streamlining dataset analysis. EGG is available from GitHub at https://github.com/fhaive/epitope_generation_gateway, registered in bio.tools (biotoolsID: epitope_generation_gateway; https://bio.tools/epitope_generation_gateway) and SciCrunch (RRID: SCR_028792), and archived as a citable workflow in WorkflowHub (DOI: 10.48546/workflowhub.workflow.2165.2).Overall, EGG links epitope prioritization to tumor systems biology and evolutionary dynamics, prioritizing durable cancer vaccine targets from SNVs, indels, fusions, and splicing derived epitopes while streamlining dataset analysis. EGG is available to download at https://github.com/fhaive/epitope_generation_gateway.

Field Code Changed

Introduction

Cancer immunotherapy has emerged as a dominant therapeutic modality in modern oncology, fundamentally transforming treatment paradigms across multiple malignancies. Among the most promising immunotherapeutic approaches, personalized neoantigen vaccines exploit tumor-specific mutations to generate highly targeted immune responses, offering unprecedented precision in cancer treatment [1,2]. Unlike conventional cytotoxic therapies such as chemotherapy and radiotherapy, which broadly target rapidly dividing cells, neoantigen vaccines provide enhanced tumor specificity and may mitigate the systemic toxicity associated with these standard interventions [3].

Field Code Changed

Field Code Changed

The therapeutic efficacy of cancer vaccines essentially depends on their capacity to trigger robust immune responses while maintaining tumor specificity, minimizing off-target effects that could compromise patient safety [4]. Current computational pipelines can identify candidate neo-antigens from SNVs, small insertions or deletions (indels), gene fusions, and aberrant splicing events. Early generation tools primarily targeted SNVs and indels with exclusive focus on Major Histocompatibility Complex (MHC) class I ligand prediction, addressing only $CD8^{+}$ T-cell responses [5–7]. Subsequent frameworks expanded to include gene-fusion breakpoints and now commonly support both class I and class II molecule prediction, engaging both cytotoxic and helper T-cell responses. The most recently developed tools further capture epitopes from aberrant splicing events [8–10]. However, existing approaches largely overlook the functional context of neoantigens, failing to integrate them within their broader transcriptomic landscape or consider the biological significance of their source genes. Moreover, vaccine efficacy is challenged by the tumor intrinsic capacity for immune evasion and the functional importance of targeted mutations. Recent findings by Al Bakir et al. demonstrate that tumors can eliminate immunogenic targets through clonal deletion under selective pressure, emphasizing the critical need to prioritize mutations in functionally indispensable genes [11]. Effective neoantigen selection must therefore consider gene essentiality: alterations in genes essential for malignant fitness or in hub genes anchoring critical signaling pathways are less likely to be purged during tumor evolution, thus representing more durable vaccine targets. Gene co-expression networks provide a practical framework for inferring such functional importance, as network centrality metrics correlate with gene essentiality and the evolutionary cost of mutational escape [12].

Field Code Changed

Field Code Changed

Field Code Changed

Field Code Changed

Field Code Changed

Systems biology treats cancer as a dynamic, complex disease, where phenotype arises from perturbed interactions rather than isolated genes. Network models are widespread tools for representing and analyzing complex systems such as tumor biology. Yet, most neoantigen pipelines still emphasize sequence level features for epitope prioritization (such as peptide MHC binding) and give limited

weight to the broader context that governs whether a candidate peptide is generated, displayed and retained by the tumor. Recent spatial approaches such as SpatialSNV further illustrate that tumor SNVs can vary across local tumor regions and microenvironmental contexts [13]. Evidence shows that subcellular origin influences both presentation and outcome: neoantigens from plasma membrane proteins are disproportionately presented and have been associated with improved responses to immune checkpoint blockade [14,15]~~[13,14]~~. At the same time, tumors can evade immunity by purging dispensable targets during evolution, underscoring the value of prioritizing alterations in vital genes, which are less likely to be lost [11,16,17]~~[11,15,16]~~.

To address these gaps, we developed Epitope Generation Gateway (EGG), an automated workflow that integrates standard callers and predictors: Mutect2 [18]~~[17]~~ for somatic SNVs/indels, STAR-Fusion [19]~~[18]~~ for fusion events, ArcasHLA [20]~~[19]~~ for HLA typing, and pVACtools [21–23]~~[20–22]~~ for class I/II epitope prediction. EGG augments sequence level scoring with essentiality aware features and places each candidate in its biological setting by building patient specific co-expression/protein-protein-interaction (PPI) networks. For prioritization, EGG integrates multiple layers of evidence regarding the source gene. These include patient specific co-expression/PPI centrality measures as network derived indicators of gene importance or essentiality, complemented by CRISPR knockout based dependency scores from DepMap [24]~~[23]~~ that provide experimentally grounded, though not patient-specific, gene essentiality information. Candidate genes are further annotated with Gene Ontology [25]~~[24]~~ terms and MSigDB Cancer Hallmarks [26]~~[25]~~ gene sets to capture pathway-level context, as well as with IntOGen [27]~~[26]~~ annotations to identify established cancer driver genes. Taken together, these data-driven and knowledge based features help to prioritize neoantigens that are not only predicted to be presented but also arise from genes whose functions are critical to tumor fitness and therefore less likely to be lost through immune evasion. EGG's design is intentionally holistic: it integrates patient specific networks with curated biological knowledge (GO, MSigDB Cancer Hallmarks, DepMap) and presentation relevant attributes (cell membrane localization). The goal is a bigger-picture view of each epitope linking peptide level scores to tumor systems biology so that selected targets are immunologically plausible, and evolutionarily durable. EGG is available from GitHub at https://github.com/fhaive/epitope_generation_gateway, registered in bio.tools (biotoolsID: epitope_generation_gateway; https://bio.tools/epitope_generation_gateway) and SciCrunch (RRID: SCR_028792), and archived as workflow in WorkflowHub (DOI: 10.48546/workflowhub.workflow.2165.2).~~EGG has been made available for download from https://github.com/fhaive/epitope_generation_gateway.~~

Formatted: English (United States)
Formatted: Font: (Default) Calibri, English (United States)
Formatted: English (United States)
Formatted: Font: (Default) Calibri, English (United States)
Field Code Changed
Formatted: Font: (Default) +Body (Calibri)
Formatted: Font: (Default) Calibri, English (United States)
Formatted: Font: (Default) +Body (Calibri)
Field Code Changed
Field Code Changed
Formatted: Font: (Default) Calibri, English (United States)
Formatted: Font: (Default) +Body (Calibri)
Field Code Changed
Formatted: Font: (Default) Calibri
Formatted: Font: (Default) +Body (Calibri)
Field Code Changed
Formatted: Font: (Default) Calibri
Formatted: Font: (Default) +Body (Calibri)
Field Code Changed
Formatted: Font: (Default) Calibri, English (United States)
Formatted: English (United States)
Field Code Changed
Formatted: Font: (Default) Calibri, English (United States)
Formatted: Font: (Default) +Body (Calibri)
Field Code Changed
Formatted: Font: (Default) Calibri, English (United States)
Formatted: Font: (Default) +Body (Calibri)
Field Code Changed
Formatted: Font: (Default) Calibri, English (United States)
Formatted: Font: (Default) +Body (Calibri)
Formatted: Font: (Default) Calibri, English (United States)
Formatted: Font: (Default) +Body (Calibri)
Field Code Changed
Field Code Changed

Material and Methods:

Data Acquisition and Preprocessing

Matched tumor RNA-seq, tumor DNA (WES), and normal DNA samples were retrieved from The Cancer Genome Atlas (TCGA) [28][27] for 30 patients diagnosed with cutaneous melanoma (TCGA-SKCM study code). Patients were randomly selected from the subset with available primary tumor RNA-seq data, corresponding tumor DNA, and matched normal DNA, explicitly excluding metastatic tissue. Datasets were retrieved using the Genomic Data Commons (GDC) client (gdc-client) [29][28], leveraging unique patient identifiers (UUIDs).

Sequencing alignment files in BAM format were downloaded and converted to paired-end FASTQ format to facilitate compatibility with the EGG pipeline. Conversion was performed using SAMtools (v1.21) [30,31][29,30], which involved: splitting BAM files by read group, sorting reads by name and extracting paired reads as compressed FASTQ files. These steps ensured the correct input data format for subsequent processing modules.

Read Quality Control and Trimming

Raw sequencing reads from tumor RNA-seq, tumor DNA, and matched normal DNA samples (all in FASTQ format) were processed using FastQC (v0.11.9) [32][31] and fastp (v 0.23.2) [33][32] to assess and improve read quality. Quality assessment was performed with FastQC to identify issues such as base quality degradation. Read trimming and filtering were subsequently carried out using fastp, which automatically detected and removed adapter sequences and excluded reads with poor quality metrics. Specifically, reads containing bases with Phred scores below 20 or with more than 30% unqualified bases were discarded. Additionally, reads shorter than 30 base pairs were removed. A sliding window quality trimming approach with a mean quality threshold of 20 was applied. All trimming settings were specified in a centralized YAML configuration file (QC_filtering_config_0.yaml), allowing users to easily adapt the preprocessing pipeline to different requirements.

RNA-Seq Processing, Fusion Detection and HLA typing

Trimmed cancer RNA-seq reads were processed using STAR-Fusion (v1.15.0) [19][18], which aligned reads and detected gene fusion events using the GRCh38 / GENCODE v37 CTAT genome reference [34,35][33,34]. This tool generated both fusion transcript calls and alignment files for downstream quantification. Reference genomes and transcript annotations (GRCh38 / GENCODE v37) were acquired from the CTAT genome library provided by STAR-Fusion [36][35]. These resources were

Field Code Changed
Formatted: Font: (Default) Calibri, English (United States)
Formatted: Font: (Default) +Body (Calibri)
Field Code Changed
Formatted: Font: (Default) Calibri, English (United States)
Formatted: Font: (Default) +Body (Calibri)
Formatted: Font: (Default) Calibri, English (United States)
Formatted: Font: (Default) +Body (Calibri)
Field Code Changed
Field Code Changed
Formatted: Font: (Default) Calibri, English (United States)
Formatted: Font: (Default) +Body (Calibri)
Field Code Changed
Formatted: Font: (Default) Calibri, English (United States)
Formatted: Font: (Default) +Body (Calibri)
Field Code Changed
Formatted: Font: (Default) Calibri, English (United States)
Formatted: Font: (Default) +Body (Calibri)
Field Code Changed
Formatted: Font: (Default) Calibri, English (United States)
Formatted: Font: (Default) +Body (Calibri)
Field Code Changed
Formatted: Font: (Default) Calibri, English (United States)
Formatted: Font: (Default) +Body (Calibri)

automatically retrieved and managed by the pipeline. STAR-Fusion was used with default filters, which require at least one junction read and one spanning read (or ≥2 junction reads if no spanning support) and a minimum expression support of FFPM (fusion fragments per million total reads) ≥0.1.

Gene expression quantification was performed with the featureCounts function from the Rsubread Bioconductor package (v2.0.8) [37,38]~~[36,37]~~, using the same annotation reference to compute raw read counts for each gene in paired-end mode. These count matrices served as input for sample specific co-expression network production implemented in later stages of the pipeline.

To enable personalized neoepitope prediction, HLA typing was conducted using arcasHLA (v0.5) [20]~~[19]~~, which genotypes both MHC class I and class II loci from RNA-seq data. After sorting the aligned BAM files with SAMtools (v1.21) [30,31]~~[29,30]~~, unmapped reads were extracted and used to predict HLA alleles. The output was converted into a format compatible with pVACtools to support downstream MHC binding predictions.

Both STAR-Fusion and arcasHLA were executed within Docker containers, ensuring reproducibility and consistency across environments. Other steps, including featureCounts and BAM sorting, were integrated into the Snakemake [39]~~[38]~~ pipeline through dedicated Conda environments.

Fusion Epitopes Prediction

Post-alignment RNA fusion calls were processed for neoepitope prediction using the pVACfuse module of pVACtools (v5.3) [22]~~[21]~~, which integrates gene fusion annotations with HLA typing and MHC binding prediction. Fusion predictions, generated by STAR-Fusion, were first annotated using AGFusion (v1.4.3) [40]~~[39]~~ with the Ensembl GRCh38 release 103 transcript database [41]~~[40]~~ and Pfam-A domain annotations [42]~~[41]~~. AGFusion resolved coding fusion junctions and transcript models, generating protein sequences suitable for downstream analysis.

These annotated fusion sequences were then passed to pVACfuse, which identified candidate fusion-derived neoantigens using a user-defined panel of MHC class I and II binding prediction algorithms (MHCNuggetsI and MHCnuggetsII) [43]~~[42]~~. HLA alleles were automatically parsed from ArcasHLA outputs however in the practical use of the pipeline they can be optionally provided as pre-existent files if the user wants to calculate them using different methods. Parameters such as epitope length (e1, e2), binding affinity thresholds, and the number of prediction threads were specified in the Snakemake pipeline and can be modified by the user by using different pVACtools options including different prediction algorithms.

All fusion epitope analyses were executed within Docker containers to ensure version consistency and reproducibility across environments. The containerized pipeline streamlined fusion annotation,

Field Code Changed
Formatted: Font: (Default) Calibri, English (United States)
Formatted: Font: (Default) +Body (Calibri)
Field Code Changed
Formatted: Font: (Default) Calibri, English (United States)
Formatted: Font: (Default) +Body (Calibri)
Formatted: Font: (Default) Calibri, English (United States)
Formatted: Font: (Default) +Body (Calibri)
Field Code Changed
Field Code Changed
Formatted: Font: (Default) Calibri, English (United States)
Formatted: Font: (Default) +Body (Calibri)
Field Code Changed
Formatted: Font: (Default) Calibri, English (United States)
Formatted: Font: (Default) +Body (Calibri)
Field Code Changed
Formatted: Font: (Default) Calibri
Formatted: Font: (Default) +Body (Calibri)
Field Code Changed
Formatted: Font: (Default) Calibri
Formatted: Font: (Default) +Body (Calibri), Finnish
Field Code Changed
Formatted: Font: (Default) Calibri, English (United States)
Formatted: Font: (Default) +Body (Calibri)
Field Code Changed
Formatted: Font: (Default) Calibri, English (United States)
Formatted: Font: (Default) +Body (Calibri)

neoantigen calling, and intermediate file handling, enabling high-throughput and customizable analysis per sample.

SNV and Indel Detection in Tumor and Normal DNA Samples:

All genomic analyses were conducted using the GRCh38 reference genome [35][34]. The reference FASTA file, its associated dictionary, and BWA index files were downloaded from the UCSC Genome Browser [44][43] and generated using SAMtools (v1.16), Genome Analysis Toolkit (GATK, v4.5.0.0) [45,46][44,45] CreateSequenceDictionary, and BWA index [47][46], respectively. Additional resources used in variant calling included a dbSNP [48][47] VCF (Broad Institute, dbSNP build 138), the gnomAD allele frequency panel [49][48], and the GATK-provided Panel of Normals (PON). These files were fetched from Broad Institute cloud repositories (https://gatk.broadinstitute.org/hc/en-us/articles/360035890811-Resource-bundle) and indexed with GATK and tabix [50][49] as needed.

Trimmed DNA-seq reads were processed following the GATK best practices pipeline for short variant discovery. Reads were aligned to the GRCh38 reference genome using BWA-MEM (v0.7.17) [51][50], with read group headers automatically extracted from the FASTQ file headers to include metadata including flowcell ID, lane, and barcode. These values were parsed directly from the first line of each FASTQ file to populate the read group (RG) tags used in alignment. The resulting alignments were sorted by coordinate using SAMtools.

To handle multi lane sequencing data, lane specific BAM files for each sample were merged using Picard (v2.27.4) MergeSamFiles [52][51] Duplicate reads were marked .using GATK MarkDuplicatesSpark to mitigate PCR induced biases. Base quality scores were recalibrated using BaseRecalibrator and ApplyBQSR with known variant sites from dbSNP as training data. These steps produced high-confidence, analysis-ready BAM files per sample.

Alignment metrics, including overall mapping quality and insert size distribution, were collected using GATK CollectAlignmentSummaryMetrics and CollectInsertSizeMetrics respectively. BAM files were indexed using SAMtools to enable efficient access during variant calling and downstream analysis. This preprocessing pipeline was applied to tumor and matched normal DNA samples for each patient, ensuring consistency and compatibility for downstream somatic variant calling with tools such as Mutect2 and HaplotypeCaller.

Germline variants were called on normal DNA samples using GATK HaplotypeCaller, which performs local de novo assembly of haplotypes in regions showing evidence of variation. Raw variant calls were separated into SNPs and indels, followed by hard filtering based on standard GATK quality metrics (e.g., QD, FS, MQ, SOR). Filter-passing variants were selected, sorted, compressed, and concatenated into a

Formatted: Font: (Default) Calibri, English (United States)
Formatted: Font: (Default) +Body (Calibri)
Field Code Changed
Field Code Changed
Formatted: Font: (Default) Calibri, English (United States)
Formatted: Font: (Default) +Body (Calibri)
Formatted: Font: (Default) Calibri
Formatted: Font: (Default) +Body (Calibri), Finnish
Field Code Changed
Formatted: Font: (Default) Calibri, English (United States)
Formatted: Font: (Default) +Body (Calibri)
Field Code Changed
Field Code Changed
Formatted: Font: (Default) Calibri, English (United States)
Formatted: Font: (Default) +Body (Calibri)
Field Code Changed
Formatted: Font: (Default) Calibri, English (United States)
Formatted: Font: (Default) +Body (Calibri)
Field Code Changed
Formatted: Font: (Default) Calibri, English (United States)
Formatted: Font: (Default) +Body (Calibri)
Field Code Changed
Formatted: Font: (Default) Calibri, English (United States)
Formatted: Font: (Default) +Body (Calibri)
Field Code Changed
Formatted: Font: (Default) Calibri, English (United States)
Formatted: Font: (Default) +Body (Calibri)

single filtered germline VCF file per sample using BCFtools (v1.16) [30][29] and tabix utilities. These high-confidence germline calls were retained for downstream annotation and comparison against somatic variants.

Somatic variants were identified using GATK Mutect2 by jointly analyzing tumor and matched-normal BAM files per sample. The analysis incorporated a germline resource (gnomAD) and a panel of normals to reduce false positives. Mutect2 output included both raw variant calls and strand orientation artifact data (F1R2).

To refine variant calls, contamination estimates were calculated using GATK GetPileupSummaries and CalculateContamination, followed by orientation bias modeling with LearnReadOrientationModel. Final variant filtering was carried out using FilterMutectCalls, integrating all calculated metrics as additional flags in the VCF for each sample.

Genomic Variant Annotation and Epitope Prediction

To enable downstream neoepitope prediction using pVACtools, we performed additional annotation and preprocessing of somatic and germline variant calls. Variant Effect Predictor (VEP, Ensembl release 103) [53][52] was used to functionally annotate VCF files from somatic and germline variant calling workflows. VEP was configured in offline mode using the GRCh38 cache and a subset of plugins (e.g., Frameshift, Wildtype) downloaded from the Ensembl GitHub repository. Annotations were run via command-line within a Docker environment using the griffithlab/pvactools container (v5.3). The pipeline was configured to automatically pull the Docker image if not already present. Somatic VCFs (from Mutect2) and filtered germline VCFs (from HaplotypeCaller) were annotated with VEP, producing functionally enriched VCF files for subsequent pVACseq input. While downstream neoepitope analysis focused on somatic mutations, filtered germline variants were obtained to support phasing. These variants were merged with somatic calls and jointly phased using GATK ReadBackedPhasing. This step allowed identification of compound heterozygous or cis/trans configured variants that could influence peptide sequences derived from cancer genomes.

In parallel, gene expression levels were quantified from tumor RNA-seq data using Kallisto (v0.46.1) [54][53], a lightweight pseudoalignment-based transcript quantifier. A Kallisto index was built from Ensembl cDNA reference transcripts (release 103), and tumor RNA reads from multiple sequencing lanes were jointly quantified. The resulting expression data was integrated into the annotated somatic VCF using vcf-expression-annotator (vatools package v5.2.0), aligning expression values with corresponding variants.

Formatted: Font: (Default) Calibri, English (United States)

Formatted: Font: (Default) +Body (Calibri)

Field Code Changed

Field Code Changed

Formatted: Font: (Default) Calibri, English (United States)

Formatted: Font: (Default) +Body (Calibri)

Formatted: Font: (Default) Calibri, English (United States)

Formatted: Font: (Default) +Body (Calibri)

Field Code Changed

To generate VCFs compatible with pVACtools, tumor-only variants were extracted using GATK SelectVariants. To enable inclusion of germline variants in neoepitope screening, sample identifiers in the germline VCFs were harmonized with the tumor VCF using BCFtools (v1.17). Germline and tumor variants were then merged using GATK CombineVariants and sorted using Picard SortVcf. These combined VCFs were phased using GATK ReadBackedPhasing and re-annotated with VEP to account for phasing effects on coding consequence.

Human leukocyte antigen (HLA) types were obtained via ArcasHLA as described earlier. HLA types were automatically extracted from ArcasHLA genotype JSON files and formatted into a pVACtools compatible input structure. Alternatively, users have the option to supply their own HLA typing externally.

The primary pVACseq analysis was then performed using the pvacseq run command inside the griffithlab/pVACtools Docker container (v5.3). The workflow supported all pVACseq-compatible MHC binding prediction algorithms, and for this analysis specifically used MHC_nuggetsI, and MHCnuggetsII. Both phased variant calls and Kallisto derived RNA expression data were supplied to enhance neoepitope prioritization. All pVACseq parameters are user configurable by modifying the Snakemake script directly. For this analysis, we used the following key parameters: --epitope-lengths 8-15 for class I predictions and --epitope-lengths 11-30 for class II, along with --depth 1000, and --phased-proximal-variants-vcf to incorporate phasing effects. For tumour-purity-dependent tiering, we retained the pVACseq default tumour purity value of 1.0; this parameter is user-configurable and can be adjusted when sample-specific tumour purity estimates are available. In this case study, retaining tumour purity = 1.0 provided a conservative variant allele frequency based parameter for identifying tumour-relevant somatic candidates. Somatic SNV and indel calls were generated with Mutect2 followed by Mutect2 post-filtering. For the proof-of-concept analysis presented here, both PASS and non-PASS Mutect2 records were retained as pVACseq input to preserve a broad candidate mutation pool before downstream prioritisation. To assess the impact of this choice, we annotated final somatic epitope candidates with the VCF FILTER status of the originating variant and repeated the prioritisation using PASS-only somatic pVACseq inputs. This sensitivity analysis showed that non-PASS calls contributed minimally to the final prioritised candidates and did not materially affect the prioritised gene set. PASS-only filtering can be applied before pVACseq when a higher-specificity input set is desired. Full details of the PASS-only sensitivity analysis are provided in Additional file 3.~~Somatic Mutect2 calls were filtered (via Mutect2's post-filtering), but variants flagged by filters (i.e. non-PASS) were not excluded both flagged and PASS variants were retained. This decision was made deliberately to capture the broadest possible mutation pool as a proof-of-concept, enabling EGG's downstream~~

Formatted: English (United States)

Formatted: English (United States)

prioritization framework to demonstrate its capacity to refine even heterogeneous candidate sets arising from a relatively small (n=30) patient cohort.

Mutect2 and STAR-Fusion were selected as pragmatic single-caller defaults for somatic SNV/indel and fusion detection, respectively. This choice was intended to keep the case-study workflow lightweight, reproducible, and computationally tractable, while relying on established tools with broad use in cancer genomics. Mutect2 is used in the GATK somatic short-variant discovery workflow for SNV and indel detection, and is widely regarded as an established, reliable somatic variant caller in cancer genomics [18,55]. STAR-Fusion is used in the NCI Genomic Data Commons RNA-seq harmonization workflow for transcript-fusion detection and has been reported among the most accurate and fastest fusion callers in benchmark analyses of cancer transcriptomes[56]. We acknowledge that consensus calling strategies can further improve input precision and reduce dependence on caller-specific thresholds; however, benchmarking alternative caller combinations was outside the scope of the present study, which focuses on evaluating EGG as an integrated neoantigen prediction and prioritisation framework.

Alternative Splicing-Derived Neoepitope Prediction

Tumor-specific alternative splicing events were identified and processed for neoepitope prediction using a combination of RegTools (v1.0.0) [57][54] and pVACsplice from the pVACtools suite. First, splice junction annotations were extracted from tumor RNA-seq alignments using RegTools, which identifies novel splice sites and exon-skipping events by comparing observed junctions against reference transcript models.

The analysis was based on the GRCh38/GENCODE v37 reference annotation to identify putative tumor-specific splicing variants. Junction files obtained in output were filtered to retain high-confidence events based on read support thresholds and junction quality metrics.

Identified splicing events were then exploited for neoepitope prediction using pVACsplice (from package pVACtools v5.3), which generates peptide sequences spanning novel exon-exon junctions and predicts their MHC binding potential. The workflow required both the RegTools junction output and the corresponding genomic VCF file containing somatic variants to account for potential interactions between splicing alterations and point mutations.

HLA alleles were provided either from ArcasHLA outputs or user-specified files, maintaining consistency with other neoepitope prediction modules. pVACsplice analysis employed the same MHC binding prediction algorithms used in pVACseq (MHCnuggetsI and MHCnuggetsII) to ensure

Formatted: English (United States)

Formatted: Font: (Default) Calibri, English (United States)

Formatted: English (United States)

Formatted: Font: (Default) Calibri, English (United States)

Formatted: English (United States)

Field Code Changed

Formatted: Font: (Default) Calibri, English (United States)

Formatted: Font: (Default) +Body (Calibri)

comparable scoring across different neoantigen sources. Epitope lengths were set to 8-15 amino acids for MHC class I and 11-30 for MHC class II predictions.

All splicing-derived neoepitope analyses were executed within the griffithlab/pvactools Docker container to maintain version consistency and reproducibility. Parameters including minimum junction read support, binding affinity thresholds, and prediction algorithms were configurable through the centralized Snakemake workflow.

Patient Specific Networks Generation

Gene level raw read count matrices previously produced in the RNA-seq processing step (described in the RNA-Seq Processing, Fusion Detection and HLA typing paragraph) were carried forward for patient-specific co-expression network generation. Gene level raw read count matrices previously produced in the RNA-seq processing step (described in the RNA-Seq Processing, Fusion Detection and HLA typing paragraph) were carried forward for patient-specific co-expression network generation. Genes were retained for network construction only if they had at least 15 raw reads in at least 20% of samples; all other genes were removed as a low-expression preprocessing step (in our cohort of 30 samples, this corresponds to requiring detectable expression in at least 6 samples). This filtering step was applied to reduce noise from genes with very low or infrequent expression, which are less likely to support stable correlation estimates in co-expression-based network inference. similar low-expression filtering steps are commonly used in RNA-seq network analysis workflows, including LIONESS-based analyses of TCGA and GTEx data [58,59].Genes with low expression (fewer than 15 reads in at least 20% of samples) were filtered out to reduce noise.

For downstream network analysis, variance stabilizing transformation (VST) was applied to the expression count using the DESeq2 package (v1.46.0) [60][55]. This transformation adjusts for the strong relationship between gene expression level and variability, producing a matrix in which genes with different expression ranges can be more reliably compared. The resulting normalized expression matrix was used to construct patient-specific co-expression networks.

To estimate individual level gene co-expression patterns, we applied the LIONESS (Linear Interpolation to Obtain Network Estimates for Single Samples) framework [61][56]. For a set of SN samples, the aggregate network was computed using Pearson correlation between all gene pairs (i, j) across the full expression matrix.

To estimate the network specific to a sample q, we first, denote the aggregate network computed across all samples as $e_{ij}^{(\alpha)}$, and the corresponding leave-one-out aggregate network excluding sample

Formatted: English (United States)

Formatted: English (United States)

Formatted: English (United States)

Formatted: English (United States)

Formatted: English (United States)

Formatted: Font: (Default) Calibri, English (United States)

Formatted: Font: (Default) Calibri, English (United States)

Formatted: Font: (Default) +Body (Calibri)

Field Code Changed

Field Code Changed

Formatted: Font: (Default) Calibri, English (United States)

Formatted: Font: (Default) +Body (Calibri)

Formatted: English (United States)

Formatted: English (United States)

$q$as $e_{ij}^{(\alpha-q)}$ computed the leave-one-out network $e_{ij}^{(\alpha-q)}$, constructed from all samples except q. The individual sample network $e_{ij}^{(q)}$ was then derived using the LIONESS formula:

$$e_{ij}^{(q)} = SN\left(e_{ij}^{(\alpha)} - e_{ij}^{(\alpha-q)}\right) + e_{ij}^{(\alpha-q)}$$

This equation quantifies the contribution of sample q. to each edge (i, j) by interpolating its influence relative to the full dataset. All sample-specific adjacency matrices were stored in sparse matrix format to facilitate downstream graph based analyses.

Following LIONESS based reconstruction, we filtered the sample-specific networks to prioritize biologically interpretable and functionally relevant edges. First, we intersected each network with an extended PPI network the HumanNet-XN v2, an integrated human functional interaction network anchored in protein-protein interactions and extended with co-expression, pathway, interolog and co-citation evidence [62][57]. HumanNet edges were mapped from Entrez to Ensembl gene identifiers using the org.Hs.eg.db library (v3.18.0) [63][58].

To construct the filtered networks, we applied a two-step edge selection strategy that combined the extended PPI informed filtering with membrane-focused expansion:

1. PPI-informed edge selection: For each gene, we retain edges with absolute correlation weights ranking in the top 20% among all edges connecting that gene to its PPI-linked neighbors. This approach prioritized patient-specific co-expression patterns that aligned with known protein-protein interactions.
2. Membrane-enriched network expansion: To supplement the PPI-filtered network, we incorporated additional edges from the gene co-expression network that involved membrane-associated genes. In the default configuration, candidate membrane-associated edges were defined as edges in which at least one endpoint gene was annotated as membrane associated. These candidate edges were ranked by absolute correlation weight, and the top-ranked edges were added to the PPI-filtered network. The number of membrane-associated edges added was controlled by a configurable proportion, set to 5% by default. In the analysis presented here, this proportion was calculated relative to the number of weighted HumanNet/PPI edges before the per-gene top-20% PPI filtering step.Specifically, we first applied the PPI filtering described above, which yielded N edges. We then calculated 5% of N to determine the target number of additional edges to include. From the full gene co-expression network, we selected the top-ranked edges (by absolute correlation weight) where at least one gene was annotated

Formatted: English (United States)

Formatted: Font: (Default) Calibri, English (United States)

Formatted: Font: (Default) +Body (Calibri)

Field Code Changed

Field Code Changed

Formatted: Font: (Default) Calibri, English (United States)

Formatted: Font: (Default) +Body (Calibri)

Formatted: English (United States)

as membrane associated, up to this 5% threshold. This strategy enriched the network for membrane localized genes while maintaining focus on the strongest co-expression relationships. Membrane associated genes were identified using Gene Ontology (GO) annotations from the following terms and their descendant terms in the GO hierarchy:

- GO:0005886 (*plasma membrane*)
- GO:0005887 (*integral component of plasma membrane*)
- GO:0046658 (*anchored component of membrane*)
- GO:0009897 (*external side of plasma membrane*)
- GO:0009986 (*cell surface*)

Membrane gene annotations were obtained from the GAF file provided by the GO Consortium and mapped to Ensembl IDs via UniProt [64][59] and BioMart (v2.58.0) [65][60].

Formatted: Font: (Default) Calibri, English (United States)

Field Code Changed

Formatted: Font: (Default) +Body (Calibri)

Field Code Changed

Formatted: Font: (Default) Calibri, English (United States)

Formatted: Font: (Default) +Body (Calibri)

Both the PPI informed edge selection and the membrane-enriched network expansion are optional; the corresponding percentages can be modified via the configuration files or effectively switched off. The final output consisted of sparse adjacency matrices representing filtered, patient-specific co-expression networks enriched for interactions with known biological support and immunological relevance. We have named these networks: Patient Specific Functional Interaction Networks (pFINs) These network matrices were saved in Matrix Market format to ensure compatibility with external graph analysis frameworks.

The pFINs exhibited a characteristic topology consisting of a dominant largest connected component (LCC) alongside smaller, isolated subnetworks. The LCC encompassed the vast majority of edges and nodes, forming a cohesive structure where genes were interconnected through direct or indirect relationships. The remaining nodes formed small, disconnected clusters or isolated pairs that did not connect to the main component. This topology, with most network connectivity concentrated in a single large component, provided a natural framework for assessing gene importance: genes critical to network integrity would be those whose removal disrupted the LCC structure most severely.

Centrality Metrics for Gene Prioritization

To evaluate the functional relevance and network prominence of candidate neoantigen source genes, we calculated node level centrality scores within each sample-specific co-expression network.

To compare the relative connectivity of genes, we computed the Weighted Connectivity Impact (WCI) for each node *v*, defined as the fraction of total network edge weights attributable to its immediate connections.

Where each edge is normalized as:

$$\widetilde{w_{uv}} = \frac{|w_{uv}|}{\max_{i,j}|w_{ij}|}$$

The numerator is the sum of normalized absolute weights of all edges incident to *v*:

$$\text{strength}(v) = \sum_{u \in N(v)} \widetilde{w_{uv}}$$

Weighted Connectivity Impact calculated as:

$$\text{WCI}(v) = \frac{\text{strength}(v)}{\sum_{e \in E} \widetilde{w_e}}$$

This yields a sample-scaled centrality score between 0 and 1, allowing for cross-sample comparisons of gene connectivity despite differences in total edge weight. Genes with high WCI constitute a disproportionately large proportion of overall network strength.

pFINs Centrality Metrics

To assess the topological relevance of neoantigen-associated genes within the pFINs networks, we computed several centrality metrics.

The first centrality metric is the Largest Component Impact (LCI) quantifies a gene's contribution to the integrity of the network's Largest Connected Component (LCC) obtained from the PPI filtering steps explained above. It is a simple way to combine strength and degree in a comparable way between different networks. For each gene *v*, the LCI was computed by measuring the change in both node connectivity and edge weight upon virtual deletion of that gene:

$$\text{Largest Component Impact}(v) = \frac{1}{2}\left[\frac{S_0 - S_v}{S_0}\right] + \frac{1}{2}\left[\frac{W_0 - W_v}{W_0}\right]$$

Where:

- S_0 is the number of nodes in the LCC of the original network,
- $S_v$is the size of the LCC after removing node *v*,

- W_0 is the sum of normalized absolute edge weights within the original LCC,
- W_v is the corresponding sum after removing *v*.

This metric captures both structural fragmentation and, in turn, functional disconnection, identifying nodes whose removal disproportionately disrupts network integrity. Conceptually, LCI relates to percolation theory, where the removal of critical nodes can trigger a phase transition that fragments a network into disconnected components. By quantifying the impact of individual gene removals on the LCC, LCI identifies genes that act as critical connectors maintaining network cohesion.

The second centrality metric computed was betweenness centrality, a dynamic node centrality measure that reflects how information propagates through the networks. In addition, we calculated node degree and weighted strength for each gene, with strength defined as the sum of the absolute weights of its incident edges using igraph (v2.0.3) [66][61].

Pan-cancer Gene Essentiality Scoring

To incorporate gene biological relevance into neoantigen prioritization, we obtained pan-cancer essentiality scores from the DepMap Public 24Q4 dataset [24][23]. The CRISPR gene effect matrix (CRISPRGeneEffect.csv) was downloaded from the Broad DepMap Figshare repository (article ID 27993248) and processed in R. This matrix contains Chronos-derived gene effect values that represent copy number corrected knockout fitness effects across cancer cell lines, where more negative values indicate greater cellular dependency [67][62].

For each gene, we computed the median Chronos gene effect across all available cell lines to derive a single pan-cancer essentiality score (PanCancer_Median_Chronos). Following DepMap guidelines, genes with scores < -0.5 indicate depletion in most cell lines, while scores < -1.0 represent strong essentiality. These pan-cancer essentiality scores were subsequently integrated into the epitopes prioritization workflow to favor epitopes derived from functionally necessary genes.

Cancer Driver Gene Annotation

To identify neoantigens derived from cancer driver genes, we obtained driver annotations from the IntOGen Drivers compendium (version 20230531) [27][26]. The compendium file was processed to retain all genes classified as either “activating” (oncogenic; Act) or “loss-of-function" (tumor suppressor; LoF) roles across cancer types. This annotation was integrated into the neoantigen prioritization framework to identify neoantigens originating from genes with established roles in cancer pathogenesis, as mutations in driver genes may be particularly relevant for immune targeting strategies.

Formatted: Font: (Default) Calibri, English (United States)

Field Code Changed

Formatted: Font: (Default) +Body (Calibri)

Field Code Changed

Formatted: Font: (Default) Calibri, English (United States)

Formatted: Font: (Default) +Body (Calibri)

Formatted: Font: (Default) Calibri, English (United States)

Formatted: Font: (Default) +Body (Calibri)

Field Code Changed

Field Code Changed

Formatted: Font: (Default) Calibri, English (United States)

Formatted: Font: (Default) +Body (Calibri)

Consensus Prioritization

Candidate neoantigens were prioritized using a weighted Borda rank aggregation implemented in R. For each feature, epitopes were ranked according to expected directionality: ascending for Median.MT.IC50.Score and Depmap_survivability_score, and descending for network metrics (Network_Betweenness, Network_Degree, Network_Impact, Network_Strength, Network_WCI). Feature-specific ranks were converted to Borda points and combined using predefined weights (default: 0.25 each for Median.MT.IC50.Score and Depmap_survivability_score; 0.10 for each network metric). The aggregate Borda score determined the final ordering, with ties resolved by average rank. Missing feature values were ignored and remaining weights renormalized per epitope. The prioritization (features, weights, rank direction, tie rule, and NA handling) is configurable via a YAML file without code changes, allowing emphasis on binding affinity, network centrality, or gene essentiality as needed.

Results and Discussion

EGG analytical flow

The complexity of cancer immunotherapy requires sophisticated computational approaches that can identify not just any neoantigens, but those most likely able to ~~elicit appropriate immune responses and to~~ remain resistant to evolutionary changes in the cancer. To address this challenge, we developed a comprehensive pipeline that integrates both established algorithms to extrapolate tumor specific neoantigens and novel network-based methodologies to improve neoantigen prioritization based on their biological context. On one hand, our pipeline includes quality control, preprocessing, extensive mutational analysis, transcriptomics data analysis with identification of fusion events, alternative splicing, and HLA typing. The additional value in this study is that we developed novel network-based methods to infer the biological context of the identified neoantigens. Such methods include the inference, manipulation and analysis of sample specific molecular networks. We envision that by integrating data driven interactional properties of network models with prior knowledge from multiple public databases, it is possible to achieve a robust and informed prioritization that improves efficacy and streamlines pre-clinical pipelines.

The EGG pipeline comprises seven modules ranging from quality control and data preprocessing to interpretable ranking of neoantigens. The diagram in Figure 1 provides a detailed overview of the pipeline structure as well as the included tools. The first module, Module 0, is dedicated to quality control and filtering of the raw sequencing reads using fastp [33] for read trimming and fastqc for quality assessment. The subsequent mutation detection module (Module 1A) analyzes paired tumor-normal DNA samples, utilizing Mutect2 [18] for somatic variant identification and HaplotypeCaller [45]

Formatted: Font: (Default) Calibri, English (United States)

Formatted: Font: (Default) Calibri, English (United States)

Formatted: Font: (Default) Calibri, English (United States)

for germline variant detection Module 1B, on the other hand, processes trimmed RNA-seq cancer data through three analytical pathways: STAR-Fusion [19] identifies structural variants, arcasHLA [20] determines patient-specific HLA allotypes, and FeatureCounts (from Rsubread) [37] generates expression profiles which are then used in network-based downstream analysis. Somatic variants identified in Module 1A undergo allele-specific epitope prediction in Module 2A, where the pVACseq [23] framework evaluates SNVs and indels for MHC-binding potential. This module also performs preprocessing steps required by pVACseq, including quantification of transcripts with Kallisto [54] and phasing variants by integrating somatic and germline calls to preserve haplotype context. Fusion transcripts detected by STAR-Fusion are analyzed in Module 2B where pVACfuse [22] is used to predict neo-antigens spanning fusion junctions.

Module 2C extracts alternative splicing events by validating variants occurring at splicing sites obtained from Module 1A, via Mutect2, by applying RegTools [57] identifying tumor-specific splicing events, and subsequently processing these through pVACsplice [21] to predict splice junction derived epitopes.

Finally, the single-sample network module constructs personalized co-expression networks using the LIONESS [61] methodology on Pearson gene co-expression matrices derived from the RNA-seq counts generated in Module 1B. The resulting networks are filtered using HumanNet-XN [62] protein-protein interaction data, and cellular membrane proteins are identified via Gene Ontology [25]. Network centrality metrics are computed for both filtered and unfiltered patient-specific networks and are used, together with relevant biological information, to prioritize candidates with high potential therapeutic relevance. The network related calculation and generation is carried out by the network generation module while the final prioritization is carried out by the epitope prioritization module. Additional information for the execution of the pipeline, including details on the implementation (e.g., Snakemake workflow [39]), retrieval of required references and tools, and execution of individual modules, is provided in Additional file 1 (Supplementary Material). Additional file 1 also includes an overview of the main third-party tools integrated in EGG and a computational resource benchmark of the main EGG-specific components, focusing on patient-specific network generation and epitope prioritisation rather than upstream third-party tools whose resource requirements depend strongly on sequencing depth, library size, and mutation burden.~~the retrieval of required references and tools, as well as the execution of individual modules, are provided in Additional file 1 (Supplementary Material).~~

Figure 1. Epitope Generation Gateway Workflow. The EGG pipeline processes matched normal/tumor DNA (WGS/WES) and tumor RNA-seq data through seven integrated modules. Following quality control, DNA undergoes BWA-MEM [51] alignment and GATK processing for germline and somatic variant calling. RNA processing includes STAR alignment, gene expression quantification, fusion detection (STAR-Fusion), and alternative splicing analysis. HLA typing and variant annotation are performed in parallel. Sample-specific co-expression networks are constructed using filtered

Formatted: Font: (Default) Calibri, English (United States)

Formatted: Font: (Default) Calibri, English (United States)

Formatted: Font: (Default) Calibri, English (United States)

Formatted: Font: (Default) Calibri, English (United States)

Formatted: Font: (Default) Calibri, English (United States)

Formatted: Font: (Default) Calibri, English (United States)

Formatted: Font: (Default) Calibri, English (United States)

Formatted: Font: (Default) Calibri, English (United States)

Formatted: Font: (Default) Calibri, English (United States)

Formatted: Font: (Default) Calibri, English (United States)

Formatted: Font: (Default) Calibri, English (United States)

Formatted: Font: (Default) Calibri, English (United States)

Formatted: English (United States)

Formatted: Justified

Formatted: Font: (Default) Calibri, English (United States)

HumanNet-XN and LIONESS methodology. Neoantigen prediction targets three categories: SNV/indel, fusion, and splicing neoantigens, all with MHC class I/II binding predictions. Network centrality analysis computes connectivity metrics for integrated prioritization combining MHC binding, centrality scores, and immunogenicity to generate prioritized neoantigens with biological context. Dashed boxes represent pipeline modules subdivision. Color scheme: blue boxes represent input data, orange boxes represent intermediate analyses or results, purple boxes denote analyses whose outputs contribute to the consensus scoring, the red box represents the consensus prioritization step, and the green box indicates the final output of prioritized epitope tables.

Inter sample variability in somatic mutations relevant to neoantigens

To demonstrate EGG's comprehensive neoantigen prediction capabilities, we analyzed the mutational landscape of 30 TCGA skin cutaneous melanoma (SKCM) samples [28]. As a descriptive view of the mutational landscape of the samples, we highlight the subset of somatic variants identified by Mutect2 that passed GATK's contamination and quality filters and were annotated as protein-coding, cancer-specific alterations (Figure 2).

Formatted: Font: (Default) Calibri, English (United States)

Specifically, we identified three classes of mutations potentially giving rise to cancer neoantigens, including SNVs (including splicing site modifiers), indels, and gene fusions. Our analysis revealed substantial inter-sample variability in mutational burden across all these three genomic alteration categories. As widely reported, including TCGA SKCM and subsequent WGS studies, SNVs are the predominant mutation class in melanoma reflecting UV-driven mutagenesis [68,69][63,64]. This is consistent with our results where we found 12,000-59,000 somatic SNVs, though one sample (TCGA-BF-AAOX) exhibited the highest burden at ~9,4005,300 variants (Figure 2A). While indel frequencies were generally lower, ranging from <50 15 to ~300 100 events per sample, with TCGA-EB-A3XF displaying the highest burden at 286 155 indels (Figure 2B). Gene fusion events, detected through STAR-Fusion analysis of tumor RNA-seq data, showed the lowest absolute frequencies with 2-24 fusion events identified per sample (Figure 2C). These results confirmed that while SNVs dominate the mutational landscape (around 90% of all variants), also indels (around 5-10%) and gene fusions (usually less than 2-3% per sample) contribute meaningfully to the total neoantigen repertoire (Figure 2D). EGG integrates several tools to carry out a comprehensive mutational profiling, allowing the users to process multiple classes of genomic alterations relevant to downstream neoantigen discovery[LM3.1].The tools embedded in the EGG workflow are reported in Additional File 1 Supplementary Material. This comprehensive mutational profiling demonstrates the ability of EGG to capture the full spectrum of genomic alterations that can generate immunologically relevant neoantigens, positioning it as a robust tool for personalized cancer immunotherapy target identification.

Formatted: Font: (Default) +Body (Calibri)

Field Code Changed

Formatted: Font: (Default) Calibri, English (United States)

Formatted: English (United States)

Figure 2. Mutational landscape analysis across TCGA samples processed by the EGG pipeline. (A) Single nucleotide variant (SNV) counts per sample, showing variable mutational burden with the sample TCGA-BF-AAOX being highly mutated (~59,3400 SNVs). (B) Indel distribution, with most samples displaying fewer than 100 events and the sample TCGA-EB-A3XF showing the highest 286155 indels. (C) Gene fusion events, range between 2-24 per sample. (D) Proportion of SNVs, Indels and Fusion events per sample displayed as boxplots.

Neoantigen burden distribution across mutation types

Neoantigen discovery modules of EGG, implemented via the pVACtools [22] suite, identified candidate epitopes derived from somatic variants (pVACseq), gene fusions (pVACfuse with STAR-Fusion input), and aberrant splicing events (pVACsplice with RegTools input) across the 30 samples selected from the TCGA melanoma cohort. Within the considered sample cohort, we identified up to 20240 epitopes in a single sample across the three mutational classes (Figure 3A–3C). The binding affinity of epitopes to the MHC class I and class II complexes were computed. MHC class II slightly outnumbered class I predictions, consistently with the broader binding motif diversity of class II molecules. These results highlight the heterogeneity of neoantigen repertoires across patients and mutation types. To determine whether this heterogeneity simply reflected differences in input event burden, we correlated candidate neoantigen counts with the corresponding numbers of somatic variants and fusion events across the 30 TCGA-SKCM samples (Additional file 3, Supplementary Figure S1A–C). For somatic variant-derived candidates, the total neoantigen count showed only a weak and non-significant positive correlation with SNV count (Spearman $\rho = 0.26$, p-value = 0.159) and with combined SNV plus indel count ($\rho = 0.29$, p-value = 0.124). Similar weak, non-significant associations were observed when MHC classes were analyzed separately, with SNV plus indel burden correlating weakly with both MHC class I candidates ($\rho = 0.27$, p-value = 0.143) and MHC class II candidates ($\rho = 0.31$, p-value = 0.100). Thus, the variability in mutation-derived neoantigen yield was not explained by SNV burden alone. By contrast, fusion-derived neoantigen counts were strongly associated with the number of detected fusion events. Total fusion-derived candidate counts correlated positively with fusion event burden ($\rho = 0.72$, p-value = 7.29×10^{-6}), and this relationship was observed for both MHC class I ($\rho = 0.65$, p-value = 1.05×10^{-4}) and MHC class II candidates ($\rho = 0.73$, p-value = 5.37×10^{-6}). Together, these analyses show that EGG's neoantigen output depends on both the number and type of underlying genomic alterations, but is not a simple linear consequence of SNV burden. Candidate peptide yield is also influenced by alteration consequence, altered protein sequence length and context, patient-specific HLA genotype and MHC class.

Formatted: Font: (Default) Calibri, English (United States)

Formatted: English (United States)

Formatted: English (United States)

Formatted: English (United States)

Formatted: English (United States)

Formatted: English (United States)

Formatted: English (United States)

Formatted: English (United States)

Formatted: English (United States)

Formatted: English (United States)

Formatted: English (United States)

Formatted: English (United States)

Formatted: English (United States)

Formatted: English (United States)

Formatted: English (United States)

Formatted: English (United States)

Since EGG jointly considers peptides predicted to bind HLA class I and HLA class II alleles, it captures candidate neoantigens that could contribute to cytotoxic CD8⁺ T-cell responses (class I) as well as helper CD4⁺ T-cell responses (class II), two complementary arms of anti-tumor immunity thought to be important for durable responses. Moreover, by adding sample-specific co-expression network analysis as a second-tier prioritization, EGG reduces large candidate pools into a smaller set of biologically relevant and therapeutically promising targets, bridging the gap between computational prediction and the practical need to select a limited number of epitopes for inclusion in a vaccine formulation.~~and the practical constraints of vaccine formulation.~~

Formatted: English (United States)

Figure 3. Predicted neoantigen landscape across TCGA melanoma samples processed by EGG. (A) Candidate neoantigens derived from somatic variants using pVACseq, stratified by MHC class (I and II). Samples yielded up to 224~~00~~ epitopes, with somatic mutations contributing the largest overall pool. (B) Candidate neoantigens derived from gene fusions using pVACfuse with STAR-Fusion input, showing smaller but variable contributions across samples. (C) Candidate neoantigens derived from aberrant splicing events.

Integrating gene essentiality signals into epitope candidate ranking

Despite current methods are capable of identifying a remarkable amount of cancer neoantigen arising from genomic events, such as somatic mutations, fusions, and alternative splicing, translating this abundance into a clinically actionable candidates remains challenging. Therapeutic cancer vaccines typically deliver only a limited epitope set per patient, most peptide or mRNA platforms administer usually between 10 to 20 epitope sequences, whereas recent DNA plasmid vaccines have encoded larger payloads (up to 40 epitopes) in combination with PD-1 blockade. These design choices reflect manufacturing constraints, immunogenicity considerations, and trial feasibility [70–72]~~[65–67]~~. Although sequencing-based pipelines can yield dozens to hundreds of candidate epitopes per tumour after standard HLA complex binding filters, current vaccine platforms can only accommodate a small subset of these in a single formulation. As a result, personalized vaccine design is not only an epitope level selection problem but also a gene level one: when only 10 to 40 epitopes can be used, choosing which genes they represent becomes necessary [2,75]~~[2,70]~~. Prioritising epitopes from genes that are embedded in central, functionally indispensable positions within tumour networks offers a principled way to bias these limited slots toward targets that may be harder for the tumour to downregulate or eliminate under immune pressure [4,76,77]~~[4,71,72]~~.

Field Code Changed

Formatted: Font: (Default) Calibri, English (United States)

Field Code Changed

Field Code Changed

Formatted: Font: (Default) Calibri, English (United States)

Formatted: Font: (Default) +Body (Calibri)

Field Code Changed

Formatted: Font: (Default) Calibri, English (United States)

Formatted: Font: (Default) +Body (Calibri)

In this study, we hypothesise that an additional layer of biological contextualisation is required to rationally select effective epitopes that are, in turn, clinically relevant for the specific patient. To address this, EGG integrates a network based filtering strategy that captures patient specific gene expression patterns and exploits several sources of prior knowledge to refine the search for cancer epitopes. Specifically, EGG builds co-expression networks that are representative of the patient-specific gene expression combinations. Such a network is modelled and refined by using experimentally validated protein-protein interactions and membrane protein annotations in order to leverage the biological relevance of the co-expression relationships. We call this integrated network, Patient Specific Functional Interaction Network (pFIN). pFINs serve as a context-aware framework for refining neoantigen candidates toward those most likely to exert meaningful therapeutic impact (Figure 4). pFINs isn't simply a gene co-expression network filtered by PPI we also enhanced it with the most confident connection related to membrane localized proteins. This membrane focused expansion is motivated by recent evidence that neoantigens derived from membrane localized proteins are enriched among immunogenic peptides and correlate with improved response to immune checkpoint blockade [14,15]~~[13,14]~~. To clarify the scale of the pruned patient-specific functional interaction networks used for prioritisation, we also quantified the number of retained genes, edges, largest connected component size, and isolated genes across all 30 melanoma samples; these network-size summaries are provided in Additional file3, Supplementary Table S2.

Formatted: Font: (Default) Calibri, English (United States)

Field Code Changed

Formatted: Font: (Default) +Body (Calibri)

Formatted: English (United States)

Formatted: English (United States)

Figure 4. Network-based integration framework for epitope prioritization. RNA-seq data from patient specific cancer biopsies were processed within the EGG pipeline to generate raw gene expression counts. Pearson correlation across the normalized matrix was used to construct an aggregate gene co-expression network, from which patient-specific networks were derived. Each patient-specific co-expression network was subsequently filtered and refined for biological relevance. Networks were first constrained to interactions present in an extended protein-protein interaction (PPI) reference (HumanNet-XN). Edges were then filtered to retain the strongest connections per node. To enhance immunological relevance, edges involving membrane associated genes (annotated by Gene Ontology terms) were added. The final output consisted of weighted, patient-specific functional interaction networks enriched for biologically supported and immunologically prioritized interactions. The steps illustrated in panels A and B are implemented by the sample_networks_generation; further usage details are provided are available as Additional file 1 (Supplementary Material).

EGG exploits the interactional properties of the pFINs predicting the functional relevance of each epitope's gene of origin using topological metrics computed on the sample specific network degree, strength, betweenness centrality, and measures of network perturbation such as Weighted Connectivity Impact (WCI) and Largest Component Impact (LCI). In this manner it captures local connectivity, control of information flow, redundancy and resilience. These gene level features are

integrated with orthogonal evidence: CRISPR dependency scores from DepMap [24] as an experimental proxy for essentiality, and epitope level sequence metrics from pVACtools (e.g., predicted HLA class I/II binding), which assess the intrinsic immunogenic potential of the peptide independently of its gene of origin. Since these inputs derive from different experiments and sources, they are, per se, non-integrable. EGG converts each data source to ranks and aggregates them via a Borda procedure to produce a consensus ranked epitope list per sample (Figure 5). To aid in the biological interpretation, prioritized genes are annotated with Gene Ontology terms, MSigDB Hallmarks of Cancer processes [26], and IntOGen driver labels [27]. We incorporated these network topological features including degree, strength, betweenness centrality, and perturbation-based scores such as LCI since it has been shown that the placement of a gene within interaction networks is informative about its essentiality. The removal of highly connected and central proteins disproportionately fragments protein-protein interaction networks ("centrality-lethality" rule) [78–80][73–75]. Percolation style measures that quantify the impact of node removal on the size and weight of the largest connected component, similar in spirit to our LCI, further highlight nodes whose loss compromises network integrity and the function of core pathways [12]. By prioritizing epitopes derived from genes with high LCI and high centrality, EGG explicitly favors targets whose deletion would incur a substantial systems-level fitness cost, and are therefore less likely to be suppressed through immune editing. This design directly addresses a limitation of current neoantigen vaccines: tumors frequently evade immune pressure by losing, downregulating, or epigenetically silencing immunogenic but dispensable neoantigens. Longitudinal studies in lung cancer under immune checkpoint blockade have documented selective loss of neoantigens and reshaping of the neoantigen landscape during therapy [16][15]. More broadly, neoantigen depletion, via copy-number loss or transcriptional repression is considered a major immune escape route that could reduce the efficacy of personalized vaccines [16][15]. Recent work further argues that vaccines targeting passenger neoepitopes will drive immune editing of those mutations, whereas antigens derived from oncogenic drivers or other functionally indispensable genes are more durable because their loss carries a higher fitness penalty [81][76].

In addition, Al Bakir et al. showed that even a clonal EGFR driver neoantigen can be lost under combined TKI and neoantigen vaccine pressure, illustrating that oncogenic drivers themselves may be affected by immune escape [11]. This indicates that simply labelling genes as drivers or non-drivers is insufficient and supports incorporating different measures of functional indispensability to select the pool of candidate cancer vaccine epitopes. To complement topology-derived measures of indispensability with experimental evidence, EGG includes pan-cancer CRISPR dependency data from DepMap. Integrative analyses have shown that, given that network based features and genome scale

Formatted: Font: (Default) Calibri, English (United States)

Formatted: Font: (Default) Calibri, English (United States)

Formatted: Font: (Default) Calibri, English (United States)

Field Code Changed

Formatted: Font: (Default) Calibri, English (United States)

Field Code Changed

Field Code Changed

Field Code Changed

Formatted: Font: (Default) Calibri, English (United States)

Formatted: Font: (Default) +Body (Calibri)

Field Code Changed

Formatted: Font: (Default) Calibri, English (United States)

Formatted: Font: (Default) +Body (Calibri)

Field Code Changed

Formatted: Font: (Default) Calibri, English (United States)

Formatted: Font: (Default) +Body (Calibri)

Field Code Changed

Formatted: English (United States)

Formatted: English (United States)

CRISPR knockout screens capture non identical sets of essential genes, their combination can widen the search space for neoantigen-deriving genes By prioritising for network-central cancer and patient specific essential genes, EGG is designed to bias neoantigen selection toward targets that should be harder to lose under evolutionary pressure caused by the immune system and the cancer treatment. To complement these topology derived measures of indispensability with experimental evidence, we include pan-cancer CRISPR dependency data from DepMap, using Chronos gene effect scores as a quantitative measure of gene essentiality. Chronos models cell population dynamics to infer knockout fitness effects; more negative gene effect values indicate stronger depletion, with scores below -0.5 typically marking genes required for viability in most cell lines and scores near -1 characteristic of pan-essential genes [62]. These CRISPR knockout dependencies provide an alternative measure of gene essentiality that is independent of patient specific co-expression structure but is instead derived from thousands of in vitro cancer cell lines. Integrative analyses have shown that network based features and genome scale CRISPR knockout screens capture overlapping but non identical sets of essential genes, and combining them can improve the selection of essential genes [67,78,82][73,77]. In this framework, the experimental dependency layer provides a broad cancer-cell fitness prior, while patient specificity is retained through the pFIN-derived topology computed from each sample. This multi-criteria scheme allows a rational prioritisation of epitopes from genes that are both central in patient-specific pFINs and strongly essential across cancer cell lines, thereby increasing the chance that selected vaccine targets will remain expressed as the tumor evolves. In EGG, network centrality metrics (degree, strength, betweenness, WCI, LCI) and DepMap derived essentiality are therefore aggregated in the Borda consensus. This multi-criteria scheme allows epitopes from genes that are both structurally central in patient-specific pFINs and strongly essential across cancer cell lines, while still capturing context specific vulnerabilities seen only in one data source, thereby increasing the chance that selected vaccine targets will remain expressed as the tumor evolves.

We further assessed the robustness and contribution of the pFIN network layer through several sensitivity analyses. Specifically, we evaluated pFIN stability using leave-one-background-sample-out jackknife analyses, top-edge consistency testing, and two GDC TCGA-SKCM benchmarks using larger n = 103 and n = 469 background cohorts. We also performed a membrane-edge expansion ablation, which showed that the membrane-focused augmentation used in this case study had a modest overall effect: it promoted selected membrane-associated candidates but did not globally dominate the final prioritisation ranks. Users who wish to apply stronger membrane-focused prioritisation can increase the membrane-edge expansion parameter in their own analyses. These analyses are provided in Additional file 3, Supplementary Tables S3–S7.

Formatted: Font: (Default) Calibri, English (United States)

Field Code Changed

Formatted: Font: (Default) +Body (Calibri)

Formatted: English (United States)

Formatted: English (United States)

Formatted: English (United States)

Our strategy explicitly mitigates immune escape via deletion or silencing of dispensable neoantigen source genes, . However, this focus also defines its main limitation: EGG does not model escape mechanisms that operate through impaired antigen presentation, nor does it capture several immune-side determinants of neoantigen immunogenicity beyond peptide-HLA binding, such as antigen processing efficiency, T-cell receptor repertoire constraints, and immunodominance. EGG should therefore be interpreted as a prioritization framework centered on the tumor-side evolutionary durability of neoantigen source genes, rather than as a complete predictor of anti-tumor immune recognition. Consistent with this limitation, ghowever it's main limitation is that it does not address escape mechanisms that circumvent antigen presentation altogether. Genetic and epigenetic alterations in HLA class I/II, β2-microglobulin, antigen processing machinery, and IFNγ signaling are frequent in clinical tumors and represent dominant routes of immune evasion under T cell pressure [83–85][78–80]. In such settings, neoantigen source gene essentiality becomes largely irrelevant, as tumor cells can evade recognition despite maintaining expression of the targeted gene.

Formatted: English (United States)

Formatted: English (United States)

Formatted: Font: (Default) Calibri, English (United States)

Field Code Changed

Figure 5. Epitope prioritisation scheme. Patient Specific Functional Interaction Networks were used as the basis for epitope prioritization. Topological network metrics obtained from degree, edges strength, betweenness (including LCI, WCI) were computed to establish a measure of gene essentiality specific to individual patients. These network-derived features were combined with external biological evidence, including CRISPR-based gene essentiality scores (DepMap), epitope specific sequence metrics such as predicted HLA binding affinity. The integrated information was aggregated into a ranked epitope list, ensuring robust consensus prioritization. To provide additional biological context, candidate genes were annotated with cancer hallmarks, Gene Ontology terms, and IntOGen driver gene information. The resulting framework enables informed selection of epitopes by linking immunogenic potential to system-level relevance and cancer biology. The steps illustrated in this figure are implemented by the epitopes_prioritisation module with details of usage are provided in Additional file 1 (Supplementary Material).

Determinants of epitope prioritization

By applying our multi-step prioritisation approach described above across our TCGA melanoma samples, we identified correlation patterns among features utilised in the epitope prioritisation. Such pairwise relationships are summarised in Figure 6A. As expected, network centrality-based metrics are strongly intercorrelated and align closely with the Borda score, indicating that candidates from highly connected or influential genes tend to occupy top positions in the rank. DepMap survivability and predicted binding affinity showed a mild negative correlation with the Borda scores (negative values imply greater essentiality), and only limited correlation with network centralities. While DepMap highlights experimentally observed dependencies and provides biologically informed metrics, network centrality measures capture data-driven patterns of connectivity based on the unique molecular

landscape of each patient. Exploiting both levels of information yields a more robust prioritization compared to single layer prioritisation methods. We hypothesize that the prioritization of epitopes according to essentiality and network centrality will constrain immune escape, as the elimination of such genes is expected to impair tumor fitness. EGG's ranking aligns most strongly with network centrality measures, while also retaining contributions from sequence-dependent features like HLA binding affinity and gene essentiality from CRISPR knockout studies. HLA expression was not included as a ranking criterion in the analysis presented here, explaining its limited correlation with the Borda score. This reflects EGG's emphasis on complementing sequence-based epitope prioritisation with gene-level functional information, including database-derived essentiality and patient-specific network topology. Nevertheless, HLA expression is retained in the final output tables and can be considered during downstream interpretation or included by the user as an additional ranking criterion in the borda score, together with other optional pVACtools algorithm outputs.HLAs expression were not included as ranking criterion, hence their lack of correlation to the Borda score. This variable, together with other optional pvactools algorithms outputs can be easily included by the user as a ranking criterion.

Formatted: English (United States)

Formatted: English (United States)

We also evaluated the contribution of membrane-associated genes to the final prioritized epitope lists, given that membrane-associated edge expansion was included as an optional network refinement step. Across the 30 patients, membrane-associated genes accounted for a median of 22.3% of all candidate epitopes. Among top-ranked candidates, the median number of membrane-associated epitopes was 2 in the top 10, 4 in the top 20, and 10 in the top 50. Thus, membrane-derived epitopes were recovered among prioritized candidates, but they did not dominate the top-ranked sets. This is consistent with the intentionally light membrane-edge expansion used in the default analysis; stronger prioritization effects would be expected only if users deliberately increase the membrane-edge expansion parameter.

Formatted: English (United States)

Driver-associated epitopes showed a modest but significant overrepresentation among the top prioritized candidates compared with the remaining candidate pool. As shown in Figure 6B, driver-derived epitopes accounted for 34/296 top-10 candidates across samples (11.5%), compared with 133/1992 non-top-10 candidates (6.7%; one-sided Fisher's exact test, odds ratio = 1.81, p-value = 0.0034). These driver-derived candidates included loss-of-function, activating, and mixed activating/loss-of-function driver annotations. Although most top-ranked epitopes were still derived from non-driver genes, the relative overrepresentation of driver-derived candidates is consistent with the inclusion of functional and network-context features in the prioritization framework. One possible explanation is that some cancer driver genes occupy more central or functionally important positions in the network, although this interpretation remains exploratory.The fraction of epitopes deriving from

known cancer drivers is enriched among the top prioritized epitopes compared to the overall pool. As shown in Figure 6, panel B, within the top 10 per sample, both loss-of-function and activating drivers contribute to this enrichment. The presence of driver genes toward higher scores may reflect the tendency of cancer driver genes to act as network hubs.

To further evaluate potential determinants of prioritisation, we tested whether peptide length or tumor DNA variant allele frequency (VAF) influenced final epitope rank. Neither feature showed a meaningful association with prioritisation outcome, supporting that EGG ranking was not primarily driven by peptide-length bias or VAF differences in this dataset (Additional file 3, Supplementary Figures 2 and 3).

In order to ascertain whether overrepresented epitopes across the population exist, we then assessed the occurrence of epitope candidates across patients. We observed that exact peptides are unique to individual patients (panel figure 6D), while the genes that give rise to epitopes show a small recurrence across cases (figure 6panel C). Altogether, these descriptive analyses illustrate the intended behavior of EGG in this case study: the pipeline defines the candidate space onto epitopes anchored in tumor-relevant biology, enriches for driver-gene targets, and produces shortlists that remain largely patient-specific at the peptide level while still revealing a small cohort of recurrent genes and epitope sequences that could inform shared targets or follow-up functional studies. Building on this, our data pinpoints a small set of recurrent targets. Among fusion derived events, KANSL1 is the most frequently shared gene (12 samples; figure 6panel C), and DIYKQIRANKVSVWRQ the most recurrent exact epitope (5 patients; figure 6panel D). These KANSL1-associated events were primarily linked to the KANSL1–ARL17/KANSARL fusion locus, which has been described previously in cancer transcriptomic studies [86]. A comparable pattern emerges for genomic mutations: exact mutation-derived peptides remain patient-specific, but the gene NRAS shows the highest recurrence (7 samples; figure 6panel C), with the epitope LDILDTAGREEYSAMRDQYMRTGEGFLCV shared across 3 patients (figure 6panel D). Since NRAS is a known recurrent melanoma driver, we compared this observation with the reported TCGA-SKCM NRAS mutation frequency of 28%. The observed frequency of NRAS-derived epitope candidates in the selected cohort was not higher than expected relative to this background rate (one-sided binomial test, p-value = 0.776), indicating that NRAS recurrence is consistent with the known melanoma mutational landscape rather than evidence of unexpected overrepresentation [69]. In both fusion and genomic contexts, the presence of recurrent epitopes highlights the potential for "off-the-shelf" vaccination strategies, an approach already explored for shared/public neoantigens such as KRAS hotspots and frameshift peptides in microsatellite instability (MSI) tumors. Early trials of shared neoantigen vaccines (including KRAS focused mRNA platforms and multi epitope regimens)

Formatted: English (United States)

Formatted: English (United States)

Formatted: English (United States)

Formatted: English (United States)

Formatted: Font: (Default) Calibri, English (United States)

Formatted: English (United States)

Formatted: English (United States)

Formatted: Font: (Default) Calibri, English (United States)

Formatted: English (United States)

Formatted: Font color: Auto

demonstrate feasibility and immunogenicity, underscoring the translational value of the recurrent sequences identified by epitope generation pipelines [87][81].

Fundamentally, the main outputs of EGG, are interactive prioritization tables for each sample that integrate epitope and gene level features into a unified view. These tables report the HLA allele predicted to present each epitope together with corresponding transcript abundance, peptide sequence, mutation source, and class I/II designation. For each candidate, network-derived features (degree, strength, betweenness, LCI, WCI) and external evidence (DepMap survivability, IntOGen driver status, cancer hallmark annotation, GO terms) are displayed alongside the consensus Borda score and rank. This design provides investigators with a transparent overview of how multiple layers of information contribute to prioritization, linking peptide level immunogenicity with the biological relevance of the source gene. Tables for each patient that include prioritised epitopes and their topological and biological features obtained in our analysis can be found in Additional file 2 (Supplementary Material). To provide orthogonal bioinformatic support for the prioritisation strategy, we compared the final EGG Borda ranking with an IC50 MHC binding affinity only ranking using external experimentally supported epitope evidence from the Immune Epitope Database (IEDB) [88], the Cancer Epitope Database and Analysis Resource (CEDAR) [89], and TSNAdb v2.0 [90]. Because exact full-length peptide matches were rare, we used a relaxed min-8 containment match to identify candidates containing experimentally supported presented or immunogenic peptide cores; this analysis showed that Borda ranking improved recovery of externally supported candidates relative to IC50-only ranking at the most selective top-ranked cutoffs (Additional file 3, Supplementary Figure S6-S8 and Supplementary Tables S8–S9). Although EGG is designed to prioritise epitopes derived from genes with greater functional and network relevance, the proposed evolutionary durability of such targets remains a hypothesis in the present study. The external epitope-database analysis supports the potential presentation and immunogenicity of selected candidates, but does not directly test whether their source genes are less likely to be lost or silenced under immune pressure.

Figure 6. Exploratory analysis of epitope features. (A) Spearman correlation between features used in the Borda ranking procedure, highlighting expected dependencies among network-based measures and binding affinity scores. (B) Fraction of epitopes derived from driver versus non-driver genes within the top-10 prioritized epitopes per sample, showing contributions from both loss-of-function (inactivating alterations disabling tumor suppressors) and activating (gain-of-function mutations activating oncogenes) drivers. (C) Distribution of genes shared between samples, showing that most candidate genes are unique to individual samples. A value of 1 indicates that the gene was observed in one sample only. (D) Distribution of epitope sequences shared between samples, with the majority being patient specific. A value of 1 indicates that the epitope sequence was observed in one sample only. Most genes and epitope sequences were patient-specific.

Field Code Changed

Formatted: Font: (Default) Calibri, English (United States)

Formatted: Font: (Default) +Body (Calibri)

Formatted: English (United States)

Formatted: English (United States)

Formatted: English (United States)

Formatted: Font: (Default) Calibri, English (United States)

Formatted: English (United States)

Formatted: Font: (Default) Calibri, English (United States)

Formatted: English (United States)

Formatted: Font: (Default) Calibri, English (United States)

Formatted: English (United States)

Formatted: English (United States)

Formatted: English (United States)

Formatted: English (United States)

Formatted: Font: Not Italic, Font color: Auto

Formatted: English (United States)

Formatted: English (United States)

Comparison of currently available neoantigen prediction pipelines

EGG adds a co-expression-network based prioritization step that ranks neoantigens by the centrality and indispensability of their source genes, with the goal of reducing immune evasion risk. The pipeline is implemented in Snakemake and automates resource retrieval and environment setup for local execution. Compared with prominent publicly available pipelines surveyed in Table 1, EGG's network-aware prioritization constitutes a distinctive feature while keeping installation and execution simple. To contextualize EGG within the current landscape, we note the following pipelines: pVACtools stands out as a modular ecosystem (pVACseq/bind/fuse/splice) with extensive algorithm support and integrated visualization (pVACview) and vaccine design utilities (pVACvector); pVACtools also includes comprehensive workflows, such as immuno.wdl on the Terra platform, to automate end-to-end analyses, including upstream preprocessing. TSNAD v2.0 is an end-to-end pipeline for tumor-normal WES/WGS (with optional RNA-seq) that incorporates expression and gene-fusion filters and uses the deep-learning predictor DeepHLApan to score both HLA binding and immunogenicity [91,92][82,83]. ScanNeo2 provides an end-to-end workflow from raw DNA/RNA data and uniquely broadens variant coverage to include exitron splicing and gene fusions alongside SNVs/indels, enabling wider candidate recovery [9]. INTEGRATE-neo is purpose-built for fusion-derived neoantigens, offering an automated pipeline from fusion calling through HLA binding specifically for gene-fusion events [5]. nextNEOpi [93][84]is a Nextflow [94][85] based, fully automated pipeline that starts from raw WES/WGS in combination with RNA-seq and additionally quantifies patient and neoepitope level features linked to immunogenicity and response to checkpoint blockade. NeoHunter emphasizes flexible input handling and systematically detects and prioritizes neoantigens not only from SNVs/indels but also from gene fusions and aberrant splicing within a single framework [10]. NeoDisc is an end-to-end clinical proteogenomic pipeline that integrates immunopeptidomics with genomics and transcriptomics to prioritize both canonical and noncanonical tumor specific antigens [95][86]. SNAF focuses on splicing-derived antigens, predicting MHC-bound T-cell epitopes and altered-surface B-cell targets and using machine-learning prioritization to enrich for immunogenic events [8]. Seq2Neo provides a one-stop workflow from raw data and incorporates a convolutional neural network immunogenicity model to rank candidates derived from SNVs, indels, and gene fusions [96][87]. TransHLA is a recent deep-learning model for HLA-presented epitope detection rather than a full sequencing-to-neoantigen workflow; it uses Transformer and Residue CNN architectures with ESM2-derived sequence and structure embeddings to predict HLA class I and class II epitopes, and notably performs peptide-level screening without requiring HLA alleles as input [97].

Crucially, the prioritization module of EGG is flexible and fully customizable by the user. Optional features can be enabled or disabled and thresholds adjusted, for example using different algorithm for

Field Code Changed

Formatted: Font: (Default) Calibri, English (United States)

Formatted: Font: (Default) +Body (Calibri)

Field Code Changed

Field Code Changed

Field Code Changed

Formatted: Font: (Default) Calibri, English (United States)

Formatted: Font: (Default) +Body (Calibri)

Field Code Changed

Formatted: Font: (Default) Calibri, English (United States)

Formatted: Font: (Default) +Body (Calibri)

Field Code Changed

Field Code Changed

Formatted: Font: (Default) Calibri, English (United States)

Formatted: Font: (Default) +Body (Calibri)

Field Code Changed

Field Code Changed

Formatted: Font: (Default) Calibri, English (United States)

Formatted: Font: (Default) +Body (Calibri), English (United States)

Formatted: English (United States)

Formatted: Font: (Default) Calibri, English (United States)

Formatted: English (United States)

peptide MHC binding, immunogenicity scores, gene essentiality, and multiple network-based centrality metrics. The relative weights of these features used during prioritization are also modifiable by the users, allowing researchers to emphasize specific criteria for a customizable ranking process.

Tool	Mutations considered	Input	MHC class	HLA typing	Co-expression context
EGG (Epitope Generation Getaway)	SNVs, indels, gene fusions, alt splicing	Fastqs	I, II	yes	yes
ScanNeo2	SNVs, indels, gene fusions, alt splicing	Fastqs, BAM, VCF	I, II	yes	no
INTEGRATE-neo	Gene fusions	GenePred, BEDPE	I	no	no
nextNEOpi	SNVs, indels, gene fusions	Fastqs, BAM	I, II	yes	no
pVACtools	SNVs, indels, gene fusions, alt splicing	Fastqs, VCF	I, II	yes	no
TSANAD v2.0	SNVs, indels	Fastqs, VCF	I	yes	no
NeoHunter	SNVs, indels, gene fusions, alt splicing	Fastqs, BAM, VCF	I	yes	no
NeoDisc	SNVs, indels, gene fusions, alt splicing	Fastqs, BAM, VCF, MS	I, II	yes	no
SNAF	Alt splicing	Fastqs	I, II	yes	no
Seq2Neo	SNVs, indels, gene fusions	Fastqs	I, II	yes	no

Table 1. Feature matrix for neoantigen prediction pipelines and epitope-prediction tools. This table compares the Epitope Generation Gateway (EGG) with selected public neoantigen prediction workflows and standalone epitope-prediction tools. For each tool, we summarize supported somatic alteration classes, input requirements, MHC class coverage, HLA typing or HLA input requirements,

Formatted: English (United States)
Formatted: Centered, Space After: 0 pt
Formatted Table
Formatted: English (United States)
Formatted: Space After: 0 pt, Line spacing: Multiple 1.15 li
Formatted: English (United States)
Formatted: Space After: 0 pt, Line spacing: Multiple 1.15 li
Formatted: English (United States)
Formatted: Space After: 0 pt, Line spacing: Multiple 1.15 li
Formatted: English (United States)
Formatted: Space After: 0 pt, Line spacing: Multiple 1.15 li
Formatted: English (United States)
Formatted: Space After: 0 pt, Line spacing: Multiple 1.15 li
Formatted: English (United States)
Formatted: Space After: 0 pt, Line spacing: Multiple 1.15 li
Formatted: English (United States)
Formatted: Space After: 0 pt, Line spacing: Multiple 1.15 li
Formatted: English (United States)
Formatted: Space After: 0 pt, Line spacing: Multiple 1.15 li
Formatted: English (United States)
Formatted: Space After: 0 pt, Line spacing: Multiple 1.15 li
Formatted: English (United States)
Formatted: Space After: 0 pt, Line spacing: Multiple 1.15 li
Formatted: English (United States)
Formatted: Font: Not Bold, English (United States)

immunogenicity or presentation prediction, and prioritization features. Because some entries, such as TransHLA, are prediction models rather than full end-to-end sequencing pipelines, not all workflow-level features are applicable. Checkmarks indicate that the feature is included or supported by the tool or tool suite; dashes indicate that the feature is not part of the standard reported functionality or is not applicable. EGG is distinguished by combining neoantigen discovery with patient-specific source-gene co-expression context, PPI-informed network metrics, DepMap-based gene essentiality, and configurable weighted consensus ranking.~~**Table 1. Feature matrix for neo-antigen prediction pipelines.** This table benchmarks the Epitope Generation Gateway (EGG) against leading public workflows, summarizing for each tool the classes of somatic alterations analyzed (SNVs, indels, gene fusions, splicing), accepted input formats, MHC coverage (class I and/or II), built-in HLA typing, and use of patient-specific gene co-expression data to provide biological context.~~

Conclusion

Epitope Generation Gateway (EGG) can bridge the gap between abundant raw neoantigen predictions and the practical need for biologically meaningful ordered results. By embedding candidate epitopes within patient-specific functional interaction networks and incorporating orthogonal evidence of essentiality and driver status, EGG prioritizes epitopes more likely to be therapeutically relevant by prioritising more essential genes less likely to be subject to immune evasion while also offering the user with alternative prioritisation methods and important gene related biological information. In the case study presented in this work, we highlight that the majority of epitopes remain unique between patients at the sequence level, whereas gene level recurrence is more common. These results show that EGG can serve as a flexible framework for integrating diverse data sources, balancing immunological plausibility with tumor systems biology, and informing the rational design of personalized cancer vaccines.

Abbreviations

EGG: Epitope Generation Gateway, GO: Gene Ontology, HLA: Human Leukocyte Antigen, LCC: Largest Connected Component, LCI: Largest Component Impact, MHC: Major Histocompatibility Complex, MSI: Microsatellite Instability, ~~-~~pFIN: Patient Specific Functional Interaction Network, PPI: Protein Protein Interaction, SKCM: Skin Cutaneous Melanoma, SNV: Single Nucleotide Variation, TCGA: The Cancer Genome Atlas, VAF: Variant Allele Frequency, vst: Variance Stabilizing Transformation, WCI: Weighted Connectivity Impact.

Data availability

The EGG source code is available from the GitHub repository: https://github.com/fhaive/epitope_generation_gateway.

The workflow version corresponding to the revised manuscript is archived in WorkflowHub version 2 and is available at https://workflowhub.eu/workflows/2165?version=2, with DOI:

Formatted: English (United States)

Formatted: English (United States)

Formatted: English (United States)

Formatted: English (United States)

Field Code Changed

Formatted: English (United States)

Formatted: English (United States)

Formatted: English (United States)

Field Code Changed

https://doi.org/10.48546/workflowhub.workflow.2165.2 GitHub Repository:

The software code is available from GitHub repository:

EGG is also registered in bio.tools (biotoolsID: epitope_generation_gateway; https://bio.tools/epitope_generation_gateway) and SciCrunch (RRID: SCR_028792).

https://github.com/fhaive/epitope_generation_gateway

Raw data:

Matched tumor RNA-seq, tumor DNA (WES), and normal DNA samples were retrieved from The Cancer Genome Atlas (TCGA) for 30 patients diagnosed with cutaneous melanoma (TCGA-SKCM study code). The 30 corresponding UUIDs are listed in Additional fileSupplementary File 2.

Output data:

Output data is provided in Additional file Supplementary File 2.

Supplementary Methods:

Pipeline Implementation is described in Additional file Supplementary File 1.

Supplementary Analyses:

Supplementary analyses are described in Additional file 3.

Competing interests

The authors declare that they have no competing interests that could have influenced the work reported in this paper.

Authors contributions

LM: Data Analysis, Investigation, Methodology, Software, Visualisation, Conceptualisation, Writing – original draft, Writing – review and editing, MF: Software, Methodology, Investigation, JC: Methodology, Writing – review and editing, FD: Methodology, Writing – review and editing, ED: Software, Writing – review and editing, MN: Writing – review and editing, AS: Methodology, Investigation, Writing – review and editing, SY: Conceptualisation, Funding Acquisition, Writing –

Formatted: English (United States)
Field Code Changed
Formatted: English (United States)
Formatted: English (United States)
Field Code Changed
Formatted: English (United States)
Formatted: English (United States)
Field Code Changed
Formatted: English (United States)
Formatted: English (United States)
Formatted: English (United States)
Formatted: Justified

review and editing, VC: Conceptualisation, Funding Acquisition, Writing – review and editing, AF: Conceptualisation, Investigation, Methodology, Supervision, Writing – original draft, Writing – review and editing, DG: Conceptualisation, Funding Acquisition, Investigation, Methodology, Supervision, Writing – review and editing

Additional files

Additional file 1 (.docx): Supplementary Methods / Supplementary Protocol.
Description: Step-by-step instructions for execution of individual pipeline modules.

Additional file 2 (.xlsx): Supplementary Tables.
Description: Patient-level tables of prioritised epitopes and associated topological and biological features.

Additional file 3 (.docx): Supplementary Analyses:

Formatted: English (United States)

Description: Additional sensitivity, robustness, benchmarking, and validation analyses supporting the prioritisation framework, including network stability, ranking-weight sensitivity, membrane-expansion ablation, peptide-length and VAF assessments, external evidence matching, and genomic-event burden correlations.

Formatted: Font: Not Bold, English (United States)

Formatted: English (United States)

Formatted: Justified

Use of AI assisted tools

During manuscript preparation, we used AI assisted tools to improve clarity and readability of text and as a coding aid. AI-assisted suggestions were reviewed and edited by the authors, who take full responsibility for the final content.

Funding

This work was supported by the Jane and Aatos Erkko Foundation.

References

1. Sahin U, Derhovanessian E, Miller M, Kloke B-P, Simon P, Löwer M, Bukur V, Tadmor AD, Luxemburger U, Schrörs B, Omokoko T, Vormehr M, Albrecht C, Paruzynski A, Kuhn AN, Buck J, Heesch S, Schreeb KH, Müller F, Ortseifer I, Vogler I, Godehardt E, Attig S, Rae R, Breitkreuz A, Tolliver C, Suchan M, Martic G, Hohberger A, Sorn P, Diekmann J, Ciesla J, Waksmann O, Brück A-K, Witt M, Zillgen M, Rothermel A, Kasemann B, Langer D, Bolte S, Diken M, Kreiter S, Nemecek R, Gebhardt C, Grabbe S, Höller C, Utikal J, Huber C, Loquai C, Türeci Ö. Personalized RNA mutanome vaccines mobilize poly-specific therapeutic immunity against cancer. *Nature*. 2017; doi: 10.1038/nature23003.

2. Ott PA, Hu Z, Keskin DB, Shukla SA, Sun J, Bozym DJ, Zhang W, Luoma A, Giobbie-Hurder A, Peter L, Chen C, Olive O, Carter TA, Li S, Lieb DJ, Eisenhaure T, Gjini E, Stevens J, Lane WJ, Javeri I, Nellaiappan K, Salazar AM, Daley H, Seaman M, Buchbinder EI, Yoon CH, Harden M, Lennon N, Gabriel S, Rodig SJ, Barouch DH, Aster JC, Getz G, Wucherpfennig K, Neuberg D, Ritz

Formatted: Bibliography, Widow/Orphan control, Adjust space between Latin and Asian text, Adjust space between Asian text and numbers

Field Code Changed

Formatted: English (United States)

J, Lander ES, Fritsch EF, Hacohen N, Wu CJ. An immunogenic personal neoantigen vaccine for patients with melanoma. *Nature*. 2017; doi: 10.1038/nature22991.

3. Li X, You J, Hong L, Liu W, Guo P, Hao X. Neoantigen cancer vaccines: a new star on the horizon. *Cancer Biol Med*. 2023; doi: 10.20892/j.issn.2095-3941.2023.0395.

4. Bang H, Park JS, Kim JY, Sung C, An J, Cho D, Lee S, Shim SB, Choi JK, Kim K. Gene essentiality for tumour growth influences neoantigen-directed immunoediting. *Clinical & Translational Med*. 2022; doi: 10.1002/ctm2.714.

5. Zhang J, Mardis ER, Maher CA. INTEGRATE-neo: a pipeline for personalized gene fusion neoantigen discovery. Hancock J, editor. *Bioinformatics*. 2017; doi: 10.1093/bioinformatics/btw674.

6. Nielsen M, Lundegaard C, Blicher T, Lamberth K, Harndahl M, Justesen S, Røder G, Peters B, Sette A, Lund O, Buus S. NetMHCpan, a Method for Quantitative Predictions of Peptide Binding to Any HLA-A and -B Locus Protein of Known Sequence. Kallas E, editor. *PLoS ONE*. 2007; doi: 10.1371/journal.pone.0000796.

7. Zhou Z, Lyu X, Wu J, Yang X, Wu S, Zhou J, Gu X, Su Z, Chen S. TSNAD: an integrated software for cancer somatic mutation and tumour-specific neoantigen detection. *R Soc Open Sci*. 2017; doi: 10.1098/rsos.170050.

8. Li G, Mahajan S, Ma S, Jeffery ED, Zhang X, Bhattacharjee A, Venkatasubramanian M, Weirauch MT, Miraldi ER, Grimes HL, Sheynkman GM, Tilburgs T, Salomonis N. Splicing neoantigen discovery with SNAF reveals shared targets for cancer immunotherapy. *Sci Transl Med*. 2024; doi: 10.1126/scitranslmed.ade2886.

9. Schäfer RA, Guo Q, Yang R. ScanNeo2: a comprehensive workflow for neoantigen detection and immunogenicity prediction from diverse genomic and transcriptomic alterations. Kendziorski C, editor. *Bioinformatics*. 2023; doi: 10.1093/bioinformatics/btad659.

10. Ma T, Zhao Z, Li H, Wei L, Zhang X. NeoHunter: Flexible software for systematically detecting neoantigens from sequencing data. *Quant Biol*. 2024; doi: 10.1002/qub2.28.

11. Al Bakir M, Reading JL, Gamble S, Rosenthal R, Uddin I, Rowan A, Przewrocka J, Rogers A, Wong YNS, Bentzen AK, Veeriah S, Ward S, Garnett AT, Kalavakur P, Martínez-Ruiz C, Puttick C, Huebner A, Cook DE, Moore DA, Abbosh C, Hiley CT, Naceur-Lombardelli C, Watkins TBK, Petkovic M, Schwarz RF, Gálvez-Cancino F, Litchfield K, Meldgaard P, Sorensen BS, Madsen LB, Jäger D, Forster MD, Arkenau T, Domingo-Vila C, Tree TIM, Kadivar M, Hadrup SR, Chain B, Quezada SA, McGranahan N, Swanton C. Clonal driver neoantigen loss under EGFR TKI and immune selection pressures. *Nature*. 2025; doi: 10.1038/s41586-025-08586-y.

12. Li X, Li W, Zeng M, Zheng R, Li M. Network-based methods for predicting essential genes or proteins: a survey. *Briefings in Bioinformatics*. 2020; doi: 10.1093/bib/bbz017.

13. Liu Y, Zhu F, Li X, Guan X, Hou Y, Feng Y, Dong X, Li Y. SpatialSNV: A novel method for identifying and analyzing spatially resolved SNVs in tumor microenvironments. *GigaScience*. 2025; doi: 10.1093/gigascience/giaf065.

14. Goldberger Z, Hauert S, Chang K, Kurtanich T, Alpar AT, Repond G, Wang Y, Gomes S, Krishnakumar R, Siddarth P, Swartz MA, Hubbell JA, Briquez PS. Membrane-localized neoantigens predict the efficacy of cancer immunotherapy. *Cell Reports Medicine*. 2023; doi: 10.1016/j.xcrm.2023.101145.

15. Castro A, Kaabinejadian S, Yari H, Hildebrand W, Zanetti M, Carter H. Subcellular location of source proteins improves prediction of neoantigens for immunotherapy. *EMBO J*. 2022; doi: 10.15252/embj.2022111071.

16. Xie N, Shen G, Gao W, Huang Z, Huang C, Fu L. Neoantigens: promising targets for cancer therapy. *Sig Transduct Target Ther*. 2023; doi: 10.1038/s41392-022-01270-x.

17. Anagnostou V, Smith KN, Forde PM, Niknafs N, Bhattacharya R, White J, Zhang T, Adleff V, Phallen J, Wali N, Hruban C, Guthrie VB, Rodgers K, Naidoo J, Kang H, Sharfman W, Georgiades C, Verde F, Illei P, Li QK, Gabrielson E, Brock MV, Zahnow CA, Baylin SB, Scharpf RB, Brahmer JR, Karchin R, Pardoll DM, Velculescu VE. Evolution of Neoantigen Landscape during Immune Checkpoint Blockade in Non–Small Cell Lung Cancer. *Cancer Discovery*. 2017; doi: 10.1158/2159-8290.CD-16-0828.

18. Benjamin D, Sato T, Cibulskis K, Getz G, Stewart C, Lichtenstein L. Calling Somatic SNVs and Indels with Mutect2. Bioinformatics;

19. Haas BJ, Dobin A, Stransky N, Li B, Yang X, Tickle T, Bankapur A, Ganote C, Doak TG, Pochet N, Sun J, Wu CJ, Gingeras TR, Regev A. STAR-Fusion: Fast and Accurate Fusion Transcript Detection from RNA-Seq. Bioinformatics;

20. Orenbuch R, Filip I, Comito D, Shaman J, Pe'er I, Rabadan R. arcasHLA: high-resolution HLA typing from RNAseq. Ponty Y, editor. *Bioinformatics*. 2020; doi: 10.1093/bioinformatics/btz474.

21. Hoang M, Richters M, Kiwala S, Griffith O, Griffith M. 32. pVACsplice: A Computational tool for predicting and prioritizing alternative splicing neoantigens. *Cancer Genetics*. 2024; doi: 10.1016/j.cancergen.2024.08.034.

22. Hundal J, Kiwala S, McMichael J, Miller CA, Xia H, Wollam AT, Liu CJ, Zhao S, Feng Y-Y, Graubert AP, Wollam AZ, Neichin J, Neveau M, Walker J, Gillanders WE, Mardis ER, Griffith OL, Griffith M. pVACtools: A Computational Toolkit to Identify and Visualize Cancer Neoantigens. *Cancer Immunology Research*. 2020; doi: 10.1158/2326-6066.CIR-19-0401.

23. Hundal J, Carreno BM, Petti AA, Linette GP, Griffith OL, Mardis ER, Griffith M. pVAC-Seq: A genome-guided in silico approach to identifying tumor neoantigens. *Genome Med*. 2016; doi: 10.1186/s13073-016-0264-5.

24. DepMap B. DepMap 24Q4 Public. Figshare+;

25. The Gene Ontology Consortium, Carbon S, Douglass E, Good BM, Unni DR, Harris NL, Mungall CJ, Basu S, Chisholm RL, Dodson RJ, Hartline E, Fey P, Thomas PD, Albou L-P, Ebert D, Kesling MJ, Mi H, Muruganujan A, Huang X, Mushayahama T, LaBonte SA, Siegele DA, Antonazzo G, Attrill H, Brown NH, Garapati P, Marygold SJ, Trovisco V, Dos Santos G, Falls K, Tabone C, Zhou P, Goodman JL, Strelets VB, Thurmond J, Garmiri P, Ishtiaq R, Rodríguez-López M, Acencio ML, Kuiper M, Lægreid A, Logie C, Lovering RC, Kramarz B, Saverimuttu SCC, Pinheiro SM, Gunn H, Su R, Thurlow KE, Chibucos M, Giglio M, Nadendla S, Munro J, Jackson R, Duesbury MJ, Del-Toro N, Meldal BHM, Paneerselvam K, Perfetto L, Porras P, Orchard S, Shrivastava A, Chang H-Y, Finn RD, Mitchell AL, Rawlings ND, Richardson L, Sangrador-Vegas A, Blake JA, Christie KR, Dolan ME, Drabkin HJ, Hill DP, Ni L, Sitnikov DM, Harris MA, Oliver SG, Rutherford K, Wood V, Hayles J, Bähler J, Bolton ER, De Pons JL, Dwinell MR, Hayman GT, Kaldunski ML, Kwitek AE, Laulederkind SJF, Plasterer C, Tutaj MA, Vedi M, Wang S-J, D'Eustachio P, Matthews L, Balhoff JP, Aleksander SA, Alexander MJ, Cherry JM, Engel SR, Gondwe F, Karra K, Miyasato SR, Nash RS, Simison M, Skrzypek MS, Weng S, Wong ED, Feuermann M, Gaudet P, Morgat A, Bakker E, Berardini TZ, Reiser L, Subramaniam S, Huala E, Arighi CN, Auchincloss A, Axelsen K, Argoud-Puy G, Bateman A, Blatter M-C, Boutet E, Bowler E, Breuza L, Bridge A, Britto R, Bye-A-Jee H,

Casas CC, Coudert E, Denny P, Estreicher A, Famiglietti ML, Georghiou G, Gos A, Gruaz-Gumowski N, Hatton-Ellis E, Hulo C, Ignatchenko A, Jungo F, Laiho K, Le Mercier P, Lieberherr D, Lock A, Lussi Y, MacDougall A, Magrane M, Martin MJ, Masson P, Natale DA, Hyka-Nouspikel N, Orchard S, Pedruzzi I, Pourcel L, Poux S, Pundir S, Rivoire C, Speretta E, Sundaram S, Tyagi N, Warner K, Zaru R, Wu CH, Diehl AD, Chan JN, Grove C, Lee RYN, Muller H-M, Raciti D, Van Auken K, Sternberg PW, Berriman M, Paulini M, Howe K, Gao S, Wright A, Stein L, Howe DG, Toro S, Westerfield M, Jaiswal P, Cooper L, Elser J. The Gene Ontology resource: enriching a GOld mine. *Nucleic Acids Research*. 2021; doi: 10.1093/nar/gkaa1113.

26. Liberzon A, Birger C, Thorvaldsdóttir H, Ghandi M, Mesirov JP, Tamayo P. The Molecular Signatures Database Hallmark Gene Set Collection. *Cell Systems*. 2015; doi: 10.1016/j.cels.2015.12.004.

27. Martínez-Jiménez F, Muiños F, Sentís I, Deu-Pons J, Reyes-Salazar I, Arnedo-Pac C, Mularoni L, Pich O, Bonet J, Kranas H, Gonzalez-Perez A, Lopez-Bigas N. A compendium of mutational cancer driver genes. *Nat Rev Cancer*. 2020; doi: 10.1038/s41568-020-0290-x.

28. The Cancer Genome Atlas Research Network, Weinstein JN, Collisson EA, Mills GB, Shaw KRM, Ozenberger BA, Ellrott K, Shmulevich I, Sander C, Stuart JM. The Cancer Genome Atlas Pan-Cancer analysis project. *Nat Genet*. 2013; doi: 10.1038/ng.2764.

29. Zhang Z, Hernandez K, Savage J, Li S, Miller D, Agrawal S, Ortuno F, Staudt LM, Heath A, Grossman RL. Uniform genomic data analysis in the NCI Genomic Data Commons. *Nat Commun*. 2021; doi: 10.1038/s41467-021-21254-9.

30. Danecek P, Bonfield JK, Liddle J, Marshall J, Ohan V, Pollard MO, Whitwham A, Keane T, McCarthy SA, Davies RM, Li H. Twelve years of SAMtools and BCFtools. *GigaScience*. 2021; doi: 10.1093/gigascience/giab008.

31. Li H, Handsaker B, Wysoker A, Fennell T, Ruan J, Homer N, Marth G, Abecasis G, Durbin R, 1000 Genome Project Data Processing Subgroup. The Sequence Alignment/Map format and SAMtools. *Bioinformatics*. 2009; doi: 10.1093/bioinformatics/btp352.

32. : Babraham Bioinformatics - FastQC A Quality Control tool for High Throughput Sequence Data. https://www.bioinformatics.babraham.ac.uk/projects/fastqc/ Accessed 2025 Dec 17.

33. Chen S, Zhou Y, Chen Y, Gu J. fastp: an ultra-fast all-in-one FASTQ preprocessor. *Bioinformatics*. 2018; doi: 10.1093/bioinformatics/bty560.

34. Mudge JM, Carbonell-Sala S, Diekhans M, Martinez JG, Hunt T, Jungreis I, Loveland JE, Arnan C, Barnes I, Bennett R, Berry A, Bignell A, Cerdán-Vélez D, Cochran K, Cortés LT, Davidson C, Donaldson S, Dursun C, Fatima R, Hardy M, Hebbar P, Hollis Z, James BT, Jiang Y, Johnson R, Kaur G, Kay M, Mangan RJ, Maquedano M, Gómez LM, Mathlouthi N, Merritt R, Ni P, Palumbo E, Perteghella T, Pozo F, Raj S, Sisu C, Steed E, Sumathipala D, Suner M-M, Uszczynska-Ratajczak B, Wass E, Yang YT, Zhang D, Finn RD, Gerstein M, Guigó R, Hubbard TJP, Kellis M, Kundaje A, Paten B, Tress ML, Birney E, Martin FJ, Frankish A. GENCODE 2025: reference gene annotation for human and mouse. *Nucleic Acids Research*. 2025; doi: 10.1093/nar/gkae1078.

35. Schneider VA, Graves-Lindsay T, Howe K, Bouk N, Chen H-C, Kitts PA, Murphy TD, Pruitt KD, Thibaud-Nissen F, Albracht D, Fulton RS, Kremitzki M, Magrini V, Markovic C, McGrath S, Steinberg KM, Auger K, Chow W, Collins J, Harden G, Hubbard T, Pelan S, Simpson JT, Threadgold G, Torrance J, Wood JM, Clarke L, Koren S, Boitano M, Peluso P, Li H, Chin C-S, Phillippy AM, Durbin R, Wilson RK, Flicek P, Eichler EE, Church DM. Evaluation of GRCh38 and de novo haploid genome assemblies demonstrates the enduring quality of the reference assembly. *Genome Res*. 2017; doi: 10.1101/gr.213611.116.

36. Ticke T, Bankapur A, Ganote C, Fulton B, Tirosh I, Chen J, Doak T, Henschel R, Pochet N, Wu C, Haas B, Regev A. Trinity CTAT: a community resource for *de novo* and reference-based RNA-Seq analysis.

37. Liao Y, Smyth GK, Shi W. The R package Rsubread is easier, faster, cheaper and better for alignment and quantification of RNA sequencing reads. *Nucleic Acids Research*. 2019; doi: 10.1093/nar/gkz114.

38. Liao Y, Smyth GK, Shi W. featureCounts: an efficient general purpose program for assigning sequence reads to genomic features. *Bioinformatics*. 2014; doi: 10.1093/bioinformatics/btt656.

39. Köster J, Rahmann S. Snakemake—a scalable bioinformatics workflow engine. *Bioinformatics*. 2012; doi: 10.1093/bioinformatics/bts480.

40. Murphy C, Elemento O. AGFusion: annotate and visualize gene fusions. Bioinformatics;

41. Cunningham F, Allen JE, Allen J, Alvarez-Jarreta J, Amode MR, Armean IM, Austine-Orimoloye O, Azov AG, Barnes I, Bennett R, Berry A, Bhai J, Bignell A, Billis K, Boddu S, Brooks L, Charkhchi M, Cummins C, Da Rin Fioretto L, Davidson C, Dodiya K, Donaldson S, El Houdaigui B, El Naboulsi T, Fatima R, Giron CG, Genez T, Martinez JG, Guijarro-Clarke C, Gymer A, Hardy M, Hollis Z, Hourlier T, Hunt T, Juettemann T, Kaikala V, Kay M, Lavidas I, Le T, Lemos D, Marugán JC, Mohanan S, Mushtaq A, Naven M, Ogeh DN, Parker A, Parton A, Perry M, Piližota I, Prosovetskaia I, Sakthivel MP, Salam AIA, Schmitt BM, Schuilenburg H, Sheppard D, Pérez-Silva JG, Stark W, Steed E, Sutinen K, Sukumaran R, Sumathipala D, Suner M-M, Szpak M, Thormann A, Tricomi FF, Urbina-Gómez D, Veidenberg A, Walsh TA, Walts B, Willhoft N, Winterbottom A, Wass E, Chakiachvili M, Flint B, Frankish A, Giorgetti S, Haggerty L, Hunt SE, IIsley GR, Loveland JE, Martin FJ, Moore B, Mudge JM, Muffato M, Perry E, Ruffier M, Tate J, Thybert D, Trevanion SJ, Dyer S, Harrison PW, Howe KL, Yates AD, Zerbino DR, Flicek P. Ensembl 2022. *Nucleic Acids Research*. 2022; doi: 10.1093/nar/gkab1049.

42. Mistry J, Chuguransky S, Williams L, Qureshi M, Salazar GA, Sonnhammer ELL, Tosatto SCE, Paladin L, Raj S, Richardson LJ, Finn RD, Bateman A. Pfam: The protein families database in 2021. *Nucleic Acids Research*. 2021; doi: 10.1093/nar/gkaa913.

43. Shao XM, Bhattacharya R, Huang J, Sivakumar IKA, Tokheim C, Zheng L, Hirsch D, Kaminow B, Omdahl A, Bonsack M, Riemer AB, Velculescu VE, Anagnostou V, Pagel KA, Karchin R. High-Throughput Prediction of MHC Class I and II Neoantigens with MHCnuggets. *Cancer Immunology Research*. 2020; doi: 10.1158/2326-6066.CIR-19-0464.

44. Kent WJ, Sugnet CW, Furey TS, Roskin KM, Pringle TH, Zahler AM, Haussler AD. The Human Genome Browser at UCSC. *Genome Res*. 2002; doi: 10.1101/gr.229102.

45. McKenna A, Hanna M, Banks E, Sivachenko A, Cibulskis K, Kernytsky A, Garimella K, Altshuler D, Gabriel S, Daly M, DePristo MA. The Genome Analysis Toolkit: A MapReduce framework for analyzing next-generation DNA sequencing data. *Genome Res*. 2010; doi: 10.1101/gr.107524.110.

46. Van Der Auwera GA, Carneiro MO, Hartl C, Poplin R, Del Angel G, Levy-Moonshine A, Jordan T, Shakir K, Roazen D, Thibault J, Banks E, Garimella KV, Altshuler D, Gabriel S, DePristo MA. From FastQ Data to High-Confidence Variant Calls: The Genome Analysis Toolkit Best Practices Pipeline. *CP in Bioinformatics*. 2013; doi: 10.1002/0471250953.bi1110s43.

47. Li H, Durbin R. Fast and accurate short read alignment with Burrows–Wheeler transform. *Bioinformatics*. 2009; doi: 10.1093/bioinformatics/btp324.

48. Sherry ST. dbSNP: the NCBI database of genetic variation. *Nucleic Acids Research*. 2001; doi: 10.1093/nar/29.1.308.

49. Karczewski KJ, Francioli LC, Tiao G, Cummings BB, Alföldi J, Wang Q, Collins RL, Laricchia KM, Ganna A, Birnbaum DP, Gauthier LD, Brand H, Solomonson M, Watts NA, Rhodes D, Singer-Berk M, England EM, Seaby EG, Kosmicki JA, Walters RK, Tashman K, Farjoun Y, Banks E, Poterba T, Wang A, Seed C, Whiffin N, Chong JX, Samocha KE, Pierce-Hoffman E, Zappala Z, O'Donnell-Luria AH, Minikel EV, Weisburd B, Lek M, Ware JS, Vittal C, Armean IM, Bergelson L, Cibulskis K, Connolly KM, Covarrubias M, Donnelly S, Ferriera S, Gabriel S, Gentry J, Gupta N, Jeandet T, Kaplan D, Llanwarne C, Munshi R, Novod S, Petrillo N, Roazen D, Ruano-Rubio V, Saltzman A, Schleicher M, Soto J, Tibbetts K, Tolonen C, Wade G, Talkowski ME, Genome Aggregation Database Consortium, Aguilar Salinas CA, Ahmad T, Albert CM, Ardissino D, Atzmon G, Barnard J, Beaugerie L, Benjamin EJ, Boehnke M, Bonnycastle LL, Bottinger EP, Bowden DW, Bown MJ, Chambers JC, Chan JC, Chasman D, Cho J, Chung MK, Cohen B, Correa A, Dabelea D, Daly MJ, Darbar D, Duggirala R, Dupuis J, Ellinor PT, Elosua R, Erdmann J, Esko T, Färkkilä M, Florez J, Franke A, Getz G, Glaser B, Glatt SJ, Goldstein D, Gonzalez C, Groop L, Haiman C, Hanis C, Harms M, Hiltunen M, Holi MM, Hultman CM, Kallela M, Kaprio J, Kathiresan S, Kim B-J, Kim YJ, Kirov G, Kooner J, Koskinen S, Krumholz HM, Kugathasan S, Kwak SH, Laakso M, Lehtimäki T, Loos RJF, Lubitz SA, Ma RCW, MacArthur DG, Marrugat J, Mattila KM, McCarroll S, McCarthy MI, McGovern D, McPherson R, Meigs JB, Melander O, Metspalu A, Neale BM, Nilsson PM, O'Donovan MC, Ongur D, Orozco L, Owen MJ, Palmer CNA, Palotie A, Park KS, Pato C, Pulver AE, Rahman N, Remes AM, Rioux JD, Ripatti S, Roden DM, Saleheen D, Salomaa V, Samani NJ, Scharf J, Schunkert H, Shoemaker MB, Sklar P, Soininen H, Sokol H, Spector T, Sullivan PF, Suvisaari J, Tai ES, Teo YY, Tiinamaija T, Tsuang M, Turner D, Tusie-Luna T, Vartiainen E, Vawter MP, Ware JS, Watkins H, Weersma RK, Wessman M, Wilson JG, Xavier RJ, Neale BM, Daly MJ, MacArthur DG. The mutational constraint spectrum quantified from variation in 141,456 humans. *Nature*. 2020; doi: 10.1038/s41586-020-2308-7.

50. Li H. Tabix: fast retrieval of sequence features from generic TAB-delimited files. *Bioinformatics*. 2011; doi: 10.1093/bioinformatics/btq671.

51. Li H. Aligning sequence reads, clone sequences and assembly contigs with BWA-MEM. arXiv;

52. : Picard Tools - By Broad Institute. https://broadinstitute.github.io/picard/ Accessed 2025 Dec 17.

53. McLaren W, Gil L, Hunt SE, Riat HS, Ritchie GRS, Thormann A, Flicek P, Cunningham F. The Ensembl Variant Effect Predictor. *Genome Biol*. 2016; doi: 10.1186/s13059-016-0974-4.

54. Bray NL, Pimentel H, Melsted P, Pachter L. Near-optimal probabilistic RNA-seq quantification. *Nat Biotechnol*. 2016; doi: 10.1038/nbt.3519.

55. Guille A, Adélaïde J, Finetti P, Andre F, Birnbaum D, Mamessier E, Bertucci F, Chaffanet M. A benchmarking study of individual somatic variant callers and voting-based ensembles for whole-exome sequencing. *Briefings in Bioinformatics*. 2024; doi: 10.1093/bib/bbae697.

56. Haas BJ, Dobin A, Li B, Stransky N, Pochet N, Regev A. Accuracy assessment of fusion transcript detection via read-mapping and de novo fusion transcript assembly-based methods. *Genome Biol*. 2019; doi: 10.1186/s13059-019-1842-9.

57. Cotto KC, Feng Y-Y, Ramu A, Richters M, Freshour SL, Skidmore ZL, Xia H, McMichael JF, Kunisaki J, Campbell KM, Chen TH-P, Rozycki EB, Adkins D, Devarakonda S, Sankararaman S, Lin Y, Chapman WC, Maher CA, Arora V, Dunn GP, Uppaluri R, Govindan R, Griffith OL, Griffith M. Integrated analysis of genomic and transcriptomic data for the discovery of splice-associated variants in cancer. *Nat Commun*. 2023; doi: 10.1038/s41467-023-37266-6.

58. Saha E, Ben Guebila M, Fanfani V, Shutta KH, DeMeo DL, Quackenbush J, Lopes-Ramos CM. Aging-associated alterations in gene regulatory networks associate with risk, prognosis and response to therapy in lung adenocarcinoma. *NPJ Aging*. 2025; doi: 10.1038/s41514-025-00247-8.

59. Fanfani V, Shutta KH, Mandros P, Fischer J, Saha E, Micheletti S, Chen C, Ben Guebila M, Lopes-Ramos CM, Quackenbush J. Reproducible processing of TCGA regulatory networks. *GigaScience*. 2025; doi: 10.1093/gigascience/giaf126.

60. Love MI, Huber W, Anders S. Moderated estimation of fold change and dispersion for RNA-seq data with DESeq2. *Genome Biol*. 2014; doi: 10.1186/s13059-014-0550-8.

61. Kuijjer ML, Hsieh P-H, Quackenbush J, Glass K. lionessR: single sample network inference in R. *BMC Cancer*. 2019; doi: 10.1186/s12885-019-6235-7.

62. Hwang S, Kim CY, Yang S, Kim E, Hart T, Marcotte EM, Lee I. HumanNet v2: human gene networks for disease research. *Nucleic Acids Research*. 2019; doi: 10.1093/nar/gky1126.

63. Carlson M. org.Hs.eg.db. Bioconductor;

64. Conin B, Billault-Chaumartin I, El Sayyed H, Quenech'Du N, Cockram C, Koszul R, Espéli O. Extended sister-chromosome catenation leads to massive reorganization of the *E. coli* genome. *Nucleic Acids Research*. 2022; doi: 10.1093/nar/gkac105.

65. Durinck S, Spellman PT, Birney E, Huber W. Mapping identifiers for the integration of genomic datasets with the R/Bioconductor package biomaRt. *Nat Protoc*. 2009; doi: 10.1038/nprot.2009.97.

66. Csárdi G, Nepusz T, Müller K, Horvát S, Traag V, Zanini F, Noom D. igraph for R: R interface of the igraph library for graph theory and network analysis. Zenodo;

67. Dempster JM, Boyle I, Vazquez F, Root DE, Boehm JS, Hahn WC, Tsherniak A, McFarland JM. Chronos: a cell population dynamics model of CRISPR experiments that improves inference of gene fitness effects. *Genome Biol*. 2021; doi: 10.1186/s13059-021-02540-7.

68. Hayward NK, Wilmott JS, Waddell N, Johansson PA, Field MA, Nones K, Patch A-M, Kakavand H, Alexandrov LB, Burke H, Jakrot V, Kazakoff S, Holmes O, Leonard C, Sabarinathan R, Mularoni L, Wood S, Xu Q, Waddell N, Tembe V, Pupo GM, De Paoli-Iseppi R, Vilain RE, Shang P, Lau LMS, Dagg RA, Schramm S-J, Pritchard A, Dutton-Regester K, Newell F, Fitzgerald A, Shang CA, Grimmond SM, Pickett HA, Yang JY, Stretch JR, Behren A, Kefford RF, Hersey P, Long GV, Cebon J, Shackleton M, Spillane AJ, Saw RPM, López-Bigas N, Pearson JV, Thompson JF, Scolyer RA, Mann GJ. Whole-genome landscapes of major melanoma subtypes. *Nature*. 2017; doi: 10.1038/nature22071.

69. Akbani R, Akdemir KC, Aksoy BA, Albert M, Ally A, Amin SB, Arachchi H, Arora A, Auman JT, Ayala B, Baboud J, Balasundaram M, Balu S, Barnabas N, Bartlett J, Bartlett P, Bastian BC, Baylin SB, Behera M, Belyaev D, Benz C, Bernard B, Beroukhim R, Bir N, Black AD, Bodenheimer T, Boice L, Boland GM, Bono R, Bootwalla MS, Bosenberg M, Bowen J, Bowlby R, Bristow CA, Brockway-Lunardi L, Brooks D, Brzezinski J, Bshara W, Buda E, Burns WR, Butterfield YSN, Button M, Calderone T, Cappellini GA, Carter C, Carter SL, Cherney L, Cherniack AD, Chevalier A, Chin L, Cho J, Cho RJ, Choi Y-L, Chu A, Chudamani S, Cibulskis K, Ciriello G, Clarke A, Coons S, Cope L, Crain D, Curley E, Danilova L, D'Atri S, Davidsen T, Davies MA, Delman KA, Demchok JA, Deng QA, Deribe YL, Dhalla N, Dhir R, DiCara D, Dinikin M, Dubina M, Ebrom JS, Egea S, Eley G, Engel J, Eschbacher JM, Fedosenko KV, Felau I, Fennell T, Ferguson ML, Fisher S, Flaherty KT, Frazer S, Frick J, Fulidou V, Gabriel SB, Gao J, Gardner J, Garraway LA, Gastier-Foster JM, Gaudioso C, Gehlenborg N, Genovese G, Gerken M, Gershenwald JE, Getz G, Gomez-Fernandez C, Gribbin T, Grimsby J, Gross B, Guin R, Gutschner T, Hadjipanayis A, Halaban R, Hanf B, Haussler

D, Haydu LE, Hayes DN, Hayward NK, Heiman DI, Herbert L, Herman JG, Hersey P, Hoadley KA, Hodis E, Holt RA, Hoon DSB, Hoppough S, Hoyle AP, Huang FW, Huang M, Huang S, Hutter CM, Ibbs M, Iype L, Jacobsen A, Jakrot V, Janning A, Jeck WR, Jefferys SR, Jensen MA, Jones CD, Jones SJM, Ju Z, Kakavand H, Kang H, Kefford RF, Khuri FR, Kim J, Kirkwood JM, Klode J, Korkut A, Korski K, Krauthammer M, Kucherlapati R, Kwong LN, Kycler W, Ladanyi M, Lai PH, Laird PW, Lander E, Lawrence MS, Lazar AJ, Łaźniak R, Lee D, Lee JE, Lee J, Lee K, Lee S, Lee W, Leporowska E, Leraas KM, Li HI, Lichtenberg TM, Lichtenstein L, Lin P, Ling S, Liu J, Liu O, Liu W, Long GV, Lu Y, Ma S, Ma Y, Mackiewicz A, Mahadeshwar HS, Malke J, Mallery D, Manikhas GM, Mann GJ, Marra MA, Matejka B, Mayo M, Mehrabi S, Meng S, Meyerson M, Mieczkowski PA, Miller JP, Miller ML, Mills GB, Moiseenko F, Moore RA, Morris S, Morrison C, Morton D, Moschos S, Mose LE, Muller FL, Mungall AJ, Murawa D, Murawa P, Murray BA, Nezi L, Ng S, Nicholson D, Noble MS, Osunkoya A, Owonikoko TK, Ozenberger BA, Pagani E, Paklina OV, Pantazi A, Parfenov M, Parfitt J, Park PJ, Park W-Y, Parker JS, Passarelli F, Penny R, Perou CM, Pihl TD, Potapova O, Prieto VG, Protopopov A, Quinn MJ, Radenbaugh A, Rai K, Ramalingam SS, Raman AT, Ramirez NC, Ramirez R, Rao U, Rathmell WK, Ren X, Reynolds SM, Roach J, Robertson AG, Ross MI, Roszik J, Russo G, Saksena G, Saller C, Samuels Y, Sander C, Sander C, Sandusky G, Santoso N, Saul M, Saw RPM, Schadendorf D, Schein JE, Schultz N, Schumacher SE, Schwallier C, Scolyer RA, Seidman J, Sekhar PC, Sekhon HS, Senbabaoglu Y, Seth S, Shannon KF, Sharpe S, Sharpless NE, Shaw KRM, Shelton C, Shelton T, Shen R, Sheth M, Shi Y, Shiau CJ, Shmulevich I, Sica GL, Simons JV, Sinha R, Sipahimalani P, Sofia HJ, Soloway MG, Song X, Sougnez C, Spillane AJ, Spychała A, Stretch JR, Stuart J, Suchorska WM, Sucker A, Sumer SO, Sun Y, Synott M, Tabak B, Tabler TR, Tam A, Tan D, Tang J, Tarnuzzer R, Tarvin K, Tatka H, Taylor BS, Teresiak M, Thiessen N, Thompson JF, Thorne L, Thorsson V, Trent JM, Triche TJ, Tsai KY, Tsou P, Van Den Berg DJ, Van Allen EM, Veluvolu U, Verhaak RG, Voet D, Voronina O, Walter V, Walton JS, Wan Y, Wang Y, Wang Z, Waring S, Watson IR, Weinhold N, Weinstein JN, Weisenberger DJ, White P, Wilkerson MD, Wilmott JS, Wise L, Wiznerowicz M, Woodman SE, Wu C-J, Wu C-C, Wu J, Wu Y, Xi R, Xu AW, Yang D, Yang L, Yang L, Zack TI, Zenklusen JC, Zhang H, Zhang J, Zhang W, Zhao X, Zhu J, Zhu K, Zimmer L, Zmuda E, Zou L. Genomic Classification of Cutaneous Melanoma. *Cell*. 2015; doi: 10.1016/j.cell.2015.05.044.

70. Yarchoan M, Gane EJ, Marron TU, Perales-Linares R, Yan J, Cooch N, Shu DH, Fertig EJ, Kagohara LT, Bartha G, Northcott J, Lyle J, Rochestie S, Peters J, Connor JT, Jaffee EM, Csiki I, Weiner DB, Perales-Puchalt A, Sardesai NY. Personalized neoantigen vaccine and pembrolizumab in advanced hepatocellular carcinoma: a phase 1/2 trial. *Nat Med*. 2024; doi: 10.1038/s41591-024-02894-y.

71. Biswas N, Chakrabarti S, Padul V, Jones LD, Ashili S. Designing neoantigen cancer vaccines, trials, and outcomes. *Front Immunol*. 2023; doi: 10.3389/fimmu.2023.1105420.

72. Wu D-W, Jia S-P, Xing S-J, Ma H, Wang X, Tang Q-Y, Li Z-W, Wu Q, Bai M, Zhang X-Y, Fu X-F, Jia M-M, Tang Y, Chen L, Li N. Personalized neoantigen cancer vaccines: current progression, challenges and a bright future. *Clin Exp Med*. 2024; doi: 10.1007/s10238-024-01436-7.

73. Cai Y, Li D, Lv D, Yu J, Ma Y, Jiang T, Ding N, Liu Z, Li Y, Xu J. MHC-I-presented non-canonical antigens expand the cancer immunotherapy targets in acute myeloid leukemia. *Sci Data*. 2024; doi: 10.1038/s41597-024-03660-y.

74. Ye L, Creaney J, Redwood A, Robinson B. The Current Lung Cancer Neoantigen Landscape and Implications for Therapy. *Journal of Thoracic Oncology*. 2021; doi: 10.1016/j.jtho.2021.01.1624.

75. Li J, Xiao Z, Wang D, Jia L, Nie S, Zeng X, Hu W. The screening, identification, design and clinical application of tumor-specific neoantigens for TCR-T cells. *Mol Cancer*. 2023; doi: 10.1186/s12943-023-01844-5.

76. Buonaguro L, Tagliamonte M. Selecting Target Antigens for Cancer Vaccine Development. *Vaccines*. 2020; doi: 10.3390/vaccines8040615.

77. Cavallo F, Calogero RA, Forni G. Are oncoantigens suitable targets for anti-tumour therapy? *Nat Rev Cancer*. 2007; doi: 10.1038/nrc2208.

78. Jiang P, Wang H, Li W, Zang C, Li B, Wong YJ, Meyer C, Liu JS, Aster JC, Liu XS. Network analysis of gene essentiality in functional genomics experiments. *Genome Biol*. 2015; doi: 10.1186/s13059-015-0808-9.

79. Jeong H, Mason SP, Barabási A-L, Oltvai ZN. Lethality and centrality in protein networks. *Nature*. 2001; doi: 10.1038/35075138.

80. Ning K, Ng HK, Srihari S, Leong HW, Nesvizhskii AI. Examination of the relationship between essential genes in PPI network and hub proteins in reverse nearest neighbor topology. *BMC Bioinformatics*. 2010; doi: 10.1186/1471-2105-11-505.

81. Crosby EJ, Hartman ZC, Lyerly HK. Beyond Neoantigens: Antigens Derived from Tumor Drivers as Cancer Vaccine Targets. *Clin Cancer Res*. 2023; doi: 10.1158/1078-0432.CCR-23-1244.

82. Azhagesan K, Ravindran B, Raman K. Network-based features enable prediction of essential genes across diverse organisms. Mande SC, editor. *PLoS ONE*. 2018; doi: 10.1371/journal.pone.0208722.

83. Hazini A, Fisher K, Seymour L. Deregulation of HLA-I in cancer and its central importance for immunotherapy. *J Immunother Cancer*. 2021; doi: 10.1136/jitc-2021-002899.

84. Taylor BC, Balko JM. Mechanisms of MHC-I Downregulation and Role in Immunotherapy Response. *Front Immunol*. 2022; doi: 10.3389/fimmu.2022.844866.

85. Zaretsky JM, Garcia-Diaz A, Shin DS, Escuin-Ordinas H, Hugo W, Hu-Lieskovan S, Torrejon DY, Abril-Rodriguez G, Sandoval S, Barthly L, Saco J, Homet Moreno B, Mezzadra R, Chmielowski B, Ruchalski K, Shintaku IP, Sanchez PJ, Puig-Saus C, Cherry G, Seja E, Kong X, Pang J, Berent-Maoz B, Comin-Anduix B, Graeber TG, Tumeh PC, Schumacher TNM, Lo RS, Ribas A. Mutations Associated with Acquired Resistance to PD-1 Blockade in Melanoma. *N Engl J Med*. 2016; doi: 10.1056/NEJMoa1604958.

86. Kalender Atak Z, Gianfelici V, Hulselmans G, De Keersmaecker K, Devasia AG, Geerdens E, Mentens N, Chiaretti S, Durinck K, Uyttebroeck A, Vandenberghe P, Wlodarska I, Cloos J, Foà R, Speleman F, Cools J, Aerts S. Comprehensive Analysis of Transcriptome Variation Uncovers Known and Novel Driver Events in T-Cell Acute Lymphoblastic Leukemia. Scott HS, editor. *PLoS Genet*. 2013; doi: 10.1371/journal.pgen.1003997.

87. Merck Sharp & Dohme LLC. A Phase 1, Open-Label, Multicenter Study to Assess the Safety and Tolerability of mRNA-5671/V941 as a Monotherapy and in Combination With Pembrolizumab in Participants With KRAS Mutant Advanced or Metastatic Non-Small Cell Lung Cancer, Colorectal Cancer or Pancreatic Adenocarcinoma. clinicaltrials.gov; 2025 Jan. Report No.: NCT03948763.

88. Vita R, Mahajan S, Overton JA, Dhanda SK, Martini S, Cantrell JR, Wheeler DK, Sette A, Peters B. The Immune Epitope Database (IEDB): 2018 update. *Nucleic Acids Research*. 2019; doi: 10.1093/nar/gky1006.

89. Sayers EW, Cavanaugh M, Clark K, Pruitt KD, Sherry ST, Yankie L, Karsch-Mizrachi I. GenBank 2023 update. *Nucleic Acids Research*. 2023; doi: 10.1093/nar/gkac1012.

90. Wu J, Chen W, Zhou Y, Chi Y, Hua X, Wu J, Gu X, Chen S, Zhou Z. TSNAdb v2.0: The Updated Version of Tumor-Specific Neoantigen Database. *Genomics, Proteomics & Bioinformatics*. 2023; doi: 10.1016/j.gpb.2022.09.012.

91. Zhou Z, Wu J, Ren J, Chen W, Zhao W, Gu X, Chi Y, He Q, Yang B, Wu J, Chen S. TSNAD v2.0: A one-stop software solution for tumor-specific neoantigen detection. *Computational and Structural Biotechnology Journal*. 2021; doi: 10.1016/j.csbj.2021.08.016.

92. Wu J, Wang W, Zhang J, Zhou B, Zhao W, Su Z, Gu X, Wu J, Zhou Z, Chen S. DeepHLApan: A Deep Learning Approach for Neoantigen Prediction Considering Both HLA-Peptide Binding and Immunogenicity. *Front Immunol*. 2019; doi: 10.3389/fimmu.2019.02559.

93. Rieder D, Fotakis G, Ausserhofer M, René G, Paster W, Trajanoski Z, Finotello F. nextNEOpi: a comprehensive pipeline for computational neoantigen prediction. Valencia A, editor. *Bioinformatics*. 2022; doi: 10.1093/bioinformatics/btab759.

94. Di Tommaso P, Chatzou M, Floden EW, Barja PP, Palumbo E, Notredame C. Nextflow enables reproducible computational workflows. *Nat Biotechnol*. 2017; doi: 10.1038/nbt.3820.

95. Huber F, Arnaud M, Stevenson BJ, Michaux J, Benedetti F, Thevenet J, Bobisse S, Chiffelle J, Gehert T, Müller M, Pak H, Krämer AI, Altimiras ER, Racle J, Taillandier-Coindard M, Muehlethaler K, Auger A, Saugy D, Murgues B, Benyagoub A, Gfeller D, Laniti DD, Kandalaft L, Rodrigo BN, Bouchaab H, Tissot S, Coukos G, Harari A, Bassani-Sternberg M. A comprehensive proteogenomic pipeline for neoantigen discovery to advance personalized cancer immunotherapy. *Nat Biotechnol*. 2025; doi: 10.1038/s41587-024-02420-y.

96. Diao K, Chen J, Wu T, Wang X, Wang G, Sun X, Zhao X, Wu C, Wang J, Yao H, Gerarduzzi C, Liu X-S. Seq2Neo: A Comprehensive Pipeline for Cancer Neoantigen Immunogenicity Prediction. *Int J Mol Sci*. 2022; doi: 10.3390/ijms231911624.

97. Lu T, Wang X, Nie W, Huo M, Li S. TransHLA: a Hybrid Transformer model for HLA-presented epitope detection. *GigaScience*. 2025; doi: 10.1093/gigascience/giaf008.

1. Sahin U, Derhovanessian E, Miller M, Kloke B-P, Simon P, Löwer M, et al.. Personalized RNA mutanome vaccines mobilize poly-specific therapeutic immunity against cancer. *Nature*. 2017; doi: 10.1038/nature23003.

2. Ott PA, Hu Z, Keskin DB, Shukla SA, Sun J, Bozym DJ, et al.. An immunogenic personal neoantigen vaccine for patients with melanoma. *Nature*. 2017; doi: 10.1038/nature22991.

3. Li X, You J, Hong L, Liu W, Guo P, Hao X. Neoantigen cancer vaccines: a new star on the horizon. *Cancer Biol Med*. 2023; doi: 10.20892/j.issn.2095-3941.2023.0395.

4. Bang H, Park JS, Kim JY, Sung C, An J, Cho D, et al.. Gene essentiality for tumour growth influences neoantigen-directed immunoediting. *Clinical & Translational Med*. 2022; doi: 10.1002/ctm2.714.

5. Zhang J, Mardis ER, Maher CA. INTEGRATE-neo: a pipeline for personalized gene fusion neoantigen discovery. Hancock J, editor. *Bioinformatics*. 2017; doi: 10.1093/bioinformatics/btw674.

6. Nielsen M, Lundegaard C, Blicher T, Lamberth K, Harndahl M, Justesen S, et al.. NetMHCpan, a Method for Quantitative Predictions of Peptide Binding to Any HLA-A and -B Locus Protein of Known Sequence. Kallas E, editor. *PLoS ONE*. 2007; doi: 10.1371/journal.pone.0000796.

7. Zhou Z, Lyu X, Wu J, Yang X, Wu S, Zhou J, et al.. TSNAD: an integrated software for cancer somatic mutation and tumour-specific neoantigen detection. *R Soc Open Sci*. 2017; doi: 10.1098/rsos.170050.

8. Li G, Mahajan S, Ma S, Jeffery ED, Zhang X, Bhattacharjee A, et al.. Splicing neoantigen discovery with SNAF reveals shared targets for cancer immunotherapy. *Sci Transl Med*. 2024; doi: 10.1126/scitranslmed.ade2886.

9. Schäfer RA, Guo Q, Yang R. ScanNeo2: a comprehensive workflow for neoantigen detection and immunogenicity prediction from diverse genomic and transcriptomic alterations. Kendziorski C, editor. *Bioinformatics*. 2023; doi: 10.1093/bioinformatics/btad659.

10. Ma T, Zhao Z, Li H, Wei L, Zhang X. NeoHunter: Flexible software for systematically detecting neoantigens from sequencing data. *Quant Biol*. 2024; doi: 10.1002/qub2.28.

11. Al Bakir M, Reading JL, Gamble S, Rosenthal R, Uddin I, Rowan A, et al.. Clonal driver neoantigen loss under EGFR TKI and immune selection pressures. *Nature*. 2025; doi: 10.1038/s41586-025-08586-y.

12. Li X, Li W, Zeng M, Zheng R, Li M. Network-based methods for predicting essential genes or proteins: a survey. *Briefings in Bioinformatics*. 2020; doi: 10.1093/bib/bbz017.

13. Goldberger Z, Hauert S, Chang K, Kurtanich T, Alpar AT, Repond G, et al.. Membrane-localized neoantigens predict the efficacy of cancer immunotherapy. *Cell Reports Medicine*. 2023; doi: 10.1016/j.xcrm.2023.101145.

14. Castro A, Kaabinejadian S, Yari H, Hildebrand W, Zanetti M, Carter H. Subcellular location of source proteins improves prediction of neoantigens for immunotherapy. *EMBO J*. 2022; doi: 10.15252/embj.2022111071.

15. Xie N, Shen G, Gao W, Huang Z, Huang C, Fu L. Neoantigens: promising targets for cancer therapy. *Sig Transduct Target Ther*. 2023; doi: 10.1038/s41392-022-01270-x.

16. Anagnostou V, Smith KN, Forde PM, Niknafs N, Bhattacharya R, White J, et al.. Evolution of Neoantigen Landscape during Immune Checkpoint Blockade in Non–Small Cell Lung Cancer. *Cancer Discovery*. 2017; doi: 10.1158/2159-8290.CD-16-0828.

17. Benjamin D, Sato T, Cibulskis K, Getz G, Stewart C, Lichtenstein L. Calling Somatic SNVs and Indels with Mutect2. Bioinformatics;

18. Haas BJ, Dobin A, Stransky N, Li B, Yang X, Tickle T, et al.. STAR-Fusion: Fast and Accurate Fusion Transcript Detection from RNA-Seq. Bioinformatics;

19. Orenbuch R, Filip I, Comito D, Shaman J, Pe'er I, Rabadan R. arcasHLA: high-resolution HLA typing from RNAseq. Ponty Y, editor. *Bioinformatics*. 2020; doi: 10.1093/bioinformatics/btz474.

20. Hoang M, Richters M, Kiwala S, Griffith O, Griffith M. 32. pVACsplice: A Computational tool for predicting and prioritizing alternative splicing neoantigens. *Cancer Genetics*. 2024; doi: 10.1016/j.cancergen.2024.08.034.

21. Hundal J, Kiwala S, McMichael J, Miller CA, Xia H, Wollam AT, et al.. pVACtools: A Computational Toolkit to Identify and Visualize Cancer Neoantigens. *Cancer Immunology Research*. 2020; doi: 10.1158/2326-6066.CIR-19-0401.

22. Hundal J, Carreno BM, Petti AA, Linette GP, Griffith OL, Mardis ER, et al.. pVAC-Seq: A genome-guided in silico approach to identifying tumor neoantigens. *Genome Med*. 2016; doi: 10.1186/s13073-016-0264-5.

23. DepMap B. DepMap 24Q4 Public. Figshare+;

24. The Gene Ontology Consortium, Carbon S, Douglass E, Good BM, Unni DR, Harris NL, et al.. The Gene Ontology resource: enriching a GOld mine. *Nucleic Acids Research*. 2021; doi: 10.1093/nar/gkaa1113.

25. Liberzon A, Birger C, Thorvaldsdóttir H, Ghandi M, Mesirov JP, Tamayo P. The Molecular Signatures Database Hallmark Gene Set Collection. *Cell Systems*. 2015; doi: 10.1016/j.cels.2015.12.004.

26. Martínez-Jiménez F, Muiños F, Sentís I, Deu-Pons J, Reyes-Salazar I, Arnedo-Pac C, et al.. A compendium of mutational cancer driver genes. *Nat Rev Cancer*. 2020; doi: 10.1038/s41568-020-0290-x.

27. The Cancer Genome Atlas Research Network, Weinstein JN, Collisson EA, Mills GB, Shaw KRM, Ozenberger BA, et al.. The Cancer Genome Atlas Pan-Cancer analysis project. *Nat Genet*. 2013; doi: 10.1038/ng.2764.

28. Zhang Z, Hernandez K, Savage J, Li S, Miller D, Agrawal S, et al.. Uniform genomic data analysis in the NCI Genomic Data Commons. *Nat Commun*. 2021; doi: 10.1038/s41467-021-21254-9.

29. Danecek P, Bonfield JK, Liddle J, Marshall J, Ohan V, Pollard MO, et al.. Twelve years of SAMtools and BCFtools. *GigaScience*. 2021; doi: 10.1093/gigascience/giab008.

30. Li H, Handsaker B, Wysoker A, Fennell T, Ruan J, Homer N, et al.. The Sequence Alignment/Map format and SAMtools. *Bioinformatics*. 2009; doi: 10.1093/bioinformatics/btp352.

31. : Babraham Bioinformatics - FastQC A Quality Control tool for High Throughput Sequence Data. https://www.bioinformatics.babraham.ac.uk/projects/fastqc/ Accessed 2025 Dec 17.

32. Chen S, Zhou Y, Chen Y, Gu J. fastp: an ultra-fast all-in-one FASTQ preprocessor. *Bioinformatics*. 2018; doi: 10.1093/bioinformatics/bty560.

33. Mudge JM, Carbonell-Sala S, Diekhans M, Martinez JG, Hunt T, Jungreis I, et al.. GENCODE 2025: reference gene annotation for human and mouse. *Nucleic Acids Research*. 2025; doi: 10.1093/nar/gkae1078.

34. Schneider VA, Graves-Lindsay T, Howe K, Bouk N, Chen H-C, Kitts PA, et al.. Evaluation of GRCh38 and de novo haploid genome assemblies demonstrates the enduring quality of the reference assembly. *Genome Res*. 2017; doi: 10.1101/gr.213611.116.

35. Ticke T, Bankapur A, Ganote C, Fulton B, Tirosh I, Chen J, et al.. Trinity CTAT: a community resource for *de novo* and reference-based RNA-Seq analysis.

36. Liao Y, Smyth GK, Shi W. The R package Rsubread is easier, faster, cheaper and better for alignment and quantification of RNA sequencing reads. *Nucleic Acids Research*. 2019; doi: 10.1093/nar/gkz114.

37. Liao Y, Smyth GK, Shi W. featureCounts: an efficient general purpose program for assigning sequence reads to genomic features. *Bioinformatics*. 2014; doi: 10.1093/bioinformatics/btt656.

38. Köster J, Rahmann S. Snakemake—a scalable bioinformatics workflow engine. *Bioinformatics*. 2012; doi: 10.1093/bioinformatics/bts480.

39. Murphy C, Elemento O. AGFusion: annotate and visualize gene fusions. Bioinformatics;

40. Cunningham F, Allen JE, Allen J, Alvarez-Jarreta J, Amode MR, Armean IM, et al.. Ensembl 2022. *Nucleic Acids Research*. 2022; doi: 10.1093/nar/gkab1049.

41. Mistry J, Chuguransky S, Williams L, Qureshi M, Salazar GA, Sonnhammer ELL, et al.. Pfam: The protein families database in 2021. *Nucleic Acids Research*. 2021; doi: 10.1093/nar/gkaa913.

42. Shao XM, Bhattacharya R, Huang J, Sivakumar IKA, Tokheim C, Zheng L, et al.. High-Throughput Prediction of MHC Class I and II Neoantigens with MHCnuggets. *Cancer Immunology Research*. 2020; doi: 10.1158/2326-6066.CIR-19-0464.

43. Kent WJ, Sugnet CW, Furey TS, Roskin KM, Pringle TH, Zahler AM, et al.. The Human Genome Browser at UCSC. *Genome Res*. 2002; doi: 10.1101/gr.229102.

44. McKenna A, Hanna M, Banks E, Sivachenko A, Cibulskis K, Kernytsky A, et al.. The Genome Analysis Toolkit: A MapReduce framework for analyzing next-generation DNA sequencing data. *Genome Res*. 2010; doi: 10.1101/gr.107524.110.

45. Van Der Auwera GA, Carneiro MO, Hartl C, Poplin R, Del Angel G, Levy-Moonshine A, et al.. From FastQ Data to High-Confidence Variant Calls: The Genome Analysis Toolkit Best Practices Pipeline. *CP in Bioinformatics*. 2013; doi: 10.1002/0471250953.bi1110s43.

46. Li H, Durbin R. Fast and accurate short read alignment with Burrows–Wheeler transform. *Bioinformatics*. 2009; doi: 10.1093/bioinformatics/btp324.

47. Sherry ST. dbSNP: the NCBI database of genetic variation. *Nucleic Acids Research*. 2001; doi: 10.1093/nar/29.1.308.

48. Karczewski KJ, Francioli LC, Tiao G, Cummings BB, Alföldi J, Wang Q, et al.. The mutational constraint spectrum quantified from variation in 141,456 humans. *Nature*. 2020; doi: 10.1038/s41586-020-2308-7.

49. Li H. Tabix: fast retrieval of sequence features from generic TAB-delimited files. *Bioinformatics*. 2011; doi: 10.1093/bioinformatics/btq671.

50. Li H. Aligning sequence reads, clone sequences and assembly contigs with BWA-MEM. arXiv;

51. : Picard Tools - By Broad Institute. https://broadinstitute.github.io/picard/ Accessed 2025 Dec 17.

52. McLaren W, Gil L, Hunt SE, Riat HS, Ritchie GRS, Thormann A, et al.. The Ensembl Variant Effect Predictor. *Genome Biol*. 2016; doi: 10.1186/s13059-016-0974-4.

53. Bray NL, Pimentel H, Melsted P, Pachter L. Near-optimal probabilistic RNA-seq quantification. *Nat Biotechnol*. 2016; doi: 10.1038/nbt.3519.

54. Cotto KC, Feng Y-Y, Ramu A, Richters M, Freshour SL, Skidmore ZL, et al.. Integrated analysis of genomic and transcriptomic data for the discovery of splice-associated variants in cancer. *Nat Commun*. 2023; doi: 10.1038/s41467-023-37266-6.

55. Love MI, Huber W, Anders S. Moderated estimation of fold change and dispersion for RNA-seq data with DESeq2. *Genome Biol*. 2014; doi: 10.1186/s13059-014-0550-8.

56. Kuijjer ML, Hsieh P-H, Quackenbush J, Glass K. lionessR: single sample network inference in R. *BMC Cancer*. 2019; doi: 10.1186/s12885-019-6235-7.

57. Hwang S, Kim CY, Yang S, Kim E, Hart T, Marcotte EM, et al.. HumanNet v2: human gene networks for disease research. *Nucleic Acids Research*. 2019; doi: 10.1093/nar/gky1126.

58. Carlson M. org.Hs.eg.db. Bioconductor;

59. Conin B, Billault-Chaumartin I, El Sayyed H, Quenech'Du N, Cockram C, Koszul R, et al.. Extended sister-chromosome catenation leads to massive reorganization of the *E. coli* genome. *Nucleic Acids Research*. 2022; doi: 10.1093/nar/gkac105.

60. Durinck S, Spellman PT, Birney E, Huber W. Mapping identifiers for the integration of genomic datasets with the R/Bioconductor package biomaRt. *Nat Protoc*. 2009; doi: 10.1038/nprot.2009.97.

61. Csárdi G, Nepusz T, Müller K, Horvát S, Traag V, Zanini F, et al.. igraph for R: R interface of the igraph library for graph theory and network analysis. Zenodo;

62. Dempster JM, Boyle I, Vazquez F, Root DE, Boehm JS, Hahn WC, et al.. Chronos: a cell population dynamics model of CRISPR experiments that improves inference of gene fitness effects. *Genome Biol*. 2021; doi: 10.1186/s13059-021-02540-7.

63. Hayward NK, Wilmott JS, Waddell N, Johansson PA, Field MA, Nones K, et al.. Whole-genome landscapes of major melanoma subtypes. *Nature*. 2017; doi: 10.1038/nature22071.

64. Akbani R, Akdemir KC, Aksoy BA, Albert M, Ally A, Amin SB, et al.. Genomic Classification of Cutaneous Melanoma. *Cell*. 2015; doi: 10.1016/j.cell.2015.05.044.

65. Yarchoan M, Gane EJ, Marron TU, Perales-Linares R, Yan J, Cooch N, et al.. Personalized neoantigen vaccine and pembrolizumab in advanced hepatocellular carcinoma: a phase 1/2 trial. *Nat Med*. 2024; doi: 10.1038/s41591-024-02894-y.

66. Biswas N, Chakrabarti S, Padul V, Jones LD, Ashili S. Designing neoantigen cancer vaccines, trials, and outcomes. *Front Immunol*. 2023; doi: 10.3389/fimmu.2023.1105420.

67. Wu D-W, Jia S-P, Xing S-J, Ma H, Wang X, Tang Q-Y, et al.. Personalized neoantigen cancer vaccines: current progression, challenges and a bright future. *Clin Exp Med*. 2024; doi: 10.1007/s10238-024-01436-7.

68. Cai Y, Li D, Lv D, Yu J, Ma Y, Jiang T, et al.. MHC-I-presented non-canonical antigens expand the cancer immunotherapy targets in acute myeloid leukemia. *Sci Data*. 2024; doi: 10.1038/s41597-024-03660-y.

69. Ye L, Creaney J, Redwood A, Robinson B. The Current Lung Cancer Neoantigen Landscape and Implications for Therapy. *Journal of Thoracic Oncology*. 2021; doi: 10.1016/j.jtho.2021.01.1624.

70. Li J, Xiao Z, Wang D, Jia L, Nie S, Zeng X, et al.. The screening, identification, design and clinical application of tumor-specific neoantigens for TCR-T cells. *Mol Cancer*. 2023; doi: 10.1186/s12943-023-01844-5.

71. Buonaguro L, Tagliamonte M. Selecting Target Antigens for Cancer Vaccine Development. *Vaccines*. 2020; doi: 10.3390/vaccines8040615.

72. Cavallo F, Calogero RA, Forni G. Are oncoantigens suitable targets for anti-tumour therapy? *Nat Rev Cancer*. 2007; doi: 10.1038/nrc2208.

73. Jiang P, Wang H, Li W, Zang C, Li B, Wong YJ, et al.. Network analysis of gene essentiality in functional genomics experiments. *Genome Biol*. 2015; doi: 10.1186/s13059-015-0808-9.

74. Jeong H, Mason SP, Barabási A-L, Oltvai ZN. Lethality and centrality in protein networks. *Nature*. 2001; doi: 10.1038/35075138.

75. Ning K, Ng HK, Srihari S, Leong HW, Nesvizhskii AI. Examination of the relationship between essential genes in PPI network and hub proteins in reverse nearest neighbor topology. *BMC Bioinformatics*. 2010; doi: 10.1186/1471-2105-11-505.

76. Crosby EJ, Hartman ZC, Lyerly HK. Beyond Neoantigens: Antigens Derived from Tumor Drivers as Cancer Vaccine Targets. *Clin Cancer Res*. 2023; doi: 10.1158/1078-0432.CCR-23-1244.

77. Azhagesan K, Ravindran B, Raman K. Network-based features enable prediction of essential genes across diverse organisms. Mande SC, editor. *PLoS ONE*. 2018; doi: 10.1371/journal.pone.0208722.

78. Hazini A, Fisher K, Seymour L. Deregulation of HLA-I in cancer and its central importance for immunotherapy. *J Immunother Cancer*. 2021; doi: 10.1136/jitc-2021-002899.

79. Taylor BC, Balko JM. Mechanisms of MHC-I Downregulation and Role in Immunotherapy Response. *Front Immunol*. 2022; doi: 10.3389/fimmu.2022.844866.

80. Zaretsky JM, Garcia-Diaz A, Shin DS, Escuin-Ordinas H, Hugo W, Hu-Lieskovan S, et al.. Mutations Associated with Acquired Resistance to PD-1 Blockade in Melanoma. *N Engl J Med*. 2016; doi: 10.1056/NEJMoa1604958.

81. Merck Sharp & Dohme LLC. A Phase 1, Open-Label, Multicenter Study to Assess the Safety and Tolerability of mRNA-5671/V941 as a Monotherapy and in Combination With Pembrolizumab in Participants With KRAS Mutant Advanced or Metastatic Non-Small Cell Lung Cancer, Colorectal Cancer or Pancreatic Adenocarcinoma. clinicaltrials.gov; 2025 Jan. Report No.: NCT03948763.

82. Zhou Z, Wu J, Ren J, Chen W, Zhao W, Gu X, et al.. TSNAD v2.0: A one-stop software solution for tumor-specific neoantigen detection. *Computational and Structural Biotechnology Journal*. 2021; doi: 10.1016/j.csbj.2021.08.016.

83. Wu J, Wang W, Zhang J, Zhou B, Zhao W, Su Z, et al.. DeepHLApan: A Deep Learning Approach for Neoantigen Prediction Considering Both HLA-Peptide Binding and Immunogenicity. *Front Immunol*. 2019; doi: 10.3389/fimmu.2019.02559.

84. Rieder D, Fotakis G, Ausserhofer M, René G, Paster W, Trajanoski Z, et al.. nextNEOpi: a comprehensive pipeline for computational neoantigen prediction. Valencia A, editor. *Bioinformatics*. 2022; doi: 10.1093/bioinformatics/btab759.

85. Di Tommaso P, Chatzou M, Floden EW, Barja PP, Palumbo E, Notredame C. Nextflow enables reproducible computational workflows. *Nat Biotechnol*. 2017; doi: 10.1038/nbt.3820.

86. Huber F, Arnaud M, Stevenson BJ, Michaux J, Benedetti F, Thevenet J, et al.. A comprehensive proteogenomic pipeline for neoantigen discovery to advance personalized cancer immunotherapy. *Nat Biotechnol*. 2025; doi: 10.1038/s41587-024-02420-y.

87. Diao K, Chen J, Wu T, Wang X, Wang G, Sun X, et al.. Seq2Neo: A Comprehensive Pipeline for Cancer Neoantigen Immunogenicity Prediction. *Int J Mol Sci*. 2022; doi: 10.3390/ijms231911624.

Table 1 Click here to access/download;Table;Table 1.pdf

Feature	EGG	pVACtools	ScanNeo2	INTEGRATE-neo	nextNEOpi	TSNAD v2.0	NeoHunter	NeoDisc	SNAF	Seq2Neo	TransHLA
End-to-end sequencing pipeline	✓	✓	✓	✓	✓	✓	✓	✓	✓	✓	—
Modular tool suite	✓	✓	—	—	—	—	—	—	—	—	—
SNV/indel neoantigens	✓	✓	✓	—	✓	✓	✓	✓	—	✓	—
Fusion neoantigens	✓	✓	✓	✓	✓	✓	✓	✓	—	✓	—
Alternative/splicing neoantigens	✓	✓	✓	—	—	—	✓	✓	✓	—	—
Noncanonical / proteogenomic antigens	—	—	—	—	—	—	—	✓	—	—	—
MHC class I	✓	✓	✓	✓	✓	✓	✓	✓	✓	✓	✓
MHC class II	✓	✓	✓	—	✓	—	—	✓	✓	✓	✓
HLA typing included / supported	✓	✓	✓	—	✓	✓	✓	✓	✓	✓	Not required
Agretopicity / mutant-vs-WT comparison	✓	✓	—	—	—	—	✓	—	—	—	—
Immunogenicity / presentation prediction	✓	✓	✓	—	✓	✓	✓	✓	✓	✓	✓
DepMap-based gene essentiality	✓	—	—	—	—	—	—	—	—	—	—
Patient-specific source-gene network context	✓	—	—	—	—	—	—	—	—	—	—
PPI-informed network context	✓	—	—	—	—	—	—	—	—	—	—
Network topology metrics	✓	—	—	—	—	—	—	—	—	—	—
Membrane/surface-gene enhancement	✓	—	—	—	—	—	—	—	✓	—	—
Gene labels (eg. IntOGen and GO)	✓	✓	—	—	✓	—	—	✓	—	—	—
Multivariable prioritization	✓	✓	✓	—	✓	✓	✓	✓	✓	✓	—
Configurable weighted consensus ranking	✓	—	—	—	—	—	—	—	—	—	—
Ranked final neoepitope table	✓	✓	✓	✓	✓	✓	✓	✓	✓	✓	—
Prediction score output	✓	✓	✓	✓	✓	✓	✓	✓	✓	✓	✓

Figure4

Click here to access/download;Figure;Figure4.pdf

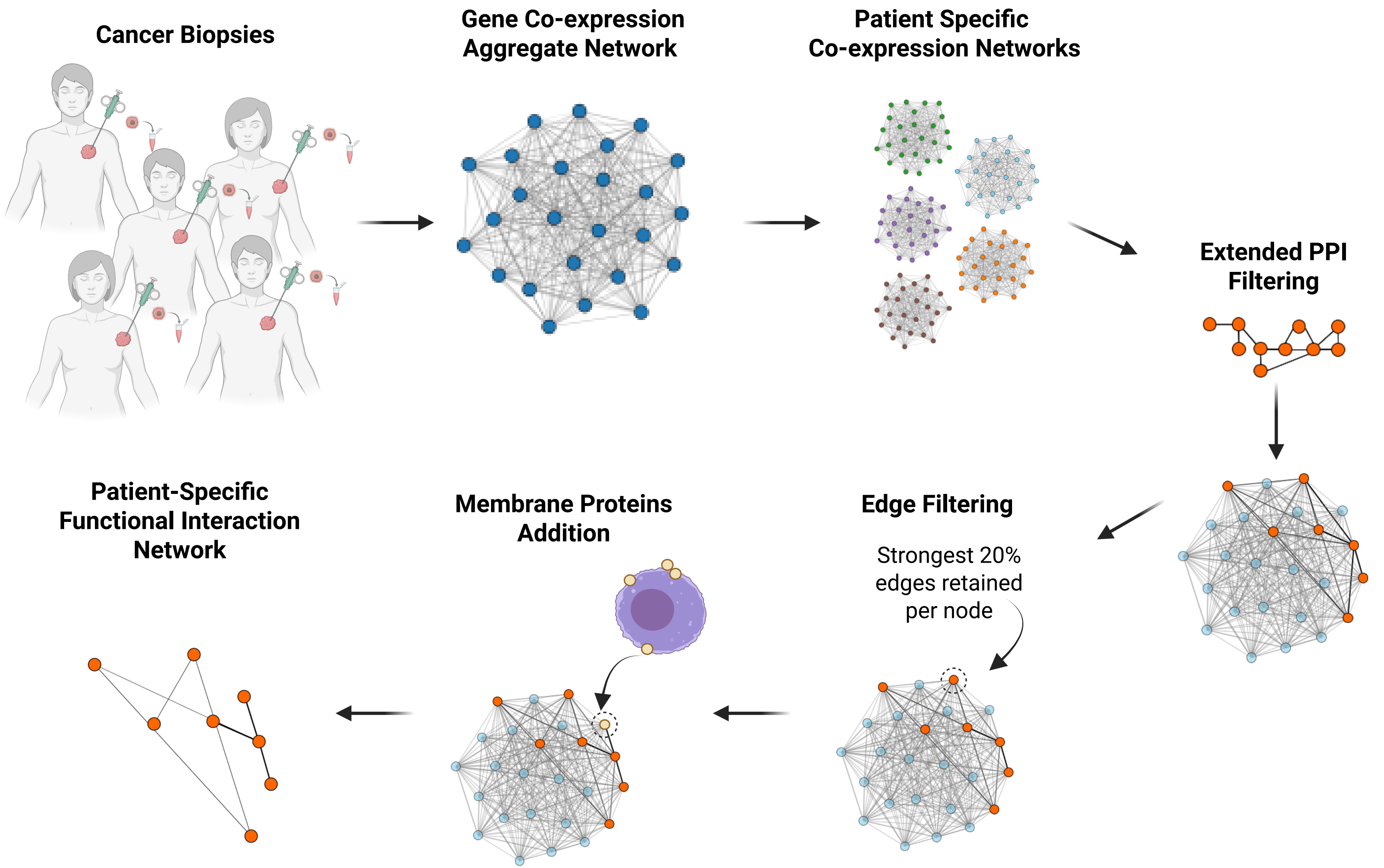

Figure5

Click here to access/download;Figure;Figure5.pdf

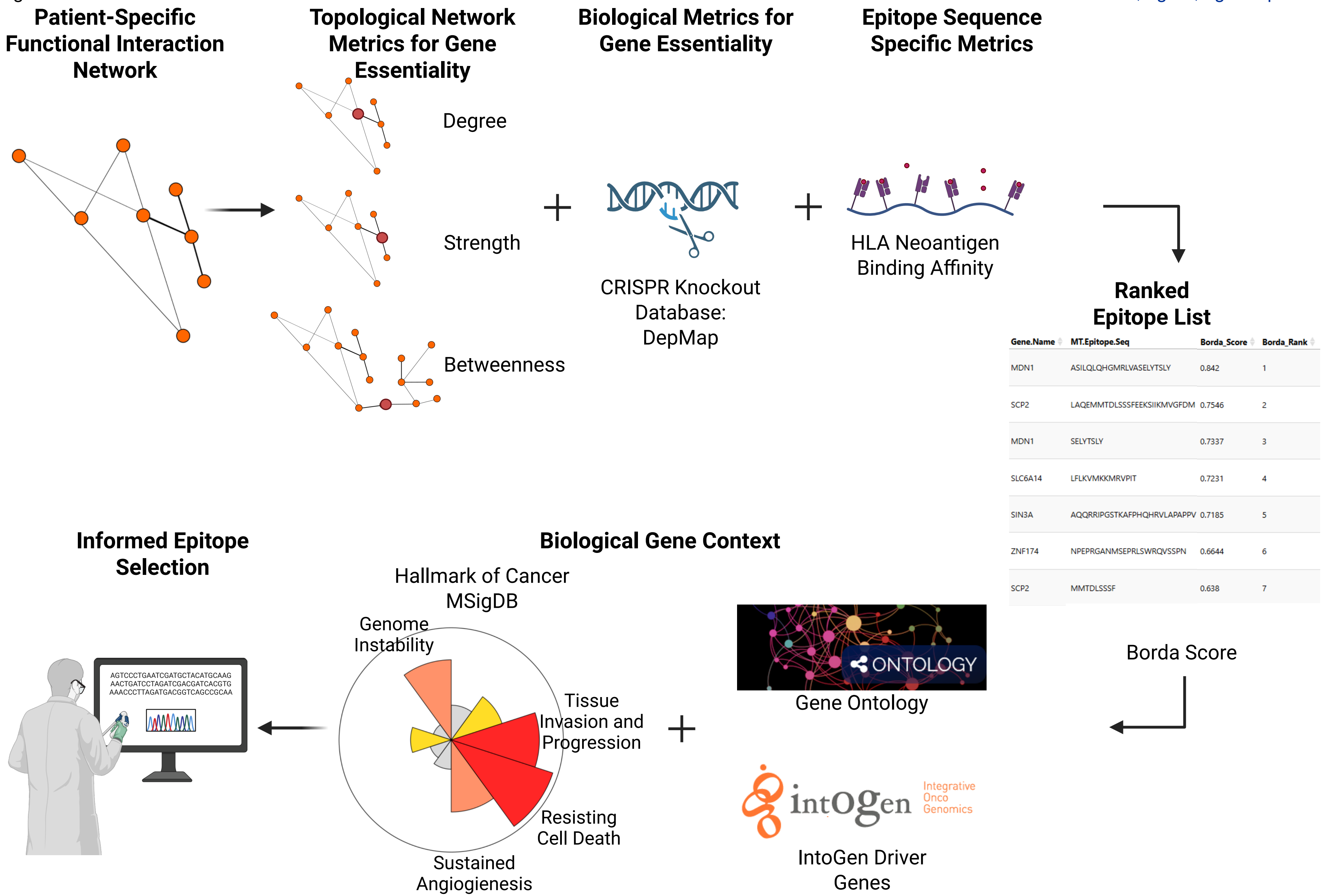

Figure 1

Click here to access/download;Figure;Figure1.pdf

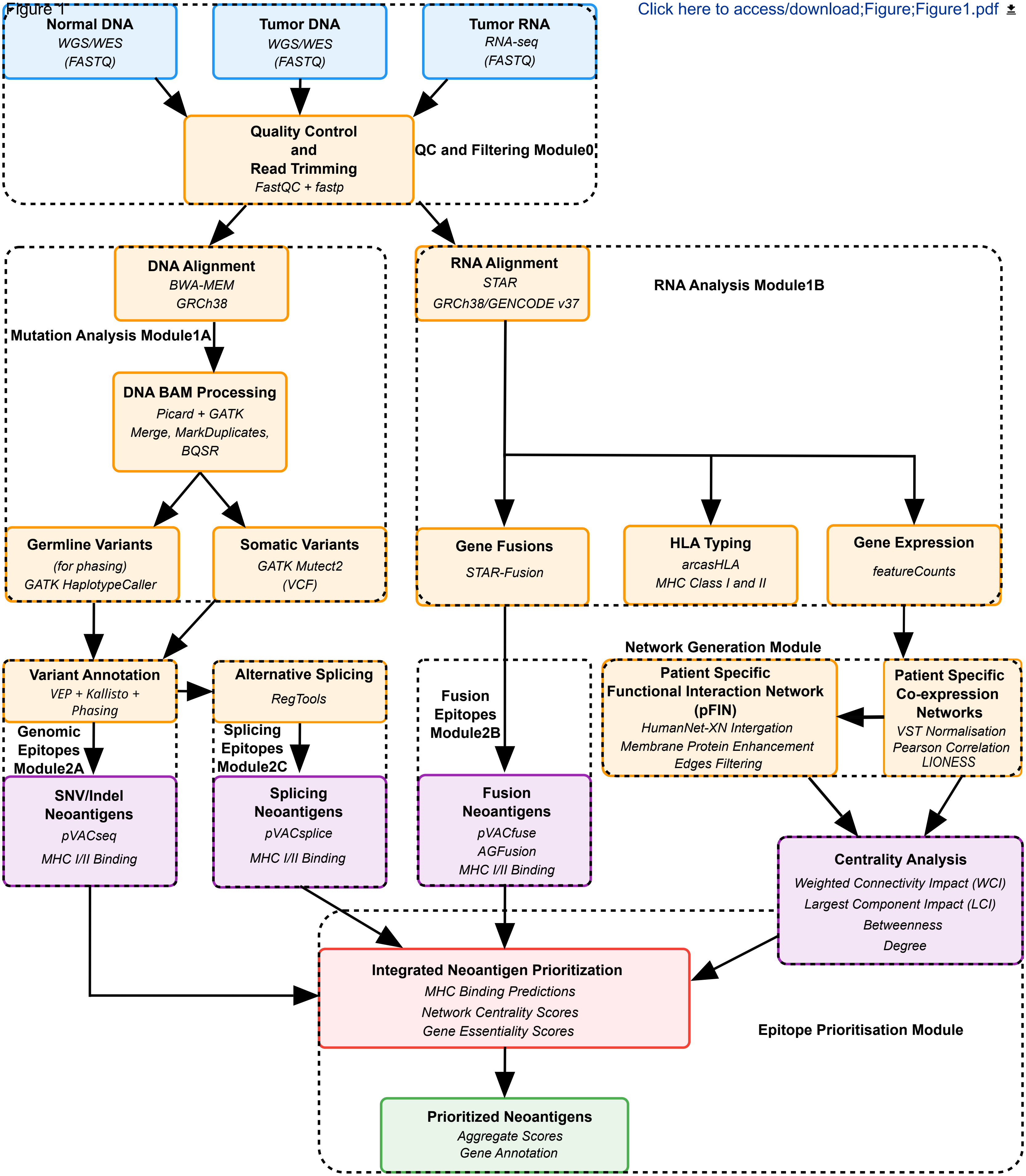

Figure 2

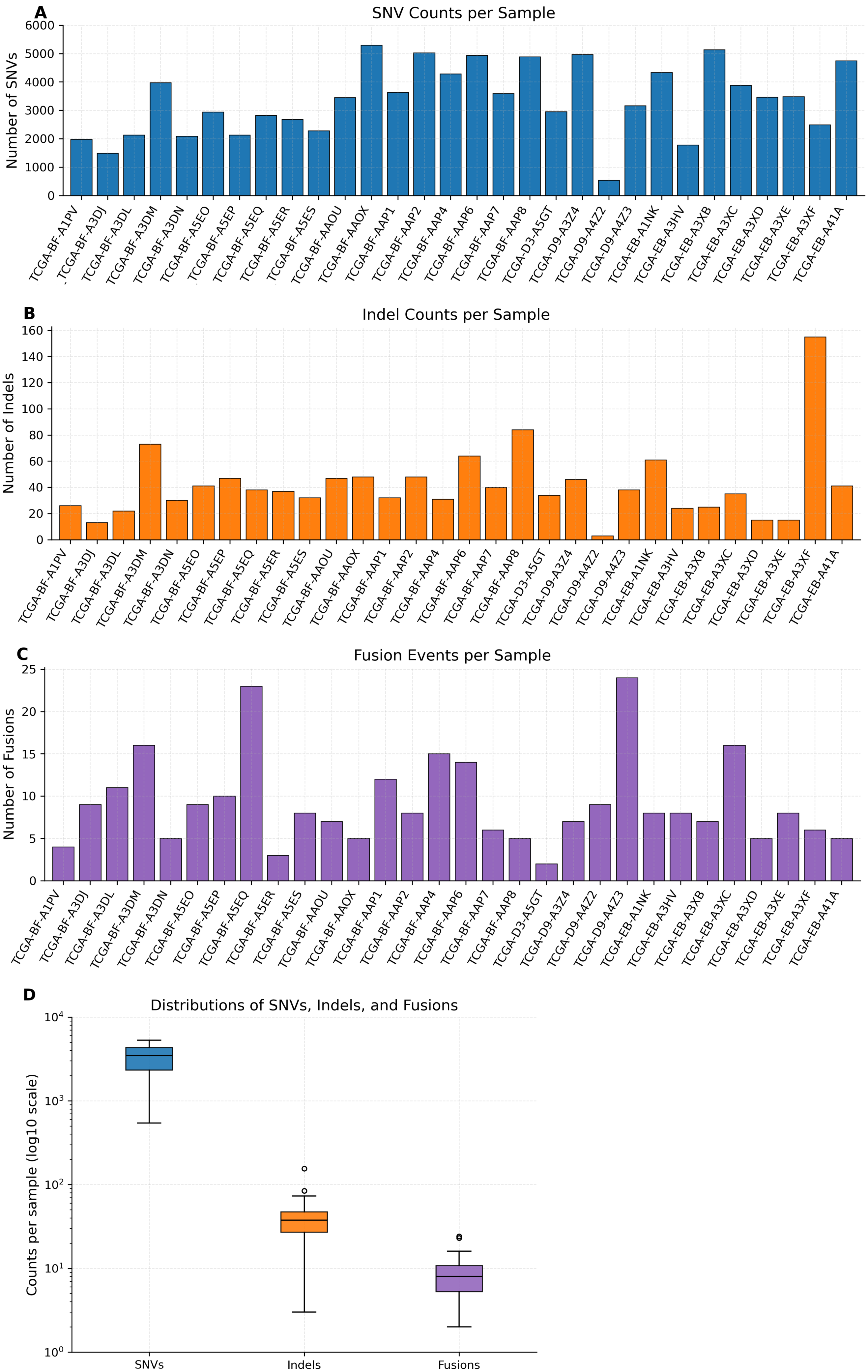
A
SNV Counts per Sample
Number of SNVs
0
1000
2000
3000
4000
5000
6000
TCGA-BF-A1PV
TCGA-BF-A3DJ
TCGA-BF-A3DL
TCGA-BF-A3DM
TCGA-BF-A3DN
TCGA-BF-A5EO
TCGA-BF-A5EP
TCGA-BF-A5EQ
TCGA-BF-A5ER
TCGA-BF-A5ES
TCGA-BF-AAOU
TCGA-BF-AAOX
TCGA-BF-AAP1
TCGA-BF-AAP2
TCGA-BF-AAP4
TCGA-BF-AAP6
TCGA-BF-AAP7
TCGA-BF-AAP8
TCGA-D3-A5GT
TCGA-D9-A3Z4
TCGA-D9-A4Z2
TCGA-D9-A4Z3
TCGA-EB-A1NK
TCGA-EB-A3HV
TCGA-EB-A3XB
TCGA-EB-A3XC
TCGA-EB-A3XD
TCGA-EB-A3XE
TCGA-EB-A3XF
TCGA-EB-A41A
B
Indel Counts per Sample
Number of Indels
0
20
40
60
80
100
120
140
160
C
Fusion Events per Sample
Number of Fusions
0
5
10
15
20
25
D
Distributions of SNVs, Indels, and Fusions
Counts per sample (log10 scale)
10^0
10^1
10^2
10^3
10^4
SNVs
Indels
Fusions

Figure 3

Click here to access/download;Figure;Figure3.pdf

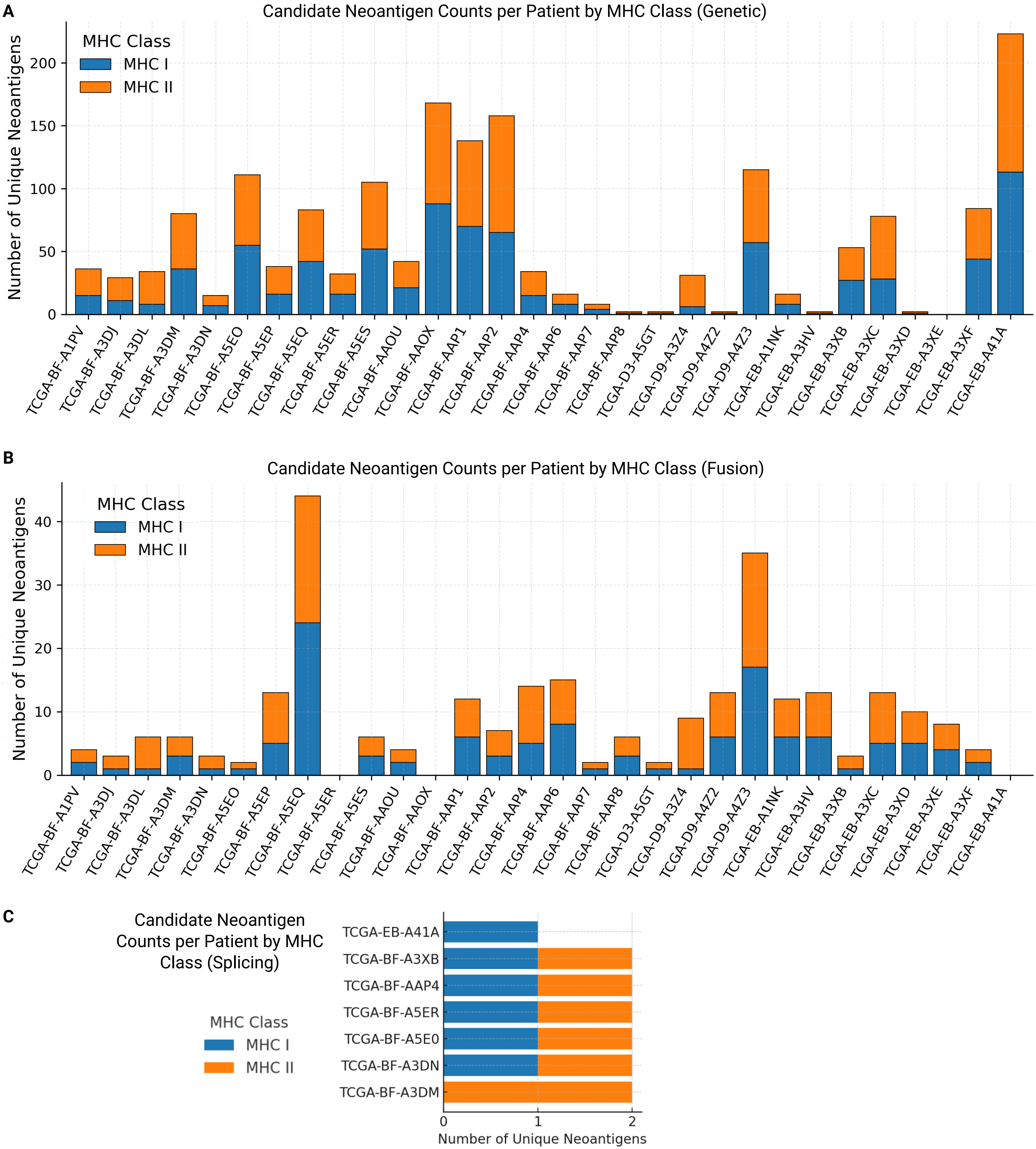

Figure 6

Click here to access/download;Figure;Figure6.pdf

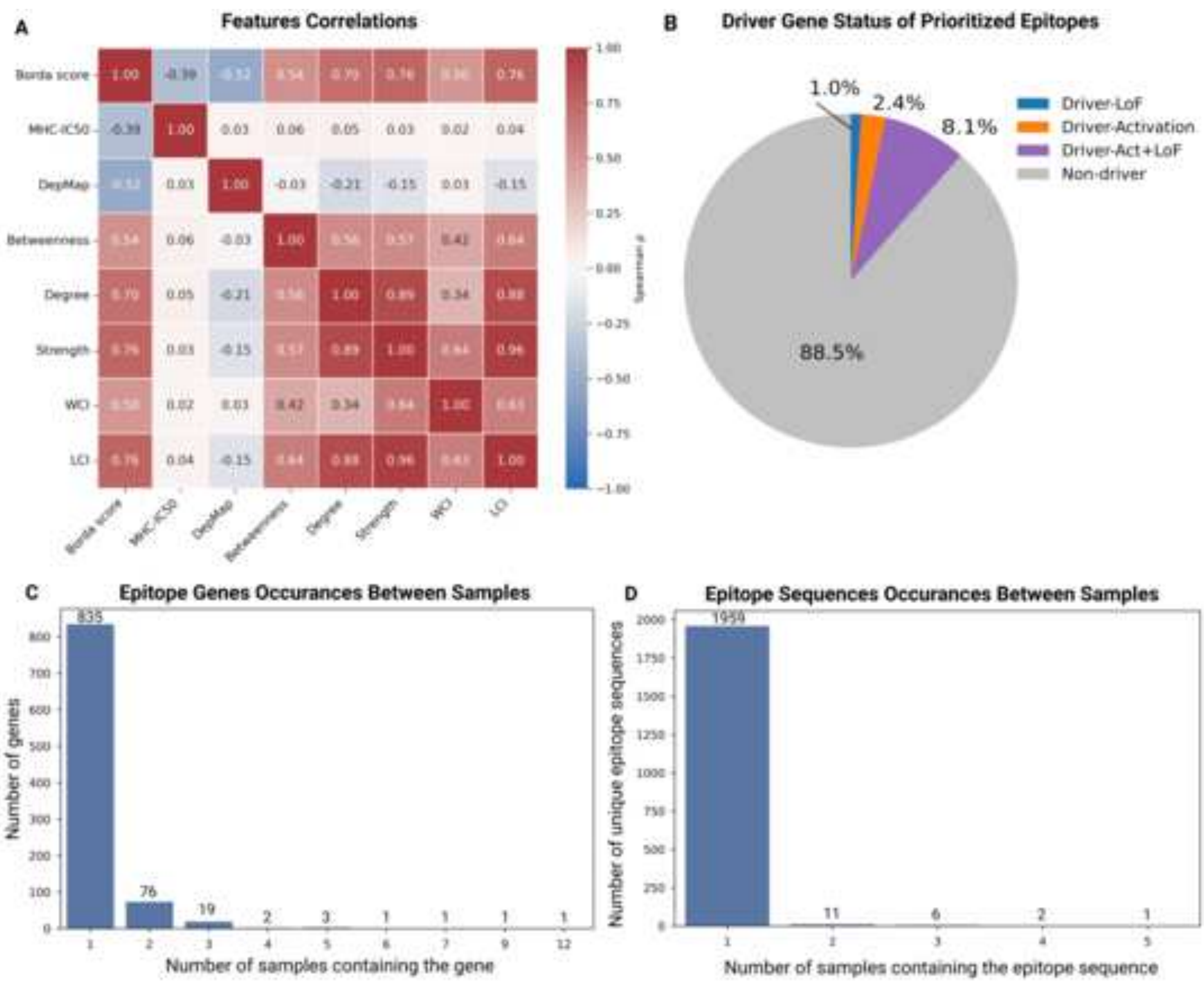

Click here to access/download
Supplementary Material
Supplementary Figure 1.pdf

Click here to access/download
Supplementary Material
Supplementary Figure 2.pdf

Supplementary Material Figure3

Click here to access/download

Supplementary Material

Supplementary Figure 3.pdf

Supplementary Material Figure4

Click here to access/download
Supplementary Material
Supplementary Figure 4.pdf

Supplementary Material Figure5

Click here to access/download
Supplementary Material
Supplementary Figure 5.pdf

Click here to access/download
Supplementary Material
Supplemetary Figure 6.pdf

Click here to access/download
Supplementary Material
Additional file 2 Supplementary Material.xlsx

Click here to access/download
Supplementary Material
Additional file 1_Supplementary Material.docx

Click here to access/download
Supplementary Material
Additional file 1_Supplementary Material_TrackChanges.docx

Click here to access/download
Supplementary Material
Additional file 3_Supplementary_Material.docx

Click here to access/download
Supplementary Material
Additional file 3_Supplementary_Material_Track_Changes.docx

Click here to access/download
Supplementary Material
Answers to reviewer comments 2.docx

Supplementary Material Figure 7

Click here to access/download
Supplementary Material
Supplementary Figure 7.pdf

Supplementary Material Figure 8

Click here to access/download
Supplementary Material
Supplementary Figure 8.pdf

Personal Cover
Click here to access/download;Personal Cover;cover_letter_revision_2.docx
TUNI Luottamuksellinen - Confidential (3Y)

Tampere, August 5^{ft}, 2026

Dear Editor of GigaScience,

Please find enclosed the revised version of our manuscript entitled "Epitope Generation Gateway (EGG): A biological context-dependent approach to cancer vaccine development", by Luca Mannino, Michele Fratello, Jacopo Chiaro, Federica D'Alessio, Emanuele Di Lieto, Minna Niittykoski, Angela Serra, Seppo Ylä-Herttuala, Vincenzo Cerullo, Antonio Federico, and Dario Greco, to be considered for publication in GigaScience.

We have carefully addressed the remaining reviewer comments and amended the manuscript and Supplementary Information accordingly. Point-by-point responses to the Editor's and reviewers' comments are provided in a separate response file.
As requested by the Editor, EGG has been registered in bio.tools (biotoolsID: epitope_generation_gateway; https://bio.tools/epitope_generation_gateway) and SciCrunch (RRID: SCR_028792).

The computational workflow has also been archived in WorkflowHub (DOI: 10.48546/workflowhub.workflow.2165.2). The scripts used to carry out these analyses are available on GitHub at https://github.com/fhaive/epitope_generation_gateway/tree/revision_scripts.

Sincerely yours,

Professor Dario Greco
Faculty of Medicine and Health Technology
Tampere University
Tampere, Finland

Division of Pharmaceutical Biosciences
Faculty of Pharmacy
University of Helsinki
Helsinki, Finland

Email: dario.greco@helsinki.fi
