# Supplementary material for: Epitope Generation Gateway: A biological context-dependent approach to cancer vaccine development
Source: Gigascience. 2026 Oct 1;15:giag095. doi: 10.1093/gigascience/giag095 (PMC13679409; doi:10.1093/gigascience/giag095)
Supplement: Supplementary file 6 [file giag095_reviewer_1_report_original_submission.pdf]

Reviewer Report

Title: Epitope Generation Gateway (EGG):

A biological context-dependent approach to cancer vaccine development

Version: Original Submission **Date:** 2/25/2026

Reviewer name: Reviewer 1

Reviewer Comments to Author:

This manuscript presents EGG, a computational framework designed to prioritize neoantigen candidates by integrating conventional epitope prediction with gene-level measures of functional indispensability derived from co-expression networks, protein-protein interaction data, and CRISPR dependency scores. The authors aim to address the problem of immune escape driven by loss of dispensable tumor antigens under immune pressure.Overall, the manuscript describes a thoughtfully engineered and reproducible computational pipeline. The integration of mutation calling, HLA typing, epitope prediction, patient-specific network construction, and multi-criteria ranking using Borda aggregation is technically solid and clearly implemented. Great attention to reproducibility is shown through well documented repositories, containerization and workflow management.Conceptually, the attempt to incorporate tumor systems biology into neoantigen prioritization represents a meaningful step beyond binding affinity-only approaches.However, several conceptual and methodological issues should be addressed before the manuscript is suitable for publication.*****************A - Major comments and questions:A1: I am wondering why the hardcoded membrane skew is necessary. I see that two papers are cited and one seems to report better survival for tumors with higher number of membrane-derived neoantigens. I can't help but wonder, however, if there is a potential bias here, especially in low-mutational burden tumors. Perhaps this membrane-targeting should be optional or extended to other localisations? So that the used can define what they want to prioritize?Also, I have noticed that there is no mention of the membrane-derived peptides in the results. Has your method recovered any? This warrants investigation since the authors make a point of mentioning it four times and then this disappears later on in the manuscriptA2: Major issues with flow. In the section "Integrating gene essentiality signals into epitope candidate ranking " the text needs tightening. There is a lot of redundancy, some parts read more like a discussion. Borda as well as DepMap explanations are mentioned several (two, three) times and are redundant and are explained in different ways with no new information. This section would gain in clarity from a text tightening.A3: In the section around Figure 2, there is a sentence: "This comprehensive mutational profiling demonstrates the ability of EGG to capture the full spectrum of genomic alterations that can generate immunologically relevant neoantigens, positioning it as a robust tool for personalized cancer immunotherapy target identification. "From what I understand, this section simply shows the mutational burden and indeed and fusion recovered by the tools. I don't think at this stage this results demonstrates anything about EGG, considering there is no peptide prioritization at this stage and it's simply applying the tools. If this is correct, authors should soften the conclusion of that

sentence to reflect what the result truly shows.A4: Could authors comment on the large variability in peptides detected in Figure 3A and 3B? Is there a correlation with the number of SNV per sample? This would add precious information about EGG's behavior and limitsA5: PPI are typically one of things that are used for the construction of gene ontologies and gene pathway annotations. It actually looks like this relationship is partially recovered in the heat map in Figure 6A, where Borda correlates with the graph metrics. Could the authors comments on the actual impact of adding or removing the gene ontologies in the final antigen prioritization? Is it necessary?A6: Authors write: "The fraction of epitopes deriving from known cancer drivers is enriched among the top prioritized epitopes compared to the overall pool" and refer to the pie chart. To me, enrichment is usually quantified with a statistical test. Looking at values alone, I wouldn't say it's particularly enriched, considering the largest proportion is the overall pool. Could authors clarify what they mean by enriched (and possibly perform a statistical test to quantify enrichment)?A7- A key limitation of the current study is the absence of benchmarking against experimentally validated neoantigens. Several melanoma epitopes have been previously identified and functionally validated, including targets derived from TCGA-associated datasets and follow-up immunological studies. Comparing EGG-prioritized candidates against such known immunogenic epitopes would provide an essential test of the framework's central premise, namely, that integrating gene indispensability improves the biological relevance of predicted targets. Even a retrospective analysis demonstrating that validated epitopes rank higher under the EGG framework than under conventional binding-based approaches would substantially strengthen the manuscript and provide empirical support for its proposed advantage.A8- Another point that warrants clarification is the apparent enrichment of longer peptide sequences among the top-ranked candidates (e.g., a 29-amino acid peptide). Antigen binding prediction algorithms (particularly for HLA class II) are known to exhibit length-related biases, often favoring longer peptides due to the flexibility of binding cores and scoring heuristics. It would therefore be important to determine whether peptide length is inadvertently influencing the EGG ranking. Providing a comparison of peptide length distributions before and after prioritization, and assessing whether length correlates with final rank, would help rule out unintended algorithmic bias and ensure that prioritization reflects biological relevance rather than systematic properties of the prediction tools.A9- Since the framework is motivated by evolutionary durability, it would be informative to assess whether top-ranked epitopes preferentially derive from clonal versus subclonal mutations. Incorporating or reporting cancer cell fraction estimates would help align the prioritization with the stated goal of evolutionary stability.A10- To me the merge between DepMap and pFINs is non-trivial. Since DepMap is constructed over a multitude of tumors and pFINs are patient-specific, how do authors justify using pan-cancer dependency scores as proxies for patient-specific tumor essentiality?A11- While the manuscript acknowledges tumor-intrinsic escape mechanisms, it does not explicitly discuss the limitation that immune-side determinants of immunogenicity beyond HLA binding (e.g., antigen processing efficiency, TCR repertoire constraints, immunodominance) are not modeled. I understand this is likely out of scope, since the authors sought to tackle the immune evasion problem from immune pressure, but I believe that clarifying the intended scope of the framework would strengthen the conceptual positioning of the study.****************B- Minor comments and typos:B1: There needs to be consistency in the formula letters used. For instance, N is used for the number of samples, number of genes as well as the scaling factor from the LIONESS algorithm. Alpha is not defined in the LIONESS-derived formula. It would make it a lot easier to follow if there is

consistency.B2- Authors should mention the number of genes they retain once they prune them out to give reader a sense of the network size.B3- In the gene co-expression graph, authors mention constructing a patient specific graph and they filter genes with less than 15 reads in 20% of samples. Am I understanding correctly that authors want genes included in the graph to be expressed in at least 24 people out of 30? Could authors comment on how stringent this is in then influencing their personalized network constructions?B4- Authors write "(..) can identify not just any neoantigens, but those most likely able to elicit appropriate immune responses and to remain resistant to evolutionary changes in the cancer. To address this challenge, we developed ". That sentence should be rephrased because authors have not addressed immune response, they only tackle evolutionary pressure.B5- In the results section, all tools used should be properly cited.B6- "with other optional pvactools algorithms outputs ", pVACtools should be capitalized properlyB7- When authors mention "Across the TCGA melanoma cohort", is this the entire melanoma cohort, or the 30 samples they selected? This sentence should be edited to clarify.B8- When authors write: "bridging the gap between computational prediction and the practical constraints of vaccine formulation." I'm not sure I follow what the practical constraint is. Once the reader finishes the entire manuscript, my best guess is the practical constraint is the number of peptides to deliver in the vaccine. Perhaps clarifying that sentence would help dispel any ambiguity.B9- Authors should always label the corresponding figure number when writing about a specific panel. Example "(12 samples; panel C)" should be Figure XC etc.B10- Authors should add the MSI abbreviation to their list of abbreviations and define it in the text.*****************C- Figure-related comments.C1- In Figure 1, the Modules 0,1 etc. should be numbered for better reference for readers.C2- Figure 6 needs polishing. The y and x ticks are merging with the negative signs. The labels still have underscores in them and are hard to read. C and D are ambiguous: does 1 mean 1 other person has the same peptide (2 occurences) or 1 means only 1 person has the peptide (1 occurence)?

Level of Interest

Please indicate how interesting you found the manuscript: Choose an item.

Quality of Written English

Please indicate the quality of language in the manuscript: Choose an item.

Declaration of Competing Interests

Please complete a declaration of competing interests, considering the following questions:

- Have you in the past five years received reimbursements, fees, funding, or salary from an organisation that may in any way gain or lose financially from the publication of this manuscript, either now or in the future?
- Do you hold any stocks or shares in an organisation that may in any way gain or lose financially from the publication of this manuscript, either now or in the future?

- Do you hold or are you currently applying for any patents relating to the content of the manuscript?
- Have you received reimbursements, fees, funding, or salary from an organization that holds or has applied for patents relating to the content of the manuscript?
- Do you have any other financial competing interests?
- Do you have any non-financial competing interests in relation to this paper?

If you can answer no to all of the above, write 'I declare that I have no competing interests' below. If your reply is yes to any, please give details below.

I declare that I have no competing interests

I agree to the open peer review policy of the journal. I understand that my name will be included on my report to the authors and, if the manuscript is accepted for publication, my named report including any attachments I upload will be posted on the website along with the authors' responses. I agree for my report to be made available under an Open Access Creative Commons CC-BY license (http://creativecommons.org/licenses/by/4.0/). I understand that any comments which I do not wish to be included in my named report can be included as confidential comments to the editors, which will not be published.

I agree to the open peer review policy of the journal

To further support our reviewers, we have joined with Publons, where you can gain additional credit to further highlight your hard work (see: https://publons.com/journal/530/gigascience). On publication of this paper, your review will be automatically added to Publons, you can then choose whether or not to claim your Publons credit. I understand this statement.

Yes Choose an item.
