# Supplementary material for: Epitope Generation Gateway: A biological context-dependent approach to cancer vaccine development
Source: Gigascience. 2026 Oct 1;15:giag095. doi: 10.1093/gigascience/giag095 (PMC13679409; doi:10.1093/gigascience/giag095)
Supplement: Supplementary file 7 [file giag095_reviewer_1_report_revision_1.pdf]

Reviewer Report

Title: Epitope Generation Gateway (EGG):

A biological context-dependent approach to cancer vaccine development

Version: Revision 1 **Date:** 6/25/2026

Reviewer name: Reviewer 1

Reviewer Comments to Author:

I thank the authors for a thorough and largely responsive revision. The pipeline is well engineered and the rebuttal resolves most of my methodological concerns. A few points remain, centered on the validation analysis and on scoping the claims to match the evidence.1. I'm still having trouble reconciling that presentation and immunogenicity are not durability.The paper's central thesis is that ranking by source-gene essentiality and network centrality yields targets the tumor is less able to silence under immune pressure.However, I think matching against IEDB/CEDAR/TSNAdb, at best, tells us a candidate peptide is more likely to be presented or T-cell-reactive.It says nothing about whether that candidate's source gene is harder to lose during tumor evolution.So the entire durability claim (the reason the network layer exists) is untouched by this analysis.I suggest authors frame the durability advantage as a hypothesis rather than something that has been validated.2. I'm also wondering if the minimum 8 containment is a good threshold given peptides vary by length. Wouldn't a % match be a better threshold? I fear that some of the result is coming from peptides that are of a longer size and for which 8 amino acid match brings them somewhere very different. Could the authors clarify if this is affected?3. Is assuming purity =1 a good measure? I wonder if this inflates the VAF interpretation.Ex: melanomas are 0.3-0.7. Have the authors tested with other purity values?4. Maybe a very minor point:â€¨The workflow is described as "lightweight," but the new benchmark reports ~75 GB peak memory for the membrane-enrichment step; the framing should be tempered.A few remaining items I noticed in the revised text:* Figure 3: the body states "up to 2240 epitopes in a single sample" while the Figure 3 caption states "up to 224 epitopes." Please reconcile â€” this looks like a residual error from the recount.* A sentence in the Patient2-Specific Networks Methods ("Gene level raw read count matrices previously produced…") is duplicated.* "Supplementray" is misspelled in several places, including the Additional file 3 description.Final point:The Abstract and title still state that EGG prioritizes "durable cancer vaccine targets" and reduces "the likelihood of immune escape." These are efficacy claims that the current validations do not establish and so given the appropriately scoped limitation now in the Discussion, please bring the Abstract (and ideally the title framing) into line so the claims match the evidence.

Level of Interest

Please indicate how interesting you found the manuscript: Choose an item.

Quality of Written English

Please indicate the quality of language in the manuscript: Choose an item.

Declaration of Competing Interests

Please complete a declaration of competing interests, considering the following questions:

- Have you in the past five years received reimbursements, fees, funding, or salary from an organisation that may in any way gain or lose financially from the publication of this manuscript, either now or in the future?
- Do you hold any stocks or shares in an organisation that may in any way gain or lose financially from the publication of this manuscript, either now or in the future?
- Do you hold or are you currently applying for any patents relating to the content of the manuscript?
- Have you received reimbursements, fees, funding, or salary from an organization that holds or has applied for patents relating to the content of the manuscript?
- Do you have any other financial competing interests?
- Do you have any non-financial competing interests in relation to this paper?

If you can answer no to all of the above, write 'I declare that I have no competing interests' below. If your reply is yes to any, please give details below.

I declare that I have no competing interests

I agree to the open peer review policy of the journal. I understand that my name will be included on my report to the authors and, if the manuscript is accepted for publication, my named report including any attachments I upload will be posted on the website along with the authors' responses. I agree for my report to be made available under an Open Access Creative Commons CC-BY license (http://creativecommons.org/licenses/by/4.0/). I understand that any comments which I do not wish to be included in my named report can be included as confidential comments to the editors, which will not be published.

I agree to the open peer review policy of the journal

To further support our reviewers, we have joined with Publons, where you can gain additional credit to further highlight your hard work (see: https://publons.com/journal/530/gigascience). On publication of this paper, your review will be automatically added to Publons, you can then choose whether or not to claim your Publons credit. I understand this statement.

Yes Choose an item.
