# Supplementary material for: Epitope Generation Gateway: A biological context-dependent approach to cancer vaccine development
Source: Gigascience. 2026 Oct 1;15:giag095. doi: 10.1093/gigascience/giag095 (PMC13679409; doi:10.1093/gigascience/giag095)
Supplement: Supplementary file 8 [file giag095_reviewer_2_report_original_submission.pdf]

Reviewer Report

Title: Epitope Generation Gateway (EGG):

A biological context-dependent approach to cancer vaccine development

Version: Original Submission **Date:** 3/3/2026

Reviewer name: Reviewer 2

Reviewer Comments to Author:

The manuscript introduces the Epitope Generation Gateway (EGG), a containerized Snakemake pipeline designed to bridge the gap between raw neoantigen discovery and systems-level tumor biology. By integrating established variant-calling workflows with a novel Patient-Specific Functional Interaction Network (pFIN) layer, the authors address a critical limitation in current pipelines: the tendency to prioritize epitopes based on sequence-level affinity while overlooking the evolutionary durability and functional essentiality of the source genes.The methodological frameworkâ€”leveraging single-sample co-expression inference (LIONESS) and multi-feature Borda rank aggregationâ€"is both well-motivated and timely. We appreciate the authors' commitment to open science by providing a transparent, containerised workflow and for providing a clear proof-of-concept application using 30 TCGA patients with cutaneous melanoma.. This work has significant potential to enhance the rational selection of durable vaccine targets. However, to transition from a conceptual framework to a robust clinical utility, the following points regarding statistical stability, ranking logic, and validation should be addressed.Major Revisions1. Functional Validation of the Prioritization FrameworkThe hypothesis that network-central, functionally essential genes yield more immune-escape-resistant neoantigen targets is biologically compelling and well-supported by the cited literature. While we appreciate the "proof-of-concept" framing, the claims related to durability made throughout the introduction and discussion require more direct support to move beyond speculation.* Recommendation: Ideally, the authors would provide evidence that network-central epitopes are enriched for biologically meaningful targets. Immunogenicity testing of a subset of top-ranked versus lower-ranked epitopes â€" through T-cell reactivity assays or MHC-eluted peptide confirmation â€" would provide the most direct evidence that the network-based prioritization identifies biologically meaningful targets beyond what MHC binding prediction alone achieves.* A more feasible bioinformatic approach would be to perform an enrichment analysis against high-quality immunopeptidomics datasets (e.g., MHC ligands identified by MS from melanoma cell lines) to determine whether epitopes that are specifically elevated in rank by the network-centrality layer â€" relative to their MHC-binding-only rank â€" are more likely to correspond to experimentally presented peptides. This would directly isolate the contribution of the network prioritization module from the baseline pVACtools prediction.2. Evaluation of Network Stability and Limit of Detection (n=30)Integrating the LIONESS framework to estimate patient-specific networks is a compelling methodological feature. However, at a sample size of n = 30, the mathematical stability of the co-expression manifold requires closer examination. In smaller cohorts, leave-one-out interpolations

can be sensitive to sampling variance and transcriptomic outliers.* Network Volatility: Since the standard error of Pearson correlation at n = 30 is high (~0.19), the authors should provide a bootstrapping or jackknife analysis. This would demonstrate that the centrality ranks (WCI/LCI) of the prioritized genes are robust and not driven by stochastic noise.* Edge Consistency: Reporting the Jaccard similarity of the top-ranked edges across the 30 samples would clarify whether the pFINs reflect a unique biological signal or the variance inherent to small-sample interpolation.* External Benchmarking: To demonstrate that EGG captures meaningful tumor biology rather than baseline noise, the authors should compare their centrality ranks against a stable baseline. This could be achieved by using either the full TCGA-SKCM cohort (~470) or a large external melanoma dataset (e.g., from GEO or EGA).3. Empirical Justification of Borda Weights and Sensitivity AnalysisThe Borda aggregation uses a "default" weighting scheme: 0.25 for MHC/DepMap and 0.10 for each network metric. While this system's flexibility is a strength, the manuscript would benefit from a more detailed rationale for these specific values.* Sensitivity Analysis: The authors should perform a sensitivity analysis to demonstrate how the final ranking changes with different weighting scenarios (e.g., weighting MHC binding at 1.0 versus the current multi-layered approach).* Addressing Feature Redundancy: Figure 6A shows a high correlation between several network metrics. Please discuss whether using five separate centrality measures leads to an inadvertent "overweighting" of the network component.4. Impact of Non-PASS Variant Retention and Single-Caller Somatic Variant CallingThe authors deliberately retained non-PASS Mutect2 variants to maximize candidate epitope diversity, and rely on a single tool for both somatic SNV/indel detection (Mutect2) and fusion calling (STAR-Fusion). While both choices are disclosed and the tools themselves are industry standards, their combined effect is a candidate epitope pool of uncertain precision that directly affects all downstream prioritization results.The impact of non-PASS variant retention is never quantified. It is unclear what fraction of top-ranked epitopes per patient derive from non-PASS variants, and whether the reported driver gene enrichment in the top 10 (Figure 6B) holds when the analysis is restricted to PASS-only variants. Furthermore, consensus somatic calling â€” for example adding Strelka2 for SNVs or Arriba for fusions â€” is known to improve precision and would reduce dependence on a single caller's filter thresholds. If the authors prefer to maintain a lightweight single-caller approach for practical reasons, a brief empirical justification should be provided, such as showing that the prioritization results are not materially sensitive to the non-PASS variant inclusion.* Recommendation: The authors should quantify the contribution of non-PASS variants to the top-ranked epitope lists and demonstrate that key findings â€” particularly driver gene enrichment â€” are robust to PASS-only filtering. If consensus calling is not feasible within the scope of this work, a clear justification for the single-caller design should be included in the Methods.5. Impact of Membrane Enrichment StepThe addition of 5% membrane-associated gene edges to the pFINs is motivated by references showing membrane-localized neoantigen enrichment in immunotherapy responders. However, the actual impact of this step on epitope prioritization is never evaluated.* Empirical Assessment: How many of the top-ranked epitopes per patient derive from membrane genes? Does the membrane expansion materially change the rankings compared to those of PPI-filtered networks alone? Although this step is presented as a key design choice, it requires empirical evaluation.Minor Revisions6. Quantitative Tool ComparisonTable 1 provides a helpful feature matrix, but a quantitative comparison would better substantiate the claim that EGG represents a concrete advance over existing tools.* Direct Comparison: A direct comparison â€” such as the overlap between

EGG's top-ranked epitopes and those of other tools on the same 30 patients â€" would concretely demonstrate what the additional prioritization layer contributes.7. Computational Resource RequirementsNo benchmarking of runtime or computational requirements is provided.* Recommendation: Given that EGG processes three data modalities and constructs single-sample networks, prospective users evaluating EGG for clinical or large-scale research use need runtime and memory information.8. Statistical Context for Recurrent EpitopesThe recurrence of KANSL1 (12/30) and NRAS (7/30) is interesting, but no statistical comparison to background recurrence rates is provided.* Clarification: Are these frequencies significantly higher than expected, given the baseline mutation rates of these genes in TCGA-SKCM? Without a null model, it is unclear whether the recurrence reflects a specific biological signal or simply the known mutational frequency in melanoma.9. Exclusion of HLA Expression from RankingBecause HLA expression levels are important for neoantigen presentation probability and expression data is already processed within EGG, the rationale for excluding it from the default ranking should be explicitly stated.Overall, EGG represents a thoughtful and timely contribution to the neoantigen discovery landscape. The integration of patient-specific network biology with established prediction tools is a conceptually strong advance, and the authors have built a flexible, well-documented pipeline that the community will find useful. The concerns raised in this review are intended constructively and primarily center on strengthening the empirical foundation of the prioritization framework, clarifying several methodological choices, and ensuring that the scope of the conclusions is proportionate to the current evidence. We are confident that addressing these points will substantially strengthen the manuscript and its impact, and we encourage the authors to consider experimental validation as a particularly powerful path forward. We look forward to reviewing a revised version.

Level of Interest

Please indicate how interesting you found the manuscript: Choose an item.

Quality of Written English

Please indicate the quality of language in the manuscript: Choose an item.

Declaration of Competing Interests

Please complete a declaration of competing interests, considering the following questions:

- Have you in the past five years received reimbursements, fees, funding, or salary from an organisation that may in any way gain or lose financially from the publication of this manuscript, either now or in the future?
- Do you hold any stocks or shares in an organisation that may in any way gain or lose financially from the publication of this manuscript, either now or in the future?
- Do you hold or are you currently applying for any patents relating to the content of the manuscript?

- Have you received reimbursements, fees, funding, or salary from an organization that holds or has applied for patents relating to the content of the manuscript?
- Do you have any other financial competing interests?
- Do you have any non-financial competing interests in relation to this paper?

If you can answer no to all of the above, write 'I declare that I have no competing interests' below. If your reply is yes to any, please give details below.

I declare that I have no competing interests.

I agree to the open peer review policy of the journal. I understand that my name will be included on my report to the authors and, if the manuscript is accepted for publication, my named report including any attachments I upload will be posted on the website along with the authors' responses. I agree for my report to be made available under an Open Access Creative Commons CC-BY license (http://creativecommons.org/licenses/by/4.0/). I understand that any comments which I do not wish to be included in my named report can be included as confidential comments to the editors, which will not be published.

I agree to the open peer review policy of the journal

To further support our reviewers, we have joined with Publons, where you can gain additional credit to further highlight your hard work (see: https://publons.com/journal/530/gigascience). On publication of this paper, your review will be automatically added to Publons, you can then choose whether or not to claim your Publons credit. I understand this statement.

Yes Choose an item.
