# Supplementary material for: Epitope Generation Gateway: A biological context-dependent approach to cancer vaccine development
Source: Gigascience. 2026 Oct 1;15:giag095. doi: 10.1093/gigascience/giag095 (PMC13679409; doi:10.1093/gigascience/giag095)
Supplement: Supplementary file 10 [file giag095_supplemental_files.zip › Additional file 1_Supplementary Material.docx]

**Epitope Generation Gateway (EGG):**

**A biological context dependent approach to cancer vaccine development**

Luca Mannino^a^, Michele Fratello^a^, Jacopo Chiaro^bcde^,  Federica D'Alessio^bcde^, Emanuele Di Lieto^a^, Minna Niittykoski^f^, Angela Serra^agh^, Seppo Ylä-Herttuala^fi^, Vincenzo Cerullo^bcdej^, Antonio Federico^agh^,  Dario Greco^ah†^

† Corresponding Author: dario.greco@helsinki.fi

**Affiliations:**

a: Faculty of Medicine and Health Technology, Tampere University, Tampere, Finland

b: Department of Pharmaceutical Biosciences, University of Helsinki, Faculty of Pharmacy ImmunoViroTherapy Lab, Drug Research Program, Helsinki, Finland

c: Helsinki Institute of Life Science (HiLIFE), University of Helsinki, Helsinki, Finland

d: Translational Immunology Program (TRIMM), Faculty of Medicine Helsinki University, University of Helsinki, Helsinki, Finland

e: Digital Precision Cancer Medicine Flagship (iCAN), University of Helsinki, Helsinki, Finland

f: A.I. Virtanen Institute for Molecular Sciences, University of Eastern Finland, Kuopio, Finland

g: Tampere Institute for Advanced Study, Tampere University, Tampere, Finland

h: Division of Pharmaceutical Biosciences, Faculty of Pharmacy, University of Helsinki, Helsinki, Finland

i: Heart Center and Gene Therapy Unit, Kuopio University Hospital, Kuopio, Finland

j: Department of Molecular Medicine and Medical Biotechnology and CEINGE, Naples University Federico II, Naples, Italy

**Supplementary Materials File 1**

**Pipeline Implementation**

EGG pipeline workflow is structured into seven modular components, each corresponding to a dedicated Snakemake file that can be executed independently with a single command. This modular design facilitates usability, reproducibility, and flexibility across different computational environments. Each module automatically handles environment setup by downloading required reference resources, installing associated Conda environments locally, and retrieving Docker images when needed. Apart from installing Snakemake, Conda and Docker, no additional setup is required, enabling seamless execution across systems. The workflow is summarized as a diagram in Figure 1. The pipeline needs sample metadata and file paths are provided via a standardized CSV file containing required columns: sample_name, fastq_R1, fastq_R2, datatype, and lane. This input format simplifies batch processing of multiple samples and ensures consistent data organization across the pipeline.

QC Filtering Module (Module 0)

This module employs FastQC for sequence quality assessment and fastp for automated adapter trimming and low-quality read filtering. All trimming thresholds and filters are fully configurable via a central YAML configuration file, ensuring downstream analysis proceeds with high-quality data.

Execution: snakemake --use-conda --snakefile scripts/final_scripts/QC_filtering_module_0.smk \

--cores {threads} --config csvfile="sample_metadata. csv" results_dir="output_directory"

Mutation Analysis Module (Module 1A)

Following GATK best practices, this module processes tumor and matched normal DNA through read alignment, base quality score recalibration (BQSR), including panel of normals and known variant resources (dbSNP) to minimize false positives. Somatic variant calling using Mutect2 and HaplotypeCaller identifies germline mutations.

Execution:

snakemake --use-conda --snakefile scripts/final_scripts/mutation_analysis_module_1A.smk \

--cores {threads} --config csvfile="sample_metadata.csv" results_dir="output_directory"

RNA Analysis Module (Module 1B)

Using trimmed Cancer RNA-seq data, this module performs three parallel analyses: STAR-Fusion detects gene fusion events, ArcasHLA conducts HLA typing, and FeatureCounts quantifies gene expression for downstream co-expression network analysis.

Execution: snakemake --use-conda --snakefile scripts/final_scripts/RNA_analysis_module_1B.smk \

--cores {threads} --config csvfile="sample_metadata.csv" results_dir="output_directory"

Genomic Mutation Epitopes Module (Module 2A)

Annotated somatic mutations from the variant calling pipeline are formatted for pVACseq analysis. The module supports allele specific binding prediction from somatic mutations including SNVs and indels.

Execution:

snakemake --use-conda --snakefile scripts/final_scripts/somatic_mutation_epitopes_2A.smk \

--cores {threads} --config csvfile="sample_metadata.csv" results_dir="output_directory"

Fusion Epitopes Module (Module 2B)

RNA-derived fusion transcripts from STAR-Fusion are processed for neoantigen prediction using pVACfuse. The module automatically handles fusion breakpoint processing and peptide candidate generation across junction sites.

Execution:

snakemake --use-conda --snakefile scripts/final_scripts/fusion_epitopes_module_2B.smk \

--cores {threads} --config csvfile="sample_metadata.csv" results_dir="output_directory"

Splicing Epitopes Module (Module 2C)

Tumor-specific alternative splicing events are identified using RegTools and processed through pVACsplice for neoepitope prediction. The module predicts viable epitopes candidate from cancer specific mutations causing alternative splicing events.

Execution:

snakemake --use-conda --snakefile scripts/final_scripts/splicing_epitopes_module_2C.smk \

--cores {threads} --config csvfile="sample_metadata.csv" results_dir="output_directory"

Network Generation Module

This module constructs personalized co-expression networks using LIONESS methodology on Pearson gene co-expression network. Gene level centrality metrics are computed and overlaid with HumanNet-XN protein-protein interaction data to identify functionally important neoantigens.

Execution:

snakemake --use-conda --snakefile scripts/final_scripts/sample_networks_generation.smk \

--cores {threads} --config csvfile="sample_metadata.csv" results_dir="output_directory"

Epitope Prioritisation Module

RNA-seq–derived patient networks and centrality features from the Co-expression Network Context Module served as inputs to a consensus epitope prioritization workflow. For each sample, we combined network‐topology measures with orthogonal evidence streams, including CRISPR-based gene essentiality (DepMap) and epitope-level sequence features such as predicted HLA binding affinity. All features are harmonized, scaled, and integrated through a consensus scoring to yield a robust, epitope rank. Candidate genes are further annotated with cancer hallmarks, Gene Ontology terms, and IntOGen driver status to provide interpretable biological context. The final output is a per sample ranked table that prioritizes neoepitope candidates occurring in biologically central and therapeutically relevant contexts.

Execution:

snakemake --use-conda --snakefile scripts/final_scripts/epitopes_prioritisation.smk \

--cores {threads} --config csvfile="sample_metadata.csv" results_dir="output_directory"

Input Format Requirements

The pipeline requires a CSV metadata file with the following structure:

| **Column** | **Description** | **Example** |
| --- | --- | --- |
| sample_name | Unique sample identifier | SAMPLE_001 |
| fastq_R1 | Path to forward reads | /path/to/sample_R1.fastq.gz |
| fastq_R2 | Path to reverse reads | /path/to/sample_R2.fastq.gz |
| datatype | Sample type (CancerRNA/CancerDNA/NormalDNA) | CancerDNA |
| lane | Lane identifier for file naming | L001 |

The modular architecture enables flexible execution patterns: users can run the complete pipeline end-to-end or execute specific modules independently based on available input data. This design supports both research and clinical applications, delivering biologically informed neoepitope predictions.

To execute EGG, Conda and Snakemake are required; we ran the pipeline using Snakemake installed in a Conda environment. All software dependencies are provisioned automatically—each module creates and uses a local Conda environment or pulls the appropriate Docker image, depending on the tool, so no manual package installation is needed. Docker must therefore be installed and available on the host system.

Below we outline the recommended execution order and key outputs to ensure reproducible runs and interpretable results.

The analysis starts with Module 0 (QC Filtering) to standardize inputs. This step runs FastQC for per-read quality profiling and fastp for adapter/quality trimming and length filtering, producing trimmed FASTQ files plus sample-level QC summaries (read counts pre/post trimming, base-quality distributions, adapter content). We recommend applying Module 0 to all raw FASTQs (RNA and DNA) before any downstream analysis so that variant calling, HLA typing, fusion detection, and expression quantification start from uniformly filtered data. For epitope generation, Module 2A (somatic mutation epitopes) must run after Module 1A (DNA mutation analysis). Module 2B (fusion epitopes) should run after Module 1B (RNA analysis). Modules 2A and 2B are otherwise independent: either can be executed without the other, and 2B does not depend on 1A. Note, however, that 2A requires HLA types. If you use pipeline-derived HLA typing, run 1B before 2A; if you provide HLA alleles externally, 2A can proceed without 1B. Module 2C (splicing epitopes) should be run after both 1B (RNA) and 1A (DNA), as it uses RNA-derived splice events together with matched DNA calls and HLA types for filtering and annotation. For prioritization, we include a configuration file only for the Borda consensus settings (feature order/weights and tie handling). pVACtools options are modified directly in the Snakemake rules, not via this config. The final prioritization table should include, at minimum: sample_id; gene; variant_class (SNV/indel/fusion/splice); peptide; peptide_length; HLA_allele; binding_affinity; binding_rank; expression; network_centrality (e.g., degree, betweenness); DepMap_dependency; subcellular_localization; driver_status (IntOGen); cancer_hallmarks; and its output will include interactive tables that include Borda_consensus_score, final_rank and Gene Ontology plus cancer hallmark enrichment.

The pipeline generates outputs organized by module:

results/

├── 0_Filtering_and_QC/

│	├── trimmed_fastq/	# Quality-filtered FASTQ files

│	├── qc_pre_filtering/	# Initial FastQC reports

│	└── qc_post_filtering/	# Post-trimming QC reports

│

├── 1A_mutation_analysis/

│	├── VCF/	# Raw variant calls

│	├── VCF_filtered/	# Filtered somatic variants

│	├── VCF_germline/	# Germline variant calls

│	├── VCF_germline_filtered/	# Filtered germline variants

│	├── bwa/	# Alignment outputs

│	├── dedup/	# Duplicate-marked BAMs/metrics

│	├── bqsr/	# BQSR-processed BAMs

│	├── merge/	# Merge steps/results

│	├── metrics/	# Alignment/QC metrics

│	└── filtering_tables/	# Variant filtering tables/logs

│

├── 1B_RNA_fusion_HLA/

│	├── ArcasHLA/	# HLA typing results

│	├── RNA_Counts/	# Gene expression quantification

│	├── StarFusionOut/	# Fusion predictions

│	└── sorted_bam/	# Coordinate-sorted RNA BAMs

│

├── 2A_somatic_mutation_epitopes/

│	├── annotated_germline_VCF/	# Annotated germline variants

│	├── annotated_germline_VCF_name_updated/# Germline VCFs with updated naming

│	├── annotated_phased_VCF/	# Annotated phased variants

│	├── annotated_somatic_VCF/	# Annotated somatic variants

│	├── combined_VCF/	# Combined VCFs (somatic/germline/etc.)

│	├── kallisto_quantification/	# Expression quantification (kallisto)

│	├── kallisto_somatic_VCF/	# Somatic VCFs used alongside expression

│	├── phased_VCF/	# Phased VCFs

│	├── pvacSeq/	# Neoepitope predictions

│	├── regtools/	# regtools outputs for splicing evidence

│	├── sorted_VCF/	# Sorted VCFs

│	└── tumor_only_VCF/	# Tumor-only variant calls

│

├── 2B_fusion_epitopes/

│	├── AGfusion/	# Fusion annotations

│	└── pvacFuse/	# Fusion neoepitope predictions

│

├── 2C_splicing_epitopes/

│	├── annotated_somatic_VCF/	# Annotated somatic VCFs (splicing context)

│	├── pvacSplice/	# Splicing neoepitope predictions

│	└── regtools_genomic_VCF_genecode/	# Splice junction calls

│

├── sample_specific_networks/

│	├── Network_Metrics_Betweenness/	# Betweenness centrality per gene

│	├── Network_Metrics_Degree/	# Degree centrality per gene

│	├── Network_Metrics_Full/	# Full metrics outputs

│	│	├── Strength/	# Strength metrics

│	│	└── WCI/	# WCI metrics

│	├── Network_Metrics_LargestComponentImpact/ # LCI scores per gene

│	├── Network_Metrics_Strength/	# Strength centrality per gene

│	├── Sample_Specific_Networks/	# Patient-specific networks

│	├── Sample_Specific_Networks_Distances/ # Network distance computations

│	├── Sample_Specific_Networks_PPI_filtered/

│	│	├── filtered_networks_matrix/

│	│	├── filtered_networks_rds/

│	│	├── filtered_networks_rds_old/

│	│	└── qc_plots/

│	├── Sample_Specific_Networks_PPI_filtered_copy/

│	│	└── filtered_networks_matrix/

│	├── gene_lists/	# Gene sets used for networks

│	└── normalised_counts/	# Normalized expression for network construction

│

└── epitopes_prioritisation/

├── Borda_Epiotpes_Prioritisation/	# Consensus scoring results

├── annotated_epitopes/	# Annotated epitopes

├── annotated_epitopes_with_network/	# Epitopes merged with network features

├── annotationhub_cache/	# AnnotationHub cache

├── combined_epitopes/	# Merged epitopes from all sources

└── final_epitopes/	# Final ranked neoepitope outputs

├── HLA/	# csv final epitopes tables

├── HTML/	# interactive .html final epitopes tables

The pipeline tools require curated reference resources (genomes, annotations, indices) that are downloaded automatically on first run and saved under the resources/ directory. The on-disk footprint for the required resources is around 117 GB.

The required reference resources are organized under resources/ with the following directory structure:

resources/

├genome_DNA/

├genome_RNA_fusion/

│ └─GRCh38_gencode_v37_CTAT_lib_Mar012021.plug-n-play/

├GTF/

├Kallisto_ref/

├PON/

├VEP/

├dbSNP/

├gnomad/

├arcasHLA/

├agfusion/

├WGS_intervals/

└─interval_list/

**Overview of external tools integrated in EGG**

EGG integrates several established third-party tools across the workflow, together with custom R/Python scripts for patient-specific network construction, feature integration, and final epitope prioritisation. The table below summarises the main software components used in the pipeline and their role in each workflow step.

| **EGG workflow step** | **Main tool/software** | **Role in EGG** |
| --- | --- | --- |
| Workflow execution | **Snakemake** | Coordinates the modular EGG workflow across QC, DNA mutation analysis, RNA analysis, epitope prediction, network analysis, and prioritisation. |
| Read QC and preprocessing | **FastQC**, **fastp** | Assesses sequencing read quality, performs adapter/quality trimming, and summarizes QC reports. |
| DNA alignment and BAM processing | **BWA-MEM**, **SAMtools**, **Picard** | Aligns DNA reads to the reference genome and performs BAM sorting, indexing, merging, and related preprocessing. |
| Somatic and germline variant processing | **GATK**, including **Mutect2**, **HaplotypeCaller**, **MarkDuplicatesSpark**, **BaseRecalibrator/ApplyBQSR**, **FilterMutectCalls**, **ReadBackedPhasing** | Performs duplicate marking, base-quality score recalibration, somatic SNV/indel calling, germline variant calling, variant filtering, and phasing. |
| Variant annotation | **Ensembl Variant Effect Predictor (VEP)** | Annotates somatic, germline, and splice-associated variants for downstream epitope prediction. |
| Transcript expression quantification | **kallisto** | Quantifies transcript expression and provides expression information used in mutation-derived epitope annotation. |
| RNA-seq fusion detection | **STAR-Fusion** | Detects candidate gene fusions from RNA-seq data. |
| HLA typing | **arcasHLA** | Infers patient HLA genotypes from RNA-seq data for downstream MHC-binding prediction. |
| Gene expression quantification | **featureCounts** | Generates gene-level RNA-seq count data used by downstream expression and network analyses. |
| Fusion annotation | **AGFusion** | Annotates fusion events detected by STAR-Fusion before fusion-derived epitope prediction. |
| Alternative splicing analysis | **RegTools** | Identifies cis-splice effects from variants and RNA-seq alignments for splice-derived epitope analysis. |
| SNV/indel neoepitope prediction | **pVACseq** | Predicts mutation-derived candidate neoepitopes from annotated VCFs and HLA genotypes. The workflow specifies MHCnuggets class I and II prediction algorithms through pVACtools. |
| Fusion neoepitope prediction | **pVACfuse** | Predicts fusion-derived candidate neoepitopes using fusion calls, fusion annotation, and HLA genotypes. |
| Splice-derived neoepitope prediction | **pVACsplice** | Predicts neoepitopes arising from splice-altering variants using RegTools output, VCF annotation, transcript references, and HLA genotypes. |
| Patient-specific network construction | **LIONESS** | Generates patient-specific gene co-expression networks used for network-based prioritisation. (implemented via custom script not R Package) |
| Network and multi-evidence prioritisation | **Custom R/Python prioritisation scripts** | Computes network features, integrates gene-level evidence such as DepMap essentiality and IntOGen driver annotation, and calculates Borda consensus rankings for candidate epitopes. |

**Supplementary Table S1. Main software tools used in the EGG workflow.**
The table lists the principal third-party tools and custom scripts used across the EGG workflow, grouped by analysis step. For each component, the table summarises its role in quality control, read preprocessing, DNA and RNA processing, variant and fusion detection, HLA typing, epitope prediction, patient-specific network construction, annotation, and final prioritisation. Existing tools are used for established upstream tasks, while custom EGG scripts implement the network-based and multi-evidence prioritisation steps introduced in this work.

**Computational resource benchmark for EGG-specific downstream modules**

To provide practical guidance for users running EGG on local or shared compute infrastructure, we benchmarked the runtime and memory requirements of the two EGG-specific downstream modules: patient-specific network generation and final epitope prioritisation. We focused on these modules because they represent the main EGG-specific computational steps. Earlier stages of the workflow rely largely on established third-party tools for read processing, variant calling, fusion detection, splicing analysis, and neoantigen prediction, whose runtime and memory requirements are documented by the corresponding developers and are strongly affected by sample-specific properties such as sequencing depth, library size, tumour purity, mutation burden, and fusion/splicing event burden. In contrast, the network-generation and prioritisation modules are central to the added functionality of EGG and therefore provide the most informative benchmark of EGG-specific computational requirements.

The network-generation benchmark was run using the same 30 TCGA-SKCM samples analysed in the manuscript. The benchmark used the sample_networks_generation.smk workflow and included RNA count normalisation, LIONESS single-sample network reconstruction, PPI-based network filtering, membrane-edge enrichment, network centrality metric calculation, largest-component impact calculation, full-network WCI/strength calculation, and QC plot generation. Snakemake was run using single-threaded rules, with BLAS/OpenMP threading fixed to one thread per job. Because membrane-edge enrichment was the most memory-intensive per-sample step, this rule was assigned a Snakemake thread reservation to limit concurrent execution while allowing lighter rules to run in parallel.

For network generation, the most memory-intensive step was membrane-edge enrichment, with a median peak resident memory of 75.36 GB per sample and a median runtime of 24.3 min per sample. The longest-running per-sample step was PPI-only filtering, with a median runtime of 2.30 h per sample and a median peak resident memory of 11.03 GB. LIONESS reconstruction was run once for the full 30-sample cohort and required 4.09 h and 36.11 GB peak resident memory. Other downstream network metric calculations were less memory-intensive but varied in runtime: degree/strength/betweenness calculation required a median of 3.49 min per sample, largest-component impact required a median of 51.0 min per sample, and full-network WCI required a median of 55.8 s per sample. These results indicate that users should pay particular attention to memory allocation and concurrency when running the membrane-edge enrichment step, and to walltime allocation for PPI filtering.

The epitope prioritisation benchmark was run across the same 30 samples and included candidate merging and IC50 harmonisation, DepMap/IntOGen annotation, addition of network metrics, Borda rank calculation, GO/Hallmark annotation, and generation of interactive HTML outputs. These steps were comparatively lightweight. Merging epitopes and harmonising IC50 values required a median of 16.0 s per sample, DepMap/IntOGen annotation required 5.3 s, addition of network metrics required 1.85 min, Borda rank calculation required 5.2 s, GO/Hallmark annotation required 36.9 s, and interactive HTML generation required 6.6 s. Peak memory was below approximately 1.3 GB for steps where reliable memory estimates were available, except that some very short rules did not produce stable peak-memory estimates and are therefore reported as not available.

Overall, this benchmark shows that EGG-specific epitope prioritisation is computationally modest after upstream candidate generation and patient-specific network metrics are available. In contrast, the main computational burden is associated with patient-specific network generation, particularly LIONESS reconstruction, PPI filtering, and membrane-edge enrichment; the latter reached approximately 75 GB peak RAM in this benchmark. These results provide practical guidance for adjusting Snakemake concurrency and memory allocation according to available compute resources.

| **Rule / step** | **n** | **Median runtime** | **Max runtime** | **Median peak RAM** | **Max peak RAM** |
| --- | --- | --- | --- | --- | --- |
| RNA normalisation | 1 | 19.3 s | 19.3 s | 0.73 GB | 0.73 GB |
| LIONESS reconstruction | 1 | 4.09 h | 4.09 h | 36.11 GB | 36.11 GB |
| PPI-only filtering | 30 | 2.30 h | 2.47 h | 11.03 GB | 11.04 GB |
| Membrane-edge enrichment | 30 | 24.3 min | 26.6 min | 75.36 GB | 75.37 GB |
| Degree/strength/betweenness metrics | 30 | 3.49 min | 3.82 min | 0.16 GB | 0.17 GB |
| Largest-component impact | 30 | 51.0 min | 56.6 min | 0.29 GB | 0.33 GB |
| Full-network WCI | 30 | 55.8 s | 1.92 min | 11.11 GB | 14.55 GB |

**Supplementary Table S2. Runtime and memory benchmark for EGG patient-specific network generation.** The table reports per-rule runtime and peak resident memory usage for the EGG network-generation workflow applied to the 30 TCGA-SKCM samples analysed in the manuscript. The benchmark was run using sample_networks_generation.smk and included RNA count normalisation, LIONESS single-sample network reconstruction, PPI-only filtering, membrane-edge enrichment, network metric calculation, largest-component impact calculation, full-network WCI/strength calculation, and QC plot generation. Runtime and memory values are reported as medians and maxima across samples where applicable. LIONESS reconstruction and RNA normalisation were run once at the cohort level, whereas subsequent network-processing steps were run per sample.

| **Rule / step** | **n** | **Median runtime** | **Max runtime** | **Median peak RAM** | **Max peak RAM** |
| --- | --- | --- | --- | --- | --- |
| Merge previous outputs | 30 | 16.0 s | 16.1 s | 0.08 GB | 0.11 GB |
| DepMap/IntOGen annotation | 30 | 5.3 s | 6.8 s | NA | NA |
| Add network metrics | 30 | 1.85 min | 2.95 min | 0.99 GB | 1.26 GB |
| Compute Borda rank | 30 | 5.2 s | 8.5 s | NA | NA |
| GO/Hallmark annotation | 30 | 36.9 s | 60.5 s | 0.95 GB | 1.05 GB |
| Build interactive HTML | 30 | 6.6 s | 9.9 s | NA | NA |

**Supplementary Table S3. Runtime and memory benchmark for EGG epitope prioritisation.**
The table reports per-rule runtime and peak resident memory usage for the EGG epitope-prioritisation workflow applied to the 30 TCGA-SKCM samples analysed in the manuscript. The benchmark included epitope merging and IC50 harmonisation, DepMap/IntOGen annotation, addition of network metrics, Borda rank calculation, GO/Hallmark annotation, and interactive HTML generation. Runtime and memory values are reported as medians and maxima across samples where applicable. Peak memory is reported as not available for short rules where stable memory estimates were not collected.
