# Supplementary material for: Epitope Generation Gateway: A biological context-dependent approach to cancer vaccine development
Source: Gigascience. 2026 Oct 1;15:giag095. doi: 10.1093/gigascience/giag095 (PMC13679409; doi:10.1093/gigascience/giag095)
Supplement: Supplementary file 10 [file giag095_supplemental_files.zip › Additional file 3_Supplementary_Material.docx]

**Epitope Generation Gateway (EGG):**

**A biological context dependent approach to cancer vaccine development**

Luca Mannino^a^, Michele Fratello^a^, Jacopo Chiaro^bcde^,  Federica D'Alessio^bcde^, Emanuele Di Lieto^a^, Minna Niittykoski^f^, Angela Serra^agh^, Seppo Ylä-Herttuala^fi^, Vincenzo Cerullo^bcdej^, Antonio Federico^agh^,  Dario Greco^ah†^

† Corresponding Author: dario.greco@helsinki.fi

**Affiliations:**

a: Faculty of Medicine and Health Technology, Tampere University, Tampere, Finland

b: Department of Pharmaceutical Biosciences, University of Helsinki, Faculty of Pharmacy ImmunoViroTherapy Lab, Drug Research Program, Helsinki, Finland

c: Helsinki Institute of Life Science (HiLIFE), University of Helsinki, Helsinki, Finland

d: Translational Immunology Program (TRIMM), Faculty of Medicine Helsinki University, University of Helsinki, Helsinki, Finland

e: Digital Precision Cancer Medicine Flagship (iCAN), University of Helsinki, Helsinki, Finland

f: A.I. Virtanen Institute for Molecular Sciences, University of Eastern Finland, Kuopio, Finland

g: Tampere Institute for Advanced Study, Tampere University, Tampere, Finland

h: Division of Pharmaceutical Biosciences, Faculty of Pharmacy, University of Helsinki, Helsinki, Finland

i: Heart Center and Gene Therapy Unit, Kuopio University Hospital, Kuopio, Finland

j: Department of Molecular Medicine and Medical Biotechnology and CEINGE, Naples University Federico II, Naples, Italy

**Supplementary Materials File 3**

**Relationship between mutations event burden and predicted neoantigen yield**

To assess whether the inter-sample variability in predicted neoantigen counts was primarily explained by differences in input genomic event burden, we correlated candidate neoantigen counts with the corresponding numbers of somatic SNVs, indels, combined SNV + indel events, and fusion events across the 30 TCGA-SKCM samples. Spearman correlation was used because both mutation burden and predicted neoantigen counts varied substantially across samples and were not assumed to follow a linear distribution.

For genomic variant-derived candidates, total predicted neoantigen yield showed only weak and non-significant positive correlations with SNV count (Spearman ρ = 0.264, p-value = 0.1595) and combined SNV + indel count (ρ = 0.287, p-value = 0.1238). Similar weak and non-significant correlations were observed when MHC class I and MHC class II genomic neoantigens were considered separately using combined SNV + indel burden (MHC I: ρ = 0.274, p-value = 0.1429; MHC II: ρ = 0.306, p-value = 0.0997). Indel count alone showed a modest positive correlation with total genomic neoantigen yield (ρ = 0.378, p-value = 0.0392), but indels represented only a small fraction of the total genomic variant burden in these samples, so this association should be interpreted cautiously.

By contrast, fusion-derived neoantigen counts were strongly correlated with the number of detected fusion events. Total fusion-derived neoantigen yield correlated positively with fusion event count (ρ = 0.720, p-value = 7.286 × 10⁻⁶), and this relationship was also observed for both MHC class I fusion candidates (ρ = 0.649, p-value = 1.053 × 10⁻⁴) and MHC class II fusion candidates (ρ = 0.727, p-value = 5.366 × 10⁻⁶). Fusion-derived neoantigen counts were not correlated with SNV count or combined SNV + indel count, indicating that fusion-derived neoantigen variability was driven by fusion burden rather than by overall somatic SNV/indel burden.

Together, these results indicate that predicted neoantigen yield depends on both the number and the type of underlying genomic events. However, genomic variant burden alone does not directly determine the final peptide yield, likely because different variants have unequal protein-coding consequences and because candidate generation also depends on altered peptide sequence context, patient-specific HLA genotype, MHC class, and pVACtools binding and filtering criteria.

| **response** | **response label** | **predictor label** | **samples** | **spearman rho** | **p_value** |
| --- | --- | --- | --- | --- | --- |
| genomic total neoantigens | Genomic total neoantigens | SNV count | 30 | 0.264 | 0.1595 |
| genomic total neoantigens | Genomic total neoantigens | Indel count | 30 | 0.378 | 0.0392 |
| genomic total neoantigens | Genomic total neoantigens | SNV + indel count | 30 | 0.287 | 0.1238 |
| genomic mhcI neoantigens | Genomic MHC I neoantigens | SNV + indel count | 30 | 0.274 | 0.1429 |
| genomic mhcII neoantigens | Somatic MHC II neoantigens | SNV + indel count | 30 | 0.306 | 0.0997 |
| fusion total neoantigens | Fusion total neoantigens | Fusion event count | 30 | 0.720 | 7.286e-06 |
| fusion mhcI neoantigens | Fusion MHC I neoantigens | Fusion event count | 30 | 0.649 | 1.053e-4 |
| fusion mhcII neoantigens | Fusion MHC II neoantigens | Fusion event count | 30 | 0.727 | 5.366e-06 |
| fusion_total_neoantigens | Fusion total neoantigens | SNV count | 30 | -0.028 | 0.8826 |
| fusion_total_neoantigens | Fusion total neoantigens | SNV + indel count | 30 | -0.035 | 0.8531 |

**Supplementary Table S1. Spearman correlations between predicted neoantigen yield and input genomic event burden across TCGA-SKCM samples.** The table reports Spearman correlation coefficients and p-values calculated across the 30 TCGA-SKCM samples to assess whether variability in predicted candidate neoantigen counts was associated with the number of underlying genomic events. Genomic neoantigens correspond to somatic SNV- and indel-derived candidates generated with pVACseq. Fusion neoantigens correspond to fusion-derived candidates generated with pVACfuse using STAR-Fusion input. Correlations were evaluated for total candidate counts and, where indicated, separately for MHC class I and MHC class II candidates. SNV + indel count represents the combined burden of somatic SNVs and indels per sample.

**Supplementary Figure S1. Relationship between input genomic event burden and predicted neoantigen yield across TCGA-SKCM samples.**
(A) Total genomic variant-derived neoantigen yield compared with combined SNV + indel burden. (B) Total genomic variant-derived neoantigen yield compared with SNV burden alone. (C) Total fusion-derived neoantigen yield compared with fusion event burden. Each point represents one TCGA-SKCM sample. Spearman correlation coefficients and p-values were calculated across the 30 samples and are reported in Supplementary Table S1. Genomic variant-derived neoantigens refer to candidates generated from somatic SNV and indel calls using pVACseq, whereas fusion-derived neoantigens refer to candidates generated from STAR-Fusion calls using pVACfuse.

**Impact of non-PASS Mutect2 variant retention on final epitope prioritisation**

To quantify the contribution of non-PASS Mutect2 calls to the final epitope rankings, we annotated somatic pVACseq-derived candidates and final prioritised epitope tables with the FILTER status of the originating VCF record. Variants with VCF FILTER equal to PASS were classified as PASS, while all other FILTER values were classified as non-PASS. Fusion- and splicing-derived candidates were analysed separately because Mutect2 FILTER status is not applicable to these candidate classes.

Non-PASS Mutect2 calls contributed minimally to the final prioritised candidate set. In the final prioritised tables, 1,737 of 2,288 candidates were somatic variant-derived, while 551 of 2,288 were fusion- or splicing-derived candidates for which Mutect2 FILTER status was not applicable. Among the somatic candidates, 1,714 of 1,737 were derived from PASS variants and 23 of 1,737 were derived from non-PASS variants, corresponding to 98.68% and 1.32% of somatic final epitopes, respectively. Non-PASS somatic candidates represented only 1.01% of all final prioritised epitopes.

We next assessed whether non-PASS variants contributed to the highest-ranked candidates. Across the top 10 ranked candidates per sample, only one somatic epitope was derived from a non-PASS variant, corresponding to 0.34% of all top-10 candidates and 0.54% of somatic top-10 candidates. Thus, non-PASS Mutect2 calls made a negligible contribution to the highest-priority epitope set.

To test whether key prioritisation results were robust to PASS-only filtering, we repeated the prioritisation using PASS-only somatic pVACseq inputs while retaining the same fusion, splicing, network, and annotation layers. Driver-gene representation among top-10 candidates was highly similar between the complete analysis and the PASS-only sensitivity analysis, with 34 of 296 top-10 candidates in the complete analysis and 33 of 296 in the PASS-only analysis. These results indicate that the driver-gene enrichment reported in Figure 6B was not materially dependent on the inclusion of non-PASS Mutect2 calls.

**Size of pruned patient-specific functional interaction networks**

To provide a quantitative summary of the effective network size after pFIN pruning, we calculated the number of retained genes and edges, the size of the largest connected component, and the number of isolated genes for each patient-specific network. Across the 30 TCGA-SKCM samples, the filtered pFINs retained a median of 21,888 genes per sample [range: 21,327–23,136] from 23,446 VST-normalized genes, corresponding to a median retention of 93.4%. The pruned networks contained a median of 181,255 edges [range: 170,853–185,124]. After pruning, the networks consisted of a large connected core together with isolated vertices: the largest connected component contained a median of 16,414 genes [range: 15,869–17,667], representing 75.0% of retained genes, and contained nearly all retained edges. These results show that pFIN pruning preserves a large patient-specific interaction network while concentrating the effective connected structure into a major component used for downstream network-based prioritisation.

| **Samples** | **Original VST genes** | **Retained genes, median [min–max]** | **Retained genes** | **Retained genes, median % of VST** | **Edges, median [min–max]** | **LCC genes, median [min–max]** | **LCC genes, median % of retained** | **LCC edges, median [min–max]** | **Isolated genes, median** |
| --- | --- | --- | --- | --- | --- | --- | --- | --- | --- |
| 30 | 23,446 | 21,888 [21,327–23,136] | 21,657–22,066 | 93.4% | 181,255 [170,853–185,124] | 16,414 [15,869–17,667] | 75.0% | 181,243 [170,831–185,112] | 5,454 |

**Supplementary Table S2. Network-size summary of pruned patient-specific functional interaction networks across TCGA-SKCM samples.** The table reports cohort-level summary statistics for the pruned patient-specific functional interaction networks generated from 30 TCGA-SKCM samples. For each network, we quantified the number of retained genes, retained edges, largest connected component genes and edges, and isolated genes after filtering and pruning. Percentages are reported relative to the original 23,446 VST-normalized genes or to the retained pruned network, as indicated.

**Network stability and benchmark analysis of pFIN-derived centrality features**

To evaluate the stability of patient-specific functional interaction networks (pFINs) generated from the 30 TCGA-SKCM samples, we performed three complementary analyses. First, we assessed whether centrality ranks were sensitive to individual background samples using a leave-one-background-sample-out jackknife analysis. Second, we evaluated the consistency of top-ranked pFIN edge sets within each patient and across patients. Third, we benchmarked pFIN centrality ranks against larger TCGA-SKCM GDC-derived background cohorts to assess whether the centrality signal was preserved when the background cohort size was increased.

For the jackknife analysis, each target patient was retained while each of the other 29 samples was removed from the background cohort in turn. Patient-specific networks were then recomputed using the same Pearson-LIONESS framework as in the original workflow, resulting in 870 same-patient jackknife comparisons across the 30 samples. For each comparison, gene-level centrality ranks from the jackknife-derived network were compared with the corresponding ranks from the original full-cohort network. Rank stability was assessed using Spearman rank correlation, Kendall τ, top-1% overlap, and top-1% Jaccard similarity.

The jackknife analysis showed high stability of pFIN-derived centrality ranks (Supplementary Table S3). WCI showed a median Spearman rank correlation of 0.997 relative to the original full-cohort ranks, with a minimum Spearman correlation of 0.990. On the PPI/membrane-filtered networks used for downstream prioritisation, degree, strength, and LCI also showed high rank stability, with median Spearman correlations of 0.998, 0.997, and 0.996, respectively. Betweenness was more sensitive to jackknife perturbation, as expected for a shortest-path-based metric, but remained concordant with the original ranks, with a median Spearman correlation of 0.933 and a minimum of 0.862. Top-ranked genes were also largely preserved, with median top-1% overlap ranging from 91.8% to 95.0% across metrics.

| **Metric** | **Network level** | **N** | **Median Spearman ρ** | **Minimum Spearman ρ** | **Median Kendall τ** | **Top 1% median overlap** | **Top 1% minimum overlap** | **Top 1% median Jaccard** |
| --- | --- | --- | --- | --- | --- | --- | --- | --- |
| WCI | Full LIONESS network | 870 | 0.997 | 0.990 | 0.957 | 93.6% | 64.7% | 0.880 |
| Degree | PPI/membrane-filtered network | 870 | 0.998 | 0.994 | 0.984 | 95.0% | 79.5% | 0.905 |
| Strength | PPI/membrane-filtered network | 870 | 0.997 | 0.989 | 0.973 | 94.0% | 72.6% | 0.884 |
| LCI | PPI/membrane-filtered network | 870 | 0.996 | 0.987 | 0.970 | 94.0% | 72.6% | 0.884 |
| Betweenness | PPI/membrane-filtered network | 870 | 0.933 | 0.862 | 0.900 | 91.8% | 74.4% | 0.848 |

**Supplementary Table S3. Jackknife stability of pFIN-derived centrality ranks.** For each target patient, patient-specific networks were recomputed after removing each of the other 29 background samples, yielding 870 same-patient jackknife comparisons across the 30 TCGA-SKCM samples. For each comparison, gene-level centrality ranks from the jackknife network were compared with the corresponding ranks from the original full-cohort network. The table reports Spearman rank correlation, Kendall τ, top-1% overlap, and top-1% Jaccard similarity for WCI, degree, strength, LCI, and betweenness.

To assess edge-level consistency, we compared top-ranked pFIN edge sets using Jaccard similarity and overlap relative to the smaller top-edge set (Supplementary Table S4). Same-patient comparisons between original and jackknife-derived networks showed stable top-edge sets, with median Jaccard values of 0.784, 0.800, 0.808, and 0.839 for the top 1%, 5%, 10%, and 20% of edges, respectively. These corresponded to median overlaps of 88.0% to 91.3% relative to the smaller top-edge set. In contrast, pairwise comparisons between the original pFINs from different patients showed very low overlap of top-ranked edges, with median Jaccard values of 0.000 for the top 1-10% edge sets and 0.005 for the top 20% edge set. These results indicate that top-ranked edges are stable within a patient under jackknife perturbation, while the highest-ranked edge structures remain largely patient-specific rather than being uniformly shared across all samples.

| **Analysis** | **Top edge fraction considered** | **Median Jaccard** | **Median overlap** |
| --- | --- | --- | --- |
| Same patient: original vs jackknife | 1% | 0.784 | 88.0% |
| Same patient: original vs jackknife | 5% | 0.800 | 88.9% |
| Same patient: original vs jackknife | 10% | 0.808 | 89.4% |
| Same patient: original vs jackknife | 20% | 0.839 | 91.3% |
| Between patients: original pFINs | 1% | 0.000 | 0.0% |
| Between patients: original pFINs | 5% | 0.000 | 0.0% |
| Between patients: original pFINs | 10% | 0.000 | 0.0% |
| Between patients: original pFINs | 20% | 0.005 | 0.9% |

**Supplementary Table S4. Edge consistency of pFIN top-ranked edge sets.** Top-ranked edge sets were compared using Jaccard similarity and overlap relative to the smaller top-edge set. Same-patient comparisons evaluate the stability of top edges between the original pFIN and jackknife-derived pFINs. Between-patient comparisons evaluate the overlap of top-ranked edges across the 30 original patient pFINs. The results distinguish within-patient edge stability from shared edge structure across different patients.

We next performed two complementary TCGA-SKCM GDC-based benchmark analyses. The first benchmark used public GDC STAR-count RNA-seq data restricted to TCGA-SKCM primary-tumor samples with available gene-level counts. In this matched public-data benchmark, the same 30 target-sample networks were reconstructed from public GDC data and then compared with networks generated after embedding those target samples in a larger n = 103 TCGA-SKCM primary-tumor background. This analysis was designed to evaluate the effect of increasing the background cohort size while keeping the data source and preprocessing framework consistent.

In the matched n = 103 GDC benchmark, WCI ranks were strongly concordant between the n = 30 and n = 103 backgrounds, with a median Spearman correlation of 0.942 and a minimum Spearman correlation of 0.910 (Supplementary Table S5). On the PPI/membrane-filtered networks, degree, strength, and LCI also showed high concordance, with median Spearman correlations of 0.950, 0.937, and 0.929, respectively. Betweenness showed lower concordance, with a median Spearman correlation of 0.675, consistent with its greater sensitivity to changes in shortest-path structure. Top-gene overlap was lower at the exact top-1% boundary but increased for broader high-ranked gene sets, supporting broad stability of the centrality signal under a larger TCGA-derived primary-tumor background while indicating that the most extreme top-ranked genes remain partly dependent on the background cohort.

As a second benchmark, we performed an extended GDC analysis using a larger TCGA-SKCM background of 469 samples. This analysis evaluated the effect of replacing the smaller n = 30 background with a much larger TCGA-derived background, restricting the comparison to genes common to both network reconstructions. In this n = 469 benchmark, WCI showed substantial concordance between the n = 30 and n = 469 backgrounds, with a median Spearman correlation of 0.824 (Supplementary Table S6). Degree and strength also showed moderate-to-substantial concordance, with median Spearman correlations of 0.771 and 0.772, respectively, while betweenness showed moderate concordance (ρ = 0.707). LCI was more sensitive to the larger background cohort, with a median Spearman correlation of 0.465, suggesting that this metric is more dependent on the inferred network background. As in the n = 103 benchmark, broader high-ranked gene sets were more stable than the exact top-1% boundary. At the top-20% threshold, median overlap increased to 74.8% for WCI, 77.7% for degree, 76.2% for strength, 78.2% for LCI, and 67.5% for betweenness.

Together, these analyses indicate that pFIN-derived centrality features are robust to leave-one-sample perturbation of the n = 30 background cohort, preserve stable same-patient edge structure, and show concordance with larger TCGA-SKCM GDC-derived background networks. The n = 103 benchmark provides a controlled comparison within public primary-tumor GDC data, while the n = 469 benchmark provides a broader cohort-size stress test. These results support the stability of the main network centrality signals used by EGG, while also indicating that the most extreme top-ranked genes and some metrics, particularly LCI and betweenness, remain more sensitive to background cohort composition.

| **Metric** | **Median Spearman ρ** | **Minimum Spearman ρ** | **Median Kendall τ** | **Top 1% median overlap** | **Top 1% minimum overlap** | **Top 1% median Jaccard** |
| --- | --- | --- | --- | --- | --- | --- |
| WCI | 0.942 | 0.910 | 0.786 | 56.7% | 42.4% | 0.365 |
| Degree | 0.950 | 0.935 | 0.890 | 72.7% | 63.1% | 0.569 |
| Strength | 0.937 | 0.917 | 0.850 | 61.5% | 48.1% | 0.444 |
| LCI | 0.929 | 0.912 | 0.840 | 61.5% | 47.7% | 0.444 |
| Betweenness | 0.675 | 0.619 | 0.616 | 63.4% | 53.3% | 0.465 |

**Supplementary Table S5. Matched GDC benchmark comparing pFIN centrality ranks generated using n = 30 and n = 103 TCGA-SKCM primary-tumor backgrounds.** The 30 target-sample networks were reconstructed using public GDC STAR-count RNA-seq data and compared with corresponding networks generated after embedding the same target samples in a larger TCGA-SKCM primary-tumor background of 103 samples. This benchmark was designed to evaluate the effect of increasing the background cohort size while keeping the data source and preprocessing framework consistent. The table reports rank concordance and top-gene overlap metrics for WCI, degree, strength, LCI, and betweenness.

| **Metric** | **Median Spearman ρ** | **Minimum Spearman ρ** | **Median Kendall τ** | **Top 1% median overlap** | **Top 1% minimum overlap** | **Top 1% median Jaccard** | **Top 20% median overlap** | **Top 20% median Jaccard** |
| --- | --- | --- | --- | --- | --- | --- | --- | --- |
| WCI | 0.824 | 0.686 | 0.628 | 52.6% | 30.4% | 0.357 | 74.8% | 0.597 |
| Degree | 0.771 | 0.700 | 0.623 | 73.2% | 57.4% | 0.578 | 77.7% | 0.635 |
| Strength | 0.772 | 0.702 | 0.594 | 66.5% | 52.8% | 0.498 | 76.2% | 0.616 |
| LCI | 0.465 | 0.285 | 0.456 | 43.5% | 30.8% | 0.278 | 78.2% | 0.642 |
| Betweenness | 0.707 | 0.587 | 0.582 | 57.4% | 46.1% | 0.403 | 67.5% | 0.510 |

**Supplementary Table S6. Extended GDC benchmark comparing pFIN centrality ranks generated using n = 30 and n = 469 TCGA-SKCM backgrounds.** The 30 target-sample networks were compared with corresponding networks generated using a larger TCGA-SKCM background of 469 samples, restricting the analysis to genes common to both network reconstructions. This benchmark evaluates the stability of pFIN-derived centrality ranks under a substantially larger TCGA-derived background. The table reports rank concordance and top-gene overlap metrics for WCI, degree, strength, LCI, and betweenness, including top-1% and top-20% overlap statistics.

**Impact of membrane-edge expansion on epitope prioritisation**

To evaluate the empirical impact of the membrane-edge expansion step, we quantified the representation of membrane-associated source genes among the final prioritised epitope candidates and performed an ablation analysis comparing the original PPI + membrane-expanded pFIN ranking with a PPI-only network ranking. Membrane-associated genes were defined using the same membrane-gene set used during pFIN expansion.

Across the 30 TCGA-SKCM samples, membrane-associated genes accounted for a median of 22.3% of all candidate epitopes. Among top-ranked candidates, the median membrane-associated fraction was 20.0% for the top 10 epitopes, 20.0% for the top 20 epitopes, and 22.0% for the top 50 epitopes. The corresponding median numbers of membrane-associated epitopes were 2, 4, and 10, respectively. These values were similar when the ranking was recomputed without membrane-edge expansion, indicating that membrane-derived epitopes were present among prioritised candidates but were not globally overrepresented relative to the full candidate pool (Supplementary Table S7).

To directly test whether membrane-edge expansion materially altered final prioritisation, we recomputed the final weighted Borda ranking using the same feature weights and ranking directions as in the original workflow, but replaced the PPI + membrane-expanded pFIN metrics with metrics calculated from PPI-filtered networks before membrane-edge addition. Non-ablated features, including binding affinity, DepMap survivability, IntOGen annotations, and other unchanged ranking inputs, were kept constant. As a validation step, we first recomputed the original weighted Borda ranking from the final feature columns; this reproduced the original ranks closely, with a median Spearman correlation of 0.9998 and a median Kendall τ of 0.993 across patients.

Removing membrane-edge expansion had only a modest effect on the final ranking. The original PPI + membrane-expanded ranking and the PPI-only ablation ranking showed high concordance, with a median Spearman correlation of 0.979, minimum Spearman correlation of 0.885, and median Kendall τ of 0.901. Top-ranked candidates were also largely preserved, with a median top-10 overlap of 9/10 epitopes and a median top-20 overlap of 18/20 epitopes. The number of membrane-derived epitopes in the top-ranked sets was essentially unchanged, with a median of 2 membrane-derived epitopes in the top 10 and 4 in the top 20 in both the original and PPI-only rankings.

Although the global ranking impact was limited, membrane-edge expansion had the intended local effect in selected cases. Across 2,288 candidate epitopes in the cohort, 523 were derived from membrane-associated genes. Among these, 236 membrane-derived epitopes across 25 patients showed some upward rank shift when membrane-edge expansion was included. More substantially, 78 membrane-derived epitopes across 17 patients improved by at least 5 rank positions, and 26 epitopes across 9 patients improved by at least 10 positions. In 8 patients, membrane-edge expansion moved at least one membrane-derived epitope into the top 10, and in 12 patients it moved at least one membrane-derived epitope into the top 20. These results indicate that membrane-edge expansion acts as a limited, targeted network augmentation step that can promote selected membrane-associated candidates, while leaving the overall prioritisation largely stable.

| **Cutoff** | **Background membrane** | **Mean membrane count, original ranking** | **Mean membrane count, original ranking without membrane enhancement** | **Top membrane % median** |
| --- | --- | --- | --- | --- |
| Top 10 epitopes | 22.3% | 2 | 2 | 20.0% |
| Top 20 epitopes | 22.3% | 4 | 4 | 20.0% |
| Top 50 epitopes | 22.3% | 10 | 10 | 22.0% |

**Supplementary Table S7. Representation of membrane-associated epitopes among top-ranked candidates with and without membrane-edge expansion.**The table reports the median fraction and number of membrane-associated epitopes among top-ranked candidates across the 30 TCGA-SKCM samples. Membrane-associated source genes were defined using the same membrane-gene set used for pFIN membrane-edge expansion. The “Background membrane” column indicates the median membrane-associated fraction in the full candidate pool. The table compares the original PPI + membrane-expanded pFIN ranking with the PPI-only ablation ranking, in which pFIN metrics were recalculated before membrane-edge addition while other ranking inputs were held constant

**Peptide length and final prioritisation rank**

To evaluate whether EGG prioritisation was influenced by peptide length, we compared peptide length distributions before and after prioritisation and tested the association between peptide length and final prioritisation rank. Because MHC class I and MHC class II prediction workflows use different peptide length ranges and binding models, all analyses were performed separately for MHC class I and MHC class II candidates.

Peptide length distributions were similar between the full candidate pool and the top 10 prioritised candidates per sample. For MHC class I candidates, the median peptide length was 11 amino acids in both the full candidate pool and the top 10 prioritised set, with no significant difference between top-ranked and non-top-ranked candidates (Mann-Whitney U test, p-value = 0.246). For MHC class II candidates, the median peptide length was 24 amino acids in the full candidate pool and 25 amino acids among the top 10 prioritised candidates, again with no significant difference between groups (p = 0.362). We also assessed whether peptide length correlated with final rank percentile. Using MHC class-specific rank percentiles, peptide length showed weak and non-significant correlations with prioritisation rank for both MHC class I candidates (Spearman ρ = -0.031, p-value = 0.313) and MHC class II candidates (ρ = 0.021, p-value = 0.467). Similar results were obtained when using overall rank percentiles across each sample.

Together, these analyses indicate that occasional long peptides among highly ranked candidates do not reflect a systematic tendency of EGG to prioritise longer peptide sequences. Instead, peptide length does not appear to be a major driver of final prioritisation rank in this analysis.

**Supplementary Figure S2. Peptide length distribution and association with final prioritisation rank.**
(A) Peptide length distribution before and after prioritisation, shown separately for MHC class I and MHC class II candidates. “All” indicates the full candidate pool before final top-ranked selection, while “Top 10” indicates the top 10 prioritised candidates per sample. Differences between top-ranked and non-top-ranked candidates were evaluated using the Mann-Whitney U test. (B) Relationship between peptide length and final prioritisation rank percentile, shown separately for MHC class I and MHC class II candidates. Spearman correlation was used to assess whether peptide length was associated with final rank. The analyses showed no significant enrichment of longer peptides among top-ranked candidates and no meaningful correlation between peptide length and final prioritisation rank.

**VAF-based clonality assessment of somatic epitope candidates**

o assess whether top-ranked somatic epitope candidates preferentially originated from variants compatible with clonal or near-clonal events, we examined tumor DNA variant allele frequency (VAF) values reported in the pVACseq-derived candidate tables. Tumor DNA VAF was used as a simplified proxy for clonality because formal cancer cell fraction estimation would require sample-specific correction for tumor purity, local copy number, and allelic imbalance. Under a conservative approximation assuming tumor purity of 1, variants with tumor DNA VAF ≥ 0.25 were considered broadly compatible with clonal or near-clonal mutations, whereas variants below this threshold would be more compatible with subclonal events.

Tumor DNA VAF values were matched for all final somatic/genomic variant-derived epitope candidates included in the prioritised output tables (1,737/1,737). All matched somatic candidates had tumor DNA VAF values ≥ 0.25, including all 185 somatic candidates present among the top 10 ranked epitopes per sample. Thus, this dataset did not contain a meaningful low-VAF/subclonal subset among the final somatic candidates, limiting the possibility of testing enrichment for clonal versus subclonal epitopes after prioritisation.

We next tested whether higher-ranked somatic epitopes were associated with higher tumor DNA VAF values. The median tumor DNA VAF was highly similar between the full somatic candidate set and the somatic candidates appearing within the top 10 overall ranked epitopes per sample (0.383 versus 0.385, Mann–Whitney p-value = 0.935). Tumor DNA VAF also showed no significant correlation with final rank percentile across all somatic candidates (Spearman ρ = -0.020, p-value = 0.411). Similar results were observed when MHC classes were analysed separately, with no significant association between tumor DNA VAF and rank percentile for either MHC class I candidates (ρ = -0.007, p-value = 0.839) or MHC class II candidates (ρ = -0.032, p-value = 0.335). These results indicate that, in this dataset, EGG prioritisation was not strongly driven by tumor DNA VAF as a simplified clonality proxy. Instead, the final somatic candidate pool was already restricted to variants with VAF values compatible with clonal or near-clonal events under the approximation used here.

**Supplementary Figure S3. Tumor DNA VAF distribution and association with final prioritisation rank among somatic epitope candidates.** (A) Tumor DNA variant allele frequency (VAF) distribution among all final somatic/genomic variant-derived epitope candidates compared with somatic candidates present among the top 10 ranked epitopes per sample. Tumor DNA VAF values were obtained from the pVACseq-derived candidate tables and were matched to all final somatic epitope candidates. The dashed threshold at VAF = 0.25 indicates the simplified cutoff used to classify variants as broadly compatible with clonal or near-clonal events under an assumed tumor purity of 1. (B) Relationship between tumor DNA VAF and final prioritisation rank percentile for somatic epitope candidates, shown separately for MHC class I and MHC class II candidates. Spearman correlation was used to test whether tumor DNA VAF was associated with final prioritisation rank. No significant difference in VAF distribution was observed between all somatic candidates and top-10 somatic candidates, and no significant correlation was observed between VAF and final rank percentile.

**PPI/topology ablation analysis**

To evaluate whether the PPI/topology-derived component of the pFIN contributed materially to epitope prioritisation, we performed an ablation analysis in which topology-derived network metrics were removed from the final Borda ranking. This analysis was designed to test whether the PPI-filtered network layer had a measurable effect on candidate ordering and on the composition of the highest-priority epitope shortlist.

In this ablation, Betweenness, Degree, LCI, and Strength were removed from the ranking, while MHC-IC50, DepMap survivability score, and WCI were retained. WCI was retained because it captures patient-specific co-expression network information rather than direct PPI/topology-derived structure. To isolate the effect of removing the PPI/topology metrics while maintaining the overall balance between antigen-prediction, essentiality, and patient-specific network context, the weight previously assigned to the removed topology metrics was reassigned to WCI.

The PPI/topology-ablated ranking remained only moderately concordant with the original ranking. Across the 30 TCGA-SKCM samples, the median Spearman correlation between the original and ablated rankings was 0.79, with an interquartile range of 0.72–0.86. The median top-10 overlap was 7/10 candidates, with an interquartile range of 6–8/10, and the median absolute rank shift was 5 positions. These results indicate that PPI/topology-derived network metrics have a measurable effect on candidate ordering and on the composition of the highest-priority shortlist. Thus, the PPI-filtered topology layer is not redundant with the other ranking features and contributes to the final prioritisation outcome.

**Supplementary Figure S4. Effect of removing PPI/topology-derived metrics from the final Borda ranking.** (A) Distribution of sample-level Spearman correlations between the original ranking and the PPI/topology-ablated ranking across the 30 TCGA-SKCM samples. (B) Distribution of top-10 overlap counts between the original ranking and the PPI/topology-ablated ranking. In this ablation, Betweenness, Degree, LCI, and Strength were removed, while MHC-IC50, DepMap survivability score, and WCI were retained. The weight previously assigned to the removed topology metrics was reassigned to WCI to preserve the overall balance between antigen-prediction, essentiality, and patient-specific network context. The moderate rank concordance and partial top-10 overlap indicate that PPI/topology-derived metrics have a measurable impact on final prioritisation.

**Borda-weight sensitivity analysis**

To evaluate whether the default Borda weighting scheme was overly dependent on any single information layer, and to assess whether correlated network metrics could inadvertently overweight the network component, we performed a sensitivity analysis across the 30 TCGA-SKCM samples. The analysis used the features included in the default Borda ranking: MHC-IC50, DepMap survivability score, and five network-derived metrics, namely Net_Betweenness, Net_Degree, Net_Impact/LCI, Net_Strength, and Net_WCI. HLA expression was not included in this sensitivity analysis because it was not part of the default Borda score in the presented case study.

We recomputed final epitope rankings under multiple alternative weighting scenarios and compared each alternative ranking with the default ranking. These scenarios included MHC-IC50-only, DepMap-only, network-only, no-network, no-MHC-IC50, no-DepMap, leave-one-network-metric-out analyses, and a network-weight sweep in which the total contribution of network-derived features was varied from 0% to 100%. Ranking similarity was evaluated using Spearman correlation across the full ranked candidate list, as well as top-10 and top-20 overlap with the default ranking.

The sensitivity analysis showed that the default ranking was not simply reproduced by any single input feature. The MHC-IC50-only ranking differed substantially from the default ranking, with a median Spearman correlation of 0.45 and median top-10 overlap of 0.40. The DepMap-only ranking also differed from the default, with a median Spearman correlation of 0.55 and median top-10 overlap of 0.45. A network-only ranking showed greater similarity to the default than either single non-network feature alone, with a median Spearman correlation of 0.80, but still did not fully reproduce the default ordering. Conversely, removing the full network component altered the ranking, with a median Spearman correlation of 0.66 and median top-10 overlap of 0.50. These results indicate that MHC binding affinity, gene essentiality, and network context each contribute to the final prioritisation, rather than the ranking being determined by one feature class alone.

We next evaluated whether the ranking was sensitive to moderate changes in the total contribution of the network layer. When the total network contribution was reduced to 25%, the median Spearman correlation with the default ranking was 0.88 and the median top-10 overlap was 0.70. When the total network contribution was increased to 75%, the median Spearman correlation was 0.91 and the median top-10 overlap was also 0.70. Thus, changing the relative weight of the network layer affected candidate ordering, as expected, but moderate perturbations did not completely reshuffle the highest-priority candidates.

To address feature redundancy among network metrics, we also examined leave-one-network-metric-out scenarios. Removing individual network metrics one at a time produced high full-rank similarity to the default ranking, with median Spearman correlations ranging from 0.983 to 0.995. The top-ranked candidate sets were also largely preserved, with median top-10 overlap ranging from 0.90 to 1.00 and median top-20 overlap approximately 0.95. These results indicate that individual network metrics contain partially overlapping information, consistent with the correlations observed among centrality-derived features, but that no single network metric alone dominates the final Borda ranking.

Together, these analyses support the use of the default Borda weighting scheme as a balanced and user-adjustable prioritisation strategy. The ranking is sensitive to the inclusion or removal of major biological information layers, particularly MHC-IC50, DepMap survivability, and the full network block, but it is not driven by a single feature or one individual centrality metric.

**Supplementary Figure S5. Sensitivity of final Borda ranking to alternative feature-weighting scenarios.** (A) Leave-one-network-metric-out full-rank similarity with the default Borda ranking. Bars show the median Spearman correlation across the 30 TCGA-SKCM samples after removing one network metric at a time. (B) Leave-one-network-metric-out overlap with the default ranking for the top 10 and top 20 candidates. Bars show the median overlap across samples. (C) Feature- and block-level sensitivity analysis showing median Spearman correlation between the default Borda ranking and alternative ranking scenarios, including MHC-IC50-only, DepMap-only, network-only, no-network, no-MHC-IC50, no-DepMap, and leave-one-network-metric-out scenarios. Blue bars indicate feature/block-level sensitivity analyses, while orange bars indicate leave-one-network-metric-out analyses. Error bars indicate variability across samples. (D) Distribution of top-10 overlap with the default ranking under selected alternative weighting scenarios. (E) Effect of changing the total network weight from 0% to 100% on similarity to the default ranking, measured by Spearman correlation and top-10 overlap. Overall, the analyses show that the default ranking is not reproduced by any single feature class, is affected by removal of major information layers, and remains relatively stable under moderate changes in total network weighting. Leave-one-network-metric-out analyses indicate that no individual centrality metric alone dominates the final ranking.

**External experimental evidence support for EGG-prioritised candidates**

To provide a bioinformatic validation of the EGG prioritisation framework, we assessed whether the final EGG/Borda ranking recovered candidates with independent experimental support more effectively than an MHC-binding-only ranking. Direct T-cell reactivity testing or MHC-eluted peptide validation would provide the strongest experimental evidence, but these assays were outside the scope of the present study. As a complementary computational analysis, we compared EGG-ranked candidates against external experimentally supported epitope evidence from IEDB, CEDAR, and TSNAdb v2.0.

External evidence tables were compiled from the IEDB, CEDAR, and TSNAdb v2.0 download/export resources. For IEDB and CEDAR, we used the full T-cell assay export (tcell_full_v3.csv) and the full MHC ligand export (mhc_ligand_full.csv). For TSNAdb v2.0, we used the collected validated neoantigen table (validated_tsnadb2_download.txt) together with the SNV-, INDEL-, and fusion-derived TSNAdb v2.0 tables. The corresponding database resources were accessed through the IEDB export page (<https://www.iedb.org/database_export_v3.php>), the CEDAR export page (<https://cedar.iedb.org/database_export_v3.php>), the CEDAR MHC ligand full export (<https://cedar.iedb.org/downloader.php?file_name=doc/mhc_ligand_full_single_file.zip>), and the TSNAdb v2.0 resource page (<https://pgx.zju.edu.cn/tsnadb/>).

The source tables were harmonised into normalized reference tables and scanned against EGG candidates using exact peptide matches and relaxed peptide-containment matches, with optional HLA and gene matching. For each patient, we compared the final EGG/Borda ranking with an IC50-only ranking and asked whether candidates ranked near the top by each method were enriched for external evidence of peptide presentation or immunogenicity. Evidence was stratified by database, evidence group, evidence class, and evidence strength.

To focus on high-confidence evidence, we retained only experimentally supported categories. Positive MHC ligand or antigen-presentation evidence from IEDB or CEDAR, together with TSNAdb tier 3 validated entries, was classified as presentation evidence. Positive T-cell assay evidence from IEDB or CEDAR, together with TSNAdb tier 2 validated entries, was classified as strong immunogenicity evidence. TSNAdb tier 1 validated entries were classified as combined strong immunogenicity and presentation evidence. Binding-only evidence, prediction-only evidence, negative assays, and uncategorised ligand records were excluded from the high-confidence experimentally supported evidence set. This filtering was chosen because the purpose of the analysis was to test whether EGG prioritisation recovered candidates with experimental evidence of peptide presentation and/or immune recognition, including T-cell reactivity, rather than candidates supported only by predicted or measured MHC binding affinity. This distinction is important because MHC binding is necessary for many neoantigen candidates but does not by itself demonstrate natural presentation or functional immunogenicity.

| **Harmonized category** | **Source evidence included** | **Rationale** |
| --- | --- | --- |
| presentation | Positive MHC ligand / antigen-presentation evidence from IEDB or CEDAR; TSNAdb tier 3 validated entries | Supports experimental peptide presentation or presentation-associated validation |
| strong immunogenicity | Positive T-cell assay evidence from IEDB or CEDAR; TSNAdb tier 2 validated entries | Supports functional immunogenicity / T-cell recognition |
| strong immunogenicity and presentation | TSNAdb tier 1 validated entries | Highest-confidence category combining immunogenicity and presentation/validation support |

**Supplementary Table S8. Harmonised experimentally supported evidence categories used for external epitope support analysis.** External evidence from IEDB, CEDAR, and TSNAdb was harmonised into presentation, strong immunogenicity, and combined strong immunogenicity plus presentation categories. Positive MHC ligand or antigen-presentation evidence from IEDB or CEDAR and TSNAdb tier 3 validated entries were classified as presentation evidence. Positive T-cell assay evidence from IEDB or CEDAR and TSNAdb tier 2 validated entries were classified as strong immunogenicity evidence. TSNAdb tier 1 validated entries were classified as combined strong immunogenicity and presentation evidence. Binding-only evidence, prediction-only evidence, negative assays, and uncategorised ligand records were excluded from the high-confidence experimentally supported evidence set.

We first evaluated exact peptide matching, requiring the full EGG candidate peptide to be identical to an experimentally supported peptide in the external reference databases. Exact matches were extremely rare. Only one EGG candidate exactly matched a validated peptide in IEDB/CEDAR, and no exact peptide match was observed against TSNAdb, despite the large number of unique experimentally supported reference peptides available in IEDB and CEDAR. This confirmed that exact full-length peptide matching was too sparse to support a meaningful comparison between ranking strategies.

We therefore used a relaxed min-8 containment match as the main analysis. Under this criterion, an EGG candidate was counted as externally supported if either the EGG candidate peptide contained the reference peptide or the reference peptide contained the EGG candidate peptide, requiring the contained sequence to be at least eight amino acids long. This approach was used because experimentally reported MHC ligands and T-cell epitopes often correspond to shorter presented or reactive peptide cores, whereas EGG candidate sequences can be longer, particularly for MHC class II or window-based predictions.

| **Database** | **Experimentally supported reference rows** | **Unique experimentally supported reference peptides** | **EGG candidates with exact peptide match** | **EGG candidates with relaxed min-8 peptide match** |
| --- | --- | --- | --- | --- |
| CEDAR | 4,346,554 | 1,223,992 | 1 | 542 |
| IEDB | 4,562,446 | 1,329,895 | 1 | 545 |
| TSNAdb_v2 | 1,854 | 1,666 | 0 | 5 |

**Supplementary Table S9. Experimentally supported external reference peptide sets and overlap with EGG candidates.** The table reports the number of experimentally supported reference rows and unique experimentally supported reference peptides obtained from CEDAR, IEDB, and TSNAdb, together with the number of EGG candidates matching each database by exact full-length peptide identity or relaxed min-8 containment matching. Exact matching required complete peptide identity between the EGG candidate and the reference peptide. Relaxed min-8 containment matching counted a candidate as supported when either the EGG candidate contained the reference peptide or the reference peptide contained the EGG candidate, with a minimum contained sequence length of eight amino acids.

Using the relaxed min-8 endpoint, the EGG/Borda ranking improved recovery of experimentally supported candidates relative to IC50-only ranking at the most selective cutoffs. In the top-N analysis, Borda showed a higher median hit rate than IC50-only ranking in the top 10 and top 20 candidates per patient, whereas differences became negligible at broader top-N cutoffs. In the top-percentile analysis, Borda also showed positive enrichment relative to IC50-only ranking at intermediate cutoffs, although the magnitude varied across thresholds and individual ranking features. These results indicate that EGG/Borda prioritisation can promote candidates containing experimentally supported presented or immunogenic peptide cores beyond MHC binding affinity alone.

This analysis should be interpreted as orthogonal database-supported evidence rather than direct functional validation. The relaxed min-8 containment strategy increases sensitivity for detecting shared presented or immunogenic peptide cores but does not prove that the full predicted EGG candidate is naturally processed, presented, or immunogenic in the corresponding patient. Nevertheless, the observation that Borda-ranked candidates recovered experimentally supported evidence more effectively than IC50-only ranking at stringent cutoffs supports the biological relevance of integrating binding affinity with gene-level and network-context features.

**Supplementary Figure S6. Recovery of experimentally supported external epitope evidence by EGG/Borda ranking compared with IC50-only ranking.** (A) Median difference in hit rate between each ranking method and IC50-only ranking across top-N candidate cutoffs within each patient, using relaxed min-8 containment matching against experimentally supported external evidence from IEDB, CEDAR, and TSNAdb. Positive values indicate higher recovery of externally supported candidates than IC50-only ranking. (B) Median difference in hit rate between each ranking method and IC50-only ranking across top-percentile cutoffs within each patient, using the same relaxed min-8 containment endpoint. Exact full-length peptide matching was also evaluated but was too sparse to support robust ranking comparisons. The relaxed min-8 analysis indicates that EGG/Borda ranking improves recovery of candidates containing experimentally supported presented or immunogenic peptide cores relative to IC50-only ranking at the most selective top-ranked cutoffs.

To assess whether the relaxed min-8 containment endpoint was influenced by peptide length, we performed an additional peptide-length-normalised coverage sensitivity analysis. For each relaxed containment match between an EGG candidate peptide and an experimentally supported external reference peptide from CEDAR, IEDB, or TSNAdb v2.0, we calculated the fraction of the longer peptide covered by the matched sequence. We then repeated the external-evidence comparison using min-8 containment alone and min-8 containment combined with ≥50%, ≥70%, and ≥80% longer-peptide coverage thresholds.

The original min-8 endpoint was reproduced exactly from the new match-level table, with no mismatches relative to the original candidate-level evidence flags. This confirmed that the coverage-sensitivity analysis was performed on the same relaxed-match endpoint used in the original ranking comparison. Increasing the required longer-peptide coverage substantially reduced database-specific support. CEDAR retained 542, 189, 40, and 14 candidates; IEDB retained 545, 192, 41, and 14 candidates; and TSNAdb v2.0 retained 5, 1, 0, and 0 candidates across the min-8, ≥50%, ≥70%, and ≥80% thresholds, respectively (Supplementary Figure S7).

We then repeated the ranking comparison using these progressively stricter coverage thresholds. The original relaxed min-8 endpoint produced the clearest recovery signal relative to IC50-only ranking at selective cutoffs, whereas this signal was attenuated under the ≥50% longer-peptide coverage threshold and became sparse at the ≥70% and ≥80% thresholds (Supplementary Figure S8). These results indicate that relaxed min-8 containment is useful for detecting shared experimentally supported peptide cores, but that it is sensitive to peptide-length-normalised stringency and should not be interpreted as full-length peptide validation.

**Supplementary Figure S7.** Effect of peptide-length-normalised coverage thresholds on database-specific external evidence support. The number of EGG candidates with experimentally supported external evidence is shown for each matching criterion, stratified by evidence database. Candidate support was assessed using the original relaxed min-8 containment endpoint and stricter criteria requiring the matched segment to cover at least 50%, 70%, or 80% of the longer peptide. Counts are shown separately for CEDAR, IEDB, and TSNAdb v2.0. Increasing the coverage threshold substantially reduced the number of supported candidates, indicating that relaxed min-8 containment should be interpreted as sensitive core-level support rather than full-length peptide validation.

**Supplementary Figure S8.** Effect of peptide-length-normalised coverage thresholds on ranking-based recovery of external evidence. Median difference in hit rate between each ranking method and IC50-only ranking is shown across top-N and top-percentile cutoffs within each patient. The analysis used experimentally supported peptide matches pooled across CEDAR, IEDB, and TSNAdb v2.0. The original relaxed min-8 containment endpoint produced the clearest recovery signal relative to IC50-only ranking at selective cutoffs. This signal was attenuated under the ≥50% longer-peptide coverage threshold and became sparse at the ≥70% and ≥80% thresholds, where median differences were zero. These results indicate that the relaxed min-8 endpoint captures shared experimentally supported peptide cores, however stricter full-length-like coverage thresholds leave too few supported candidates for robust ranking comparison.
