# Supplementary material for: Epitope Generation Gateway: A biological context-dependent approach to cancer vaccine development
Source: Gigascience. 2026 Oct 1;15:giag095. doi: 10.1093/gigascience/giag095 (PMC13679409; doi:10.1093/gigascience/giag095)
Supplement: Supplementary file 10 [file giag095_supplemental_files.zip › Supplementary Figure 1.pdf]

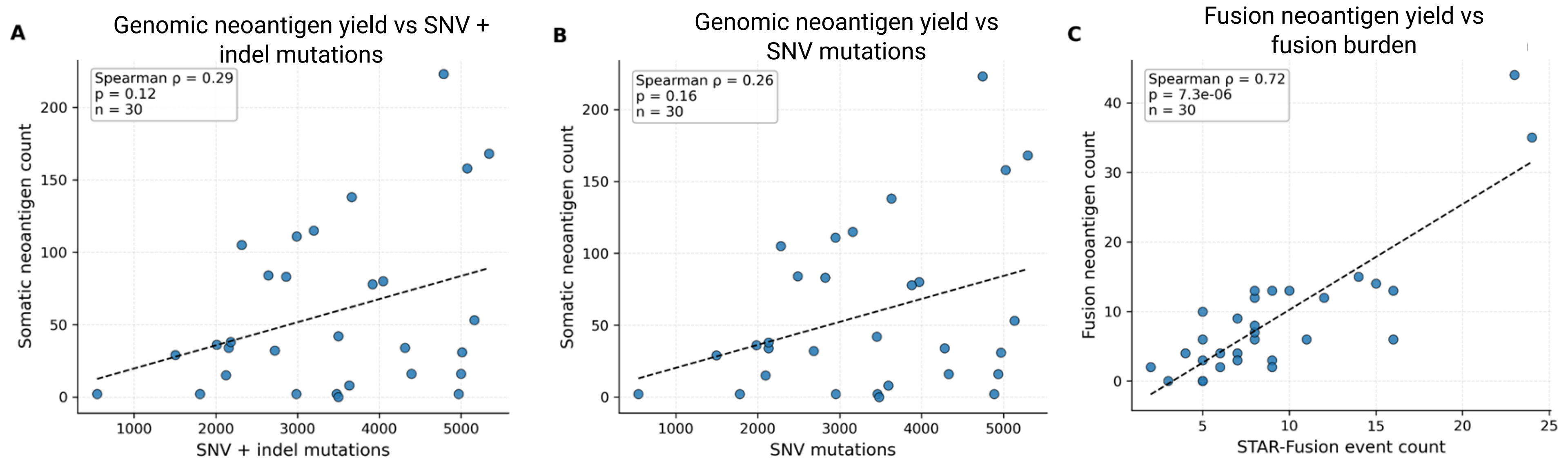
A
Genomic neoantigen yield vs SNV + indel mutations
Spearman ρ = 0.29
p = 0.12
n = 30
Somatic neoantigen count
SNV + indel mutations
B
Genomic neoantigen yield vs SNV mutations
Spearman ρ = 0.26
p = 0.16
n = 30
Somatic neoantigen count
SNV mutations
C
Fusion neoantigen yield vs fusion burden
Spearman ρ = 0.72
p = 7.3e-06
n = 30
Fusion neoantigen count
STAR-Fusion event count
