# Supplementary material for: Epitope Generation Gateway: A biological context-dependent approach to cancer vaccine development
Source: Gigascience. 2026 Oct 1;15:giag095. doi: 10.1093/gigascience/giag095 (PMC13679409; doi:10.1093/gigascience/giag095)
Supplement: Supplementary file 10 [file giag095_supplemental_files.zip › Supplementary Figure 2.pdf]

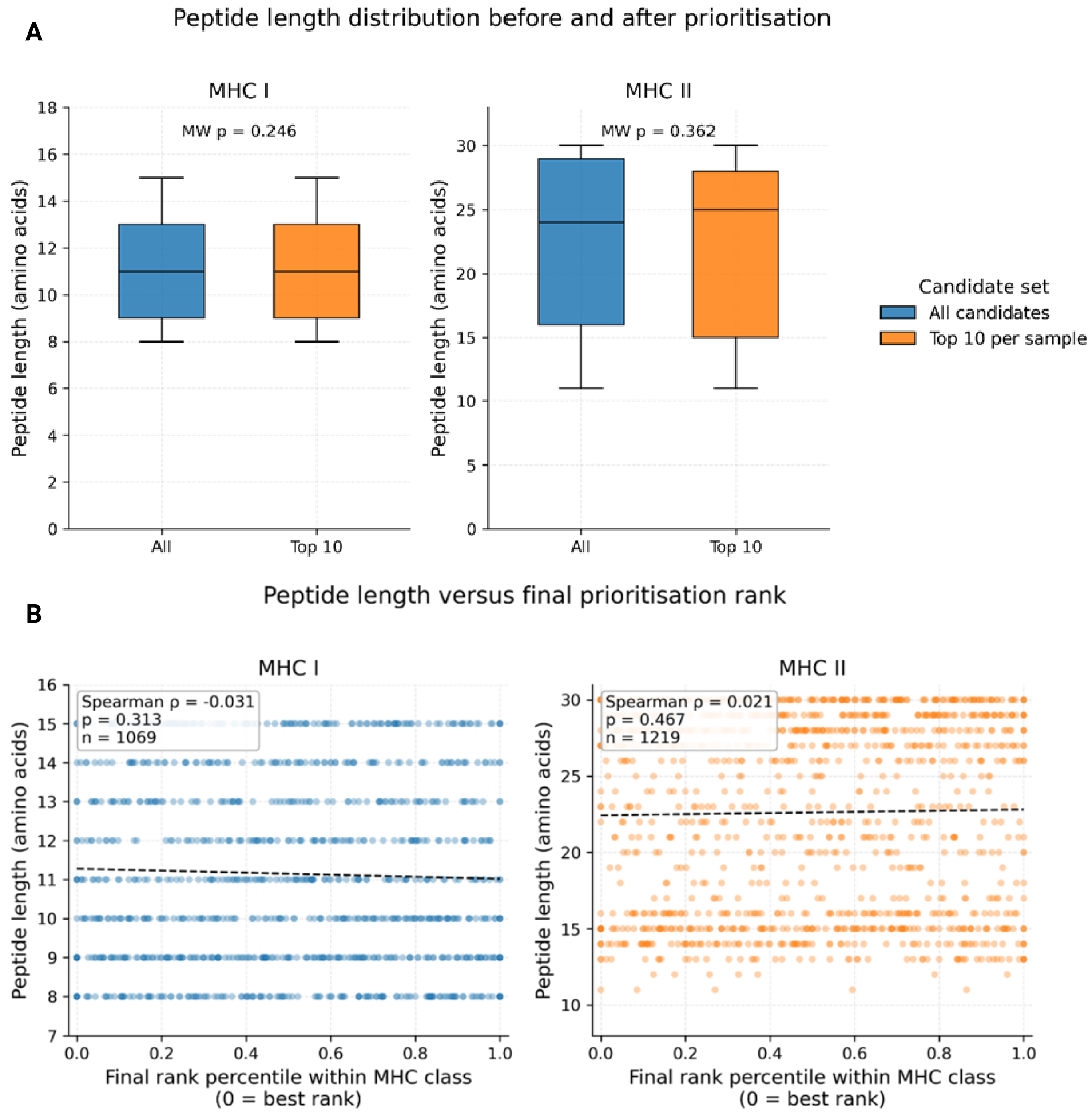
A
Peptide length distribution before and after prioritisation
MHC I
MW p = 0.246
MHC II
MW p = 0.362
Peptide length (amino acids)
All
Top 10
Candidate set
All candidates
Top 10 per sample
B
Peptide length versus final prioritisation rank
MHC I
Spearman ρ = -0.031
p = 0.313
n = 1069
MHC II
Spearman ρ = 0.021
p = 0.467
n = 1219
Final rank percentile within MHC class
(0 = best rank)
