# Supplementary material for: Epitope Generation Gateway: A biological context-dependent approach to cancer vaccine development
Source: Gigascience. 2026 Oct 1;15:giag095. doi: 10.1093/gigascience/giag095 (PMC13679409; doi:10.1093/gigascience/giag095)
Supplement: Supplementary file 10 [file giag095_supplemental_files.zip › Supplementary Figure 3.pdf]

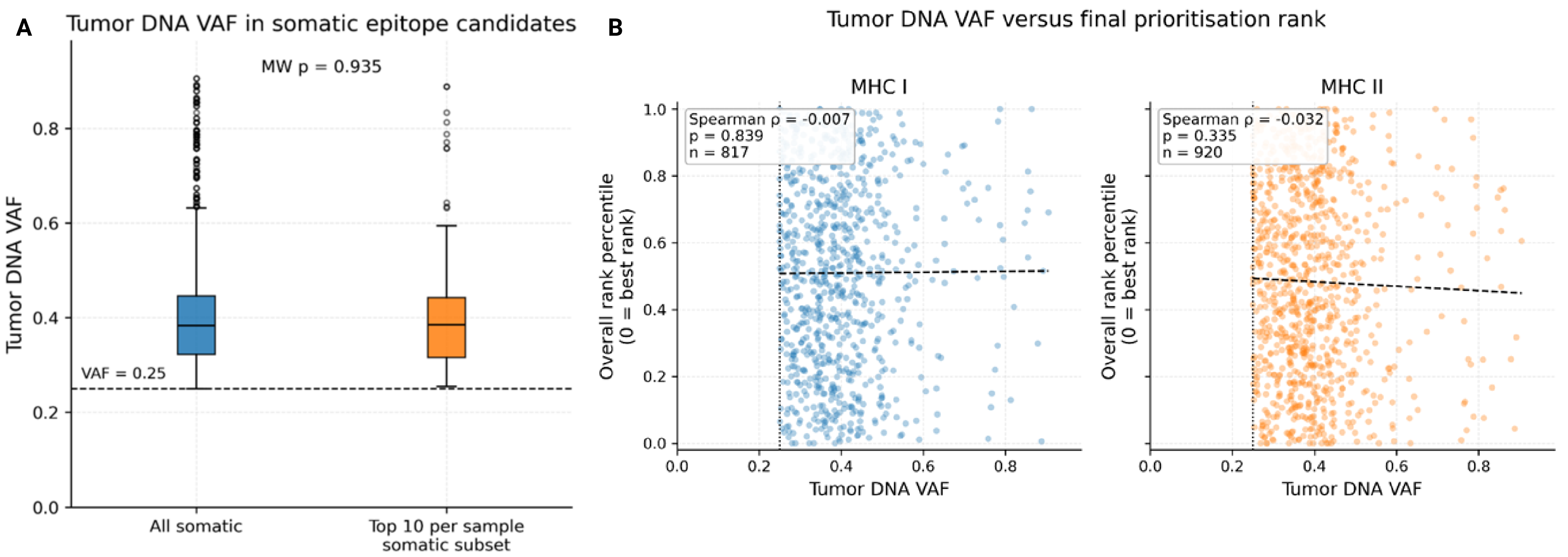
A
Tumor DNA VAF in somatic epitope candidates
MW p = 0.935
Tumor DNA VAF
VAF = 0.25
0.0
0.2
0.4
0.6
0.8
All somatic
Top 10 per sample somatic subset
B
Tumor DNA VAF versus final prioritisation rank
MHC I
Spearman ρ = -0.007
p = 0.839
n = 817
MHC II
Spearman ρ = -0.032
p = 0.335
n = 920
Overall rank percentile (0 = best rank)
Tumor DNA VAF
0.0
0.2
0.4
0.6
0.8
1.0
