# Supplementary material for: Epitope Generation Gateway: A biological context-dependent approach to cancer vaccine development
Source: Gigascience. 2026 Oct 1;15:giag095. doi: 10.1093/gigascience/giag095 (PMC13679409; doi:10.1093/gigascience/giag095)
Supplement: Supplementary file 10 [file giag095_supplemental_files.zip › Supplementary Figure 4.pdf]

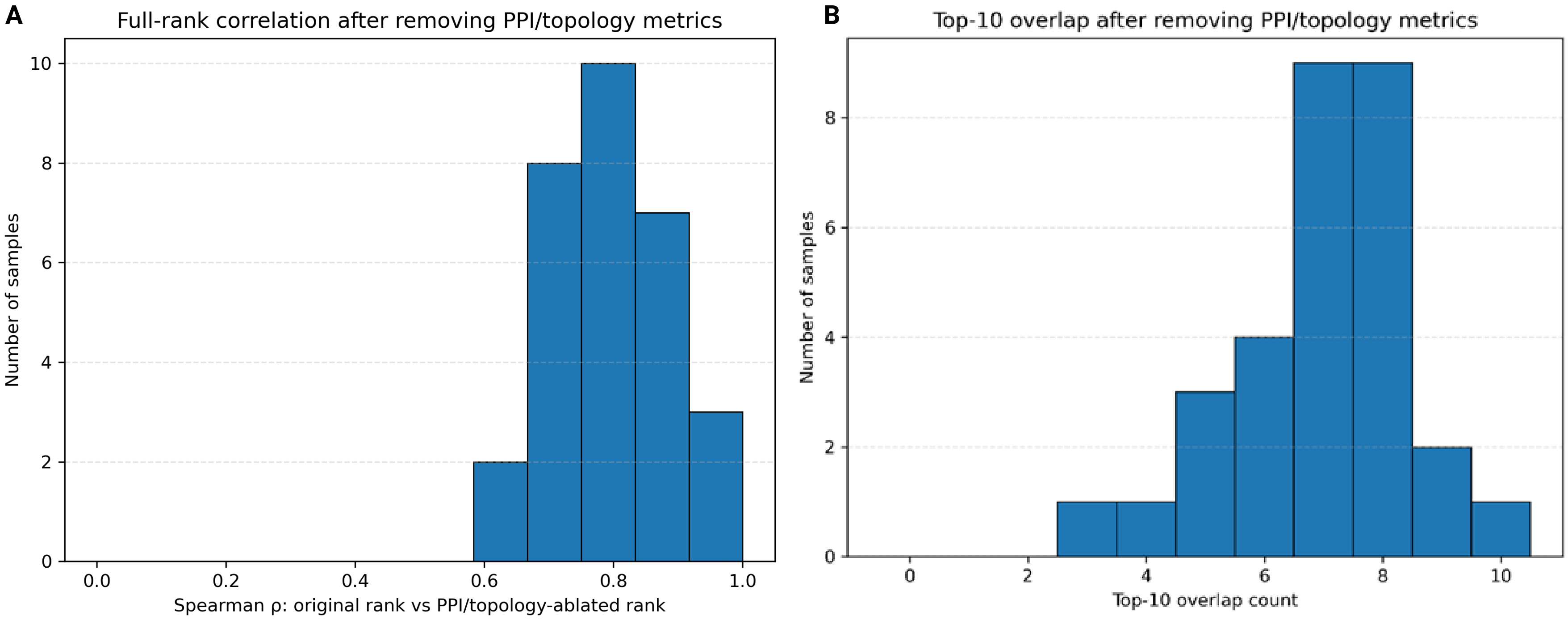
A
Full-rank correlation after removing PPI/topology metrics
Number of samples
0
2
4
6
8
10
0.0
0.2
0.4
0.6
0.8
1.0
Spearman ρ: original rank vs PPI/topology-ablated rank
B
Top-10 overlap after removing PPI/topology metrics
Number of samples
0
2
4
6
8
0
2
4
6
8
10
Top-10 overlap count
