# Supplementary material for: Epitope Generation Gateway: A biological context-dependent approach to cancer vaccine development
Source: Gigascience. 2026 Oct 1;15:giag095. doi: 10.1093/gigascience/giag095 (PMC13679409; doi:10.1093/gigascience/giag095)
Supplement: Supplementary file 10 [file giag095_supplemental_files.zip › Supplementary Figure 5.pdf]

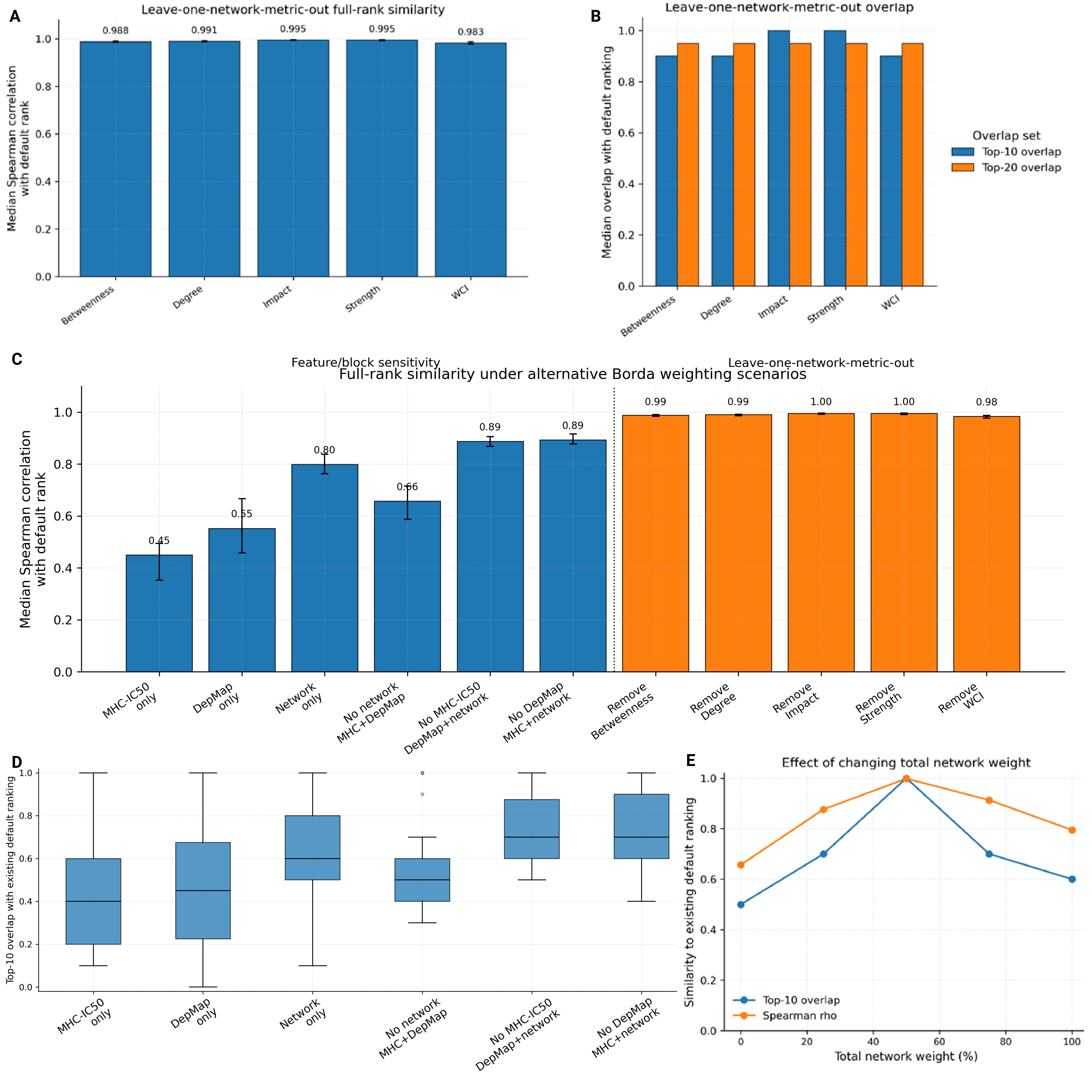

A
Leave-one-network-metric-out full-rank similarity
Median Spearman correlation with default rank
0.988
0.991
0.995
0.995
0.983
Betweenness
Degree
Impact
Strength
WCI
B
Leave-one-network-metric-out overlap
Median overlap with default ranking
Overlap set
Top-10 overlap
Top-20 overlap
Betweenness
Degree
Impact
Strength
WCI
C
Full-rank similarity under alternative Borda weighting scenarios
Feature/block sensitivity
Leave-one-network-metric-out
Median Spearman correlation with default rank
0.45
0.55
0.80
0.66
0.89
0.89
0.99
0.99
1.00
1.00
0.98
MHC-IC50 only
DepMap only
Network only
No network MHC+DepMap
No MHC-IC50 DepMap+network
No DepMap MHC+network
Remove Betweenness
Remove Degree
Remove Impact
Remove Strength
Remove WCI
D
Top-10 overlap with existing default ranking
MHC-IC50 only
DepMap only
Network only
No network MHC+DepMap
No MHC-IC50 DepMap+network
No DepMap MHC+network
E
Effect of changing total network weight
Similarity to existing default ranking
Top-10 overlap
Spearman rho
Total network weight (%)
