# Supplementary material for: Epitope Generation Gateway: A biological context-dependent approach to cancer vaccine development
Source: Gigascience. 2026 Oct 1;15:giag095. doi: 10.1093/gigascience/giag095 (PMC13679409; doi:10.1093/gigascience/giag095)
Supplement: Supplementary file 10 [file giag095_supplemental_files.zip › Supplementary Figure 7.pdf]

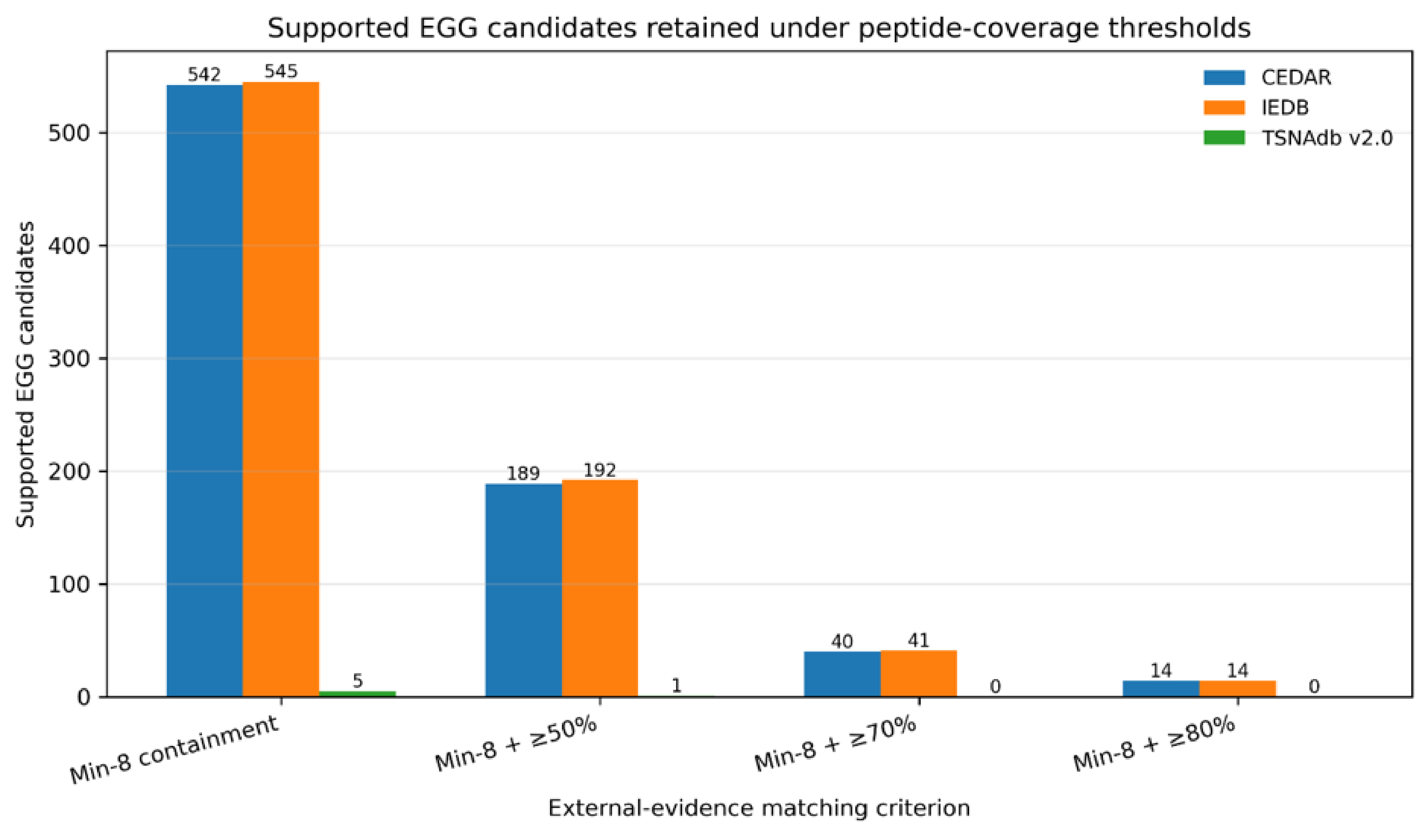
Supported EGG candidates retained under peptide-coverage thresholds
CEDAR
IEDB
TSNAdb v2.0
Supported EGG candidates
External-evidence matching criterion
Min-8 containment
Min-8 + ≥50%
Min-8 + ≥70%
Min-8 + ≥80%
542
545
5
189
192
1
40
41
0
14
14
0
0
100
200
300
400
500
