# Supplementary material for: Epitope Generation Gateway: A biological context-dependent approach to cancer vaccine development
Source: Gigascience. 2026 Oct 1;15:giag095. doi: 10.1093/gigascience/giag095 (PMC13679409; doi:10.1093/gigascience/giag095)

Effect of peptide-length-normalised coverage thresholds on ranking-based recovery of external evidence

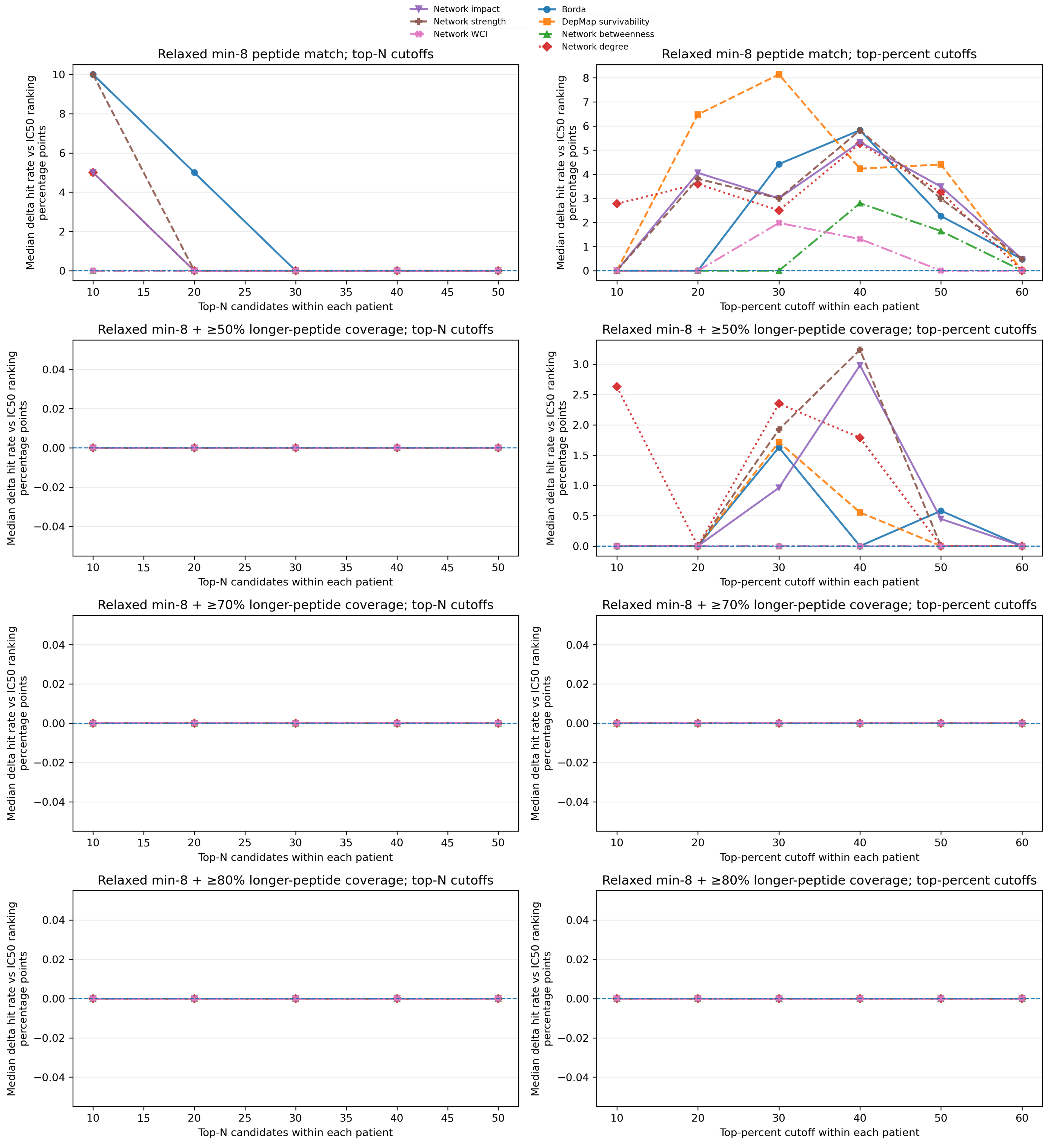

Supplement: Supplementary file 10 [file giag095_supplemental_files.zip › Supplementary Figure 8.pdf]
