# Supplementary material for: Epitope Generation Gateway: A biological context-dependent approach to cancer vaccine development
Source: Gigascience. 2026 Oct 1;15:giag095. doi: 10.1093/gigascience/giag095 (PMC13679409; doi:10.1093/gigascience/giag095)
Supplement: Supplementary file 10 [file giag095_supplemental_files.zip › Supplemetary Figure 6.pdf]

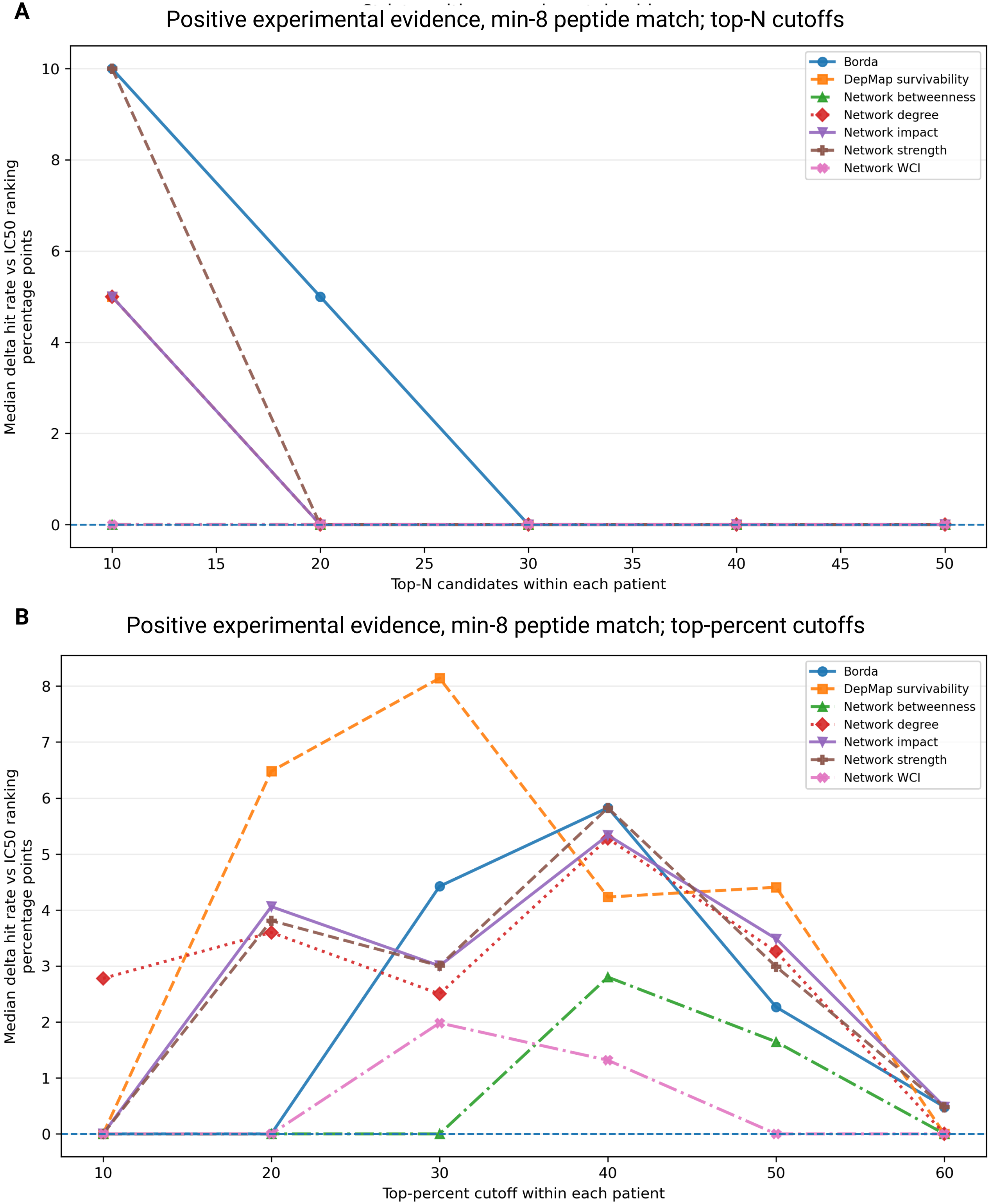
A
Positive experimental evidence, min-8 peptide match; top-N cutoffs
Median delta hit rate vs IC50 ranking percentage points
Top-N candidates within each patient
Borda
DepMap survivability
Network betweenness
Network degree
Network impact
Network strength
Network WCI
B
Positive experimental evidence, min-8 peptide match; top-percent cutoffs
Median delta hit rate vs IC50 ranking percentage points
Top-percent cutoff within each patient
Borda
DepMap survivability
Network betweenness
Network degree
Network impact
Network strength
Network WCI
